# Design, Synthesis, Biological Evaluation, and In Silico Studies of Novel Multitarget Cinnamic Acid Hybrids †

**DOI:** 10.3390/molecules30234582

**Published:** 2025-11-28

**Authors:** Ioanna-Chrysoula Tsopka, Eleni Pontiki, Ioanna Sigala, Eleni Nikolakaki, Kyriakos C. Prousis, Dimitra Hadjipavlou-Litina

**Affiliations:** 1 Department of Pharmaceutical Chemistry, School of Pharmacy, Faculty of Health Sciences, Aristotle University of Thessaloniki, 54124 Thessaloniki, Greece; joannatsopka@gmail.com (I.-C.T.); epontiki@pharm.auth.gr (E.P.); 2 Laboratory of Biochemistry, Department of Chemistry, Aristotle University of Thessaloniki, 54124 Thessaloniki, Greece; isigala@chem.auth.gr (I.S.); nikol@chem.auth.gr (E.N.); 3 Institute of Chemical Biology, National Hellenic Research Foundation, 48 Vassileos Constantinou Avenue, 11635 Athens, Greece; kyrprous@eie.gr

**Keywords:** chronic inflammation, LOX, COX, cinnamic acid, nitric oxide, NO donor, multitarget agents, hybrid molecules

## Abstract

Chronic inflammation is implicated in the development of various multifactorial diseases, including cancer, diabetes, arthritis, cardiovascular disorders, Alzheimer’s disease, and autoimmune diseases. The enzymes that play a key role in the onset of the inflammation are cyclooxygenases (COXs) and lipoxygenases (LOXs). In recent years, cinnamic acid hybrid molecules, particularly those incorporating a nitric oxide (NO) donor moiety, have attracted considerable attention as potential pharmacological agents for the treatment of multifactorial diseases. In the present study, novel cinnamic acid–nitric oxide (NO) donor hybrids were synthesized as multitarget agents and evaluated for their antioxidant, anti-inflammatory, and cytotoxic properties. In particular, hybrids **5a**–**i**, **6a**–**i**, **9a**–**i**, and **11** were synthesized and evaluated as lipid peroxidation and LOX inhibitors, while selected molecules were further tested as COX-1 and COX-2 inhibitors. Hybrids **6a**–**i**, **9a**–**i**, and **11** that contain a NO donor moiety, were additionally tested as albumin denaturation inhibitors and for their ability to release NO. The results indicated that compound **9a** is a promising multitarget agent, exhibiting the lowest IC_50_ for LOX inhibition, significant antioxidant activity, and the highest NO donor potency. Furthermore, compound **9e** demonstrated significant inhibitory activity against both COX-2 and LOX, suggesting its potential as a dual COX–LOX inhibitor. Additionally, compound **6i** exhibited the strongest cytotoxic activity among the tested compounds, with EC_50_ values ranging from 36 to 45 μM across multiple cancer cell lines. All synthesized compounds were also evaluated through in silico studies.

## 1. Introduction

Inflammation is the multifactorial defense mechanism of the body to stimuli of various etiologies (microbial, chemical, etc.) that can cause pathophysiological results. There are two main types of inflammation: acute and chronic. Acute inflammation is sudden and temporary, while chronic inflammation is a process of prolonged duration. Chronic inflammation can lead to a variety of multifactorial diseases such as cancer, diabetes, arthritis, cardiovascular, Alzheimer’s, and autoimmune diseases [1,2].

Important enzymes that play a key role in the mechanism of inflammation are cyclooxygenases (COXs) and lipoxygenases (LOXs). Cyclooxygenases metabolize arachidonic acid to produce prostaglandins, prostacyclins, and thromboxane A2, while lipoxygenases lead to leukotrienes [3,4].

Furthermore, reactive oxygen species (ROS) are also significant factors in the progress of inflammatory disorders acting as key signaling molecules. An enhanced ROS generation by polymorphonuclear neutrophils (PMNs) at the site of inflammation causes endothelial dysfunction and tissue injury. In addition, oxidative stress is viewed as an imbalance between the production of ROS and their elimination by protective mechanisms, which can lead to chronic inflammation. High levels of ROS can produce changes, e.g., in (OXPHOS), transition metal ions, oxidase activity, protein folding, thymidine, and polyamine catabolism, lipids, proteins, and DNA. It is consistent that rates of ROS production are increased in most diseases [5,6,7].

Nitric oxide (NO) seems to be important in the process of inflammation. It is a neurotransmitter that lowers blood pressure and is an essential reactive oxygen species. It is produced in endothelial cells from nitic oxide synthases (NOSs) by the conversion of L-arginine to L-citrulline and NO. Afterwards, NO enters adjacent cells and stimulates the enzyme soluble guanylate cyclase (sGC) that regulates various molecular targets that lead to a range of physiological responses [8]. Into the organism, NO reacts with oxygen to give nitric oxides. In pathological conditions, such as inflammation, there is low bioavailability of NO in the body due to high production of ROS. NO donors are the appropriate therapy in these cases. They can release NO into the body increasing the body’s NO levels in pathological conditions, used to treat cardiovascular disorders. Many well-known NO donors are organic nitrites, oximes, furoxans, NONOates, etc. [9,10].

On the other hand, cinnamic acid is a natural product found in essential oils, resins, and balsams, serving as a precursor to various hydroxy-substituted derivatives with notable biological activities. Synthetic modifications of its structure have led to the development of commercially significant molecules used in producing bioactive compounds and drugs. Substitutions on the cinnamic acid backbone result in derivatives with diverse biological properties, including anti-inflammatory, antioxidant, anticancer, and antimicrobial activities [11,12].

Today, there is an increase interest in the combination of two or more pharmacophores on the same scaffold, leadings to hybrid molecules. Hybrid drugs are produced with the goal of creating a chemical entity, more medically effective than its individual components. Furthermore, multifunctional molecules can be beneficial for the treatment of complex and multifactorial diseases instead of a single-target molecule [13]. A variety of multitarget molecules containing the cinnamic acid moiety have been reported.

Many efforts have been devoted to the synthesis of cinnamic hybrids in order to obtain multifunctional drugs [14]. Notably, hybrids incorporating nitric oxide (NO) donor groups have been synthesized and evaluated. The integration of NO donor groups into existing bioactive molecules has been shown to enhance their biological properties, thereby offering potential for improved therapeutic applications [15].

A considerable number of NO donors have been synthesized as hybrids combined with other pharmacophoric groups. Among them, oximes and furoxanes are of particular interest. Oximes represent one of the most common and widely recognized nitrogen-containing scaffolds, exhibiting diverse biological activities such as antibacterial, antifungal, anti-inflammatory, antioxidant, and anticancer effects [16,17,18,19]. In 1998, Koikov et al. [20] reported that oximes, amidoximes, and hydroxamic acids can act as NO donors, releasing NO and the corresponding parent ketones under mild oxidation conditions. Moreover, amidoximes and oximes exert significant physiological effects, including relaxation of both aortic and tracheal rings, as well as reduction of arterial pressure and thrombus formation.

Experimental studies have demonstrated that furoxan derivatives can activate soluble guanylate cyclase (sGC) by releasing nitric oxide in the presence of thiol cofactors. Since NO is involved in many bioregulatory processes, the furoxan scaffold represents a valuable structure for the design of hybrid molecules. Such hybrids could serve as novel vasodilators, combining typical NO-dependent effects on both the microvascular and macrovascular systems with additional α_1_-antagonist activity [21,22].

**Figure 1:**
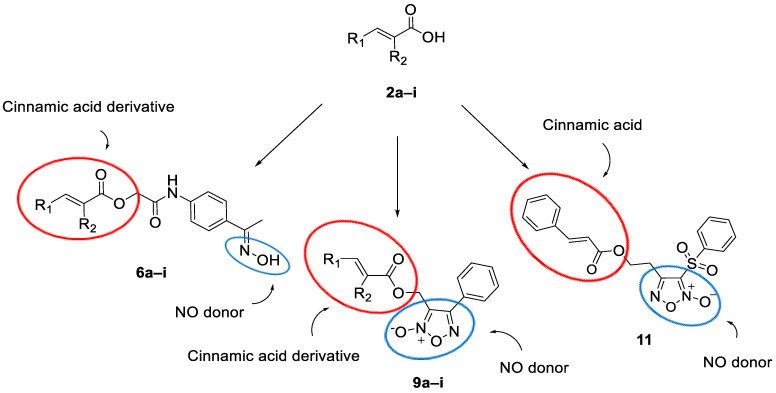
General structures of the new hybrids with cinnamic acid derivatives containing an oxime or a furoxan ring as NO donor.

Thus, we decided to design and synthesize a series of hybrids with cinnamic acid derivatives as advantageous multi-target agents, containing an oxime or a furoxan ring (Figure 1). We made a library of molecules, and we used drug-likeness and modeling studies on our library structures, as tools, in order to design LOX and COX-2 hybrid inhibitors acting as NO donors. The structural modifications are mainly focused on the cinnamic acid (simple) or with condensed rings, heterocyclic rings, and substitution on the double bond.

The drug-likeness of the hybrids was determined from the theoretical calculation of various molecular properties. The in silico results were performed, and the violations of Lipinski’s rule were considered using several platforms as well as the prediction of Absorbance, Distribution, Metabolism, Elimination, Toxicology (ADMET) properties. The gathered information supported the synthesis of the hybrid compounds in an attempt to define the importance and correlation of lipophilicity, steric, and electronic parameters on the biological activities.

Synthesis of the new hybrids was followed by verification of their structure with NMR, HRMS, and LCMS, while characterization of appearance, melting point, and R_f_ was performed.

Furthermore, the new synthesized hybrids were evaluated through in vitro studies considering multifunctionality. More specifically they were tested as COX-2/LOX inhibitors, as antioxidants, and also for their ability to release NO. Additionally, in vitro studies were performed in selected hybrids to evaluate their ability as COX-1 inhibitors and as anti-inflammatories by inhibition of denaturation of albumin. Finally, their cytotoxicity was screened in various cancer cell lines.

## 2. Results and Discussion

### 2.1. Chemistry

The design and synthesis of the new multitarget cinnamic hybrids were based on in silico analysis according to the ADMET results, modeling studies and 2D-QSAR studies supported by our previous publications [23].

**Scheme 1:**
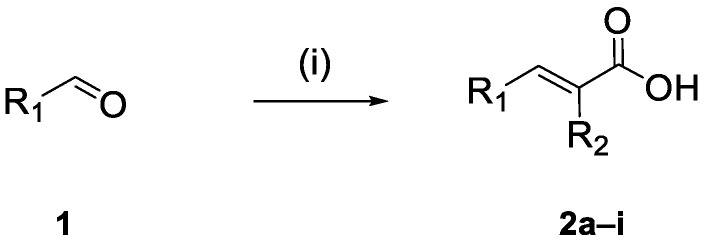
Synthesis of cinnamic acids **2a**–**i**. (i) malonic acid, piperidine, pyridine or phenylacetic acid, triethylamine (Et_3_N), and acetic anhydride (Ac_2_O).

To synthesize the new cinnamic acid–NO donor molecules, cinnamic acids **2a**–**i** were used. The acids were synthesized via a Knoevenagel–Doebner condensation of the suitable aldehyde with malonic acid in the presence of pyridine and piperidine [24,25], as shown in Scheme 1. The structures of the known compounds (Table 1) were verified according to literature spectral data or melting points [26,27,28,29,30,31,32,33].

#### 2.1.1. Synthesis of the Cinnamic Acid-Arylacetamide Oxime Hybrids **6a**–**i**

**Scheme 2:**
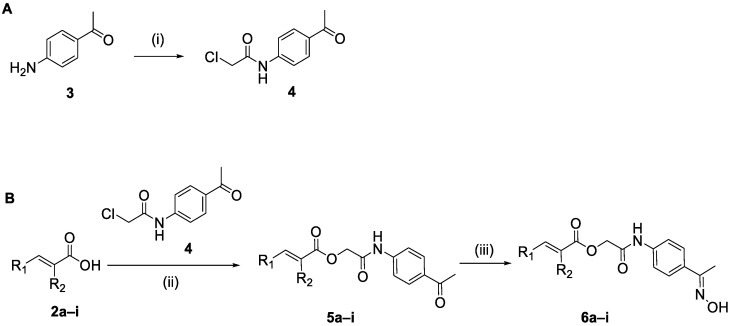
(**A**) Synthesis of *N*-(4-acetylphenyl)-2-chloroacetamide **4**. (i) ClCH_2_COCl, K_2_CO_3_, DCM, rt, 3 h. (**B**) General synthesis of oxime hybrids **6**. (ii) **2a**–**i**, Et_3_N, KI, DMF, microwave at 50 W, 50 °C, 5 min. (iii) NH_2_OH·HCl, CH_3_COONa, EtOH, microwave at 50 W, 50 °C, 5–15 min.

The synthesis of the hybrid oxime compounds **6a**–**i** involved the synthesis of the precursor *N*-(4-acetylphenyl)-2-chloroacetamide (**4**) [34]. Compound **4** was obtained easily from 4-aminoacetophenone (**3**) and chloroacetic chloride in the presence of anhydrous potassium carbonate, in dichloromethane DCM (yield: 72%). A coupling reaction between *N*-(4-acetylphenyl)-2-chloroacetamide (**4**) and cinnamic acid derivatives **2a**–**i** in the presence of triethylamine and potassium iodide in dimethylformamide DMF resulted in the hybrid cinnamic acid–arylacetamide ketones **5a**–**i**, in yields between 16 and 54%. Nucleophilic addition of hydroxylamine to hybrids **5** in ethanol EtOH and basic conditions, furnished the oxime hybrids **6a**–**i**, as presented in Scheme 2, in yields between 70 and 94%. All the reactions took place in microwaves at 50 W and 50 °C. The synthesis of compound **6a**, following a different synthetic route, has already been published [35], however, herein for a first time is presented its synthesis with the aim of microwave irradiation (Scheme 2).

The structural characterization of the new cinnamic acid analogues **5a**–**i** and **6a**–**i** was based on their spectral data and HRMS/LCMS analysis (Supplementary materials). All the compounds are novel with the exception of **5a**. Compound **5a** is the only known derivative mentioned in the literature for which its physicochemical and spectroscopic data were identical to the reported data [36].

Hybrids **5b**–**i** were analyzed based on their spectroscopic data to confirm their structures. More specifically, in the ^1^H NMR spectra recorded in DMSO-d_6_, a characteristic doublet appears around 6 ppm, corresponding to one of the two protons of the molecule’s double bond. The J values (15–17 Hz) are characteristic of a trans (E) configuration of the double bond for the cinnamic hybrids **5a**–**f**. Additionally, the NMR spectra of compounds **5g**–**i** shows the presence of a single isomer, which is likely the E form, as it is generally the more stable configuration. The chemical shift of the second proton of the double bond appears between 7 and 8 ppm also as a doublet, although it may be less distinguishable due to the presence of aromatic protons. Additionally, a distinct signal is observed near 9.5 ppm in all spectra, corresponding to the –NH proton. Subsequent analysis of the ^13^C NMR spectra in dimethyl sulfoxide DMSO-d_6_ showed the three carbonyl carbons of the molecules, appearing above 160 ppm, as expected, while the –CH_2_ and –CH_3_ group carbons appeared around 60 and 25 ppm, respectively. Finally, the LC–MS results underlined the presence of [M + Na]^+^ and [M − H]^−^.

Regarding hybrids **6a**–**i**, in the ^1^H NMR spectra recorded in DMSO-d_6_, a characteristic doublet appears approximately at 6 ppm, corresponding to one of the two protons of the molecule’s double bond. This signal shows a slight shift compared to the corresponding shift in the ketonic hybrids **5a**–**i**. Furthermore, the trans-stereochemistry is maintained **in 6a**–**f** cinnamic hybrids, as indicated by the J values of 15–17 Hz. Additionally, the NMR spectra of compounds **6g**–**i** shows the presence of a single isomer, which is likely the E form, as it is generally the more stable configuration. The second proton appears in the aromatic region (7–8 ppm) as a doublet, although it may not be clearly distinguishable due to the overlapping of aromatic protons. Additionally, the –NH proton signal is consistently observed near 9.5 ppm in all spectra, and additionally, in these molecules, the proton of the oxime group is also clearly visible as a distinct peak near 10 ppm. In the ^13^C NMR spectra (DMSO-d_6_), the two carbonyl carbons appear around 160 ppm, as expected. The third carbonyl carbon that was observed in the ketonic hybrids **5a**–**i**, now is shifted to approximately 150 ppm due to the introduction of the oxime group. The –CH_2_ carbon appears relatively unchanged compared to cinnamic hybrids **5a**–**i**, around 60 ppm, while the –CH_3_ carbon is now shifted to approximately 10 ppm, indicating the transformation of the ketone group to an oxime on the adjacent carbon. Finally, HRMS results showed the presence of [M + H]^+^ and [M − H]^−^, while LC–MS results revealed the presence of [M + Na]^+^ and [M − H]^−^ in most cases.

Detailed NMR spectra and the physicochemical properties of the novel derivatives are given in the experimental section (Supplementary materials).

#### 2.1.2. Synthesis of the Cinnamic Acid–Phenyl Furoxan Hybrids **9a**–**i**

**Scheme 3:**
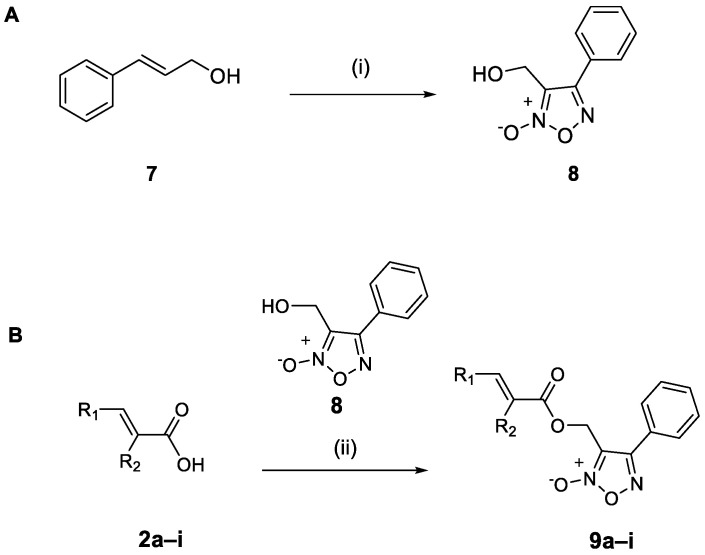
(**A**) Synthesis of 3-(hydroxymethyl)-4-phenyl-furoxan (**8**). (i) NaNO_2_, AcOH, rt, 24 h. (**B**) Synthsis of hybrids **9a**–**i**. (ii) EDCI·HCl, 4-DMAP, DMF, microwave at 50 W, 50 °C, 5–45 min.

The synthesis of the cinnamic acid–phenyl furoxan hybrids **9a**–**i** involved the synthesis of the precursor 3-(hydroxymethyl)-4-phenyl-furoxan (**8**) [37]. Compound **8** was obtained from cinnamoyl alcohol (**7**) in the presence of sodium nitrite in acetic acid (yield: 62%). A coupling reaction between furoxan **8** and cinnamic acid derivatives **2a**–**i** in the presence of 1-Ethyl-3-(3-dimethylaminopropyl)carbodiimide EDCI hydrochloride and 4-dimethylamino pyridine DMAP in dimethylformamide DMF provided the hybrid compounds **9a**–**i** in yields between 40 and 87%, as shown in Scheme 3.

The structural characterization of the new cinnamic acid–phenyl furoxan analogues **9a**–**i** was based on their spectral data and HRMS analysis. (Supplementary materials). More specifically, in the ^1^H NMR spectra in CDCl_3_, a characteristic doublet appears near 6.5 ppm, which is attributed to one of the two protons of the molecule’s double bond. The J values (15–17 Hz) are characteristic of a trans (E) configuration of the double bond of the cinnamic moiety for compounds **9a**–**f**. Additionally, the NMR spectra of cinnamic hybrids **9g**–**i** support the presence of a single isomer, which is likely the E form, as it is generally the more stable configuration. The second proton of the double bond appears in the aromatic region between 7 and 8 ppm as a doublet, which may not be clearly distinguishable in the spectrum due to the presence of aromatic hydrogens. Additionally, the signal of the –CH_2_ hydrogen is characteristic in these molecules, appearing around 5.5 ppm in all analogues’ spectra. Subsequently, the analysis of the ^13^C NMR spectra in CDCl_3_ showed that the most characteristic signal is the carbonyl carbon near 165 ppm, as expected, and the carbon of the –CH_2_ group appearing near 60 ppm. Finally, the HRMS results showed the presence of [M + H]^+^ and [M − H]^−^ ions, while the LC–MS results showed the presence of [M + Na]^+^ and [M − H]^−^ ions in most cases.

Detailed NMR spectra and the physicochemical properties of the novel derivatives are given in the experimental section (Supplementary materials).

#### 2.1.3. Synthesis of the Cinnamic Acid–Phenylsulfonyl Furoxan Hybrid **11**

**Scheme 4:**
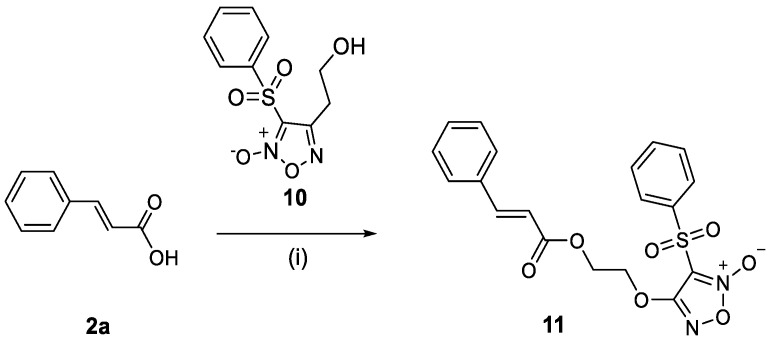
Synthesis of phenylsulfonyl furoxan hybrid molecule **11**. (i) EDCI·HCl, 4-DMAP, DMF, microwave at 50 W, 50 °C, 5 min.

A coupling reaction between phenylsulfonyl furoxan analogue **10** and cinnamic acid **2a** in the presence of EDCI hydrochloride and DMAP in DMF provided the cinnamic acid–phenylsulfonyl hybrid compound **11** with 35% yield (Scheme 4). Furoxan **10** was synthesized according to the literature [38].

Regarding hybrid **11**, its structural characterization was based on spectroscopic data, which were compared with literature data [39]. More specifically, in the ^1^H NMR spectra in CDCl_3_, a characteristic doublet appears near 6.5 ppm, which is attributed to one of the two protons of the molecule’s double bond. The J value (16 Hz) indicates the presence of a trans (E) configuration of the double bond. The second proton of the double bond appears in the aromatic region (7–8 ppm) as a doublet, which may not be clearly distinguishable in the spectrum due to the presence of aromatic hydrogens. Additionally, characteristic signals for the –CH_2_ protons of the molecule are observed around 4.5 ppm, appearing as two symmetrical multiplets. Subsequently, the analysis of the ^13^C NMR spectra in CDCl_3_ showed the most characteristic signals to be the carbonyl carbon appearing around 165 ppm, and the carbons of the –CH_2_ groups appearing around 60–70 ppm.

### 2.2. Physicochemical Studies

#### 2.2.1. In Silico Determination of Drug-likeness and ADMET Properties

Drug discovery is an expensive, time-consuming and challenging procedure. Due to these disadvantages, in the last decades, the determination of human pharmacokinetic properties has been studied by in silico procedures that have replaced the experimental procedures in an early stage. In silico studies include the determination of ADMET properties and drug-likeness of the molecule [40].

Drug-likeness stands for the possibility of a molecule becoming an oral drug, and it is a qualitative method that predicts the bioavailability of the molecule. One of the most widely used approaches for evaluating drug-likeness is the rule of five developed by Lipinski et al. [41]. According to Lipinski’s rule, poor absorption of a molecule is related to the presence of more than five H-bond donors and ten H-bond acceptors. Furthermore, values of the molecular weight (MW) > 500 and calculated logP value > five lead to poor absorption as well [42,43].

We calculated the physicochemical and molecular properties to analyze the hybrids **5a**–**i**, **6a**–**i**, **9a**–**i**, and **11** via the cheminformatics software tools of Molinspiration https://www.molinspiration.com/ (10 May 2025). According to Lipinski’s rule, all 28 synthesized compounds present druglikeness, with the exception of compounds **5h**, **6h**, **9b**, **9e**, **9g**, **9h**, and **9i**, for which one violation is presented (the milogP value) (Table 2).

In drug design, another important parameter is the blood–brain barrier penetration of a molecule that can lead to side effects [44]. The calculation of BBB penetration was measured using preADME software https://preadmet.webservice.bmdrc.org/ (10 April 2025), which calculates the in vivo blood–brain barrier (BBB) penetration, often expressed as the ratio C_brain_/C_blood_. The factors that influence BBB penetration value are lipophilicity (logP), TPSA and molecular weight [45]. When value C_brain_/C_blood_ is higher than 1, this indicates effective BBB penetration, with higher concentrations in the brain than in the blood. Such compounds are considered CNS-active. When value C_brain_/C_blood_ is between 0.1 to 1, that suggests partial penetration. The compound may have some CNS effects, depending on its pharmacodynamic properties. And finally, when value C_brain_/C_blood_ is lower than 0.1, that indicates poor BBB penetration. These compounds likely have limited CNS effects unless they are transported actively [46].

Regarding the tested analogues, it seems that almost half of them exhibit moderate absorption through the BBB (scores 0.1–1), while the other half shown low BBB penetration (scores < 0.1). Only compound **9g** presents high BBB penetration (score > 1). All the above are depicted in Table 2.

**Table 2:** Molecular properties prediction—Lipinski’s Rule of Five. The prediction is calculated by preADME [46] and Molinsiration [47] platforms.

Compound	Milog P ^a^	TPSA ^b^	N^o^ Atoms	N^o^ O, N ^c^	N^o^ OH, NH ^d^	N^o^ Violations	N^o^ Rotational Bonds ^e^	Volume ^f^	MW ^g^	logBB ^h^
**5a**	3.23	72.47	24	5	1	0	7	294.57	323.35	0.0909
**5b**	4.21	72.47	28	5	1	0	7	338.56	373.41	0.0295
**5c**	2.95	72.47	23	5	1	0	7	285.28	329.38	0.3054
**5d**	2.31	85.61	23	6	1	0	7	276.14	313.31	0.2410
**5e**	4.25	72.47	27	5	1	0	7	329.27	379.44	0.0369
**5f**	3.61	85.61	27	6	1	0	7	320.13	363.37	0.0282
**5g**	4.56	72.47	30	5	1	0	8	365.98	399.45	0.2139
**5h**	5.54	72.47	34	5	1	1	8	409.97	449.51	0.0762
**5i**	4.28	72.47	29	5	1	0	8	356.69	405.48	0.7968
**6a**	3.29	88.00	25	6	2	0	7	306.86	338.36	0.0797
**6b**	4.27	88.00	29	6	2	0	7	350.86	388.42	0.0980
**6c**	3.01	88.00	24	6	2	0	7	297.58	344.39	0.0296
**6d**	2.37	101.14	24	7	2	0	7	288.43	328.32	0.0286
**6e**	4.32	88.00	28	6	2	0	7	341.57	394.45	0.0231
**6f**	3.68	101.14	28	7	2	0	7	332.42	378.38	0.0287
**6g**	4.63	88.00	31	6	2	0	8	378.27	414.46	0.2401
**6h**	5.61	88.00	35	6	2	1	8	422.26	464.52	0.3068
**6i**	4.35	88.00	30	6	2	0	8	368.98	420.49	0.0733
**9a**	4.45	77.80	24	6	0	0	6	280.81	322.32	0.4173
**9b**	5.43	77.80	28	6	0	1	6	324.80	372.38	0.0433
**9c**	4.17	77.80	23	6	0	0	6	271.52	328.35	0.3747
**9d**	3.53	90.94	23	7	0	0	6	262.38	312.28	0.3675
**9e**	5.48	77.80	27	6	0	1	6	315.51	378.41	0.1693
**9f**	4.83	90.94	27	7	0	0	6	306.37	362.34	0.1375
**9g**	5.79	77.80	30	6	0	1	7	352.22	398.42	1.3975
**9h**	6.77	77.80	34	6	0	1	7	396.21	448.48	0.2642
**9i**	5.50	77.80	29	6	0	1	7	342.93	404.45	0.9812
**11**	3.78	121.17	29	9	0	0	9	338.03	416.41	0.1441

^a^ Logarithm of partition coefficient between n-octanol and water (milog P); ^b^ topological polar surface area (TPSA) values expressed as Å^2^; ^c^ number of hydrogen bond acceptors (n-ON); ^d^ number of hydrogen bond donors (n-OHNH); ^e^ number of rotatable bonds (n-rotb); ^f^ molecular volume; ^g^ molecular weight; and ^h^ blood–brain barrier.

Another important parameter is the inhibition of Cytochrome P450 (CYP) enzymes. These are a group of enzymes that play a crucial role in the metabolism of drugs in the liver and other tissues. They are involved in the phase I metabolism of many drugs, which typically involves oxidation reactions, and can affect the pharmacokinetics, efficacy, and toxicity of a drug. Thus, the in silico prediction of metabolism is essential for assessing the risk of drug–drug interactions early, in the drug discovery process [48,49,50].

The prediction of CYP metabolism of the new hybrids using CypRules [51] is shown in Table 3. All compounds **5a**–**i** and **6a**–**i** do not inhibit the CYP2C19 enzyme, while all the furoxans **9a**–**i** and **11** showed inhibition of this enzyme. In addition, compounds **9a**–**i** and **11** showed inhibition of CYP2C9 enzyme while compounds **5a**–**i** and **6a**–**i** did not show any inhibition activity except the most lipophilic ones, **5g**, **5h**, **6g**, and **6h**. All compounds showed no inhibition activity for CYP2D6, while all of them are predicted to inhibit the CYP3A4 enzyme.

Furthermore, toxicity is an important property to be considered in relation to discovery of new drugs. The potential toxicity of the tested compounds is predicted through the preADMET platform [46] in various tests. These tests are hERG inhibition, Carcinogenicity in Mouse, Carcinogenicity in Rat, Ames test and specifically in bacterial strains TA100_NA, TA100_10RLI, TA1535_NA and TA1535_10RLI.

##### hERG Inhibition

The hERG potassium channel (human Ether-à-go-go-Related Gene) plays a critical role in maintaining the proper electrical activity of the heart, specifically contributing to the repolarization phase of the cardiac action potential, the process by which the heart returns to its resting state following each contraction. Inhibition of this channel by a compound can result in QT interval prolongation on the electrocardiogram, potentially leading to life-threatening arrhythmia or even sudden cardiac death. For this reason, hERG inhibition is a key step in the assessment of the cardiotoxic potential of novel drug candidates’ prediction [50].

The hERG inhibitory potential of the newly synthesized compounds was predicted, and the results are summarized in Table 3. The table categorizes the compounds based on their predicted hERG inhibition risk correlated with the cause of cardiotoxicity [49].

Compounds **5g**, **5h**, **9a**, **9b**, **9e**–**9i**, and **11** were predicted to be of low risk, suggesting minimal interaction with the hERG channel. In contrast, most of the **5**-series compounds (except for **5g** and **5h**), as well as **9c**, **9d**, and selected compounds from the **6**-series, were classified of moderate risk. Two compounds, **6b** and **6i**, are identified to induce severe cardiac arrhythmias.

##### Carcinogenicity in Mouse and Rat

Carcinogenicity refers to the potential of a chemical compound to induce neoplastic transformations in biological systems. Traditionally, the assessment of carcinogenicity involves long-term in vivo studies, typically lasting two years, in which mice or rats are chronically exposed to the test compound. The preADMET platform [46] enables in silico prediction of carcinogenic potential, based on models trained using data from the U.S. National Toxicology Program (NTP) and the Food and Drug Administration (FDA), derived from standardized long-term rodent bioassays [46].

The in silico predictions, summarized in Table 3, indicate that the majority of the tested compounds were predicted to be carcinogenic in mice, suggesting a high probability of tumorigenicity in these species. The only exception was compound **6a**, which exhibited a negative carcinogenicity prediction.

Conversely, the predictions for rat models showed lower sensitivity. While ketone derivatives **5a**–**i** and oxime derivatives **6a**–**i** were consistently predicted to be carcinogenic, several furoxan-based compounds **9a**–**i** demonstrated favorable results, suggesting a lower potential for carcinogenic liability.

These results highlight species-specific differences in predicted carcinogenic responses, with rats displaying lower sensitivity, particularly toward furoxan scaffold. These findings emphasize the necessity of performing cross-species evaluations when assessing the long-term toxicological risk of novel chemical entities, to ensure a more comprehensive and accurate risk assessment.

##### Ames Test

The Ames test is a biological assay used to assess the mutagenic potential of chemical compounds. The test uses specific strains of the bacterium *Salmonella typhimurium* that carry a mutation rendering them unable to synthesize the amino acid histidine. These bacteria are exposed to the test compound, and the ability of the compound to cause mutations—which reverts the mutation in the bacteria (allowing them to grow without histidine)—is predicted. A negative result in the Ames test is desirable, as it suggests that the substance does not cause mutations and is likely safe. A positive result indicates the substance is mutagenic, meaning it may cause genetic mutations and could potentially be harmful [52,53].

In this study, the mutagenic potential of the novel synthetic compounds **5a**–**i**, **6a**–**i**, and **11** was evaluated in silico. The preADMET platform predicts Ames test outcomes based on robust predictive models. As shown in Table 3, 26 out of the 27 compounds were predicted to be mutagenic, with only compound **5g** yielding a non-mutagenic prediction.

The predominance of mutagenic predictions indicates a considerable genotoxic concern that warrants cautious interpretation, particularly in the context of drug development and experimental safety. Compound **5g** appears to be the only candidate with a potentially safer mutagenic profile, although additional experimental validation is essential for a comprehensive risk assessment.

The in silico Ames test results from the preADMET platform [46] indicate a high mutagenic potential for most of the evaluated compounds, particularly after metabolic activation with liver enzymes. Ketone compounds **5a**–**i** and oximes derivatives **6a**–**i** were generally negative in the absence of metabolic activation (TA100_NA and TA1535_NA), suggesting low direct mutagenicity. However, they became mostly positive when metabolic activation was introduced (TA100_10RLI and TA1535_10RLI), indicating possible pro-mutagenic behavior. Furoxan derivatives **9a**–**i** and **11** showed mixed results without activation but were positive after metabolism. Results are shown in Table 4.

Among all tested compounds, only compound **5g** remained consistently negative across all models, making it a potential candidate for further investigation. Nonetheless, additional in vitro and in vivo studies are essential to confirm these findings and to fully assess the safety profile of these compounds.

Furthermore, in drug design, descriptors such as TPSA are widely used to estimate ADME properties, since polar surface area strongly influences intestinal absorption and oral bioavailability. Therefore, the percentage of absorption (%ABS) was calculated with the equation below [54]:

%ABS = 109 − 0.345 TPSA (1)

TPSA was calculated via Molinspiration platform [47] (Table 1). Results of the %ABS of the new molecules **5a**–**i**, **6a**–**i**, **9a**–**i**, and **11** are shown in Table 5. The bioavailability of bioactive molecules is closely linked to their topological polar surface area (TPSA), a descriptor that correlates strongly with hydrogen bonding capacity [55]. The TPSA values of the hybrids ranged from 67.20 to 84 Å^2^, which are all below the commonly accepted threshold of 160 Å^2^, underlying a good oral bioavailability.

With the exception of hybrid **11**, all the other hybrids presented a very high absorption, as indicated by their %ABS values derived from TPSA. Specifically, hybrids **5a**–**5i** and **9a**–**9i** exhibited %ABS values in the range of 77.6–84.0%, while hybrids **6a**–**6i** ranged around 78.6%, all suggesting excellent intestinal absorption. Hybrid **11**, with a TPSA of 121.17 Å^2^ and a %ABS of 66.2%, was the only compound with notably lower absorption supporting the suitability for oral administration.

Importantly, only the hybrids within the TPSA range of 72.47–90.94 Å^2^ are expected to be capable of crossing the blood–brain barrier.

### 2.3. Biological Evaluation

In the present study, the synthesized hybrids **5a**–**i**, **6a**–**i**, **9a**–**i**, and **11** were evaluated for their in vitro activity as inhibitors of lipid peroxidation and soybean lipoxygenase. The hybrid oximes **6a**–**i** and furoxans **9a**–**i** and **11** that contain a NO donor moiety, were also evaluated for their ability to release NO, to inhibit COX-2 and albumin denaturation. Selected hydrids were tested for COX-1 inhibition. Finally, the selected hybrids **6c**, **6i**, **9e**, **9g**, and **9i** were tested for their cytotoxic activity with MTT viability assay in various cancer cell lines.

#### 2.3.1. In Vitro Lipid Peroxidation

Cell metabolism is a continuous source of reactive oxygen species (ROS), some of which are highly toxic. The body relies on enzymatic and non-enzymatic mechanisms to detoxify these compounds rapidly. However, their extreme reactivity and ability to initiate chain reactions can lead to pathological conditions. Studies have reported a marked increase in lipid peroxidation in the brains of Alzheimer’s disease patients, linking oxidative stress to the disease’s progression. Similarly, elevated levels of ROS and lipid peroxidation products have been observed in Parkinson’s disease, amyotrophic lateral sclerosis, multiple sclerosis, and Huntington’s disease, suggesting a common role of oxidative damage in neurodegeneration. In addition, oxidative stress is implicated in cardiovascular diseases, diabetes, cancer, and chronic inflammatory conditions. Consequently, antioxidants that prevent lipid peroxidation in the brain and other tissues could hold significant therapeutic potential [56,57,58].

In the present study, the aim was to evaluate the anti-lipid peroxidation activities of the synthesized compounds **5a**–**i**, **6a**–**i**, **9a**–**i**, and **11** and compare their efficacy to that of Trolox, a well-known antioxidant. Using the water-soluble compound 2,2′-azobis(2-amidinopropane) hydrochloride (AAPH), peroxyl radicals are generated through spontaneous thermal decomposition in vitro. This experimental setup imitated the lipid peroxidation processes occurring in cells due to radical activity allowing the investigation of the effectiveness of the tested compounds under conditions resembling cellular oxidative stress [56].

In general, within the compounds, the most potent anti-lipid peroxidation activity was shown by the derivatives **5g**, **6b**, **6g**, **6h**, **9a**, **9f**, **9g**, **9h**, and **11** with 89–97.7% inhibition activity that was comparable to the reference compound Trolox (93%) at 100 μM concentration. All the above compounds as well as compounds **9b** and **9d** have a better or similar activity to cinnamic acid (78%). All the compounds, except **5g**, contain either an oxime or a furoxan group, indicating the presence of a moiety that can potentially act as a ROS scavenger. Furthermore, **5g**, **6g**, and **9g** exhibited significant lipid peroxidation activity, highlighting the importance of this cinnamic derivative. Also, it seemed that higher lipophilicity leads to higher anti-lipid peroxidation activity, as the above compounds have lipophilicity values between 4.27 and 6.77. The anti-lipid peroxidation activity of all compounds **5a**–**i**, **6a**–**i**, **9a**–**i**, and **11** is presented in Table 6.

Perusal of the results among the hybrids of each group shows that for group **5** with R_1_ = H, the anti-lipid peroxidation activity seems to be almost equal for **5a** ≥ **5c**, whereas in **5b** the naphthyl condensed derivative does not present any result under the experimental condition. It seems that among the heterocyclic compounds thienyl **5c** presents antioxidant behavior whereas the furyl **5d** not any. This is changed in **5f**, the condensed benzofuryl in relation to **5d**. We observed also that replacement of the phenyl ring by a naphthyl led to an inactive compound **5b** as well as the presence of a benzothienyl ring in place of thienyl. The results are differentiated with the presence of R_2_ = phenyl as follows, **5h** > **5b**, **5i** > **5c**, **5g** > **5a**. It seems that for this group the presence of a **R_2_** = **phenyl substituent** leads to enhancement.

For the group **6**, the naphthyl-substituted derivative (compound **6b**), is the most potent (**6b** > **6d** > **6a**) followed by the furyl and thienyl derivative. Subsequently, **6c** as well as **6e** and **6f**—the two heteroaromatic condensed derivatives—are inactive. Among the R_2_ = phenyl substituted derivatives, only **6g** and **6h** are highly active.

The transformation of ketones **5** to oximes **6** is accompanied by an increase in some cases, e.g., **5a**/**6a**, **5b**/**6b**, **5d**/**6d**, and **5h**/**6h**.

The hybrids of group **9** present higher anti-lipid peroxidation activity (67–97%) with the exception of compound **9i**. The presence of the phenyl furoxanyl group as well as of the phenylsulfonyl furoxanyl ring in **11** seems to play a crucial role.

#### 2.3.2. In Vitro LOX Inhibition

Inhibition of lipoxygenases (LOXs) plays a crucial role in managing inflammation, as LOXs are enzymes involved in the metabolism of arachidonic acid, leading to the production of bioactive lipid mediators called leukotrienes. By inhibiting LOXs, it is possible to reduce the production of leukotrienes and other inflammatory mediators, thus reducing inflammation. This has therapeutic implications for various inflammatory conditions, such as asthma, rheumatoid arthritis, and inflammatory bowel diseases [3,59].

In vitro anti-LOX activities were evaluated using soybean lipoxygenase isoenzyme-1 (SLOX-1), a commonly used model for mammalian LOXs [60]. SLOX-1 shows its activity at pH 9.0 and primarily catalyzes the formation of the 13-hydroperoxide derivative from the substrate (linoleic acid). The production of this derivative was monitored by its absorbance at 234 nm [61].

Compared to the reference compound NDGA, all tested compounds displayed lower potency (Table 6). The best inhibition activity was shown by compound **9a** the furoxanyl representative with IC_50_ = 3.5 μΜ. Comparison of the two IC_50_ values of cinnamic acid (56 μM) and compound **9a**, showed that the addition of the furoxanyl moiety plays a key role in the enhancement of the LOX inhibition. High potency is also shown by thiophene-oxime analogue **6c** and by the double-bond-substituted furoxan compound **9h** with IC_50_ = 10 μΜ. Compound **9h** is the most lipophilic of all the tested compounds with milogP 6.77. Furthermore, the compounds **5h**, **6a**, **6g**, and **9g** showed better inhibition of LOX than cinnamic acid. These molecules, except **6a**, contain a phenyl-substituted double bond in their structures and are highly lipophilic.

The study revealed distinct differences in lipoxygenase inhibition among the tested compounds. In group **5**, only three compounds **5b**, **5f**, and **5h** showed activity, with the lipophilic derivative **5h** being the most potent (IC_50_ = 33 μM). Among them, two derivatives have the R_1_ = naphthyl group and one has a benzofuryl group. Both substituents offer to the overall lipophilicity and to the volume of the molecules. The presence of R_2_ = phenyl leads to higher inhibition (**5b/5h**). Within group **6**, seven active compounds are yielded, suggesting that the oxime moiety may enhance enzyme interaction due to the oximes behavior to act as ligands with metals. Compound **6c**, which contains a thiophene group on its structure, shows significant activity (IC_50_ = 10 μM). Among the furoxan derivatives, the simplest of all, **9a**, demonstrated the best overall inhibition (IC_50_ = 3.5 μM), followed by **9h** with IC_50_ = 10 μΜ, and R_1_ = naphthyl and R_2_ = phenyl groups. Significant results were given by **9b**, **9c**, and **9i** with R_1_ substituents naphthyl and thienyl groups on the furoxan ring as well as compound **11** in which the sulfonyl furoxanyl substitution led to complete loss of activity. A phenyl group in R_2_ resulted to the potent inhibitor **9h** compared to **9b** R_2_ = H. The presence of the benzothienyl group led to an inhibitory activity **9e** compared to **9c**. No significant change was noticed when the furyl group was replaced by a benzofuryl group. Lipophilicity does not influence this biological activity.

#### 2.3.3. In Vitro COX-2 and COX-1 Inhibition

Cyclooxygenase (COX) isoforms catalyze two key reactions. First, they convert arachidonic acid into prostaglandin G_2_ (PGG_2_). Then, through their peroxidase activity, PGG_2_ is reduced to prostaglandin H_2_ (PGH_2_). COX-1 isoform is constitutively expressed in most tissues, and it is involved in maintaining normal physiological functions like protecting the gastric lining, regulating renal blood flow, and platelet aggregation. COX-2 isoform is an inducible enzyme expressed during inflammation, pain, and fever; primarily involved in the production of pro-inflammatory prostaglandins [4,62].

Non-selective NSAIDs (e.g., ibuprofen, aspirin) inhibit both COX-1 and COX-2, which can lead to side effects such as gastrointestinal irritation or ulcers due to COX-1 inhibition. Selective COX-2 inhibitors (e.g., celecoxib) target COX-2, reducing inflammation and pain with fewer gastrointestinal side effects [4,62].

Enzyme inactivation can occur due to the accumulation of hydroperoxides formed from arachidonic acid or other fatty acid substrates. In order to prevent this in the in vitro experiment used for testing COX inhibition activity of the new molecules, a co-substrate like *N*,*N*,*N*′,*N*′-tetramethyl-p-phenylenediamine (TMPD) is included, as it undergoes co-oxidation by PGG_2_. The oxidized TMPD has a maximum absorbance at 590 nm. COX enzymes also depend on heme (Fe^3+^-protoporphyrin IX) as a cofactor, which can dissociate during purification. To ensure maximum enzyme activity, heme is added to the reaction mixture during the in vitro assay for ovine COX-2 inhibition and COX-1 inhibition [63].

**Figure 2:**
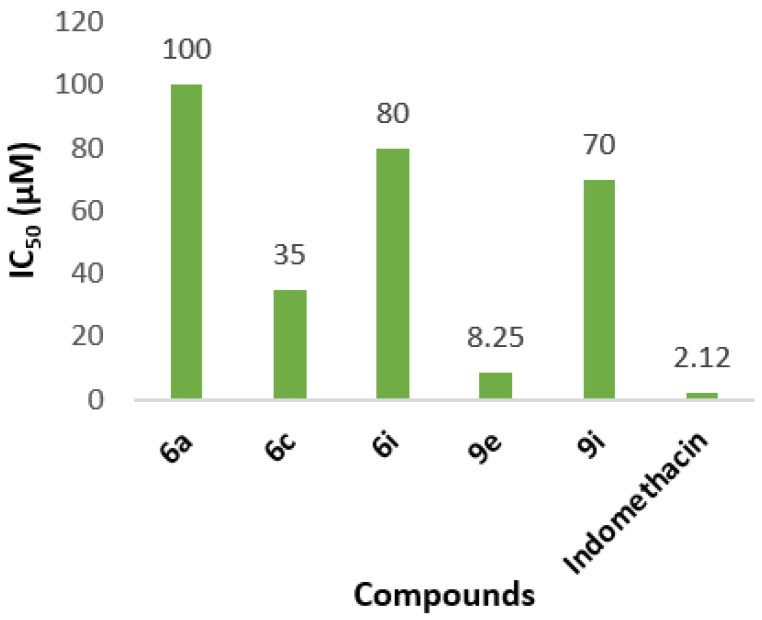
IC_50_ values of the selected tested compounds with COX-2 inhibition activity.

None of the tested compounds **6a**–**i**, **9a**–**i**, and **11** have shown better activity than indomethacin, used as standard in COX-2 inhibition assay (Table 6). The best inhibitor is the compound **9e** with IC_50_ = 8.25 μM with milogP value 5.48. Compound **6c** has the second better IC_50_ value at 35 μM. Both compounds contain a sulfur atom at their structure in the cinnamic acid ring. Only compounds **6a**, **6i**, and **9i** have shown inhibition in terms of IC_50_ values (IC_50_ = 100, 80, 70 μM, respectively) as COX-2 inhibitors (Figure 2).

**Figure 3:**
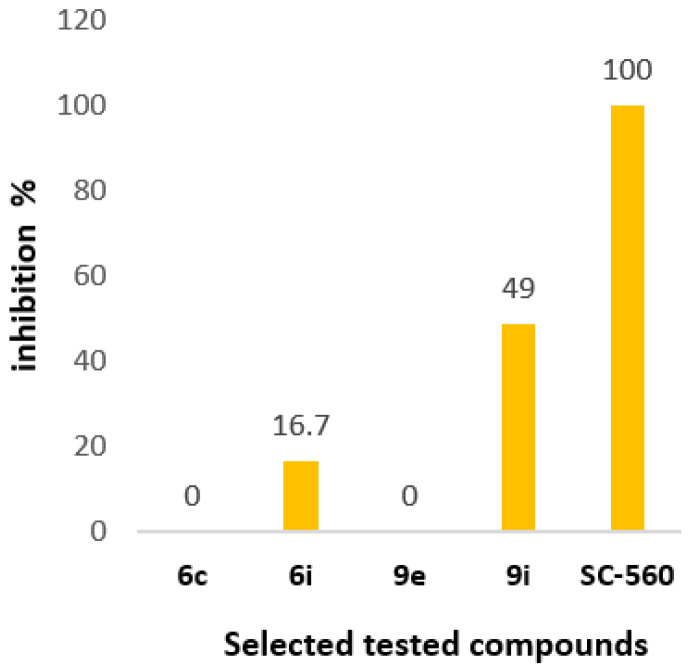
% inhibition of the selected tested compounds as COX-1 inhibitors at 100 μM.

Furthermore, to better clarify if the above compounds **6c**, **6i**, **9e**, and **9i** are selective COX-2 inhibitors, we tested them as COX-1 inhibitors (Table 6). Compounds **6c** and **9e** did not show any inhibition activity of COX-1 isoform and they are selective COX-2 inhibitors. The best COX-1 % inhibition activity has been presented by the compound **9i**, while compound **6i** showed lower inhibition activity, underlying the importance of the furoxan moiety for COX-1 inhibition activity. None of the compounds showed better activity compared to the reference standard SC-560 (100%) at 100 μΜ (Figure 3). Lipophilicity does not show any role.

#### 2.3.4. NO Release Ability

**Scheme 5:**
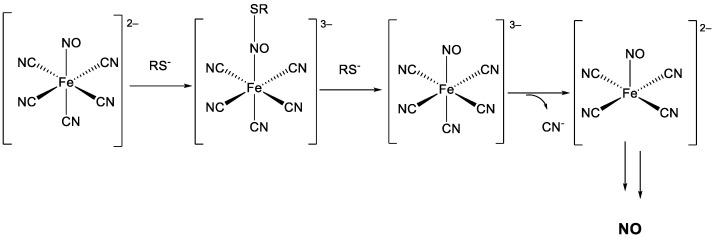
Mechanism of NO release from SNP with thiol compound.

This assay evaluates the ability of nitrate esters to release nitric oxide (NO). First, sodium nitroprusside (SNP) reacts with the thiol compound, cysteine, which serves as a reducing agent (Scheme 5). The thiol donates electrons, facilitating the breakdown of the nitroprusside complex. Afterwards, the reaction leads to the formation of intermediate complexes, such as nitrosothiol species and the decomposition of these intermediates results in the release of nitric oxide (NO). According to the literature [64], measurements are conducted both in the presence and absence of the thiol agent, cysteine. No significant difference was observed between the two experimental conditions, and the results were reproducible. Sodium nitroprusside (SNP) was used as the reference compound [65].

**Figure 4:**
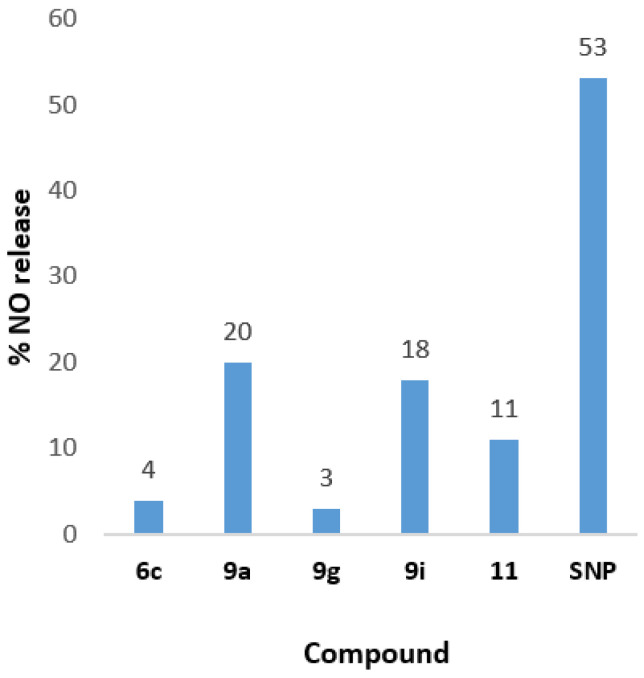
In vitro NO-releasing ability of the compounds with the best activity.

According to the experimental results (Table 6), the compounds **6a**–**i**, which contain oxime moiety as an NO-donating group, did not exhibit significant nitric oxide (NO) releasing ability overall. Specifically, compounds **6b**, **6c**, **6d**, **6e**, and **6f** displayed a slight ability to release NO, with low percentages ranging from 0.2% to 4%, while the remaining compounds **6a**, **6g**, **6h**, and **6i** showed no activity. These compounds were compared to the reference compound SNP, which demonstrates an NO release of 53% at 100 μΜ. Regarding compounds **9a**–**i** and compound **11**, which feature a furoxan moiety as an NO donor, the results showed that compounds **9a** and **11** exhibited good NO-releasing ability, with percentages of 20% and 11%, respectively. Both compounds have a cinnamic acid moiety in their structure. Additionally, it appears that the substitution pattern on the furoxan ring influences its NO-donating ability, with the benzofuroxan structure proving to be the most effective. These compounds showed the highest activity among all the new compounds **6a**–**i**, **9a**–**i**, and **11**, although their NO release was still lower than that of the reference SNP (53%) (Figure 4). However, the furoxan group proved to be more promising and effective than the oxime group, as indicated by the results.

#### 2.3.5. In Vitro Inhibition of Albumin Denaturation

The ability of a compound to inhibit the thermal denaturation of albumin is considered an indicator of anti-inflammatory activity [66]. Heat exposure simulates conditions of cellular stress or inflammation, during which protein and tissue damage occur. Certain substances can protect proteins from denaturation upon heating by stabilizing their structure and preventing aggregation and precipitation.

The experimental results showed that almost all compounds **6a**–**i** and **9a**–**i**, as well as compound **11**, did not exhibit inhibitory activity. The only exception was shown by compounds **6d** and **6e**, which demonstrated inhibition rates of 10% and 63.6%, respectively. These were the only compounds that showed activity in this assay. Notably, the thiol-containing compound **6e** exhibited stronger inhibitory capacity than the reference compound acetylsalicylic acid (31%). The results of the assay are shown in Table 6.

#### 2.3.6. MTT Assays on Cancer Cell Lines

The cytotoxic activity of five compounds was initially evaluated in HeLa (cervical cancer) and MCF-7 (breast cancer) cell lines using the MTT viability assay. As shown in Table 7, **6i** exhibited the highest potency among all compounds tested, with EC_50_ values of 36.69 μM (HeLa) and 38.34 μM (MCF-7), following treatment for 48 h [67].

Following these data, compound **6i** was further tested in four additional cell lines: U251 and U87, which are derived from glioblastoma; MDA-MB-435, which originates from melanoma; and MDA-MB-231, which is derived from triple-negative breast cancer.

As shown in Table 8, this compound demonstrated the strongest cytotoxic effect in HeLa cell line (EC_50_ = 36.69 μM), while the lowest activity was observed in MDA-MB-435 (EC_50_ = 52.89 μM). In the remaining cell lines (U251, U87, and MDA-MB-231), EC_50_ values ranged from 40.94 to 45.44 μM, indicating moderate sensitivity.

### 2.4. Computational Studies—Docking Simulation

#### 2.4.1. Docking Studies on Soybean Lipoxygenase

The interactions at the molecular level between the ligands and soybean lipoxygenase were explored through molecular docking. Soybean lipoxygenase-1 crystal structure (PDB ID: 3PZW) was selected to rationalize the obtained biological results. The crystal structure of soybean lipoxygenase-1 (PDB ID: 3PZW) is lacking a co-crystallized ligand, requiring the identification of possible allosteric binding sites beyond the known iron-binding and substrate-binding regions, as reported in recent studies. Detsi, A. et al. [68] have identified three potential binding sites using SiteMap’s [69]. Researchers have found that Site 1 is located between the amino-terminal β-barrel (PLAT domain) and the α-helical domain, which houses the catalytic iron [70,71,72]. Sites 2 and 3 are also positioned within the α-helical domain. Additionally, Site 1 is recognized as a potential binding site in blind docking studies [70,71,72].

**Figure 5:**
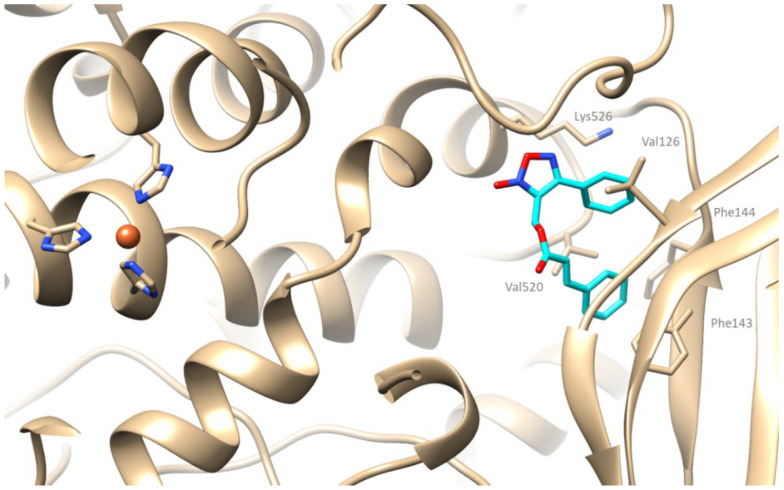
Putative binding mode of compound **9a** (depicted in cyan) bound to SLOX-1. Iron appears as an orange sphere.

Based on the above research studies, blind docking was performed in addition to that of the active center encompassing all potential binding sites. The selected for synthesis candidates have been concluded that bind to Site 1 as previously reported [70,71,72]. Compound **9a** being the most active in vitro within the series with an IC_50_ of 3.5 μΜ, develops hydrophobic interactions with the amino acids Val126, Phe143, Phe144, Val520, and π-cation interaction with Lys526 (Figure 5).

#### 2.4.2. Docking Studies on COX-2

**Figure 6:**
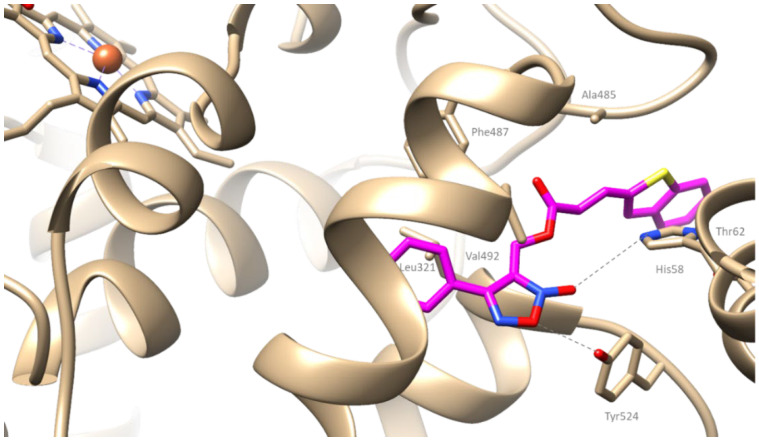
Putative binding mode of compound **9e** (depicted in magenta) bound to COX-2.

Compound **9e**, being the most potent in vitro among the series, presented a binding score of −9 kcal/mol. It seems to enter in the active cavity interacting hydrophobically with residues Thr62, Leu321, Ala485, Phe487, and Val492. Additionally, it develops two hydrogen bonds with His58 and Tyr324 and a salt bridge with His58 (Figure 6).

## 3. Materials and Methods

### 3.1. General Information

Reagents were purchased from Acros^®^ (Osaka, Japan), Alfa-Aesar^®^ (Karlsruhe, Germany), Fluka^®^ (Buchs, Switzerland), Fluorochem^®^ (Hadfield, UK), Merck^®^ (Darmstadt, Germany), Sigma-Aldrich^®^ (Saint Louis, MO, USA), and TCI^®^ (Tokyo, Japan). Solvents used were of analytical grade (Merck, Sigma-Aldrich, SDS (Hong Kong, China)). When required, drying of solvents was performed according to the standard literature procedures. Solvents used in column chromatography were distilled prior to use. Reactions involving moisture-sensitive reagents were carried out in oven-dried glassware under inert conditions. Anhydrous solvents and reagents were transferred using syringes. Thin-layer chromatography (TLC) was performed using pre-coated silica gel 60 F254 plates (code 5, Merck) to monitor reaction progress and product purification. Visualization of TLC plates was performed under UV light at 254 nm. Flash column chromatography was employed for the isolation or purification of products. Columns were packed with amorphous silica gel (SiO_2_, 70–230 mesh, Merck), and mobile phases were prepared as described in the corresponding procedures. Analytical samples were dried using appropriate low-pressure apparatus. Column chromatography was carried out using Silica gel 60 (Merck KGaA, Darmstadt, Germany).

Melting points were determined in capillary tubes using a MEL-Temp II apparatus (Lab. Devices, Holliston, MA, USA) and are uncorrected. Nuclear Magnetic Resonance (NMR) spectra were recorded on an Agilent (Santa Clara, CA, USA) 500/54 DD2 spectrometer (500 MHz for ^1^H and 125 MHz for ^13^C) at the Department of Chemistry, Aristotle University of Thessaloniki. Samples were dissolved in deuterated dimethyl sulfoxide (DMSO-d_6_) or deuterated chloroform (CDCl_3_). Chemical shifts (δ) are reported in parts per million (ppm) relative to the residual solvent peak as an internal standard. The following abbreviations are used for proton multiplicities: s (singlet), d (doublet), t (triplet), q (quartet), m (multiplet), br (broad), and combinations thereof. Coupling constants (J) are given in Hertz (Hz). High-resolution mass spectra (HRMS, ESI-MS) were obtained using a ThermoFisher Scientific LTQ Orbitrap Discovery MS (168 Third Avenue, Waltham, MA, USA). Theoretical fragments were calculated using the online tool available at https://www.sisweb.com/mstools/isotope.htm, 28 August 2025. Additional mass spectra were recorded using an LC-MS 2010 EV spectrometer (Shimadzu, Tokyo, Japan). Fragmentation patterns were predicted using PerkinElmer ChemDraw Professional 17.1 (PerkinElmer Informatics Inc., Waltham, MA, USA). All microwave irradiation experiments were carried out in CEM-Discover monomode microwave device. UV-Vis spectra were recorded on a Shimadzu PharmaSpec UV-1700 spectrophotometer (Kyoto, Japan) using 1 cm pathlength cuvette.

Biological assays were conducted using reagents including soybean lipoxygenase, sodium linoleate, 2,2′-azobis(2-amidinopropane) dihydrochloride (AAPH), human cyclooxygenase-2, albumin, Trolox, nordihydroguaiaretic acid (NDGA), *N*,*N*,*N*′,*N*′-tetramethyl-p-phenylenediamine (TMPD), heme, cyclooxygenase-1, phosphate buffer (pH 7.4), L-cysteine, Griess reagent, and sodium nitroprusside (SNP), all purchased from Merck and Sigma-Aldrich. Cancer Cell Lines HeLa, MCF-7, U251, U87, MDA-MB-435, and MDA-MB-231 used in the biological experiments were obtained from the American Type Culture Collection (ATCC) and cultured in Dulbecco’s Modified Eagle’s Medium (DMEM).

The following software tools were used and are discussed in detail in the corresponding sections:

- AutoDockVina v.1.1.1 (The Scripps Research Institute), via the PyRx program [73]
- UCSF Chimera v.1.5.3 (University of California) [74]
- GROMACS toolkit v.4.6.5 [75]
- Modeller [76]
- OpenBabel v.2.2.3 [77]
- AmberTools [78]
- Molinspiration (https://www.molinspiration.com, accessed on 10 May 2025) [47]
- preADMET (https://preadmet.webservice.bmdrc.org/, accessed on 10 April 2025) [46]

### 3.2. Computational Studies

#### 3.2.1. Molecular Docking Studies on Soybean Lipoxygenase

The protein structure (PDB ID: 3PZW) was visualized and pre-processed using UCSF Chimera (version 1.17) [79]. Water molecules and non-essential crystallographic components were removed via Chimera. Missing residues (Met1–Phe2–Ser3–Ala4–Gly5; Glu21–Val22–Asn23–Pro24–Asp25–Gly26–Ser27–Ala28–Val28–Asp29; Ile117–Ser118–Asn119–Gln120) were added using Modeller (v. 10.3) [80]. Hydrogen atoms and partial charges were incorporated using AmberTools 23 [81,82]. The iron center was assigned a +2.0 charge based on the 12–6 Lennard-Jones (LJ) non-bonded model [83]. Histidine residues (His499, His504, His690), which coordinate the iron, were modeled as neutral with δ-nitrogen protonation. The TIP3P water model was used for solvation, with the simulation box maintaining at least 12 Å between the solute and the box boundary. Ligand 3D structures were generated and minimized using OpenBabel (v. 3.1.1) [77] with the MMFF94 force field [84]. Ligand topologies and parameters were generated with ACPYPE [85], employing AnteChamber (AmberTools v. 22.10) [86]. Protein energy minimization was executed using GROMACS (v. 4.6.5) [87]. Ligand docking was carried out with AutoDock Vina (v. 1.2.3) [88], using a grid box centered at x = 1.35 Å, y = 14.3 Å, z = −34.60 Å, and with dimensions x = 100 Å, y = 70 Å, and z = 70 Å. The exhaustiveness was set to 10, with up to 20 docking modes generated. Docking outcomes were examined using UCSF Chimera.

#### 3.2.2. Molecular Docking Studies on COX-2

Due to lack of ovine cyclooxygenase in the PDB, the human cyclooxygenase-2 (PDB code: 1CX2) was selected, due the high homology with the ovine COX-2 which was used in the in vitro experiments. The primary sequences of the two proteins were aligned using UniProt (www.uniprot.org) and the results showed 86.4% homology. The same procedure described above was used to conduct the molecular docking studies on the COX-2. The dimensions of the grid box used were 25 Å in all three axes. The analysis of the results was performed using UCSF Chimera. The docking calculations were carried out with an exhaustiveness value of 16 and maximum output of 20 docking poses.

### 3.3. Physicochemical Studies

#### 3.3.1. In Silico Determination of miLogP

Lipophilicity was theoretically calculated as miLogP values by the Molinspiration software (https://www.molinspiration.com/ [47] (accessed on 10 May 2025).

#### 3.3.2. In Silico Determination of ADMET Properties and Drug-likeness

All compounds were subjected to in silico evaluation analysis of their drug-likeness and ADMET properties.

### 3.4. Chemistry

#### 3.4.1. Synthesis of *N*-(4-Acetylphenyl)-2-chloroacetamide (**4**) [34]

To a stirred solution of 4-aminoacetophenone (**7**) (0.79 mmol) in dichloromethane (1.2 mL), K_2_CO_3_ (2.22 mmol) was added under argon atmosphere. The reaction mixture was cooled down to 0 °C and chloroacetyl chloride (1.25 mmol) was added dropwise. The reaction mixture was allowed to stir at room temperature for 3 h. After completion of the reaction, the solvent was evaporated and the residue that obtained was washed with water and filtered to isolate compound **8**. Yield: 72.2%; white solid; M.p. 152–153 °C; ^1^H NMR (500 MHz, CDCl_3_) *δ* 8.37 (s, 1H), 8.01–7.96 (m, 2H), 7.70–7.66 (m, 2H), 4.22 (s, 2H), 2.59 (s, 3H) ppm.

#### 3.4.2. General Procedure for the Synthesis of Hybrid Compounds **5a**–**i**

To a stirred solution of the appropriate acid (1 eq) and compound **4** (1 eq) in DMF, triethylamine (2.3 eq) and potassium iodide (2.3 eq) were added. The reaction mixture was heated at 50 °C for 5 min under microwave at 50 W. After completion of the reaction, hydroxy ammonium solution 12% was added until pH = 12 and the residue that was filtered and washed with water to furnish compounds **5a**–**i**.

##### 2-((4-Acetylphenyl)amino)-2-oxoethyl Cinnamate (**5a**)

Compound **5a** was synthesized according to the general procedure from cinnamic acid **2a** (37 mg, 0.25 mmol) and **4** (53 mg, 0.25 mmol) in dry DMF (0.3 mL). Yield: 16%; white solid; R_f_: 0.48 (PE/EtOAc 6:4); M.p.: 157–159 °C; LC-MS (ESI, *m*/*z*): [M + Na]^+^ calculated for C_19_H_17_NO_4_: 346.34, found: 345.85, [M−H]^−^ calculated for C_19_H_17_NO_4_: 322.34, found: 321.90; ^1^H NMR (500 MHz, DMSO-d_6_) δ 9.57 (s, 1H, NH), 6.98 (d, *J* = 8.3 Hz, 2H), 6.83–6.71 (m, 5H), 6.48 (d, *J* = 5.0 Hz, 3H), 5.79 (d, *J* = 16.1 Hz, 1H), 3.87 (s, 2H), 1.56 (s, 3H) ppm [36].

##### 2-((4-Acetylphenyl)amino)-2-oxoethyl (E)-3-(naphthalen-1-yl)acrylate (**5b**)

Compound **5b** was synthesized according to the general procedure from cinnamic acid derivative **2b** (50 mg, 0.25 mmol) and **4** (53 mg, 0.25 mmol) in dry DMF (0.3 mL). Yield: 32%; white solid; R_f_: 0.28 (PE/EtOAc 6:4); M.p.: 162–164 °C; LC-MS (ESI, *m*/*z*): [2M]^+^ calculated for C_23_H_19_NO_4_: 746.82, found: 746.80, [M−H]^−^ calculated for C_19_H_17_NO_4_: 372.40, found: 371.90.; ^1^H NMR (500 MHz, DMSO-d_6_) δ 10.56 (s, 1H), 8.55 (d, *J* = 15.7 Hz, 1H), 8.24 (d, *J* = 8.4 Hz, 1H), 8.06 (t, *J* = 6.8 Hz, 2H), 8.01 (d, *J* = 8.1 Hz, 1H), 7.96 (d, *J* = 8.5 Hz, 2H), 7.75 (d, *J* = 8.5 Hz, 2H), 7.66 (ddd, *J* = 8.4, 6.8, 1.5 Hz, 1H), 7.60 (td, *J* = 7.7, 3.9 Hz, 2H), 6.84 (d, *J* = 15.8 Hz, 1H), 4.90 (s, 2H), 2.53 (s, 3H) ppm.; ^13^C NMR (125 MHz, DMSO-d_6_) δ 196.53, 166.12, 165.65, 142.80, 141.63, 133.32, 132.00, 130.90, 130.79, 130.56, 129.56, 128.80, 127.31, 126.39, 125.75, 125.58, 122.91, 119.88, 118.56, 62.86, 26.45 ppm.

##### 2-((4-Acetylphenyl)amino)-2-oxoethyl (E)-3-(thiophen-2-yl)acrylate (**5c**)

Compound **5c** was synthesized according to the general procedure from cinnamic acid derivative **2c** (53 mg, 0.25 mmol) and **4** (53 mg, 0.25 mmol) in dry DMF (0.3 mL). Yield: 37%; white solid; R_f_: 0.28 (PE/EtOAc 6:4); M.p.: 155–158 °C; LC-MS (ESI, *m*/*z*): [2M]^+^ calculated for C_10_H_10_NO_3_: 384.14, found: 383.90, [M−H]^−^ calculated for C_17_H_15_NO_4_S: 328.36, found: 327.95.; ^1^H NMR (500 MHz, DMSO-d_6_) *δ* 10.48 (s, 1H), 7.95 (d, *J* = 8.5 Hz, 2H), 7.90 (d, *J* = 15.7 Hz, 1H), 7.77 (d, *J* = 5.1 Hz, 1H), 7.73 (d, *J* = 8.7 Hz, 2H), 7.61 (d, *J* = 3.6 Hz, 1H), 7.18 (dd, *J* = 5.1, 3.6 Hz, 1H), 6.40 (d, *J* = 15.7 Hz, 1H), 4.82 (s, 2H), 2.53 (s, 3H) ppm.; ^13^C NMR (126 MHz, DMSO-d_6_) *δ* 196.52, 166.10, 165.54, 142.75, 138.60, 138.17, 132.53, 131.99, 130.39, 129.54, 128.66, 118.57, 115.37, 62.70, 26.44 ppm.

##### 2-((4-Acetylphenyl)amino)-2-oxoethyl (E)-3-(furan-2-yl)acrylate (**5d**)

Compound **5d** was synthesized according to the general procedure from cinnamic acid derivative **2d** (111 mg, 0.80 mmol) and **4** (130 mg, 0.66 mmol) in dry DMF (3.7 mL). Yield: 34%; off-white solid; R_f:_ 0.20 (PE/EtOAc 7:3); M.p.: 156–159 °C; LC-MS (ESI, *m*/*z*): [M+Na]^+^ calculated for C_17_H_15_NO_5_: 336.30, found: 335.80, [M−H]^−^ calculated for C_17_H_15_NO_5_: 313.31, found: 311.90.; ^1^H NMR (500 MHz, DMSO-*d*_6_) δ 10.49 (s, 1H), 7.94–7.88 (m, 3H), 7.71 (d, *J* = 6.5 Hz, 2H), 7.55 (d, *J* = 15.5 Hz, 1H), 7.02 (s, 1H), 6.66 (s, 1H), 6.34 (d, *J* = 16.3 Hz, 1H), 4.80 (s, 2H), 2.51 (s, 3H) ppm.; ^13^C NMR (126 MHz, DMSO-d_6_) δ 196.98, 166.55, 166.08, 150.58, 146.79, 143.22, 132.53, 132.42, 130.00, 119.01, 117.11, 114.31, 113.37, 63.16, 26.90 ppm.

##### 2-((4-Acetylphenyl)amino)-2-oxoethyl (E)-3-(benzo[b]thiophen-2-yl)acrylate (**5e**)

Compound **5e** was synthesized according to the general procedure from cinnamic acid derivative **2e** (90 mg, 0.44 mmol) and **4** (72 mg, 0.34 mmol) in dry DMF (2 mL). Yield: 24%; yellowish solid; R_f_: 0.57 (PE/EtOAc 5:5); M.p.: 210–213 °C; LC-MS (ESI, *m*/*z*): [M+Na]^+^ calculated for C_21_H_17_NO_4_S: 402.42, found: 401.85, [M−H]^−^ calculated for C_21_H_17_NO_4_S: 378.42, found: 377.90.; ^1^H NMR (500 MHz, DMSO-d^6^) δ 10.56 (s, 1H), 8.07–7.89 (m, 6H), 7.74 (d, *J* = 8.5 Hz, 2H), 7.44 (t, *J* = 9.4 Hz, 2H), 6.45 (d, *J* = 15.8 Hz, 1H), 4.86 (s, 2H), 2.53 (s, 3H) ppm.; ^13^C NMR (126 MHz, DMSO-d_6_) δ 196.99, 166.48, 165.68, 143.22, 140.14, 139.75, 139.19, 139.16, 132.44, 130.77, 130.01, 127.14, 125.64, 125.27, 123.24, 119.03, 118.57, 63.31, 26.91 ppm.

##### 2-((4-Acetylphenyl)amino)-2-oxoethyl (E)-3-(benzofuran-2-yl)acrylate (**5f**)

Compound **5f** was synthesized according to the general procedure from cinnamic acid derivative **2f** (150 mg, 0.80 mmol) and **4** (130 mg, 0.61 mmol) in dry DMF (3.7 mL). Yield: 30%; white solid; R_f_: 0.63 (PE/EtOAc 5:5); M.p.: 219–222 °C; LC-MS (ESI, *m*/*z*): [M+Na]^+^ calculated for C_21_H_17_NO_5_: 386.36, found: 385.90, [M−H]^−^ calculated for C_21_H_17_NO_5_: 362.36, found: 361.90.; ^1^H NMR (500 MHz, DMSO-d_6_) δ 10.56 (s, 1H), 7.95 (d, *J* = 8.7 Hz, 2H), 7.75 (t, *J* = 10.3 Hz, 4H), 7.64 (d, *J* = 8.3 Hz, 1H), 7.49–7.41 (m, 2H), 7.31 (t, *J* = 7.5 Hz, 1H), 6.61 (d, *J* = 15.8 Hz, 1H), 4.86 (s, 2H), 2.53 (s, 3H) ppm.; ^13^C NMR (126 MHz, DMSO-d_6_) δ 196.99, 166.44, 165.73, 155.47, 152.25, 143.22, 132.86, 132.44, 130.00, 128.45, 127.44, 124.12, 122.77, 119.04, 117.69, 113.22, 111.88, 63.34, 26.92 ppm.

##### 2-((4-Acetylphenyl)amino)-2-oxoethyl (E)-2,3-diphenylacrylate (**5g**)

Compound **5g** was synthesized according to the general procedure from cinnamic acid derivative **2g** (40 mg, 0.18 mmol) and **4** (29 mg, 0.14 mmol) in dry DMF (0.25 mL). Yield: 48%; white solid; R_f_: 0.38 (PE/EtOAc 6:4); M.p.: 105–108 °C (decomposition); LC-MS (ESI, *m*/*z*): [M+Na]^+^ calculated for C_25_H_21_NO_4_: 422.44, found: = 421.90, [M−H]^−^ calculated for C_25_H_21_NO_4_: 398.44, found: = 397.95.; ^1^H NMR (500 MHz, DMSO-d_6_) δ 10.52 (s, 1H), 7.95 (d, *J* = 8.4 Hz, 2H), 7.89 (s, 1H), 7.72 (d, *J* = 8.3 Hz, 2H), 7.40 (dq, *J* = 12.8, 7.2 Hz, 3H), 7.25 (dq, *J* = 20.5, 7.2 Hz, 5H), 7.09 (d, *J* = 7.7 Hz, 2H), 4.85 (s, 2H), 2.53 (s, 3H) ppm.; ^13^C NMR (126 MHz, DMSO-d_6_) δ 196.50, 166.52, 165.95, 142.77, 140.51, 135.39, 134.05, 131.94, 131.78, 130.35, 129.57 (d, *J* = 3.0 Hz), 129.46, 128.69, 128.38, 128.00, 118.42, 63.24, 26.43 ppm.

##### 2-((4-Acetylphenyl)amino)-2-oxoethyl (E)-3-(naphthalen-1-yl)-2-phenylacrylate (**5h**)

Compound **5h** was synthesized according to the general procedure from cinnamic acid derivative **2h** (100 mg, 0.36 mmol) and **4** (60 mg, 0.28 mmol) in dry DMF (0.5 mL). Yield: 52%; yellowish solid; R_f_: 0.35 (PE/EtOAc 6:4); M.p.: 120–122 °C (decomposition); LC-MS (ESI, *m*/*z*): [M+Na]^+^ calculated for C_29_H_23_NO_4_: 472.15, found: = 471.90, [M−H]^−^ calculated for C_29_H_23_NO_4_: 449.50, found: 447.95.; ^1^H NMR (500 MHz, DMSO-d_6_) δ 10.59 (s, 1H), 8.47 (s, 1H), 8.08 (d, *J* = 8.4 Hz, 1H), 7.99–7.93 (m, 3H), 7.84 (d, *J* = 8.2 Hz, 1H), 7.75 (d, *J* = 8.7 Hz, 2H), 7.67–7.55 (m, 2H), 7.30–7.19 (m, 4H), 7.16 (dd, *J* = 6.6, 3.1 Hz, 2H), 7.01 (d, *J* = 7.2 Hz, 1H), 4.94 (s, 2H), 2.54 (s, 3H) ppm.; ^13^C NMR (126 MHz, DMSO-d_6_) δ 196.51, 166.44, 166.01, 142.79, 138.71, 134.86, 134.46, 132.97, 131.97, 131.73, 131.19, 129.93, 129.58, 128.95, 128.64, 128.06, 127.68, 127.58, 126.93, 126.34, 125.13, 123.85, 118.47, 63.38, 26.44 ppm.

##### 2-((4-Acetylphenyl)amino)-2-oxoethyl (E)-2-phenyl-3-(thiophen-2-yl)acrylate (**5i**)

Compound **5i** was synthesized according to the general procedure from cinnamic acid derivative **2i** (100 mg, 0.44 mmol) and **4** (72 mg, 0.34 mmol) in dry DMF (1 mL). Yield: 54%; brown solid; R_f_: 0.35 (PE/EtOAc 6:4); M.p.: 137–140 °C (decomposition); LC-MS (ESI, *m*/*z*): [M+Na]^+^ calculated for C_23_H_19_NO_4_S: 428.46, found: 427.75.; ^1^H NMR (500 MHz, DMSO-d_6_) δ 10.51 (s, 1H), 8.14 (s, 1H), 7.94 (d, *J* = 8.5 Hz, 2H), 7.70 (d, *J* = 8.5 Hz, 2H), 7.58 (t, *J* = 7.0 Hz, 1H), 7.52–7.43 (m, 4H), 7.28 (d, *J* = 7.2 Hz, 2H), 7.05 (t, *J* = 4.5 Hz, 1H), 4.81 (s, 2H), 2.53 (s, 3H) ppm.; ^13^C NMR (126 MHz, DMSO-d_6_) δ 196.57, 166.22, 166.04, 142.81, 137.67, 135.09, 134.80, 134.36, 132.28, 131.95, 129.89, 129.59, 129.16, 128.76, 128.64, 128.12, 127.03, 118.45, 63.11, 26.46 ppm.

#### 3.4.3. General Procedure for the Synthesis of Hybrid Compounds **6a**–**i**

To a stirred solution of the appropriate ketone derivative **5a**–**i** (1 eq) in EtOH, sodium acetate (3.5 eq) and NH_2_OH·HCl (7 eq) were added. The reaction mixture is heated at 50 °C for 5–15 min under microwave at 50W. After completion of the reaction, water was added and the obtained residue was filtered to furnish compounds **6a**–**i**.

##### 2-((4-((E)-1-(Hydroxyimino)ethyl)phenyl)amino)-2-oxoethyl cinnamate (**6a**)

Compound **6a** was synthesized according to the general procedure from compound **5a** (17 mg, 0.054 mmol) in EtOH (0.36 mL) for 5 min. Yield: 70%; white solid; R_f_: 0.48 (PE/EtOAc 6:4); M.p.: 178–180 °C; LC-MS (ESI, *m*/*z*): [M+Na]^+^ calculated for C_19_H_18_N_2_O_4_: 361.35, found: 360.85, [M+Na+MeOH]^+^ calculated for C_19_H_18_N_2_O_4_: 393.39, found: 392.90, [M−H]^−^ calculated for C_19_H_18_N_2_O_4_: 337.35, found: 336.90.; ^1^H NMR (500 MHz, DMSO-d_6_) *δ* 10.14 (s, 1H), 9.31 (s, 1H), 6.81–6.75 (m, 3H), 6.68–6.61 (m, 4H), 6.49 (dd, *J* = 4.9, 1.9 Hz, 3H), 5.79 (d, *J* = 16.1 Hz, 1H), 3.84 (s, 2H), 1.16 (s, 3H) ppm.; ^13^C NMR (126 MHz, DMSO-d_6_) *δ* 165.96, 165.72, 152.59, 145.54, 138.89, 134.02, 132.24, 129.14, 128.62, 126.21, 119.12, 117.56, 117.52, 62.81, 11.48 ppm.

##### 2-((4-((E)-1-(Hydroxyimino)ethyl)phenyl)amino)-2-oxoethyl (E)-3-(naphthalen-1-yl)acrylate (**6b**)

Compound **6b** was synthesized according to the general procedure from compound **5b** (20 mg, 0.054 mmol) in EtOH (0.36 mL) for 5 min. Yield: 85%; white solid; R_f_: 0.5 (PE/EtOAc 6:4); M.p.: 165–168 °C; HRMS (ESI, *m*/*z*): [M+H]^+^ calculated for C_23_H_20_N_2_O_4_: 389.1496, found: 389.1489, [M−H]^−^ calculated for C_23_H_20_N_2_O_4_: 387.1345, found: 387.1342.; LC-MS (ESI, *m*/*z*): [M+Na+MeOH]^+^ calculated for C_10_H_11_N_2_O_3_: 262.11, found: 262.85, [M−H]^−^ calculated for C_19_H_18_N_2_O_4_: 206.07, found: = 206.90.; ^1^H NMR (500 MHz, DMSO-d_6_) *δ* 11.08 (s, 1H), 10.29 (s, 1H), 8.55 (d, *J* = 15.8 Hz, 1H), 8.24 (d, *J* = 8.4 Hz, 1H), 8.04 (dt, *J* = 16.6, 7.9 Hz, 3H), 7.68–7.58 (m, 7H), 6.84 (d, *J* = 15.7 Hz, 1H), 4.87 (s, 2H), 2.13 (s, 3H) ppm.; ^13^C NMR (126 MHz, DMSO-d_6_) *δ* 165.66, 165.58, 152.38, 141.55, 138.83, 133.33, 132.12, 130.88, 130.80, 130.59, 128.80, 127.32, 126.40, 126.10, 125.76, 125.57, 122.93, 119.99, 119.01, 62.85, 11.37 ppm.

##### 2-((4-((E)-1-(Hydroxyimino)ethyl)phenyl)amino)-2-oxoethyl (E)-3-(thiophen-2-yl)acrylate (**6c**)

Compound **6c** was synthesized according to the general procedure from compound **5c** (18 mg, 0.054 mmol) in EtOH (0.36 mL) for 5 min. Yield: 80%; white solid; R_f_: 0.48 (PE/EtOAc 5:5); M.p.: 170–172 °C; HRMS (ESI, *m*/*z*): [M + H]^+^ calculated for C_17_H_16_N_2_O_4_S: 345.0909, found: 345.0902, [M−H]^−^ calculated for C_17_H_16_N_2_O_4_S: 343.0752, found: 343.0750.; LC-MS (ESI, *m*/*z*): [M+Na+MeOH]^+^ calculated for C_17_H_16_N_2_O_4_S: 399.11, found: 398.90, [M+Na]^+^ calculated for C_17_H_16_N_2_O_4_S: 367.07, found: 366.85, [M−H]^−^ calculated for C_17_H_16_N_2_O_4_S: 343.07, found: 342.95.; ^1^H NMR (500 MHz, DMSO-d_6_) *δ* 11.08 (s, 1H), 10.22 (s, 1H), 7.90 (d, *J* = 15.8 Hz, 1H), 7.77 (d, *J* = 5.0 Hz, 1H), 7.61 (s, 5H), 7.17 (dd, *J* = 5.1, 3.6 Hz, 1H), 6.39 (d, *J* = 15.8 Hz, 1H), 4.78 (s, 2H), 2.12 (s, 3H) ppm.; ^13^C NMR (126 MHz, DMSO-d_6_) *δ* 165.55 (d, *J* = 1.8 Hz), 152.36, 138.78, 138.63, 138.09, 132.49, 132.11, 130.34, 128.66, 126.06, 119.02, 115.48, 62.69, 11.36 ppm.

##### 2-((4-((E)-1-(Hydroxyimino)ethyl)phenyl)amino)-2-oxoethyl (E)-3-(furan-2-yl)acrylate (**6d**)

Compound **6d** was synthesized according to the general procedure from compound **5d** (18 mg, 0.054 mmol) in EtOH (0.36 mL) for 15 min. Yield: 94%; off-white solid; R_f_: (PE/EtOAc 7:3) 0.13; M.p.: 185–187 °C; HRMS (ESI, *m*/*z*): [M+H]^+^ calculated for C_17_H_16_N_2_O_5_: 329.1137, found: 329.1131, [M−H]^−^ calculated for C_17_H_17_N_2_O_5_Ν_2_: 327.0981, found: = 327.0979.; LC-MS (ESI, *m*/*z*): [M+Na]^+^ calculated for C_17_H_16_N_2_O_5_: 351.10, found: 350.85, [M+ Na+MeOH]^+^ calculated for C_17_H_16_N_2_O_5_: 383.13, found: 382.85, [M−H]^−^ calculated for C_17_H_16_N_2_O_5_: 327.10, found: 326.85.; ^1^H NMR (500 MHz, DMSO-d_6_) *δ* 11.09 (s, 1H), 10.24 (s, 1H), 7.88 (s, 1H), 7.61 (t, *J* = 6.9 Hz, 4H), 7.40 (t, *J* = 8.0 Hz, 3H), 7.25 (tt, *J* = 14.9, 7.5 Hz, 5H), 7.09 (d, *J* = 16.6 Hz, 2H), 4.78 (s, 2H), 2.12 (s, 3H) ppm.; ^13^C NMR (126 MHz, DMSO-d_6_) *δ* 187.19, 182.04, 165.65, 150.15, 146.34, 138.82, 132.00, 126.08, 119.03, 118.57, 116.61, 114.02, 112.93, 62.70, 11.37 ppm.

##### 2-((4-((E)-1-(Hydroxyimino)ethyl)phenyl)amino)-2-oxoethyl (E)-3-(benzo[b]thiophen-2-yl)acrylate (**6e**)

Compound **6e** was synthesized according to the general procedure from compound **5e** (30 mg, 0.067 mmol) in EtOH (0.6 mL) for 5 min. Yield: 87%; yellowish solid; R_f_: 0.18 (PE/EtOAc 7:3); M.p.: 177–179 °C; HRMS (ESI, *m*/*z*): [M+H]^+^ calculated for C_21_H_18_N_2_O_4_S: 395.1065), found: 395.1057, [M−H]^−^ calculated for C_21_H_18_N_2_O_4_S: 393.0909, found: 393.0904.; LC-MS (ESI, *m*/*z*): [M+Na]^+^ calculated for C_21_H_18_N_2_O_4_S: 417.09, found: 416.85, [M+Na+MeOH]^+^ calculated for C_21_H_18_N_2_O_4_S: 449.12, found: 449.00, [M−H]^−^ calculated for C_21_H_18_N_2_O_4_S: 393.09, found: 392.90.; ^1^H NMR (500 MHz, DMSO-d_6_) *δ* 11.06 (s, 1H), 10.23 (s, 1H), 7.73–7.69 (s, 2H), 7.61–7.59 (m, 5H), 7.42–7.39 (m, 2H), 7.29–7.26 (t, *J* = 7.4 Hz, 1H), 6.58 (d, *J* = 15.9 Hz, 1H), 4.80 (s, 2H), 2.09 (s, 3H) ppm.; ^13^C NMR (126 MHz, DMSO-d_6_) δ 165.62, 165.45, 155.19, 152.53, 152.00, 138.94, 132.48, 132.30, 128.18, 127.15, 126.24, 123.84, 122.48, 119.22, 117.52, 112.85, 111.60, 63.05, 11.53 ppm.

##### 2-((4-((E)-1-(Hydroxyimino)ethyl)phenyl)amino)-2-oxoethyl (E)-3-(benzofuran-2-yl)acrylate (**6f**)

Compound **6f** was synthesized according to the general procedure from compound **5f** (30 mg, 0.082 mmol) in EtOH (2 mL) for 30 min. Yield: 87%; white solid; Rf: 0.16 (PE/EtOAc 7:3); M.p.: 222–225 °C (decomposition); HRMS (ESI, *m*/*z*): [M+H]^+^ calculated for C_21_H_18_N_2_O_5_: 379.1294, found: 379.1289, [M−H]^−^ calculated for C_21_H_18_N_2_O_5_: 377.1137, found: 377.1135.; LC-MS (ESI, *m*/*z*): [M+Na]^+^ calculated for C_21_H_18_N_2_O_5_: 401.11, found: 400.85, [M+Na+MeOH]^+^ calculated for C_21_H_18_N_2_O_5_: 433.14, found: 432.85, [M−H]^−^ calculated for C_21_H_18_N_2_O_5_: 377.11, found: 376.95.; ^1^H NMR (500 MHz, DMSO-*d*_6_) δ 11.09 (s, 1H), 10.26 (s, 1H), 7.78–7.71 (m, 2H), 7.62 (s, 5H), 7.48–7.41 (m, 2H), 7.31 (t, *J* = 7.5 Hz, 1H), 6.61 (d, *J* = 15.8 Hz, 1H), 4.83 (s, 2H), 2.13 (s, 3H) ppm.; ^13^C NMR (126 MHz, DMSO-d_6_) δ 165.45, 165.28, 155.02, 152.36, 151.83, 138.78, 132.32, 132.13, 128.02, 126.98, 126.07, 123.67, 122.31, 119.05, 117.35, 112.68, 111.43, 62.88, 11.37 ppm.

##### 2-((4-((E)-1-(Hydroxyimino)ethyl)phenyl)amino)-2-oxoethyl (E)-2,3-diphenylacrylate (**6g**)

Compound **6g** was synthesized according to the general procedure from compound **5g** (30 mg, 0.075 mmol) in EtOH (2 mL) for 15 min. Yield: 94%; white solid; R_f_: 0.6 (PE/EtOAc 4:6); M.p.:185–187 °C (decomposition); HRMS (ESI, *m*/*z*): [M+H]^+^ calculated for C_25_H_22_N_2_O_4_: 415.1658, found: 415.1650, [M−H]^−^ calculated for C_21_H_18_N_2_O_5_: 413.1501, found: 413.1499.; LC-MS (ESI, *m*/*z*): [M+Na]^+^ calculated for C_25_H_22_N_2_O_4_: 437.15, found: 436.85, [M−H]^−^ calculated for C_25_H_22_N_2_O_4_: 413.15, found: 413.00.; ^1^H NMR (500 MHz, DMSO-*d*_6_) δ 11.07 (s, 1H), 10.24 (s, 1H), 7.88 (s, 1H), 7.61 (t, *J* = 6.9 Hz, 4H), 7.40 (t, *J* = 8.0 Hz, 3H), 7.25 (tt, *J* = 14.9, 7.5 Hz, 5H), 7.09 (d, *J* = 7.6 Hz, 2H), 4.81 (s, 2H), 2.12 (s, 3H) ppm.; ^13^C NMR (126 MHz, DMSO-*d*_6_) δ 166.58, 165.47, 152.40, 140.52, 138.87, 135.48, 134.11, 132.07, 131.87, 130.41, 129.65, 129.51, 128.75, 128.44, 128.06, 126.15, 118.86, 63.28, 11.41 ppm.

##### 2-((4-((E)-1-(Hydroxyimino)ethyl)phenyl)amino)-2-oxoethyl (E)-3-(naphthalen-1-yl)-2-phenylacrylate (**6h**)

Compound **6h** was synthesized according to the general procedure from compound **5h** (30 mg, 0.067 mmol) in EtOH (0.6 mL) for 5 min. Yield: 87%; yellowish solid; R_f_: 0.6 (PE/EtOAc 5:5); M.p.: 177–179 °C (decomposition); HRMS (ESI, *m*/*z*): [M+H]^+^ calculated for C_29_H_24_N_2_O_4_: 465.1814, found: 465.1806, [M−H]^−^ calculated for C_29_H_24_N_2_O_4_: 463.1658, found: 463.1654.; LC-MS (ESI, *m*/*z*): [M+Na]^+^ calculated for C_29_H_24_N_2_O_4_: 487.16, found: 486.95, [M−H]^−^ calculated for C_29_H_24_N_2_O_4_: 463.50, found: 463.00.; ^1^H NMR (500 MHz, DMSO-*d*_6_) δ 11.08 (s, 1H), 10.32 (s, 1H), 8.46 (s, 1H), 8.08 (d, *J* = 8.3 Hz, 1H), 7.95 (d, *J* = 7.8 Hz, 1H), 7.84 (d, *J* = 8.2 Hz, 1H), 7.63 (d, *J* = 8.3 Hz, 4H), 7.61–7.55 (m, 2H), 7.30–7.20 (m, 4H), 7.20–7.12 (m, 2H), 7.01 (d, *J* = 7.1 Hz, 1H), 4.90 (s, 2H), 2.13 (s, 3H) ppm.; ^13^C NMR (126 MHz, DMSO-*d*_6_) δ 166.91, 152.81, 147.33, 139.30, 139.11, 135.35, 134.98, 133.43, 132.23, 131.66, 130.40, 130.30, 129.39, 129.10, 128.51, 128.13, 128.04, 127.39, 126.81, 126.57, 125.59, 124.33, 119.33, 63.83, 11.82 ppm.

##### 2-((4-((E)-1-(Hydroxyimino)ethyl)phenyl)amino)-2-oxoethyl (E)-2-phenyl-3-(thiophen-2-yl)acrylate (**6i**)

Compound **6i** was synthesized according to the general procedure from compound **5i** (31 mg, 0.067 mmol) in EtOH 1 mL) for 15 min. Yield: 79%; yellow brown solid; R_f_: 0.58 (PE/EtOAc 5:5); M.p.: 199–201 °C; HRMS (ESI, *m*/*z*): [M+H]^+^ calculated for C_23_H_20_N_2_O_4_S: 421.1222, found: 421.1218, [M−H]^−^ calculated for C_23_H_20_N_2_O_4_S: 419.1065, found: 419.1064.; LC-MS (ESI, *m*/*z*): [M+Na]^+^ calculated for C_23_H_20_N_2_O_4_S: 443.10, found: 442.85.; ^1^H NMR (500 MHz, DMSO-*d*_6_) δ 11.08 (s, 1H), 10.21 (s, 1H), 8.14 (s, 1H), 7.60 (dt, *J* = 10.4, 6.2 Hz, 5H), 7.47 (h, *J* = 8.9, 8.0 Hz, 4H), 7.29 (d, *J* = 7.2 Hz, 2H), 7.05 (q, *J* = 5.9, 4.3 Hz, 1H), 4.78 (s, 2H), 2.12 (s, 3H) ppm.; ^13^C NMR (126 MHz, DMSO-*d*_6_) δ 166.17, 165.45, 152.35, 138.81, 137.67, 135.02, 134.25, 132.20, 129.87, 129.11, 128.72, 128.58, 128.16, 126.98, 126.27, 126.08, 118.83, 63.09, 11.35 ppm.

#### 3.4.4. Synthesis of 3-(Hydroxymethyl)-4-phenyl-1,2,5-oxadiazole 2-oxide (**8**)

NaNO_2_ (1.8 g, 26.0 mmol, 7.0 eq) is gradually added to 5–10 °C over 2 h in a solution of cinnamyl alcohol (0.5 mL, 3.90 mmol, 1 eq) in AcOH (8 mL). The reaction mixture was then allowed to warm to room temperature and stirred for 24 h. Upon completion of the reaction, water was added, and the mixture was extracted with EtOAc (5×). The combined organic layers were dried over anhydrous MgSO_4_, filtered, and concentrated under reduced pressure. The crude oily product was purified by flash column chromatography with silica gel, using a mixture of PE/EtOAc (95:5 *v*/*v*) as the eluent to obtain compound 8. Yield: 62%; white solid; ^1^H NMR (500 MHz, CDCl_3_) δ 7.86–7.77 (m, 2H), 7.56 (q, *J* = 6.6 Hz, 3H), 4.75 (s, 2H), 2.76 (br, 1H) ppm.; ^13^C NMR (126 MHz, CDCl_3_) δ 156.97, 131.51, 129.55, 127.92, 126.30, 114.99, 53.48 ppm [37].

#### 3.4.5. General Procedure for the Synthesis of Hybrid Compounds **9a**–**i**

To a solution of the appropriate cinnamic acid **2a**–**i** in dry DMF, the reagents EDCI·HCl, DMAP, and compound **8** were added. The reaction mixture was heated at 50 °C in a microwave oven at 50 W for 5–45 min. After completion of the reaction, water was added, and the mixture was extracted with EtOAc (×2). The combined organic layers were washed with brine, dried over anhydrous MgSO_4_, filtered, and concentrated under reduced pressure. The crude yellow oily product was purified by flash column chromatography with silica gel, using a PE/EtOAc solvent mixture as eluent to isolate compounds **9a**–**i**.

##### 3-((Cinnamoyloxy)methyl)-4-phenyl-1,2,5-oxadiazole 2-oxide (**9a**)

Compound **9a** was synthesized according to the general procedure for the preparation of the hybrid compounds from cinnamic acid **2a** (20 mg, 0.14 mmol, 1.2 equiv), EDCI·HCl (40 mg, 0.21 mmol, 1.1 equiv), DMAP (26 mg, 0.21 mmol, 1.1 equiv), and compound **8** (22 mg, 0.11 mmol, 1.0 equiv) in dry DMF (1.7 mL), with a total reaction time of 5 min. After purification by flash column chromatography using a PE/EtOAc 95:5 *v*/*v* eluent mixture, compound **9a** was obtained. Yield: 40%; yellow oily solid; R_f_: 0.71 (PE/EtOAc 75:25); LC-MS (ESI, *m*/*z*): [M+Na]^+^ calculated for C_18_H_14_N_2_O_4_: 345.09, found: = 344.85, ^1^H NMR (500 MHz, CDCl_3_) δ 7.76–7.73 (m, 2H), 7.71 (d, *J* = 16.0 Hz, 1H), 7.60–7.49 (m, 5H), 7.43–7.37 (m, 3H), 6.43 (d, *J* = 16.0 Hz, 1H), 5.28 (s, 2H) ppm.; ^13^C NMR (126 MHz, CDCl_3_) δ 166.03, 156.94, 147.02, 134.01, 131.56, 131.02, 129.58, 129.14, 128.44, 127.79, 126.22, 116.21 (d, *J* = 1.4 Hz), 111.52, 54.35 ppm.

##### 3-(((3-(Naphthalen-1-yl)acryloyl)oxy)methyl)-4-phenyl-1,2,5-oxadiazole 2-oxide (**9b**)

Compound **9b** was synthesized according to the general procedure for the preparation of the hybrid compounds from cinnamic acid **2b** (28 mg, 0.14 mmol, 1.2 equiv), EDCI·HCl (40 mg, 0.21 mmol, 1.1 equiv), DMAP (26 mg, 0.21 mmol, 1.1 equiv), and compound **8** (22 mg, 0.11 mmol, 1.0 equiv) in dry DMF (1.7 mL), with a total reaction time of 5 min. After purification by flash column chromatography using a PE/EtOAc 95:5 *v*/*v* eluent mixture, compound **9b** was obtained. Yield: 66%; yellow oily solid; R_f_: 0.66 (PE/EtOAc 75:25); HRMS (ESI, *m*/*z*): [M+H]^+^ calculated for C_22_H_16_N_2_O_4_: 373.1188, found: 373.1173, [M−H]^−^ calculated for C_22_H_16_N_2_O_4_: 371.1032, found: 371.1036.; LC-MS (ESI, *m*/*z*): [M+Na]^+^ calculated for C_22_H_16_N_2_O_4_: 395.10, found: 394.85.; ^1^H NMR (500 MHz, CDCl_3_) δ 8.56 (d, *J* = 15.7 Hz, 1H), 8.12 (d, *J* = 8.3 Hz, 1H), 7.90 (dd, *J* = 18.7, 8.0 Hz, 2H), 7.85–7.66 (m, 3H), 7.57 (h, *J* = 7.4 Hz, 5H), 7.52–7.44 (m, 1H), 6.53 (d, *J* = 15.7 Hz, 1H), 5.33 (s, 2H) ppm.; ^13^C NMR (126 MHz, CDCl_3_) δ 165.90, 156.93, 143.98, 133.79, 131.47, 131.24, 129.52, 128.97, 128.90, 127.84, 127.75, 126.42, 126.23, 125.49, 123.30, 123.28, 118.71, 118.62, 111.53, 54.43 ppm.

##### (E)-4-Phenyl-3-(((3-(thiophen-2-yl)acryloyl)oxy)methyl)-1,2,5-oxadiazole 2-oxide (**9c**)

Compound **9c** was synthesized according to the general procedure for the preparation of the hybrid compounds from cinnamic acid **2c** (30 mg, 0.19 mmol, 1.2 equiv), EDCI·HCl (56 mg, 0.29 mmol, 1.8 equiv), DMAP (35 mg, 0.29 mmol, 1.8 equiv), and compound **8** (30 mg, 0.16 mmol, 1.0 equiv) in dry DMF (2.3 mL), with a total reaction time of 5 min. After purification by flash column chromatography using a PE/EtOAc 95:5 *v*/*v* eluent mixture, compound **9c** was obtained. Yield: 50%; pink solid; R_f_: 0.63 (PE/EtOAc 75:25); M.p.: 57–59 °C; HRMS (ESI, *m*/*z*): [M+H]^+^ calculated for C_16_H_12_N_2_O_4_S: 329.0596, found: 329.0589, [M−H]^−^ calculated for C_16_H_12_N_2_O_4_S: 327.0439, found: 327.0438.; LC-MS (ESI, *m*/*z*): [M+Na]^+^ calculated for C_16_H_12_N_2_O_4_S: 351.04, found: 350.85; ^1^H NMR (500 MHz, CDCl_3_) δ 7.79 (d, *J* = 15.7 Hz, 1H), 7.76–7.70 (m, 2H), 7.55 (q, *J* = 6.7, 6.2 Hz, 3H), 7.42 (d, *J* = 5.0 Hz, 1H), 7.27 (d, *J* = 3.6 Hz, 1H), 7.08–7.06 (m, 1H), 6.21 (d, *J* = 15.7 Hz, 1H), 5.26 (s, 2H) ppm.; ^13^C NMR (126 MHz, CDCl_3_) δ 165.88, 156.92, 139.28, 139.16, 131.96, 131.55, 129.57, 129.51, 128.40, 127.78, 126.20, 114.77, 111.51, 54.30 ppm.

##### (E)-3-(((3-(Furan-2-yl)acryloyl)oxy)methyl)-4-phenyl-1,2,5-oxadiazole 2-oxide (**9d**)

Compound **9d** was synthesized according to the general procedure for the preparation of the hybrid compounds from cinnamic acid **2d** (30 mg, 0.19 mmol, 1.2 equiv), EDCI·HCl (60 mg, 0.32 mmol, 2.0 equiv), DMAP (39 mg, 0.32 mmol, 2.0 equiv), and compound **8** (31 mg, 0.16 mmol, 1.0 equiv) in dry DMF (2.5 mL), with a total reaction time of 10 min. After purification by flash column chromatography using a PE/EtOAc 95:5 *v*/*v* eluent mixture, compound **9d** was obtained. Yield: 58%; yellow oily solid; R_f_: 0.73 (PE/EtOAc 7:3); HRMS (ESI, *m*/*z*): [M+H]^+^ calculated for C_23_H_20_N_2_O_4_S: 313.0824, found: 313.0817.; LC-MS (ESI, *m*/*z*): [M+Na]^+^ calculated for C_16_H_12_N_2_O_5_: 335.06, found: 334.80.; ^1^H NMR (500 MHz, CDCl_3_) δ 7.75–7.73 (m, 2H), 7.58–7.50 (m, 4H), 7.45–7.42 (m, 1H), 6.65 (d, *J* = 3.5 Hz, 1H), 6.48 (dt, *J* = 3.2, 1.4 Hz, 1H), 6.30 (d, *J* = 15.7 Hz, 1H), 5.25 (d, *J* = 1.2 Hz, 2H) ppm.; ^13^C NMR (126 MHz, CDCl_3_) δ 166.07, 156.94, 150.60, 145.49, 132.92, 131.53, 129.55, 127.76, 126.18, 116.20, 113.64, 112.66, 111.52, 54.27 ppm.

##### (E)-3-(((3-(Benzo[b]thiophen-2-yl)acryloyl)oxy)methyl)-4-phenyl-1,2,5-oxadiazole 2-oxide (**9e**)

Compound **9e** was synthesized according to the general procedure for the preparation of the hybrid compounds from cinnamic acid **2e** (35 mg, 0.17 mmol, 1.3 equiv), EDCI·HCl (49 mg, 0.26 mmol, 2.0 equiv), DMAP (31 mg, 0.26 mmol, 2.0 equiv), and compound **8** (25 mg, 0.13 mmol, 1.0 equiv) in dry DMF (2 mL), with a total reaction time of 10 min. After purification by flash column chromatography using a PE/EtOAc 95:5 *v*/*v* eluent mixture, compound **9d** was obtained. Yield: 57%; yellowish solid; R_f_: 0.76 (PE/EtOAc 7:3); M.p.: 120–123 °C; HRMS (ESI, *m*/*z*): [M+H]^+^ calculated for C_20_H_14_N_2_O_4_S: 379.0752, found: 379.0742, [M−H]^−^ calculated for C_20_H_14_N_2_O_4_S: 377.0596, found: 377.0597.; LC-MS (ESI, *m*/*z*): [M+Na]^+^ calculated for C_20_H_14_N_2_O_4_S: 401.06, found: 400.80.; ^1^H NMR (500 MHz, CDCl_3_) δ 7.89 (d, *J* = 15.6 Hz, 1H), 7.82–7.70 (m, 4H), 7.57 (d, *J* = 5.4 Hz, 3H), 7.49 (s, 1H), 7.38 (t, *J* = 8.1 Hz, 2H), 6.28 (d, *J* = 17.4 Hz, 1H), 5.29 (s, 2H) ppm.; ^13^C NMR (126 MHz, CDCl_3_) δ 165.60, 156.92, 140.56, 139.87, 139.53, 139.02, 131.58, 129.89, 129.59, 127.79, 126.76, 126.16, 125.15, 124.80, 122.65, 117.22, 111.47, 54.42 ppm.

##### (E)-3-(((3-(Benzofuran-2-yl)acryloyl)oxy)methyl)-4-phenyl-1,2,5-oxadiazole 2-oxide (**9f**)

Compound **9f** was synthesized according to the general procedure for the preparation of the hybrid compounds from cinnamic acid **2f** (40 mg, 0.21 mmol, 1.3 equiv), EDCI·HCl (60 mg, 0.32 mmol, 2.0 equiv), DMAP (39 mg, 0.32 mmol, 2.0 equiv), and compound **8** (31 mg, 0.16 mmol, 1.0 equiv) in dry DMF (2.5 mL), with a total reaction time of 10 min. After purification by flash column chromatography using a PE/EtOAc 95:5 *v*/*v* eluent mixture, compound **9f** was obtained. Yield: 73%; white solid; R_f_: 0.76 (PE/EtOAc 7:3); M.p.: 114–117 °C; HRMS (ESI, *m*/*z*): [M+H]^+^ calculated for C_20_H_14_N_2_O_5_: 363.0981, found: 363.0975.; LC-MS (ESI, *m*/*z*): [M+Na]^+^ calculated for C_20_H_14_N_2_O_5_: 385.08, found: 384.85.; ^1^H NMR (500 MHz, CDCl_3_) δ 7.77–7.75 (m, 2H), 7.61–7.55 (m, 5H), 7.48 (d, *J* = 9.2 Hz, 1H), 7.40–7.37 (m, 1H), 7.28–7.24 (m, 1H), 6.98 (d, *J* = 1.9 Hz, 1H), 6.57 (dd, *J* = 15.6, 1.9 Hz, 1H), 5.29 (d, *J* = 1.9 Hz, 2H) ppm.; ^13^C NMR (126 MHz, CDCl_3_) δ 165.74, 156.94, 155.84, 151.92, 133.27, 131.57, 129.59, 128.34, 127.78, 127.07, 126.18, 123.64, 122.11, 116.68, 112.52, 111.62, 111.45, 54.45 ppm.

##### (E)-3-(((2,3-Diphenylacryloyl)oxy)methyl)-4-phenyl-1,2,5-oxadiazole 2-oxide (**9g**)

Compound **9g** was synthesized according to the general procedure for the preparation of the hybrid compounds from cinnamic acid **2g** (42 mg, 0.19 mmol, 1.9 equiv), EDCI·HCl (48 mg, 0.25 mmol, 2.5 equiv), DMAP (18 mg, 0.25 mmol, 2.5 equiv), and compound **8** (21 mg, 0.10 mmol, 1.0 equiv) in dry DMF (2.5 mL), with a total reaction time of 45 min. After purification by flash column chromatography using a PE/EtOAc 92:8 *v*/*v* eluent mixture, compound **9g** was obtained. Yield: 87%; yellow sticky solid; R_f_: 0.68 (PE/EtOAc 75:25); M.p.: 91–94 °C; HRMS (ESI, *m*/*z*): [M+H]^+^ calculated for C_24_H_18_N_2_O_4_: 399.1345, found: 399.1333, [M−H]^−^ calculated for C_24_H_18_N_2_O_4_: 397.1188, found: 397.1184.; LC-MS (ESI, *m*/*z*): [M+Na]^+^ calculated for C_24_H_18_N_2_O_4_: 421.12, found: 420.85, [M−H]^−^ calculated for C_24_H_18_N_2_O_4_: 397.12, found: 396.90.; ^1^H NMR (500 MHz, CDCl_3_) δ 7.83 (s, 1H), 7.61 (dd, *J* = 7.1, 1.4 Hz, 2H), 7.58–7.51 (m, 1H), 7.46 (t, *J* = 7.6 Hz, 2H), 7.35 (dd, *J* = 4.6, 2.0 Hz, 3H), 7.27–7.19 (m, 1H), 7.18–7.08 (m, 4H), 7.01 (d, *J* = 7.7 Hz, 2H), 5.26 (s, 2H) ppm.; ^13^C NMR (126 MHz, CDCl_3_) δ 171.29, 166.88, 157.00, 142.49, 135.24, 134.21, 131.43, 131.07, 130.97, 129.73, 129.46, 128.97, 128.43, 128.25, 127.81, 126.19, 111.41, 54.92 ppm.

##### (E)-3-(((3-(Naphthalen-1-yl)-2-phenylacryloyl)oxy)methyl)-4-phenyl-1,2,5-oxadiazole 2-oxide (**9h**)

Compound **9h** was synthesized according to the general procedure for the preparation of the hybrid compounds from cinnamic acid **2h** (52 mg, 0.19 mmol, 1.2 equiv), EDCI·HCl (72 mg, 0.38 mmol, 2.3 equiv), DMAP (46 mg, 0.38 mmol, 2.3 equiv), and compound **8** (30 mg, 0.16 mmol, 1.0 equiv) in dry DMF (1.2 mL), with a total reaction time of 45 min. After purification by flash column chromatography using a PE/EtOAc 95:5 *v*/*v* eluent mixture, compound **9h** was obtained. Yield: 67%; yellow sticky solid; R_f_: 0.66 (PE/EtOAc 75:25); M.p.: 114–117 °C; HRMS (ESI, *m*/*z*): [M]^+^ calculated for C_28_H_20_N_2_O_4_: 448.1423, found: 448.1415, [M−H]^−^ calculated for C_28_H_20_N_2_O_4_: 447.1345, found: 447.1338.; LC-MS (ESI, *m*/*z*): [M+Na]^+^ calculated for C_28_H_20_N_2_O_4_: 471.13, found: 470.85, [M−H]^−^ calculated for C_28_H_20_N_2_O_4_: 447.13, found: 446.80.; ^1^H NMR (500 MHz, CDCl_3_) δ 8.49 (s, 1H), 8.01 (d, *J* = 8.2 Hz, 1H), 7.86–7.80 (m, 1H), 7.74–7.65 (m, 3H), 7.60–7.46 (m, 5H), 7.24–7.16 (m, 3H), 7.13 (t, *J* = 7.7 Hz, 1H), 7.06 (dt, *J* = 6.6, 1.6 Hz, 2H), 6.95 (d, *J* = 7.2 Hz, 1H), 5.36 (s, 2H) ppm.; ^13^C NMR (126 MHz, CDCl_3_) δ 168.15, 166.75, 140.67, 134.74, 133.52, 133.49, 132.11, 131.68, 131.50, 130.20, 129.52, 129.46, 128.86, 128.42, 128.00, 127.84, 127.18, 126.88, 126.32, 126.25, 125.13, 124.07, 111.44, 55.07 pm.

##### (E)-4-Phenyl-3-(((2-phenyl-3-(thiophen-2-yl)acryloyl)oxy)methyl)-1,2,5-oxadiazole 2-oxide (**9i**)

Compound **9i** was synthesized according to the general procedure for the preparation of the hybrid compounds from cinnamic acid **2i** (44 mg, 0.19 mmol, 1.2 equiv), EDCI·HCl (72 mg, 0.38 mmol, 2.3 equiv), DMAP (46 mg, 0.38 mmol, 2.3 equiv), and compound **8** (30 mg, 0.16 mmol, 1.0 equiv) in dry DMF (1.2 mL), with a total reaction time of 5 min. After purification by flash column chromatography using a PE/EtOAc 95:5 *v*/*v* eluent mixture, compound **9i** was obtained. Yield: 51%; brown oily solid; R_f_: 0.63 (PE/EtOAc 75:25); HRMS (ESI, *m*/*z*): [M+H]^+^ calculated for C_22_H_16_N_2_O_4_S: 404.0831, found: 404.0827, [M−H]^−^ calculated for C_22_H_16_N_2_O_4_S: 403.0752, found: 403.0749.; LC-MS (ESI, *m*/*z*): [M+Na]^+^ calculated for C_22_H_16_N_2_O_4_S: 427.07, found: 426.80.; ^1^H NMR (500 MHz, CDCl_3_) δ 8.03 (s, 1H), 7.59 (dd, *J* = 8.0, 1.4 Hz, 2H), 7.56–7.49 (m, 1H), 7.47–7.39 (m, 5H), 7.27–7.23 (m, 1H), 7.19–7.11 (m, 3H), 6.96–6.89 (m, 1H), 5.23 (s, 2H) ppm.; ^13^C NMR (126 MHz, CDCl_3_) δ 166.54, 157.03, 138.32, 135.77, 134.59, 134.41, 131.64, 131.42, 130.00, 129.44, 129.43, 128.87, 127.81, 127.74, 126.92, 126.19, 111.44, 54.83 ppm.

#### 3.4.6. Synthesis of the 4-(2-(Cinnamoyloxy)ethoxy)-3-(phenylsulfonyl)-1,2,5-oxadiazole 2-oxide (**11**)

To a solution of cinnamic acid **2a** (44 mg, 0.19 mmol, 1.2 equiv) in dry DMF (1.2 mL), the reagents EDCI·HCl (72 mg, 0.38 mmol, 2.3 equiv), DMAP (46 mg, 0.38 mmol, 2.3 equiv), and compound **10** (30 mg, 0.16 mmol, 1.0 equiv) were added. The reaction mixture was heated at 50 °C in a microwave oven at 50 W for 5 min. After completion of the reaction, water was added, and the mixture was extracted with EtOAc (×2). The combined organic layers were washed with brine, dried over anhydrous MgSO_4_, filtered, and concentrated under reduced pressure. The crude yellow oily product was purified by flash column chromatography with silica gel, using a PE/EtOAc 95:5 *v*/*v* solvent mixture as eluent, to isolate compound **11**. Yield: 61%; yellow solid; R_f_: 0.52 (PE/EtOAc 7:3); M.p.: 55–57 °C; ^1^H NMR (500 MHz, CDCl_3_) δ 8.07–8.05 (m, 1H), 7.75 (d, *J* = 16.0 Hz, 1H), 7.70 (t, *J* = 7.4 Hz, 1H), 7.60–7.50 (m, 4H), 7.42 (q, *J* = 3.7 Hz, 3H), 6.47 (d, *J* = 16.0 Hz, 1H), 4.72–4.70 (m, 2H), 4.63–4.61 (m, 2H) ppm.; ^13^C NMR (126 MHz, CDCl_3_) δ 166.48, 158.70, 146.00, 138.01, 135.64, 134.07, 130.70, 129.66, 129.01, 128.59, 128.22, 117.04, 69.00, 61.33 ppm [38].

### 3.5. Biological In Vitro Assays

For the in vitro assays, a 10 mM stock solution of the tested compounds was prepared in dimethyl sulfoxide (DMSO) and subsequently diluted with the appropriate buffer to achieve the desired concentrations. Each experiment was conducted in triplicate or more, with the standard deviation of absorbance remaining within 10%. The compounds were found to be stable under the in vitro experimental conditions. Their stability was studied with TLC and spectroscopically with NMR.

#### 3.5.1. In Vitro Inhibition of Linoleic Acid Lipid Peroxidation

An in vitro assay was performed following previously established protocols [89]. The compound 2,2′-Azobis(2-methylpropionamidine) dihydrochloride (AAPH) was used as a controlled source of thermally generated alkylperoxy free radicals. The formation of conjugated diene hydroperoxides through the oxidation of sodium linoleate in an aqueous dispersion at 37 °C was measured by monitoring absorbance at 234 nm. The results were compared with the reference compound, Trolox (93%) (Table 6).

#### 3.5.2. In Vitro Inhibition of Soybean Lipoxygenase

An in vitro study was conducted following a previously reported method [90]. The tested compounds were dissolved in DMSO (100 µM) and incubated at room temperature with 0.1 mL of sodium linoleate as the substrate and 0.2 mL of soybean lipoxygenase solution in Tris-HCl buffer (pH 9.0). The conversion of sodium linoleate to 13-hydroperoxylinoleic acid was monitored by measuring absorbance at 234 nm and compared to the standard inhibitor NDGA (IC_50_ = 0.45 µM) (Table 6). Different concentrations ranging from 100 µM to 1 µM were tested to determine IC_50_ values.

#### 3.5.3. In Vitro Inhibition of Ovine Cyclooxygenase-2 (COX-2)

The in vitro COX-2 inhibitory activity was assessed using arachidonic acid (AA) as a substrate and *N*,*N*,*N*′,*N*′-tetramethyl-p-phenylenediamine (TMPD) as a co-substrate in a Tris-HCl buffer (pH 8.5), following the reported method [89]. To the test tube, 10 µL of the compound dissolved in DMSO was added to a mixture containing 730 µL of Tris-HCl buffer (pH 8.5), 50 µL of heme, 10 µL of COX-2 enzyme, and 100 µL of TMPD. The absorbance was measured at 590 nm. Subsequently, 100 µL of AA was added, followed by a second 5-min incubation at 37 °C with vigorous shaking, and the absorbance was measured again at 611 nm. Different concentrations (ranging from 100 µM to 1 µM) were tested to determine IC_50_ values. A blank determination was conducted as a negative control. Results were compared against the standard inhibitor Indomethacin, as shown in Table 6.

#### 3.5.4. In Vitro Inhibition of COX-1

Cyclooxygenase (COX) activity was measured using arachidonic acid (AA) as the substrate and *N*,*N*,*N*′,*N*′-tetramethylphenylenediamine (TMPD) as the co-substrate following the reported method [90]. The reaction mixture (1 mL) consisted of 0.75 mM heme, 128 mM TMPD, 80 mM AA, and 1.5 mg of enzyme in 0.1 M Tris-HCl buffer (pH 8.5). The substrate oxidation was monitored at room temperature by recording the increase in absorbance at 590 nm. Absorbance due to the spontaneous oxidation of TMPD was subtracted from the initial oxidation rate observed in the presence of AA. The inhibitory activity of the test compounds at a concentration of 100 μM was evaluated after a 6-min pre-incubation with the enzyme in the presence of heme and TMPD. The reaction was initiated by adding AA measured again at 611 nm. SC-560 was used as the reference COX-1 inhibitor (Table 6).

#### 3.5.5. In Vitro Determination of NO Release with Griess Reagent

The compounds (from a 10 mM stock solution in DMSO, final concentration 100 μM) are added (20 μL) to each test tube. Then, 2 mL of phosphate buffer solution (pH 7.4) is added. L-cysteine, used as the thiol agent, is added to the mixture at a final concentration of 0.5 mM. The solution is incubated at 37 °C for 60 min, after which 1 mL is removed and mixed with 250 μL of Griess reagent (4 g sulfanilamide, 0.2 g *N*-(1-naphthyl)-ethylenediamine dihydrochloride, and 10 mL of H_3_PO_4_ solution, final volume adjusted to 100 mL). The mixture is allowed to stand for 10 min at room temperature, and the absorbance of the produced compound is measured at 540 nm. Sodium nitroprusside (SNP) is used as the reference compound. The results are presented as the release of NO_2_^−^ (% NO_2_^−^ (mol/mol)) induced by the compounds in the presence of L-cysteine (Table 6).

#### 3.5.6. In Vitro Inhibition of Albumin

Inhibition of albumin denaturation was measured following the reported method [66]. In a test tube, 1 mL of bovine serum albumin solution (fraction V, 1% *w*/*v* in phosphate buffer, pH 7.4) and 100 μL of the test compound solution were added sequentially. The mixture was incubated at 37 °C for 15 min, followed by heating at 60 °C for 10 min. After cooling to room temperature, the absorbance was measured at 660 nm. DMSO was used as the blank, and the results were compared to acetylsalicylic acid, which served as the reference compound (Table 6).

#### 3.5.7. MTT Assays on Cancer Cell Lines

##### Cell Culture

HeLa, MCF-7, U251, U87, MDA-MB-435, and MDA-MB-231 cancer cell lines were maintained in incubators at 37 °C with 5% CO_2_ in DMEM medium supplemented with 10% (*v*/*v*) fetal bovine serum (FBS) and antibiotics/antimycotics. All cell lines used in this study originate from the Laboratory of Biochemistry, Department of Chemistry, Aristotle University of Thessaloniki. They are low-passage, mycoplasma-free cancer cell lines that have been extensively used over the past 25 years in numerous studies across various fields of cancer research, including drug cytotoxicity testing.

##### MTT Assays

All cell lines tested were seeded in 96-well plates at a concentration of 6 × 10^3^ cells per well, except HeLa, which were seeded at 3 × 10^3^ cells per well. The day after, cells were exposed to increasing concentrations of the compounds for 48 h. Cell viability was determined using the 3-(4,5-dimethylthiazol-2-yl)-2,5-diphenyltetrazolium bromide (MTT) assay, a colorimetric assessment of cellular metabolic activity as described previously [67]. Absorbance values from treated samples were normalized to those of untreated controls, which were assigned to a 100% viability value. The half-maximal effective concentration (EC_50_), defined as the concentration of treatment resulting in a 50% reduction in cell viability relative to controls, was subsequently calculated. As a positive control, doxorubicin exhibited an IC_50_ value of approximately 500 nM following 48 h of exposure in HeLa cells as determined by the MTT assay. The results are shown in Table 7 and Table 8.

## 4. Conclusions

In this study, 28 new cinnamic acid hybrids were synthesized, 19 of which incorporated a nitric oxide donor moiety (oxime or furoxan). Computational studies confirmed the ability of the selected compounds **9a** and **9e** to interact with LOX and COX-2, in agreement with the in vitro results. In addition, in silico analyses indicated that most derivatives fulfilled drug-likeness criteria, with some exceptions in lipophilicity and blood–brain barrier permeability.

Biological evaluations revealed several potent inhibitors of lipid peroxidation (e.g., **5g**, **6g**, **9a**, **9h**, **11**), with activity comparable to Trolox, while compound **9a** displayed also a remarkable LOX inhibitory activity (IC_50_ = 3.5 μM). Furthermore, compound **9e** showed selective inhibition of COX-2, whereas **6e** exhibited significant inhibition of albumin denaturation activity, surpassing that of aspirin. Compound **9i** is a double COX-1/COX-2 inhibitor. Notably, some furoxan derivatives (**9a**, **11**) demonstrated efficient nitric oxide release, in higher proportions than the corresponding oximes. Regarding anticancer potential, compound **6i** exhibited the strongest cytotoxic activity, with EC_50_ values ranging from 36–45 μM across multiple cancer cell lines, highlighting its potential as a promising lead for further development.

Overall, these findings support that cinnamic acid-based hybrids bearing oxime and furoxan moieties represent promising multi-target agents with antioxidant, anti-inflammatory, and cytotoxic properties. These results are important to enhance our knowledge for good candidates for anticancer agents. Further investigation will follow to delineate the possible mechanism. In the literature, cinnamic acid has reported to demonstrate anticancer activity by inducing apoptosis, inhibiting cell proliferation, and inhibiting with pathways crucial for tumor growth and metastasis [11,12]. Its anticancer effects are partly due to its ability to damage DNA, block signaling pathways, and act as a potent antioxidant. However, the clinical application of cinnamic acid is currently limited due to poor water solubility and membrane permeability. Thus, we believe that the herein presented hybrids will offer some interesting ideas to skip these problems. Among them, compounds **9a**, **9e**, and **6i** emerged as the most active candidates and may serve as lead compounds for further research, whereas **9i** could be subjected to structural modifications to lead to a COX-2 inhibitor.

## Figures and Tables

**Table 1:** The chemical structures of compounds **2**, **5**, **6**, and **9**.

Compound 2, 5, 6, and 9	R_1_	R_2_
**a**	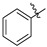	H
**b**	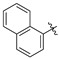	H
**c**	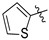	H
**d**	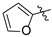	H
**e**	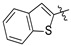	H
**f**	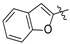	H
**g**	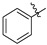	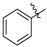
**h**	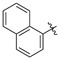	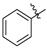
**i**	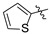	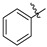

**Table 3:** Prediction of the CYP metabolism of the new hybrids using the CypRules [51] platform.

Compound	CYP2C19 Inhibition	CYP2C9 Inhibition	CYP2D6 Inhibition	CYP3A4 Inhibition
**5a**	no	no	no	yes
**5b**	no	no	no	yes
**5c**	no	no	no	yes
**5d**	no	no	no	yes
**5e**	no	no	no	yes
**5f**	no	no	no	yes
**5g**	no	yes	no	yes
**5h**	no	yes	no	yes
**5i**	no	no	no	yes
**6a**	no	no	no	yes
**6b**	no	no	no	yes
**6c**	no	no	no	yes
**6d**	no	no	no	yes
**6e**	no	no	no	yes
**6f**	no	no	no	yes
**6g**	no	yes	no	yes
**6h**	no	yes	no	yes
**6i**	no	no	no	yes
**9a**	yes	yes	no	yes
**9b**	yes	yes	no	yes
**9c**	yes	yes	no	yes
**9d**	yes	yes	no	yes
**9e**	yes	yes	no	yes
**9f**	yes	yes	no	yes
**9g**	yes	yes	no	yes
**9h**	yes	yes	no	yes
**9i**	yes	yes	no	yes
**11**	yes	yes	no	yes

**Table 4:** Toxicity assessment of compounds **5a**–**i**, **6a**–**i**, **9a**–**i**, and **11** using preADMET platform [46].

Compound	hERG Inhibition	Carcinogenicity in Mouse	Carcinogenicity in Rat	Ames Test	TA100_NA	TA100_10RLI	TA1535_NA	TA1535_10RLI
**5a**	Medium risk	negative	negative	mutagen	negative	positive	negative	negative
**5b**	Medium risk	negative	negative	mutagen	negative	positive	negative	negative
**5c**	Medium risk	negative	negative	mutagen	negative	positive	negative	positive
**5d**	Medium risk	negative	negative	mutagen	positive	positive	negative	positive
**5e**	Medium risk	negative	negative	mutagen	negative	positive	negative	negative
**5f**	Medium risk	negative	negative	mutagen	positive	positive	negative	negative
**5g**	Low risk	negative	negative	Non-mutagen	negative	negative	negative	negative
**5h**	Low risk	negative	negative	mutagen	negative	negative	negative	negative
**5i**	Medium risk	negative	negative	mutagen	negative	negative	negative	positive
**6a**	Medium risk	positive	negative	mutagen	negative	positive	negative	negative
**6b**	high risk	negative	negative	mutagen	negative	positive	negative	negative
**6c**	Medium risk	negative	negative	mutagen	negative	positive	negative	positive
**6d**	Medium risk	negative	negative	mutagen	positive	positive	negative	positive
**6e**	Medium risk	negative	negative	mutagen	negative	positive	negative	negative
**6f**	Medium risk	negative	negative	mutagen	positive	positive	negative	negative
**6g**	ambiguous	negative	negative	mutagen	negative	positive	negative	negative
**6h**	ambiguous	negative	negative	mutagen	negative	positive	negative	negative
**6i**	high risk	negative	negative	mutagen	negative	positive	negative	positive
**9a**	low risk	negative	positive	mutagen	positive	positive	positive	positive
**9b**	low risk	negative	positive	mutagen	negative	positive	positive	negative
**9c**	Medium risk	negative	positive	mutagen	positive	positive	positive	positive
**9d**	Medium risk	negative	positive	mutagen	positive	positive	positive	positive
**9e**	low risk	negative	positive	mutagen	positive	positive	positive	negative
**9f**	low risk	negative	negative	mutagen	positive	positive	positive	negative
**9g**	low risk	negative	positive	mutagen	positive	positive	positive	positive
**9h**	low risk	negative	negative	mutagen	negative	negative	negative	negative
**9i**	low risk	negative	positive	mutagen	negative	positive	positive	positive
**11**	low risk	negative	negative	mutagen	positive	positive	positive	positive

**Table 5:** %ABS values of the new molecules **5a**–**i**, **6a**–**i**, **9a**–**i**, and **11.**.

Compound	%ABS	Compound	%ABS
**5a**	84	**6f**	74.11
**5b**	84	**6g**	78.64
**5c**	84	**6h**	78.64
**5d**	79.46	**6i**	78.64
**5e**	84	**9a**	82.16
**5f**	79.46	**9b**	82.16
**5g**	84	**9c**	82.16
**5h**	84	**9d**	77.63
**5i**	84	**9e**	82.16
**6a**	78.64	**9f**	77.63
**6b**	78.64	**9g**	82.16
**6c**	78.64	**9h**	82.16
**6d**	74.11	**9i**	82.16
**6e**	78.64	**11**	67.20

**Table 6:** Representation of the biological results of the new compounds **5a**–**i**, **6a**–**i**, **9a**–**i**, and **11**.

Compound	AAPH Inh. at 100 μΜ	LOX Inh. IC_50_ (μΜ)	COX-2 Inh. IC_50_ (μΜ)	COX-1 at 100 μM	%NO_2_^−^ Release mol/mol at 100 μΜ	Albumin
**5a**	31.9%	no	nt	nt	nt	nt
**5b**	no	57.5	nt	nt	nt	nt
**5c**	29%	no	nt	nt	nt	nt
**5d**	no	no	nt	nt	nt	nt
**5e**	no	no	nt	nt	nt	nt
**5f**	39%	80	nt	nt	nt	nt
**5g**	94.7%	no	nt	nt	nt	nt
**5h**	75.8%	33	nt	nt	nt	nt
**5i**	77%	no	nt	nt	nt	nt
**6a**	50.2%	50	100	nt	no	no
**6b**	94%	60	no	nt	1.1%	no
**6c**	4%	10	35	no	4%	no
**6d**	54%	no	no	nt	0.2%	10%
**6e**	no	no	no	nt	1.1%	63%
**6f**	no	61	no	nt	0.3%	no
**6g**	89%	44	nt	nt	no	no
**6h**	97.7%	62.5	nt	nt	no	no
**6i**	28%	60	80	16.7%	no	no
**9a**	91.6%	3.5	no	nt	20%	no
**9b**	84%	no	no	nt	1.2%	no
**9c**	73.3%	no	no	nt	1.2%	no
**9d**	78%	63	no	nt	no	no
**9e**	67%	65	8.25	no	no	no
**9f**	97%	70	no	nt	no	no
**9g**	90%	55.5	no	nt	3%	no
**9h**	94.5%	10	no	nt	no	no
**9i**	no	no	70	49%	1%	no
**11**	90%	no	no	nt	11%	no
**Cinnamic acid**	78%	56	-	-	-	-
**Trolox**	93%	-	-	-	-	-
**NDGA**	-	0.45	-	-	-	-
**Indomethacin**	-	-	2.12	-	-	-
**SC-560**	-	-	-	100%	-	-
**SNP**	-	-	-	-	53%	-
**Acetylsalicylic acid**						31%

no: no activity under the reported experimental conditions; nt: not tested.

**Table 7:** Half maximal effective concentration (EC_50_ values) of **6c**, **6i**, **9e**, **9g**, and **9i** in HeLa and MCF-7 cancer cell lines.

Compound	HeLa EC_50_ (μΜ)	MCF-7 EC_50_ (μΜ)
**6c**	83.04	>100
**6i**	36.69	38.34
**9e**	55.46	58.53
**9g**	46.59	>100
**9i**	55.96	>100

**Table 8:** Half maximal effective concentration (EC_50_ values) of compound **6i** in HeLa, MCF-7, U251, U87, MDA-MB-435, and MDA-MB-231 cancer cell lines.

Cell Line	Compound 6i EC_50_ (μM)
**HeLa**	36.69
**MCF-7**	38.34
**U251**	40.94
**U87**	45.44
**MDA-MB-231**	42.82
**MDA-MB-435**	52.89

## Data Availability

The synthesized compounds are available from the laboratory of Pharmaceutical chemistry, under the responsibility of Hadjipavlou-Litina.

## Supplementary Materials

The following supporting information can be downloaded at: https://www.mdpi.com/article/10.3390/molecules30234582/s1.
