# Supplementary material for: Design, Synthesis, Biological Evaluation, and In Silico Studies of Novel Multitarget Cinnamic Acid Hybrids
Source: Molecules. 2025 Nov 28;30(23):4582. doi: 10.3390/molecules30234582 (PMC12693419; doi:10.3390/molecules30234582)
Supplement: Supplementary file 1 [file molecules-30-04582-s001.zip › molecules-3969524-supplementary.pdf]

Design, Synthesis, Biological Evaluation, and In Silico Studies of Novel Multitarget Cinnamic Acid Hybrids †

Ioanna-Chrysoula Tsopka [1], Eleni Pontiki [1], Ioanna Sigala [2], Eleni Nikolakaki [2], Kyriakos C. Prousis [3] and Dimitra Hadjipavlou-Litina [1,*]

[1] Department of Pharmaceutical Chemistry, School of Pharmacy, Faculty of Health Sciences, Aristotle University of Thessaloniki, 54124 Thessaloniki, Greece; joannatsopka@gmail.com (I.-C.T.); epontiki@pharm.auth.gr (E.P.)

[2] Laboratory of Biochemistry, Department of Chemistry, Aristotle University of Thessaloniki, 54124 Thessaloniki, Greece; isigala@chem.auth.gr (I.S.); nikol@chem.auth.gr (E.N.)

[3] Institute of Chemical Biology, National Hellenic Research Foundation, 48 Vassileos Constantinou Avenue, 11635 Athens, Greece; kyrprous@eie.gr

* Correspondence: hadjipav@pharm.auth.gr; Tel.: +30-231-099-7627; Fax: +30-231-099-7679

† In memory of professor Ioanna Andreadou.

Contents

^{1}H NMR and LCMS spectrums of compound **5a** 3

^{1}H NMR, ^{13}C NMR and LCMS spectrums of compound **5b** 5

^{1}H NMR, ^{13}C NMR and LCMS spectrums of compound **5c** 8

^{1}H NMR, ^{13}C NMR and LCMS spectrums of compound **5d** 11

^{1}H NMR, ^{13}C NMR and LCMS spectrums of compound **5e** 14

^{1}H NMR, ^{13}C NMR and LCMS spectrums of compound **5f** 17

^{1}H NMR, ^{13}C NMR and LCMS spectrums of compound **5g** 20

^{1}H NMR, ^{13}C NMR and LCMS spectrums of compound **5h** 23

^{1}H NMR, ^{13}C NMR and LCMS spectrums of compound **5i** 26

^{1}H NMR, ^{13}C NMR and LCMS spectrums of compound **6a** 29

^{1}H NMR, ^{13}C NMR, HRMS and LCMS spectrums of compound **6b** 32

^{1}H NMR, ^{13}C NMR, HRMS and LCMS spectrums of compound **6c** 37
^{1}H NMR, ^{13}C NMR, HRMS and LCMS spectrums of compound **6d** 42
^{1}H NMR, ^{13}C NMR, HRMS and LCMS spectrums of compound **6e** 47
^{1}H NMR, ^{13}C NMR, HRMS and LCMS spectrums of compound **6f** 52
^{1}H NMR, ^{13}C NMR, HRMS and LCMS spectrums of compound **6g** 57
^{1}H NMR, ^{13}C NMR, HRMS and LCMS spectrums of compound **6h** 62
^{1}H NMR, ^{13}C NMR, HRMS and LCMS spectrums of compound **6i** 67
^{1}H NMR, ^{13}C NMR and LCMS spectrums of compound **9a** 72
^{1}H NMR, ^{13}C NMR, HRMS and LCMS spectrums of compound **9b** 75
^{1}H NMR, ^{13}C NMR, HRMS and LCMS spectrums of compound **9c** 79
^{1}H NMR, ^{13}C NMR, HRMS and LCMS spectrums of compound **9d** 83
^{1}H NMR, ^{13}C NMR, HRMS and LCMS spectrums of compound **9e** 86
^{1}H NMR, ^{13}C NMR, HRMS and LCMS spectrums of compound **9f** 90
^{1}H NMR, ^{13}C NMR, HRMS and LCMS spectrums of compound **9g** 93
^{1}H NMR, ^{13}C NMR, HRMS and LCMS spectrums of compound **9h** 97
^{1}H NMR, ^{13}C NMR, HRMS and LCMS spectrums of compound **9i** 101
^{1}H NMR, ^{13}C NMR spectrums of compound **11** 105

^{1}H NMR and LCMS spectrums of compound **5a**

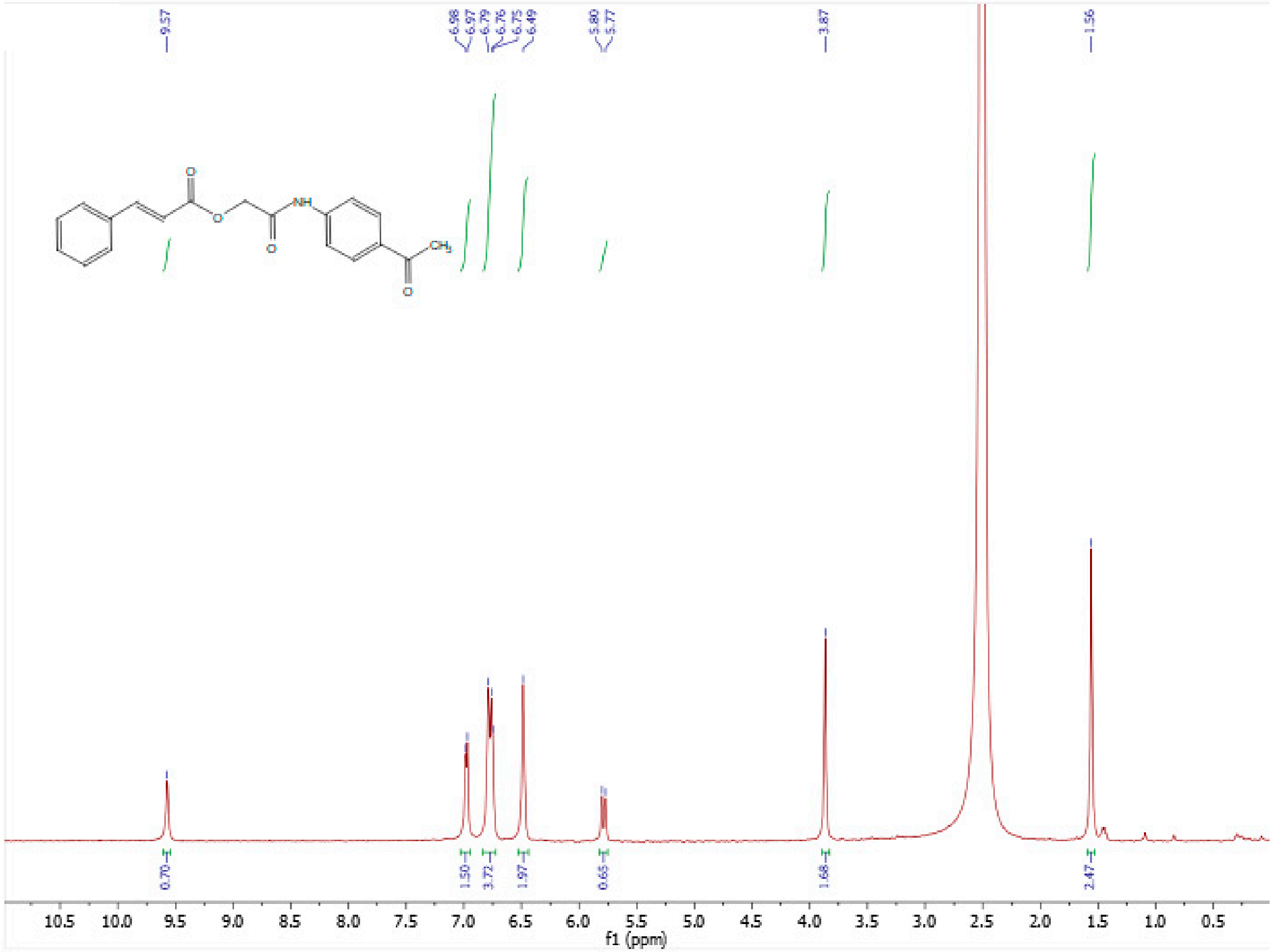

Figure S1: ^{1}H NMR spectrum of compound **5a**

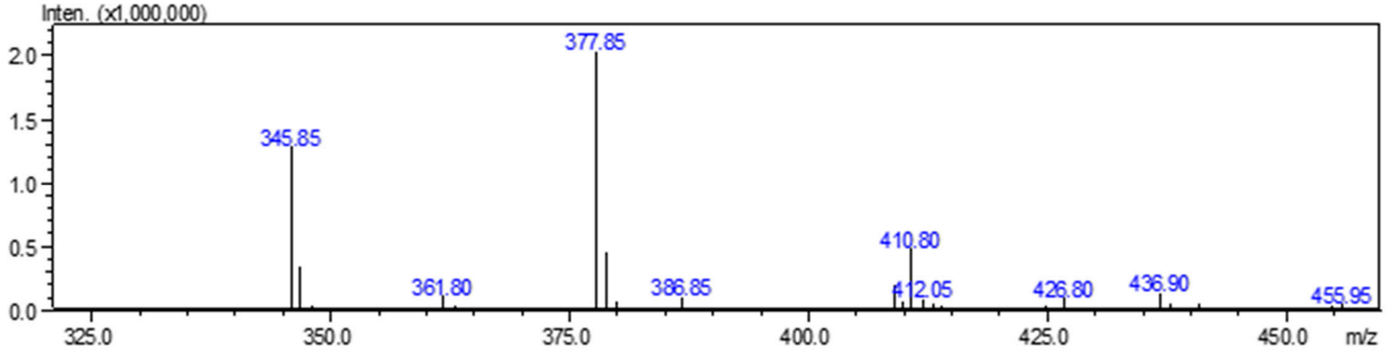

Figure S2: *Positive* LC-MS spectrum of compound **5a**

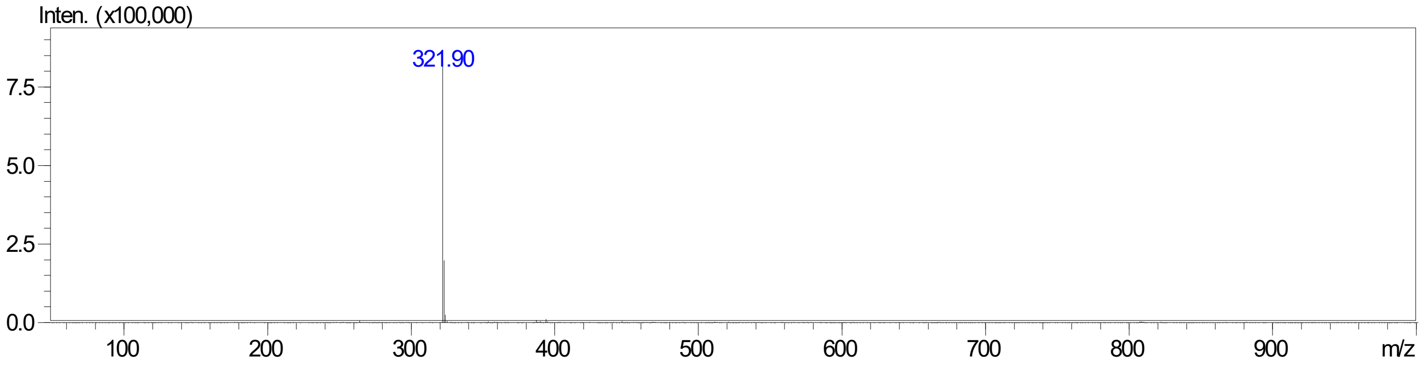

Figure S3: *Negative* LC-MS spectrum of compound **5a**

^{1}H NMR, ^{13}C NMR and LCMS spectrums of compound **5b**

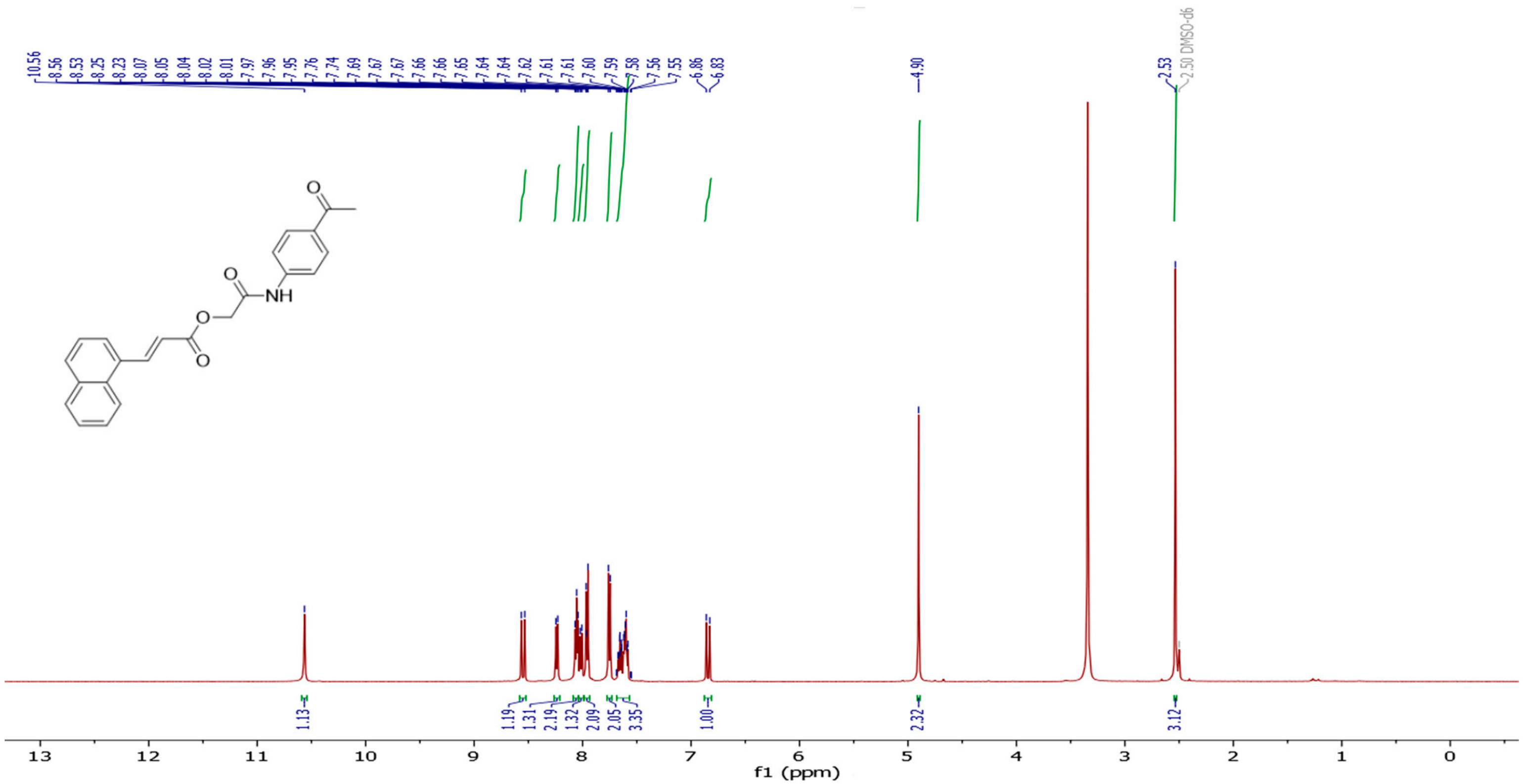

Figure S4: ^{1}H NMR spectrum of compound **5b**

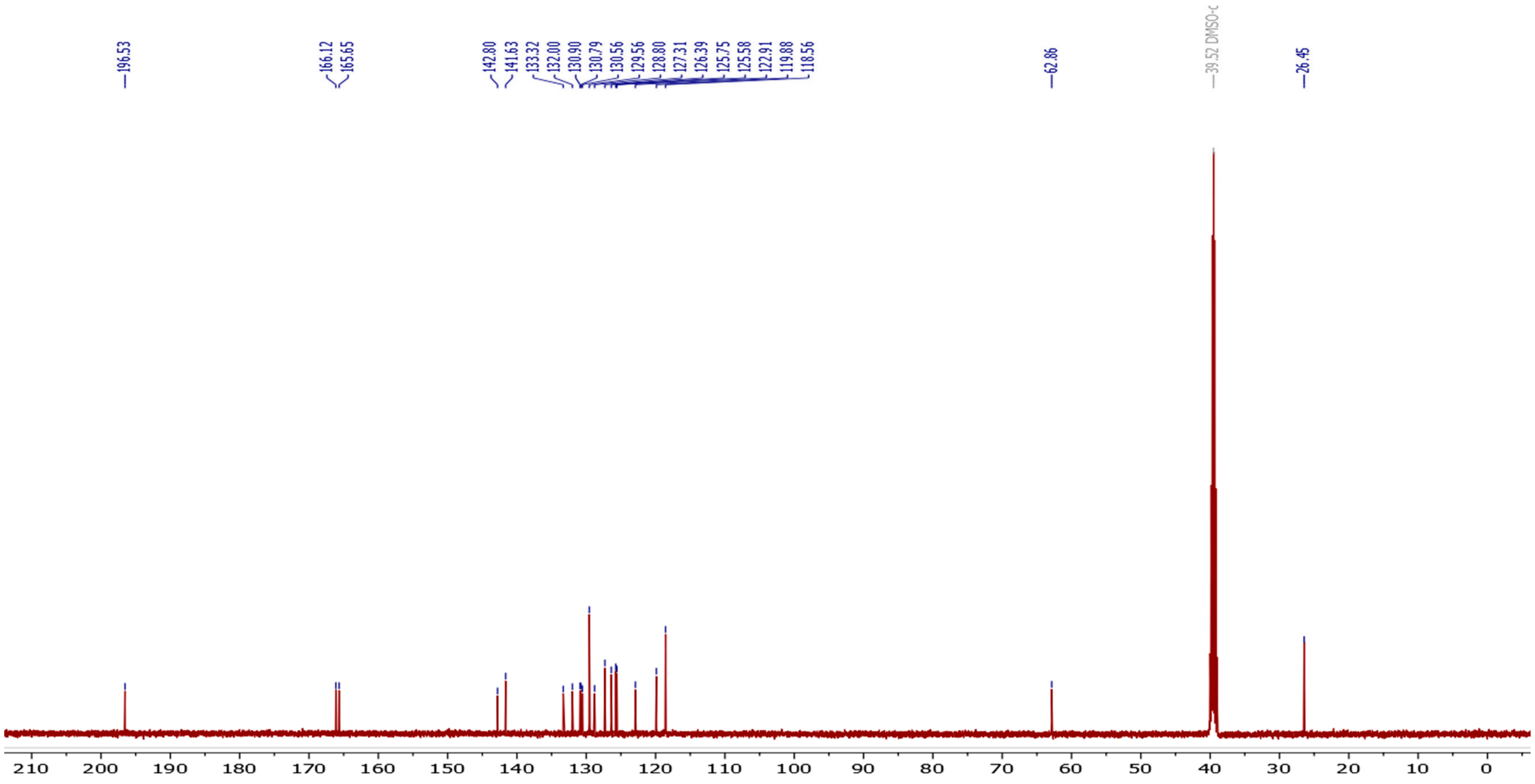

Figure S5: ^{13}C NMR spectrum of compound **5b**

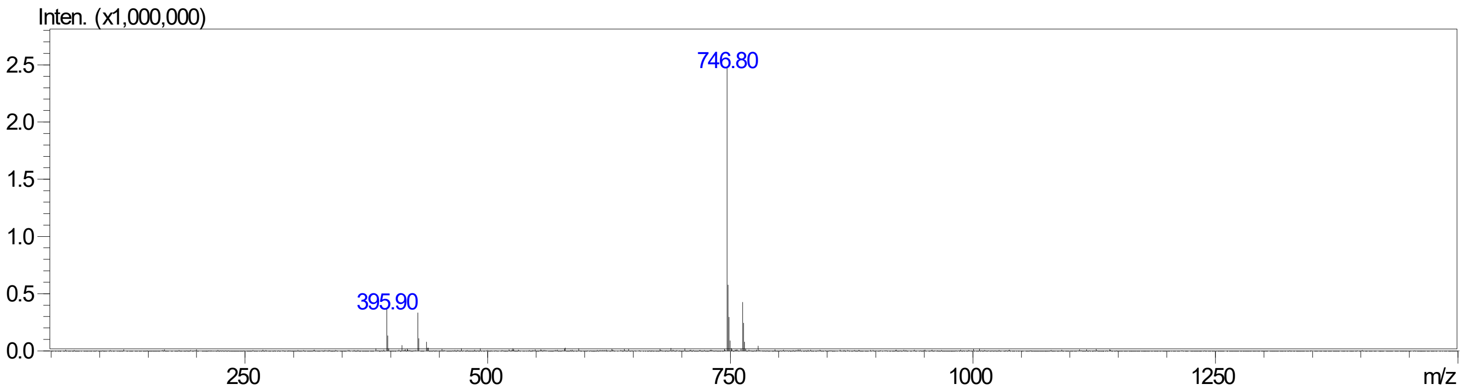

Figure S6: *Positive* LC-MS spectrum of compound **5b**

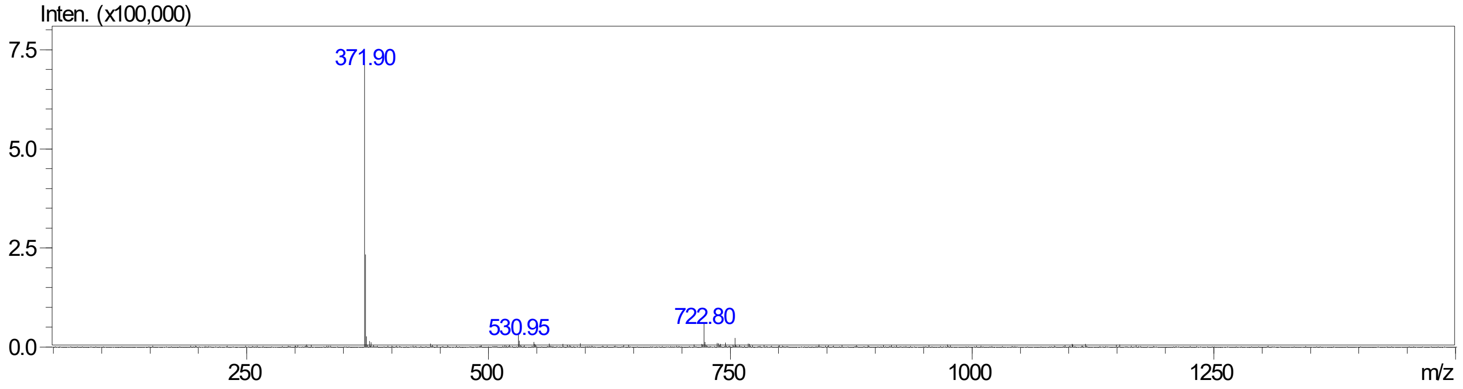

Figure S7: *Negative* LC-MS spectrum of compound **5b**

^{1}H NMR, ^{13}C NMR and LCMS spectrums of compound **5c**

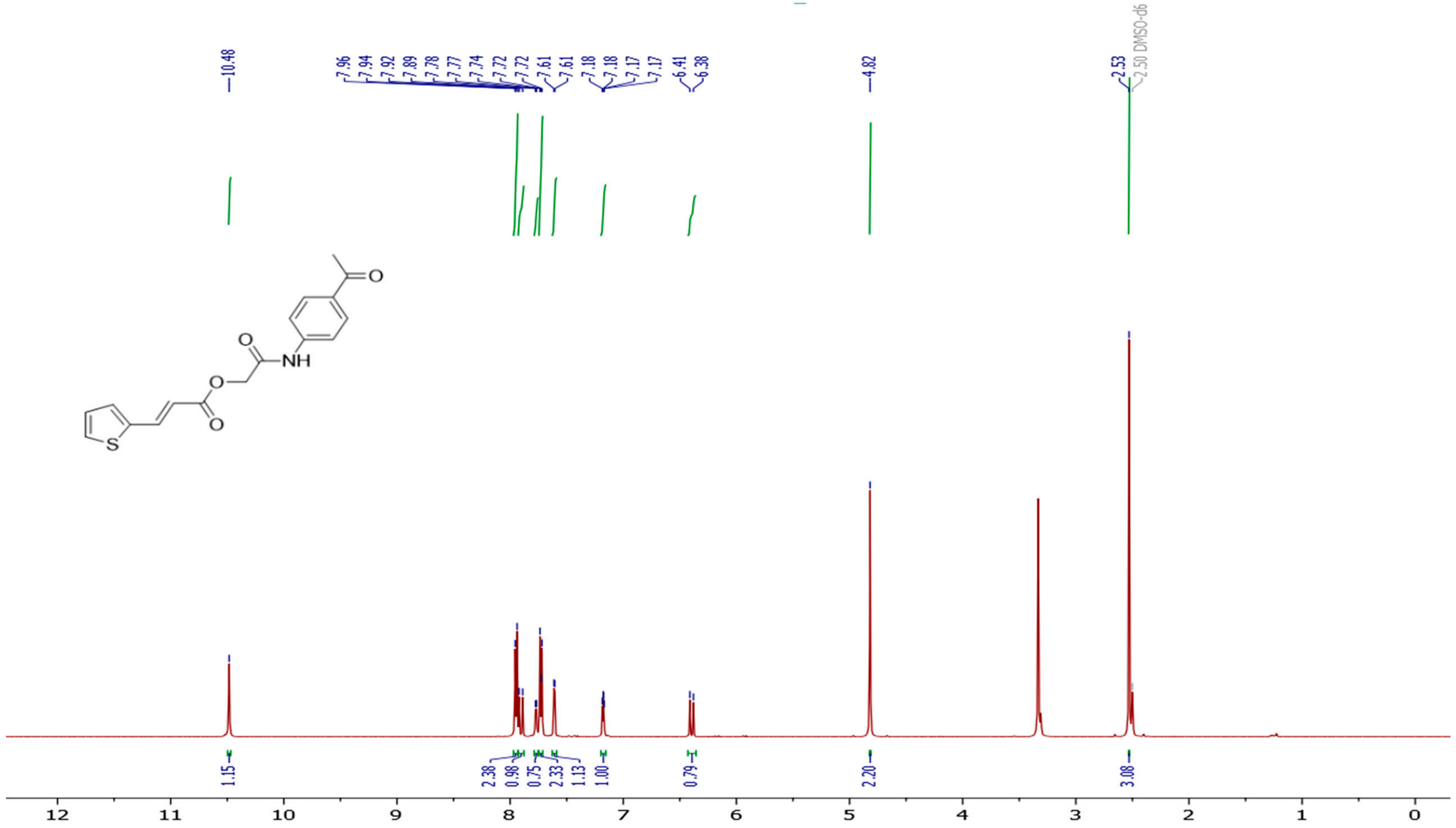

Figure S8: ^{1}H NMR spectrum of compound **5c**

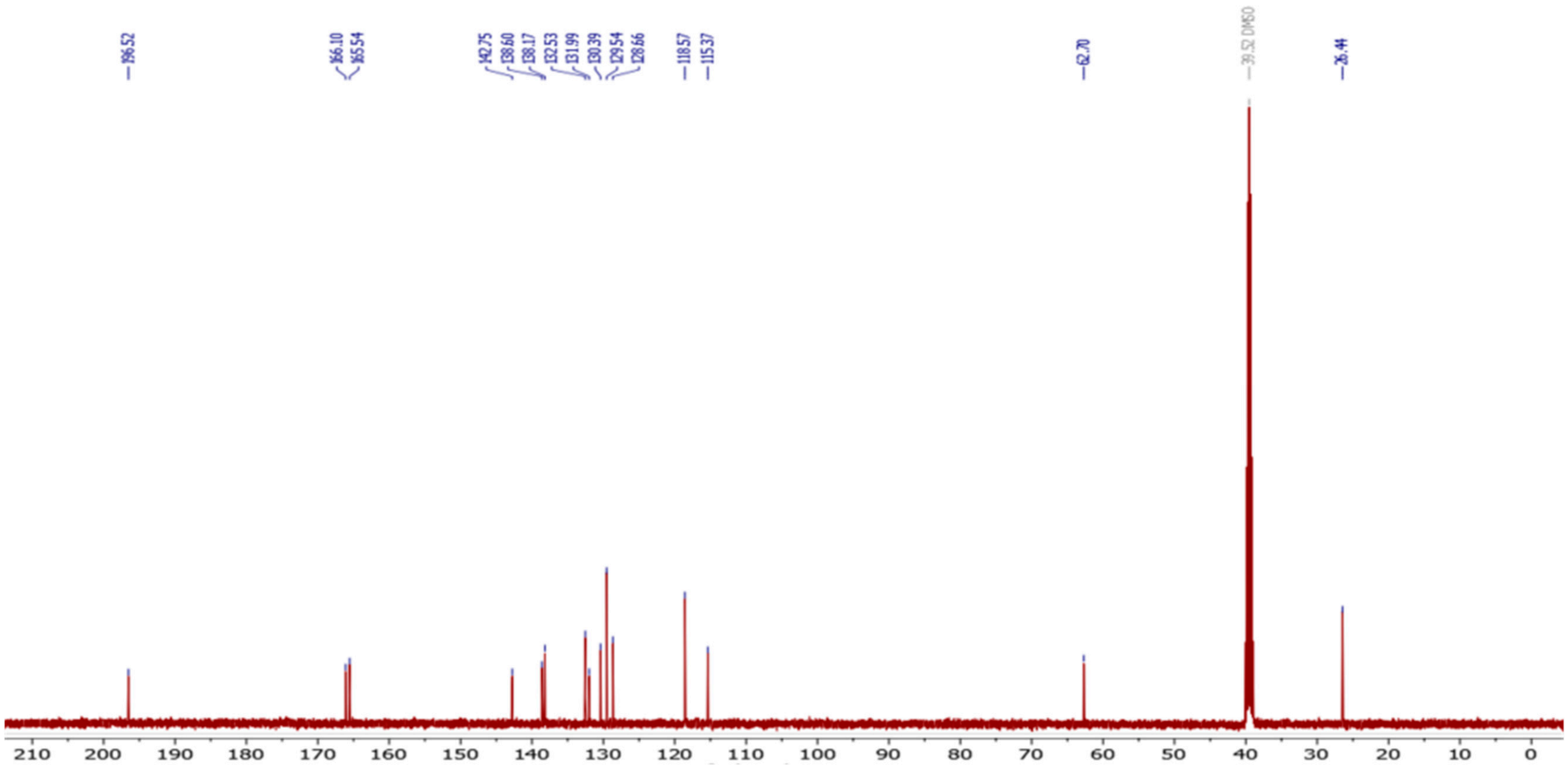

Figure S9: ^{13}C NMR spectrum of compound **5c**

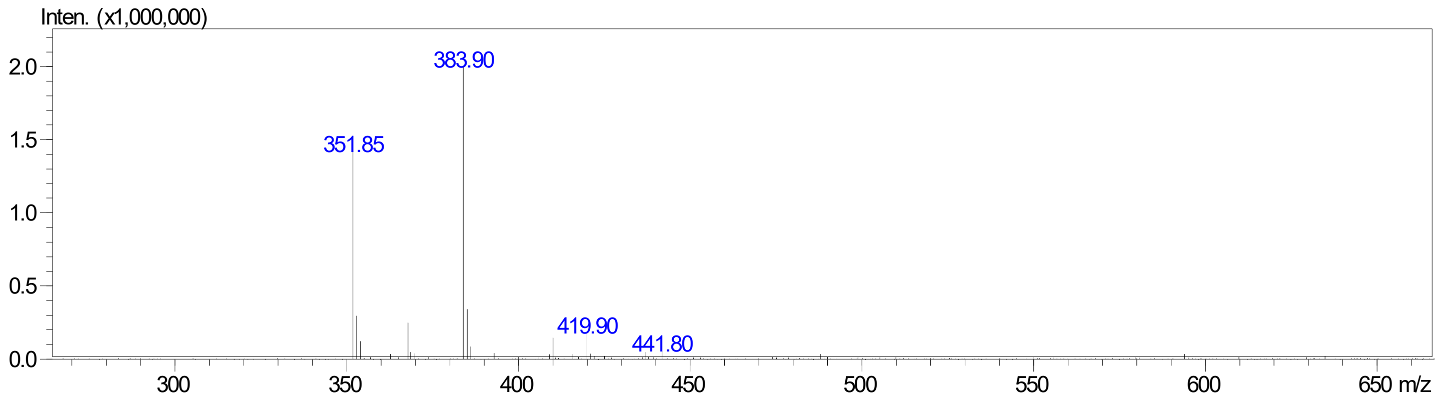

Figure S10: *Positive* LC-MS spectrum of compound **5c**

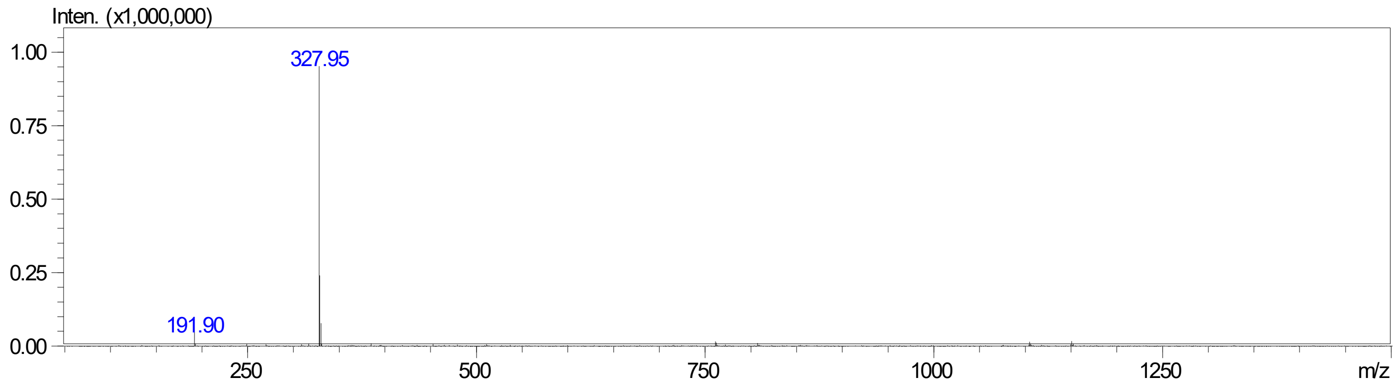

Figure S11: *Negative* LC-MS spectrum of compound **5c**

^1H NMR, ^{13}C NMR and LCMS spectrums of compound **5d**

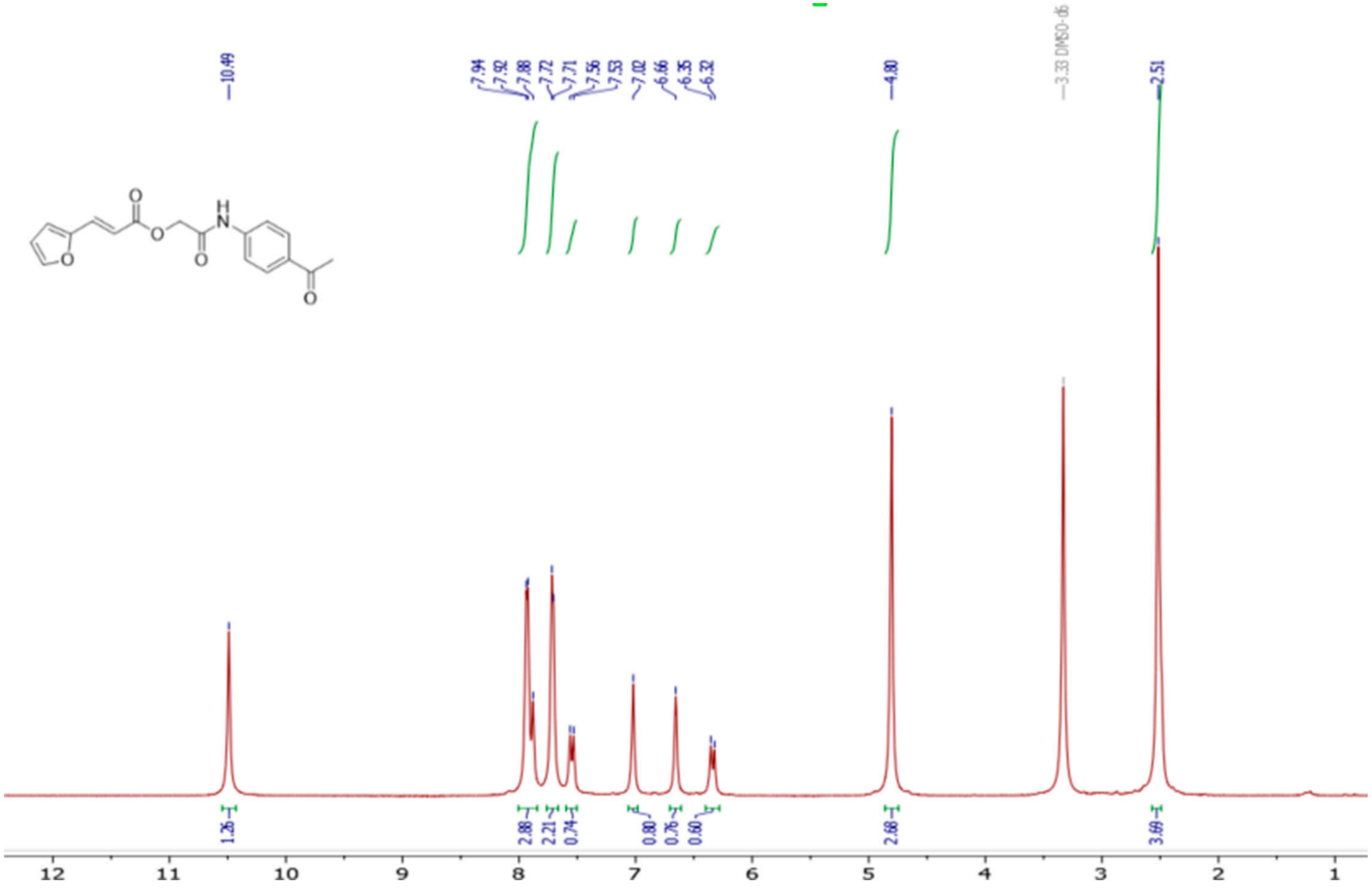

Figure S12: ^1H NMR spectrum of compound **5d**

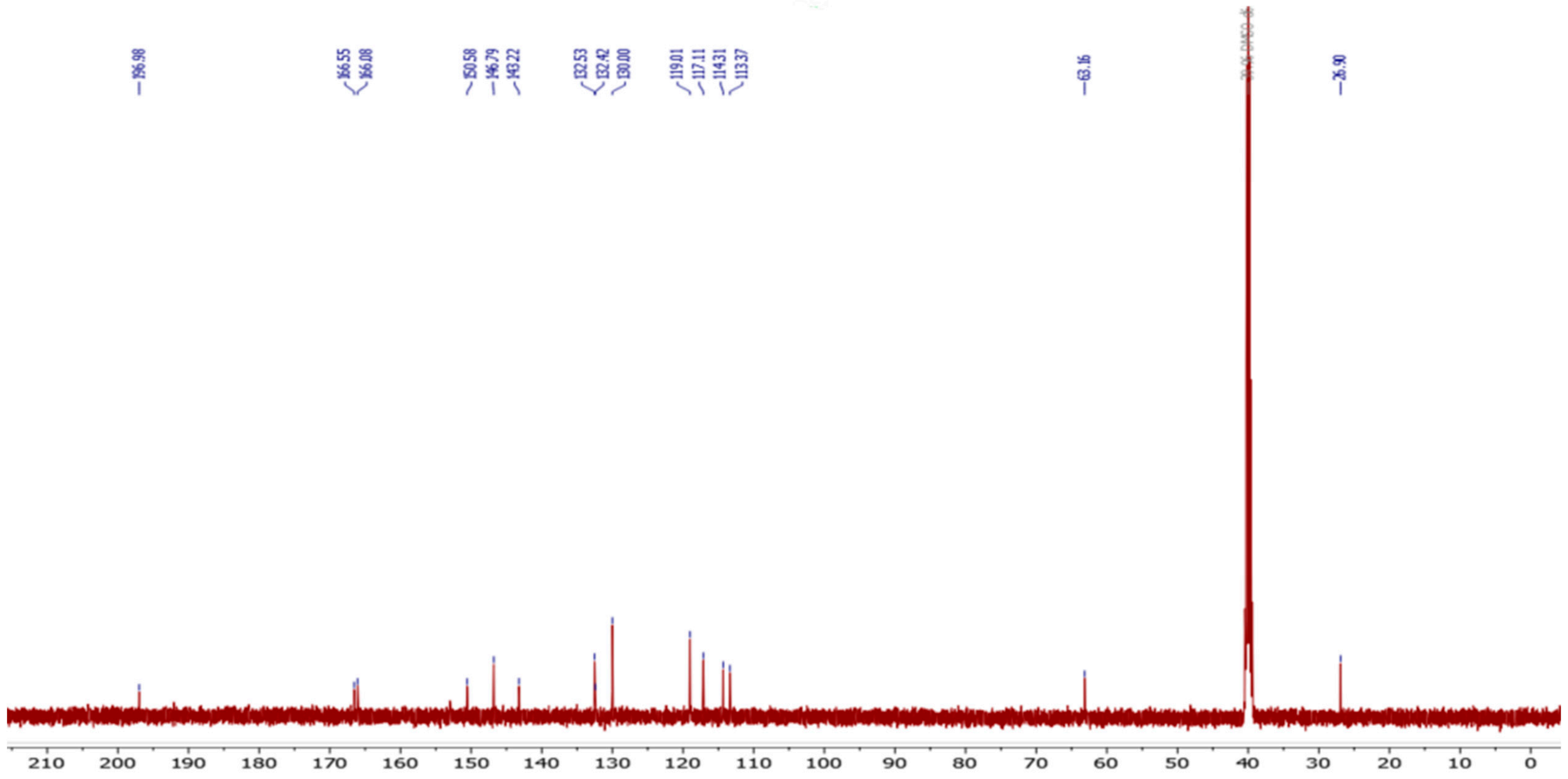

Figure S13: ^{13}C NMR spectrum of compound **5d**

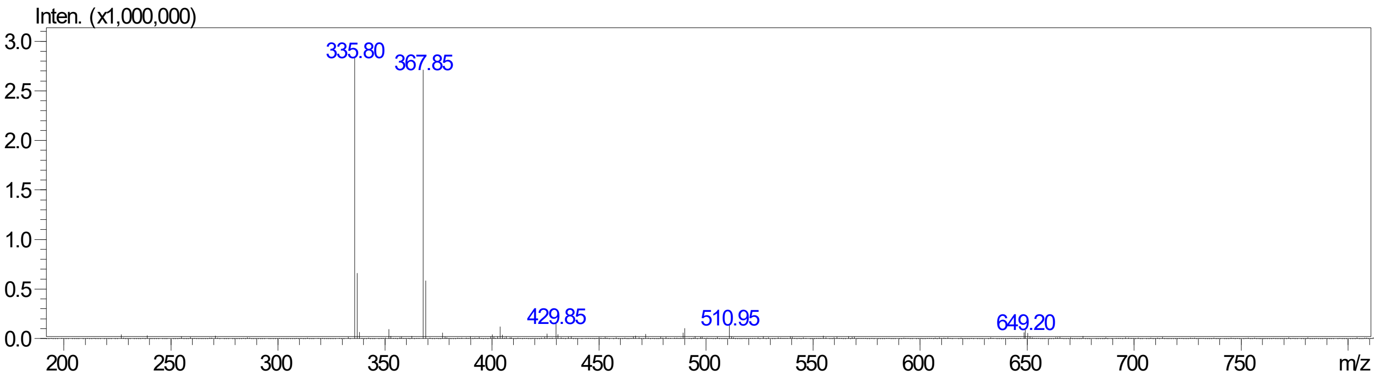

Figure S14: *Positive* LC-MS spectrum of compound **5d**

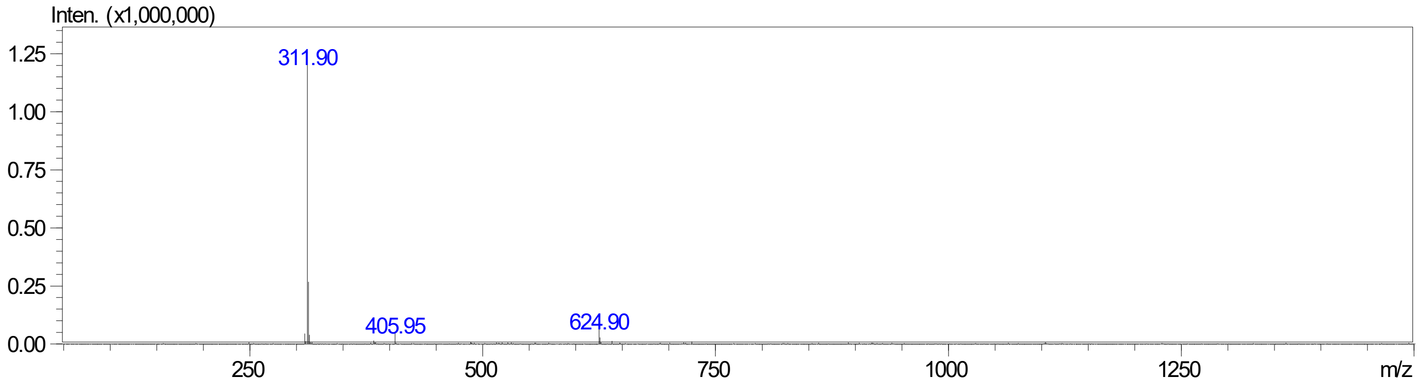

Figure S15: *Negative* LC-MS spectrum of compound **5d**

^{1}H NMR, ^{13}C NMR and LCMS spectrums of compound **5e**

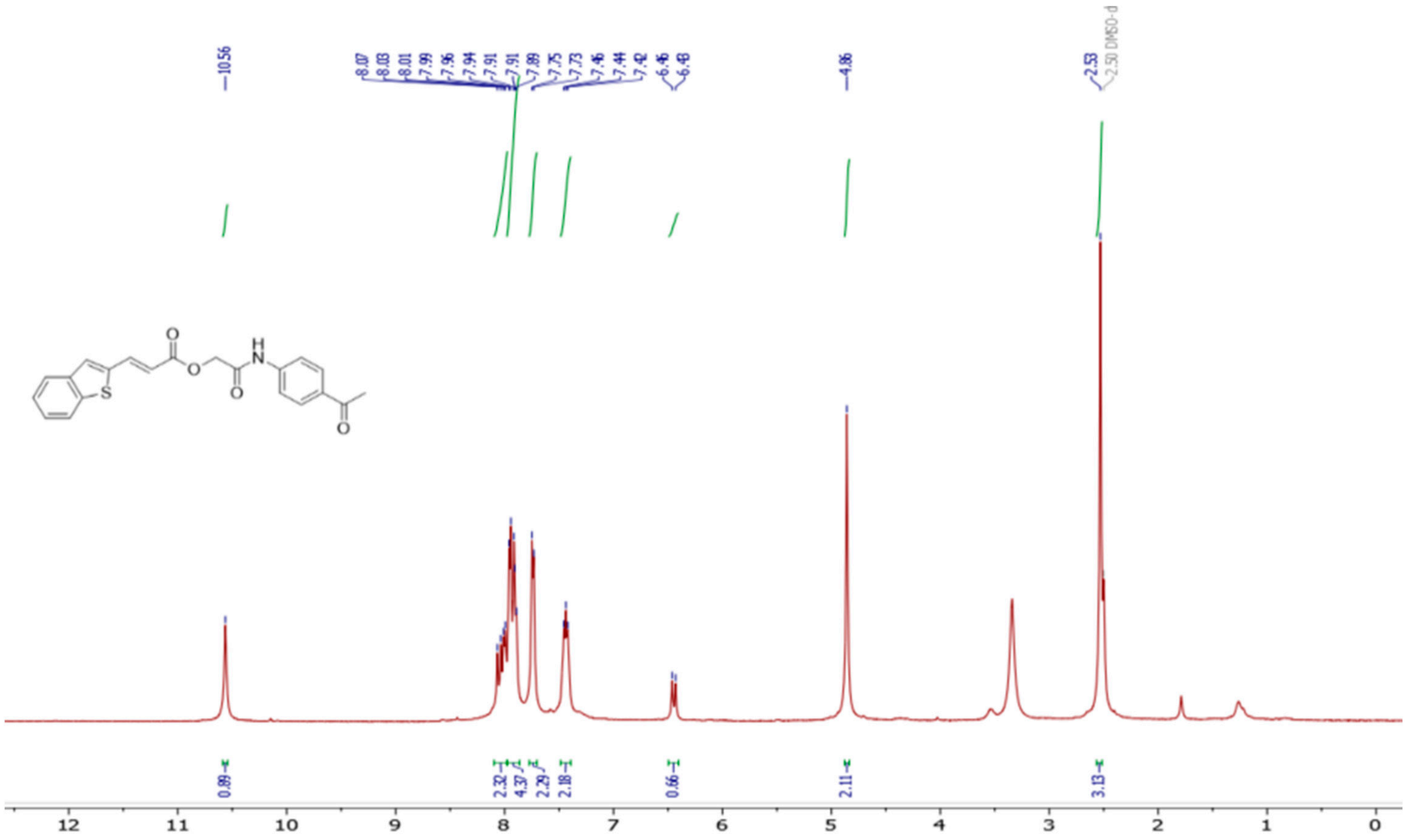

Figure S16: ^{1}H NMR spectrum of compound **5e**

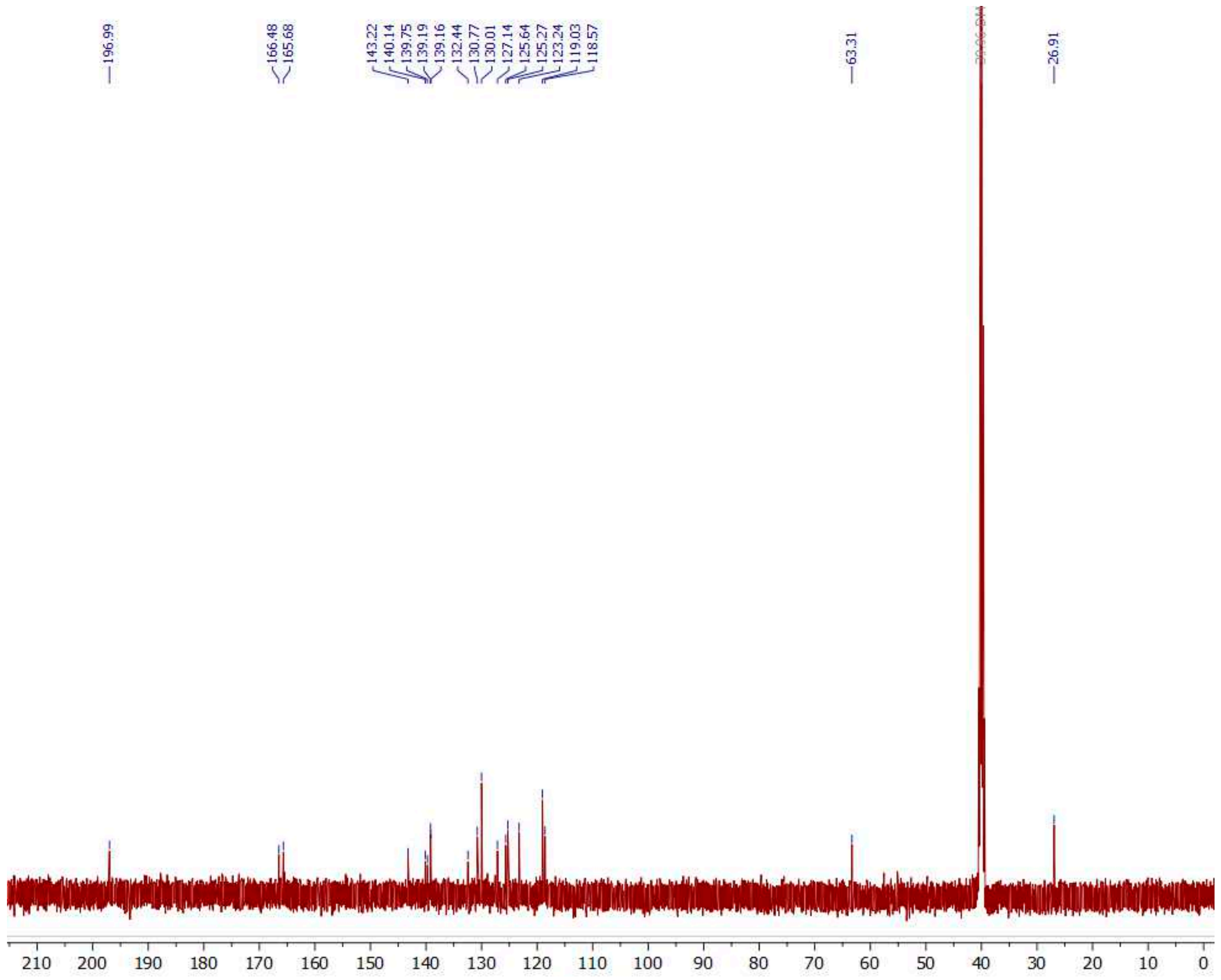

Figure S17: ^{13}C NMR spectrum of compound **5e**

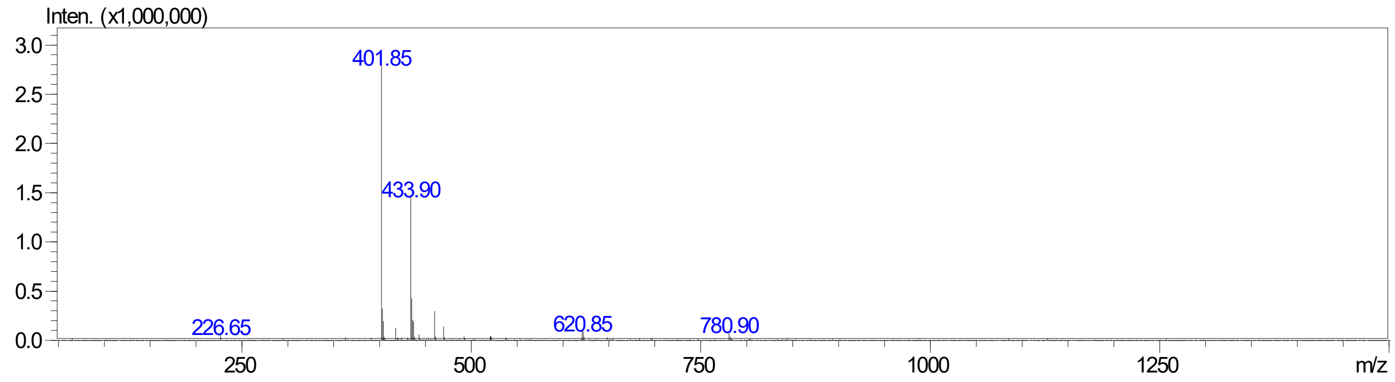

Figure S18: *Positive* LC-MS spectrum of compound **5e**

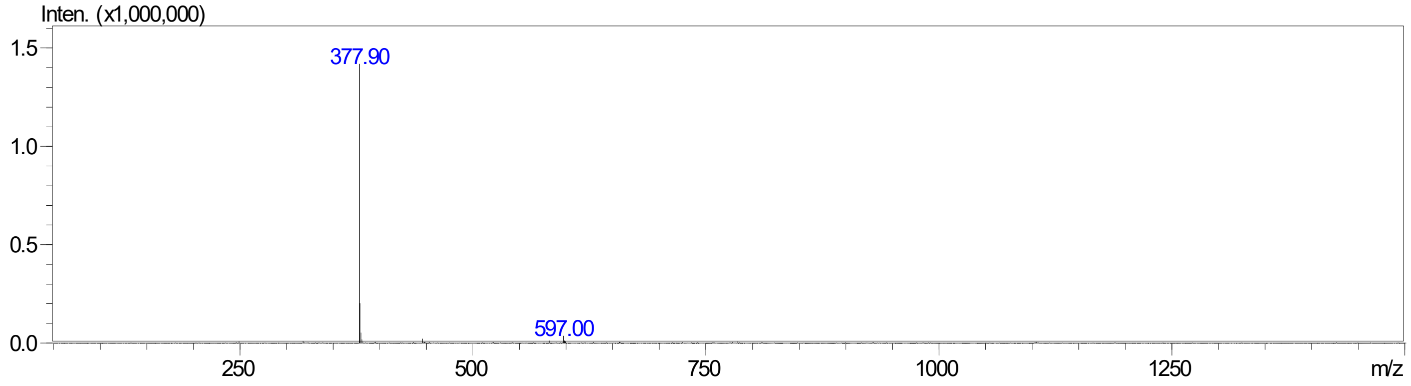

Figure S19: *Negative* LC-MS spectrum of compound **5e**

^{1}H NMR, ^{13}C NMR and LCMS spectrums of compound **5f**

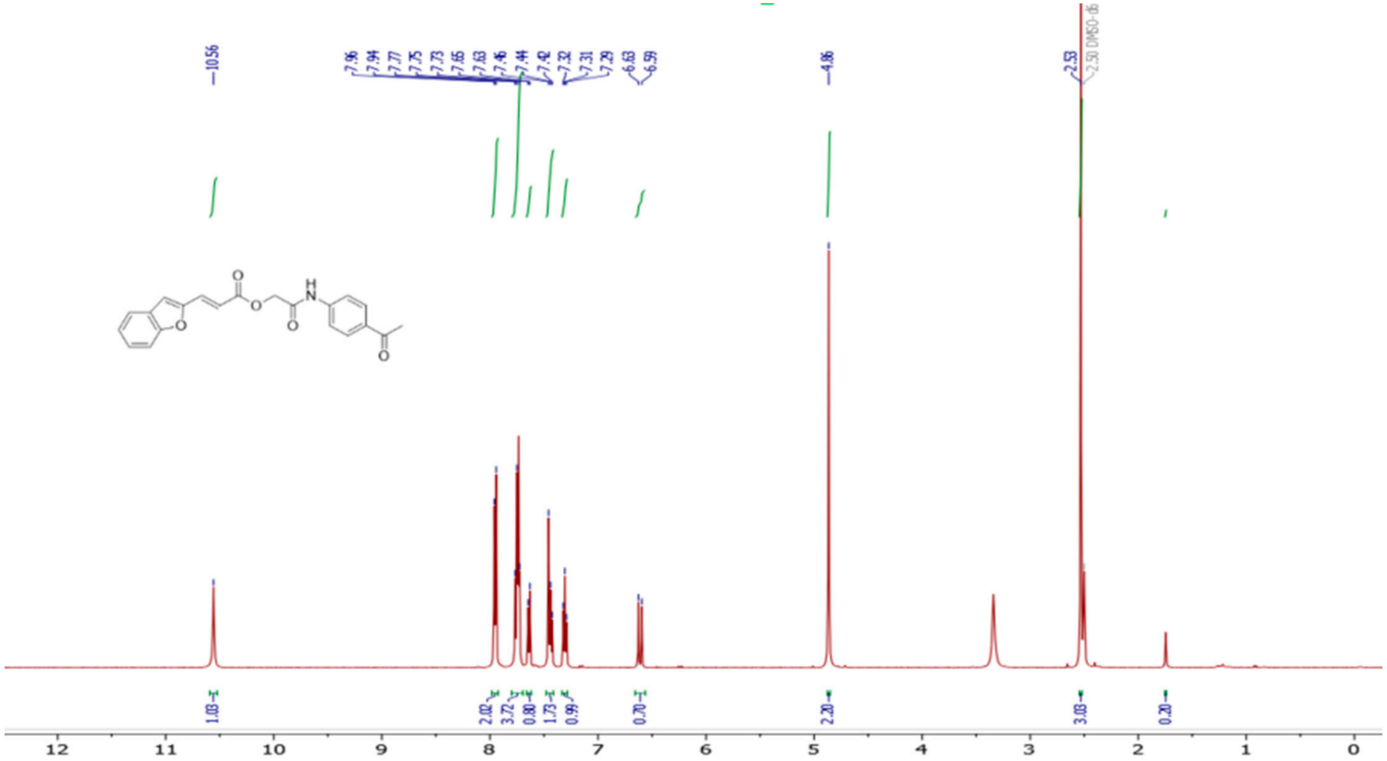

Figure S20: ^{1}H NMR spectrum of compound **5f**

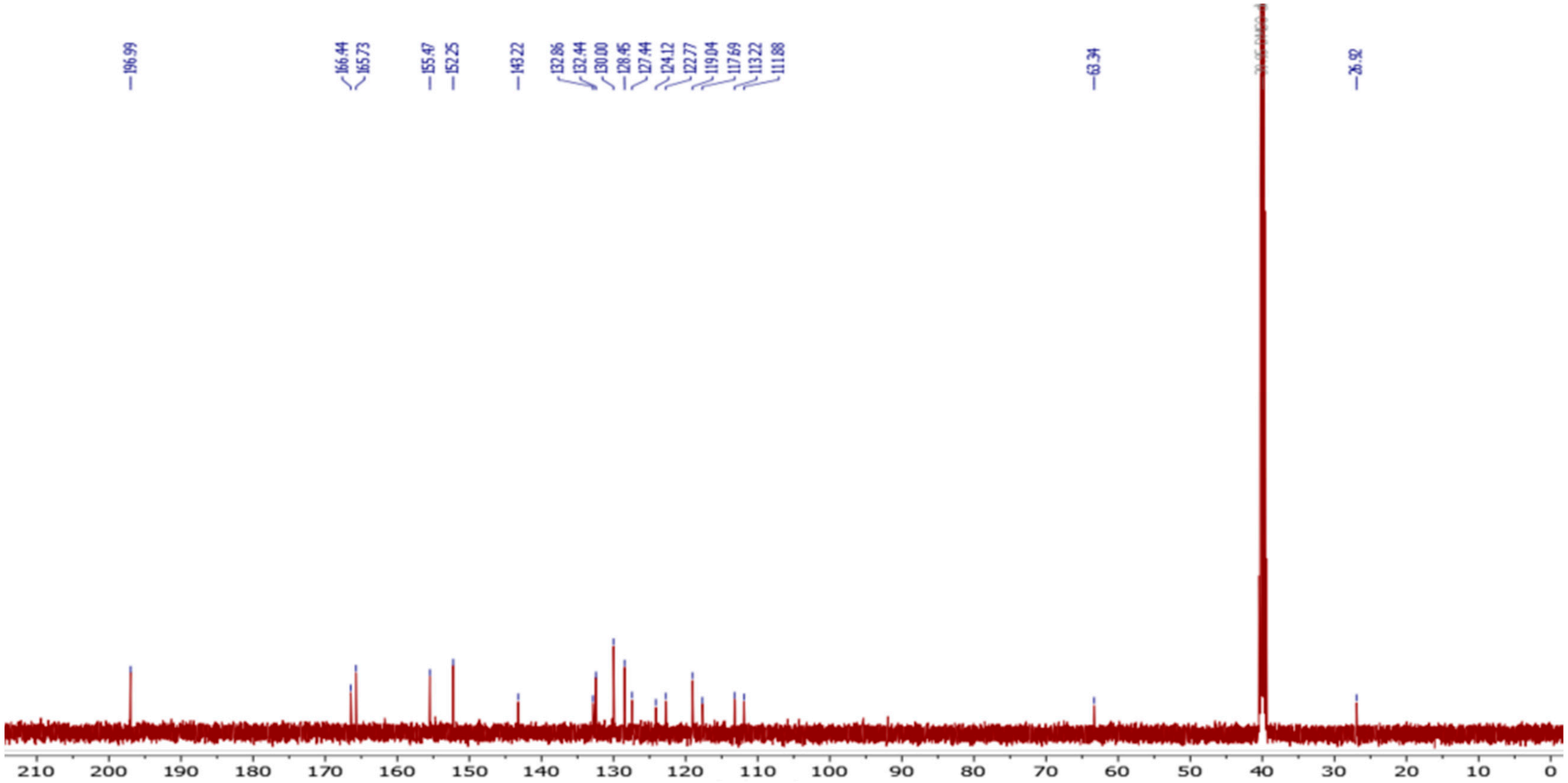

Figure S21: ^{13}C NMR spectrum of compound **5f**

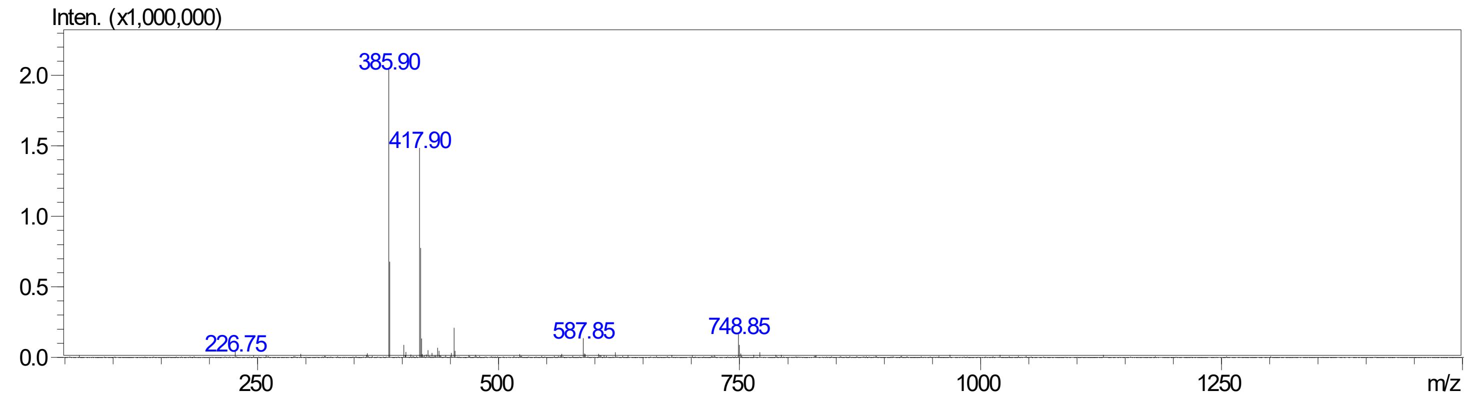

Figure S22: *Positive* LC-MS spectrum of compound **5f**

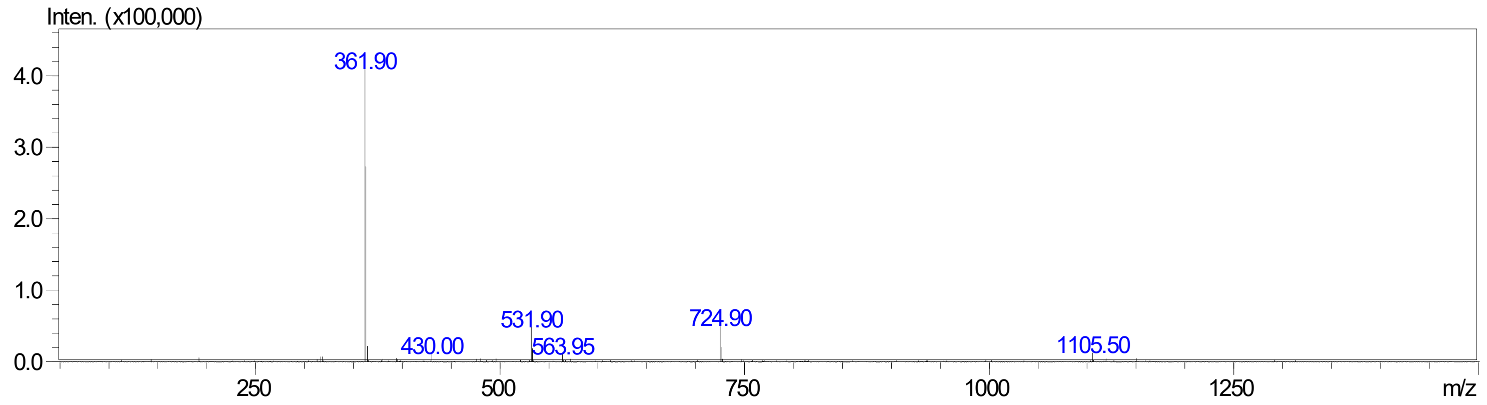

Figure S23: *Negative* LC-MS spectrum of compound **5f**

^{1}H NMR, ^{13}C NMR and LCMS spectrums of compound **5g**

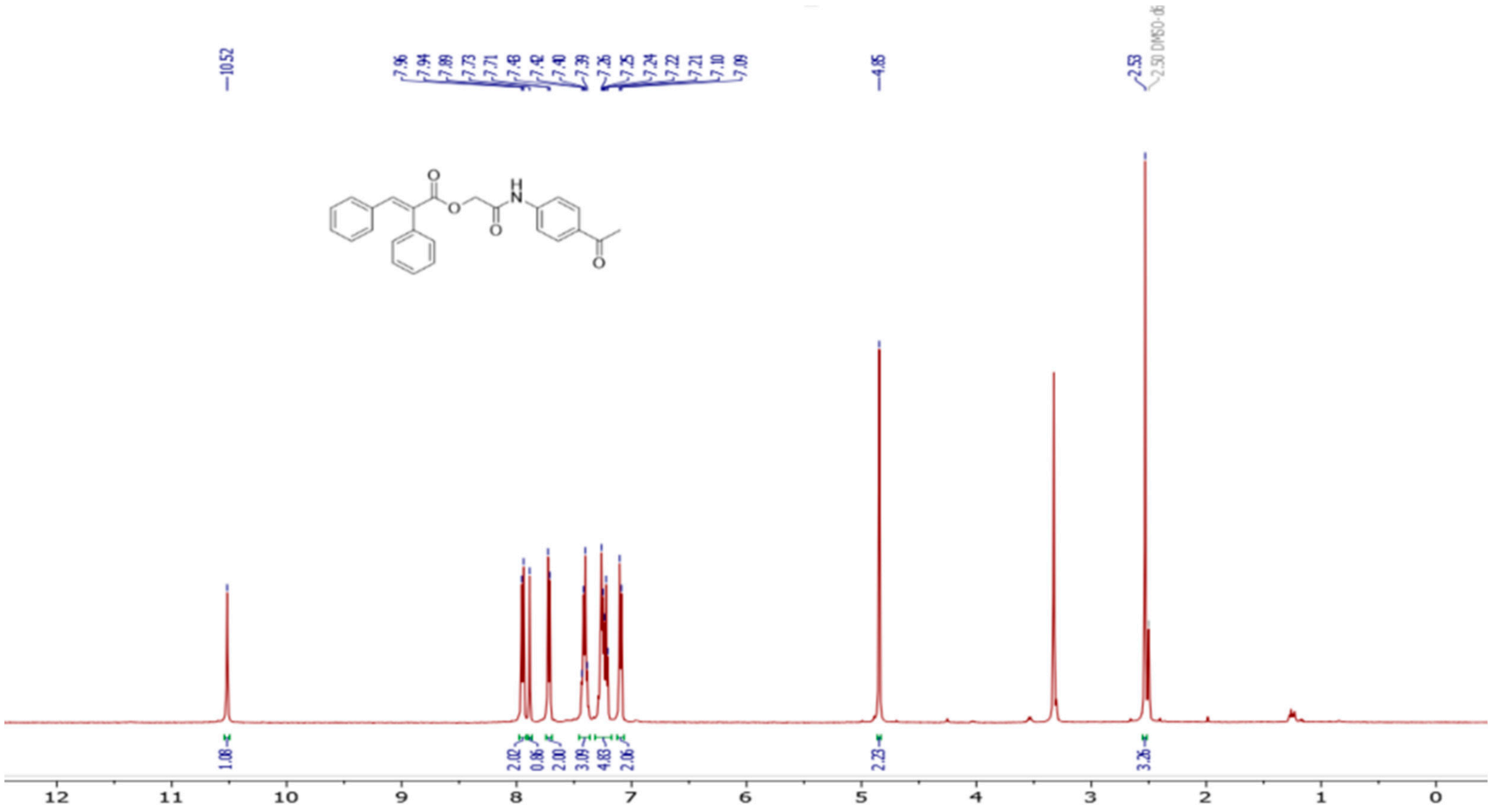

Figure S24: ^{1}H NMR spectrum of compound **5g**

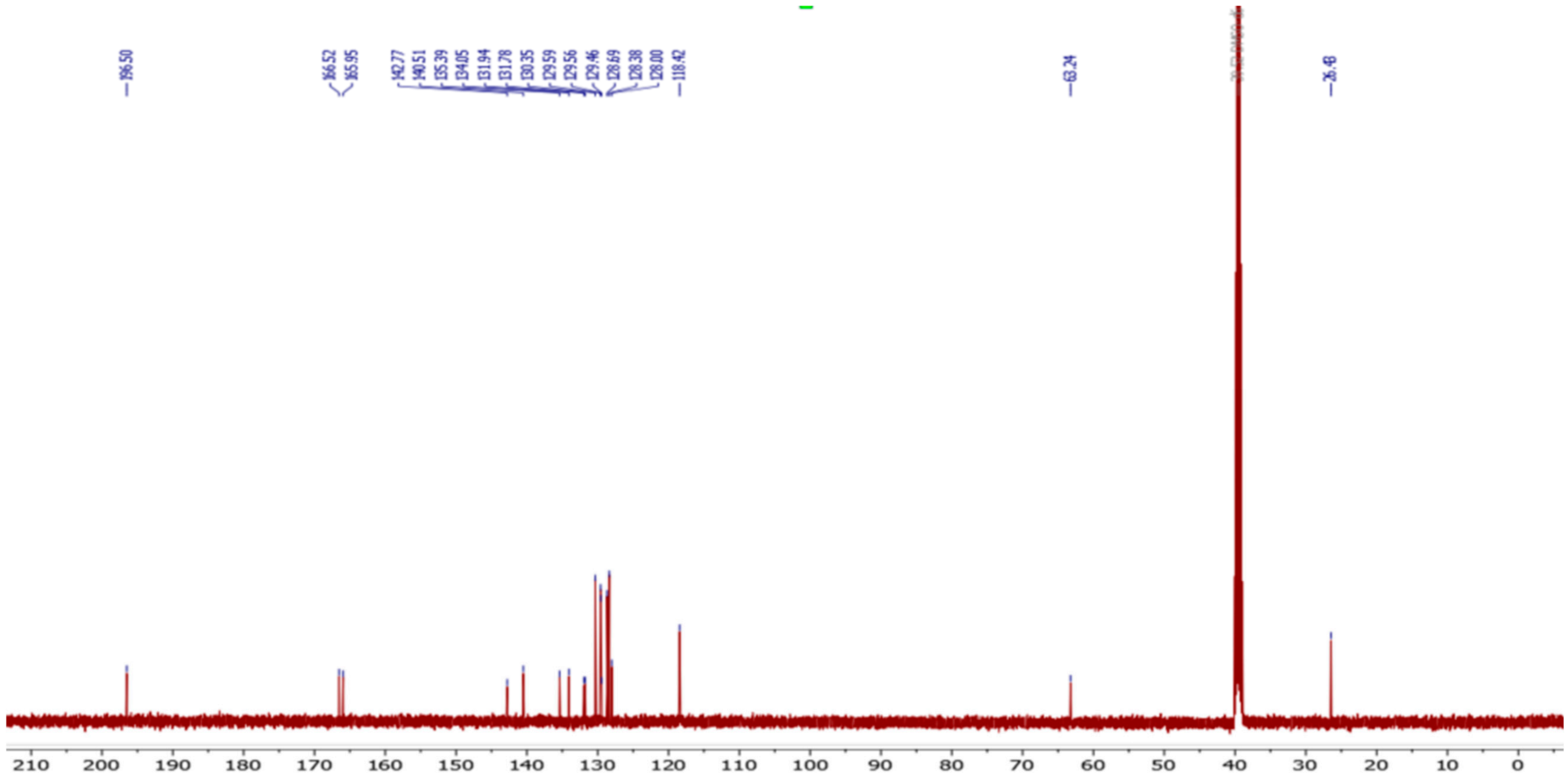

Figure S25: ^{13}C NMR spectrum of compound **5g**

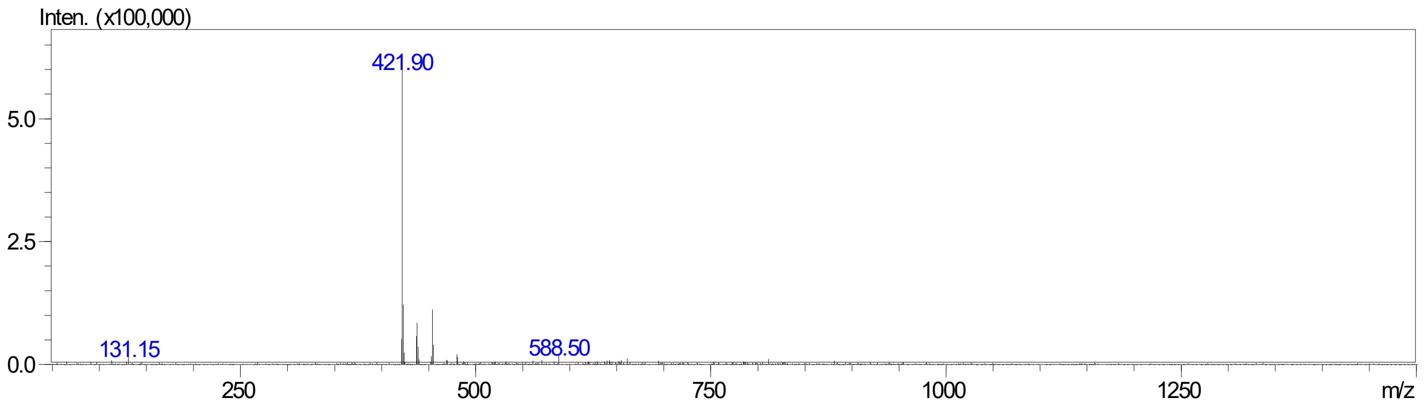

Figure S26: *Positive* LC-MS spectrum of compound **5g**

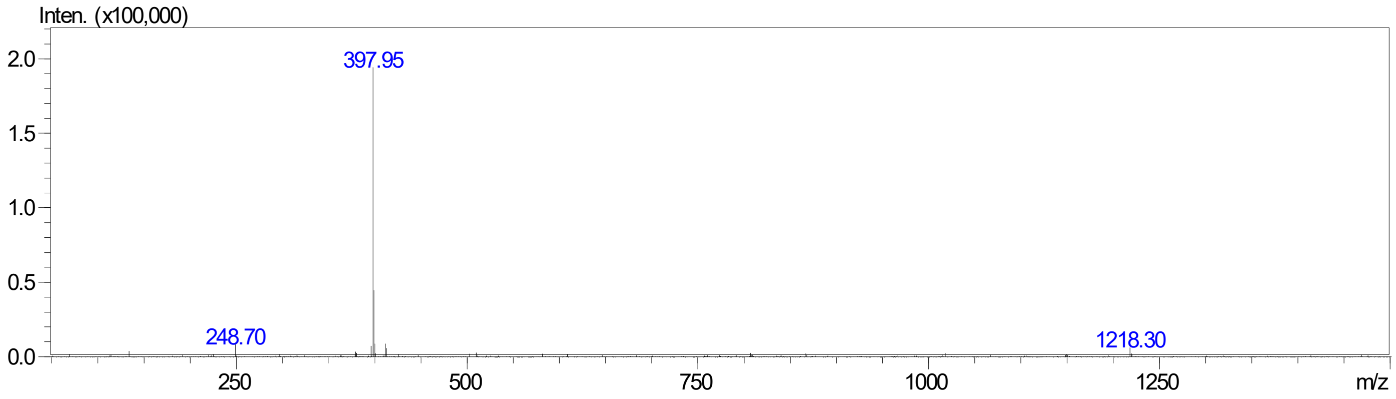

Figure S27: *Negative* LC-MS spectrum of compound **5g**

^{1}H NMR, ^{13}C NMR and LCMS spectrums of compound **5h**

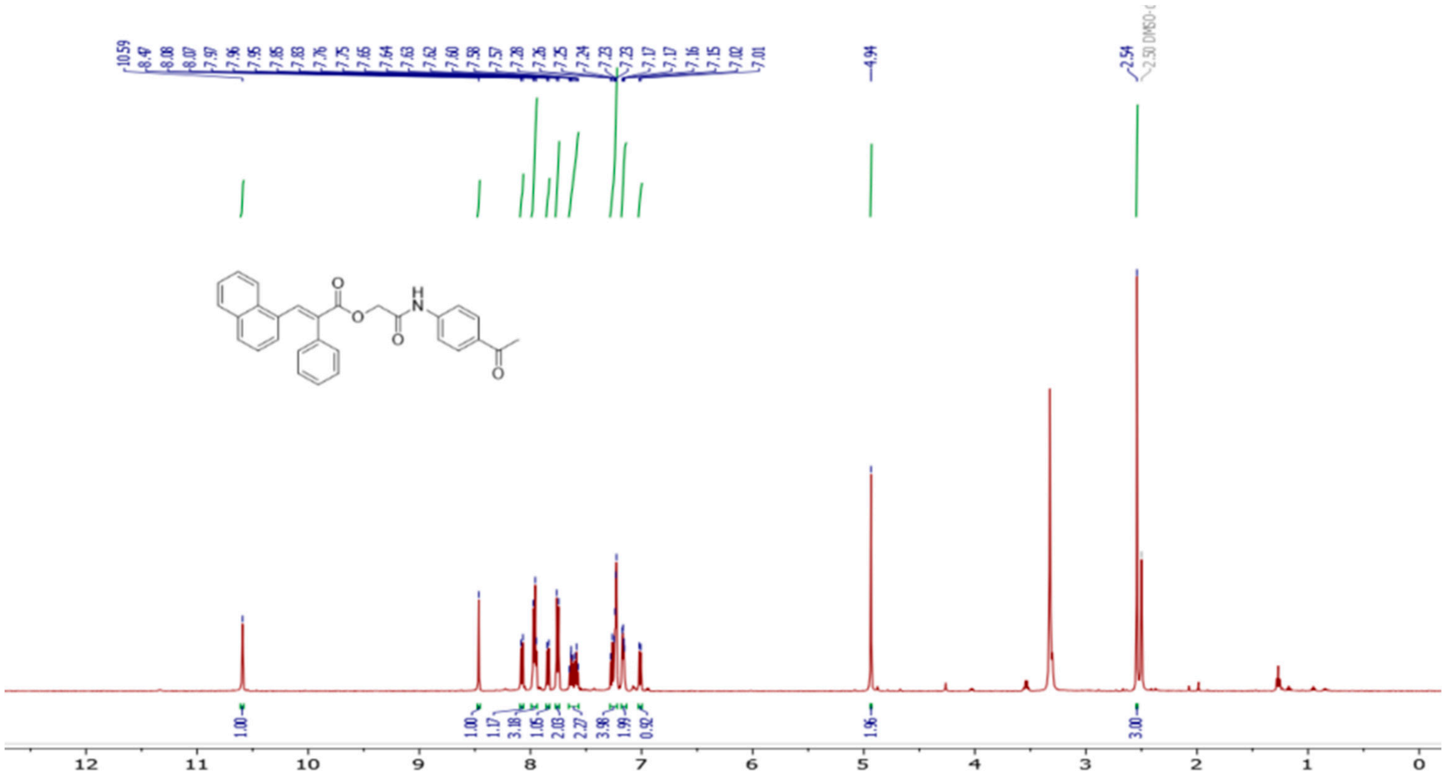

Figure S28: ^{1}H NMR spectrum of compound **5h**

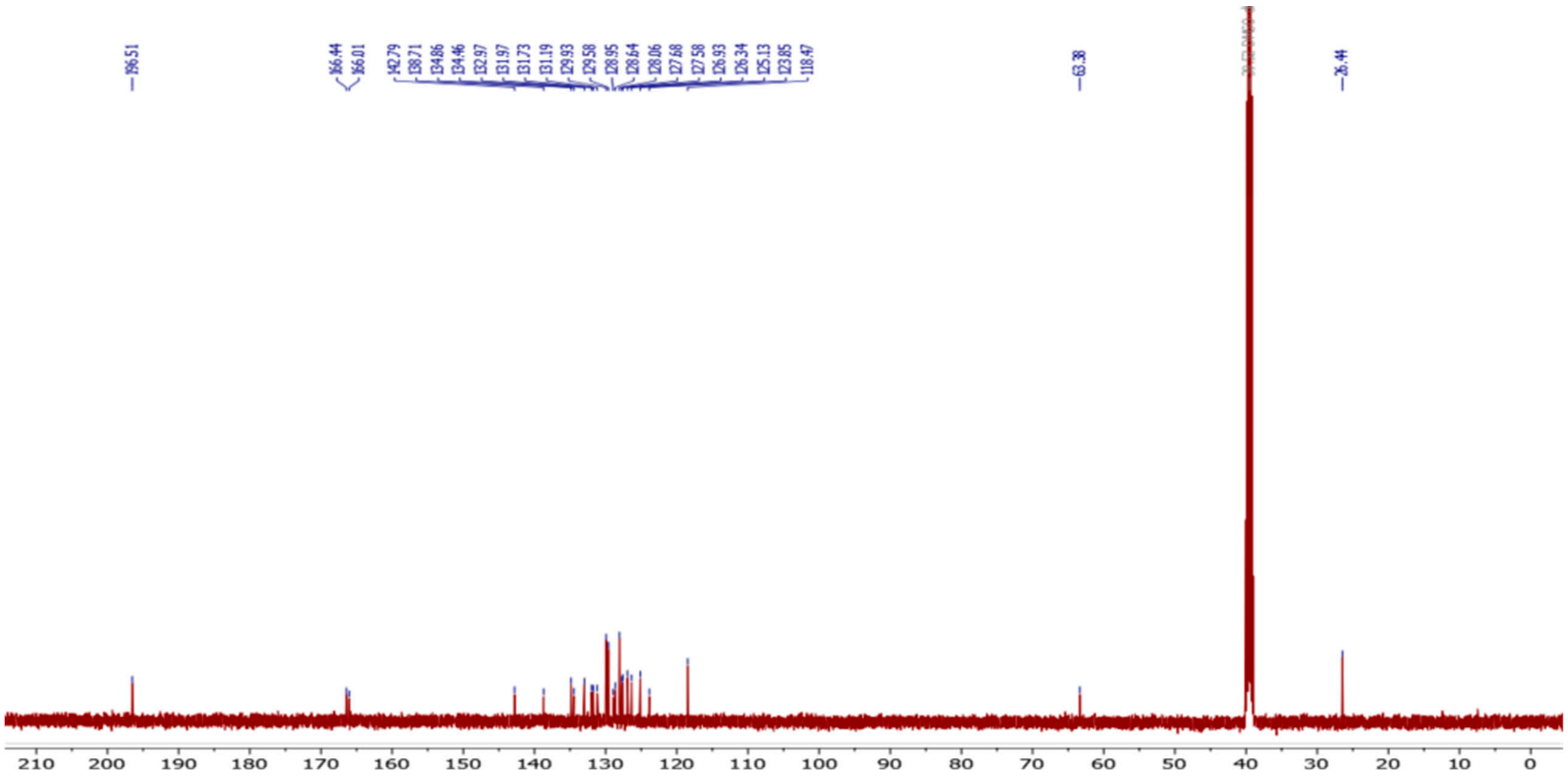

Figure S29: ^{13}C NMR spectrum of compound **5h**

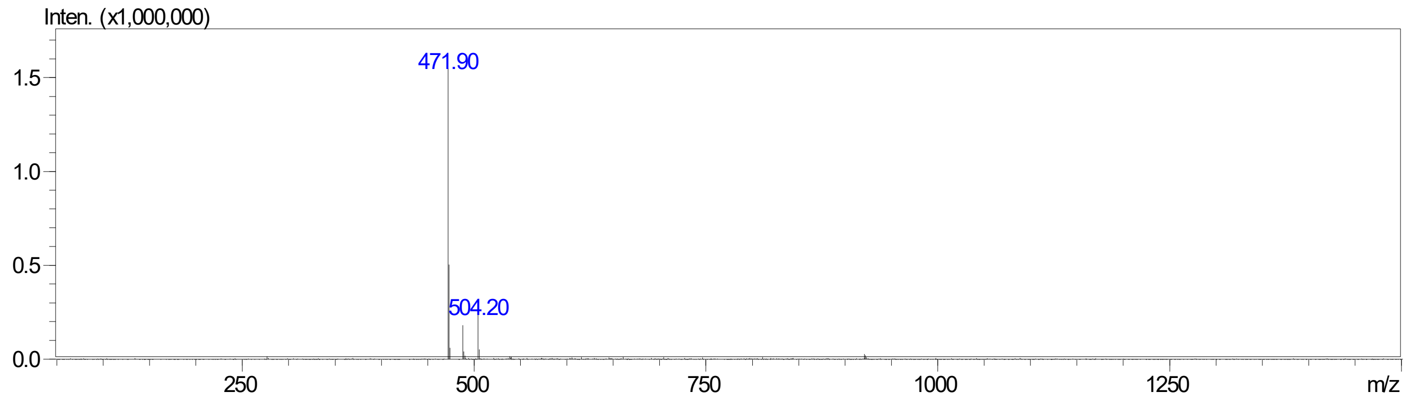

Figure S30: *Positive* LC-MS spectrum of compound **5h**

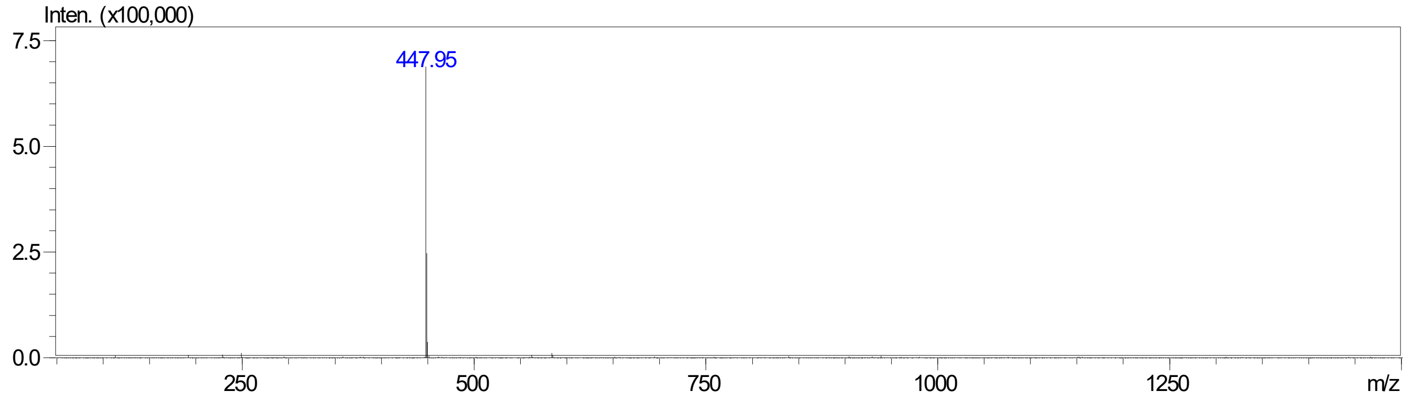

Figure S31: *Negative* LC-MS spectrum of compound **5h**

^{1}H NMR, ^{13}C NMR and LCMS spectrums of compound **5i**

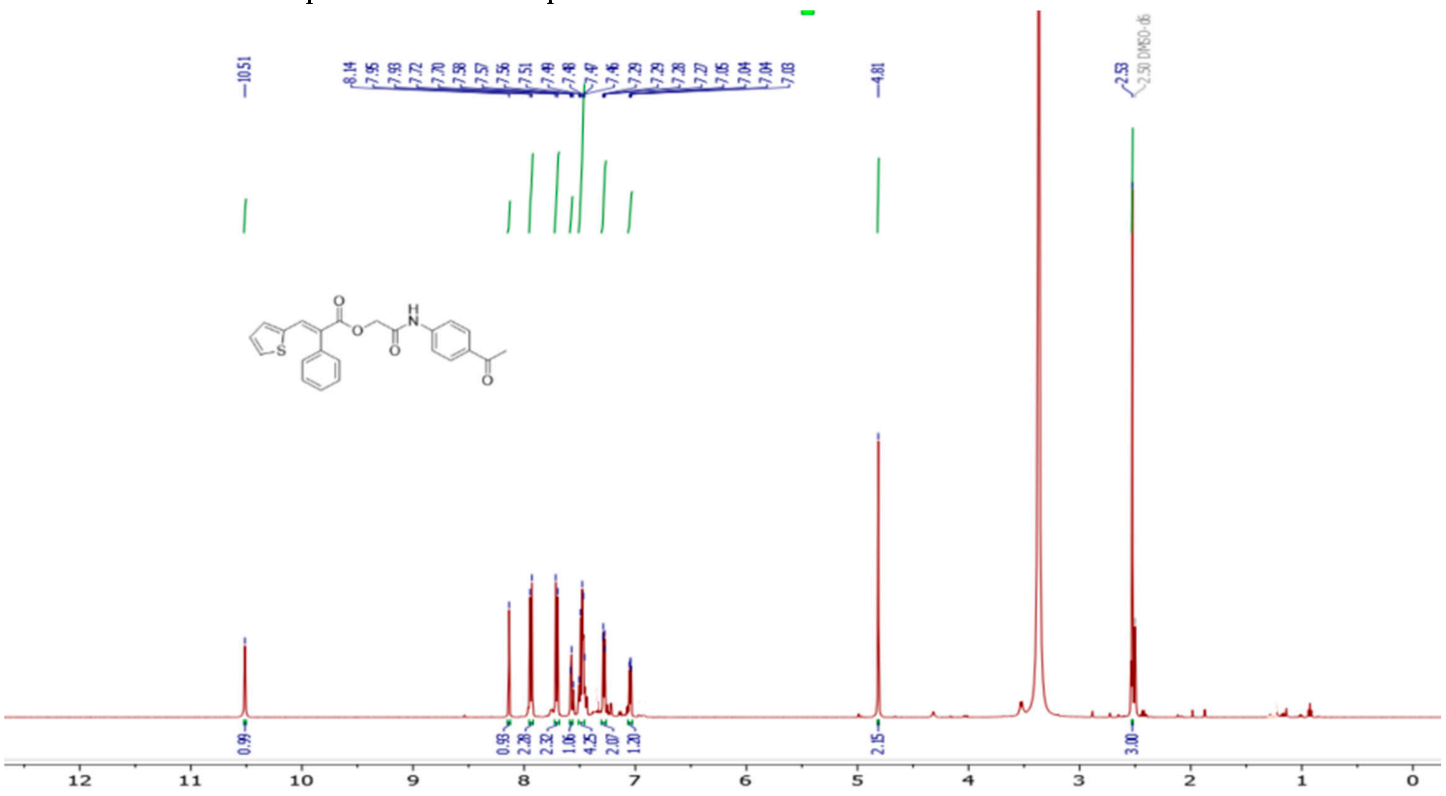

Figure S32: ^{1}H NMR spectrum of compound **5i**

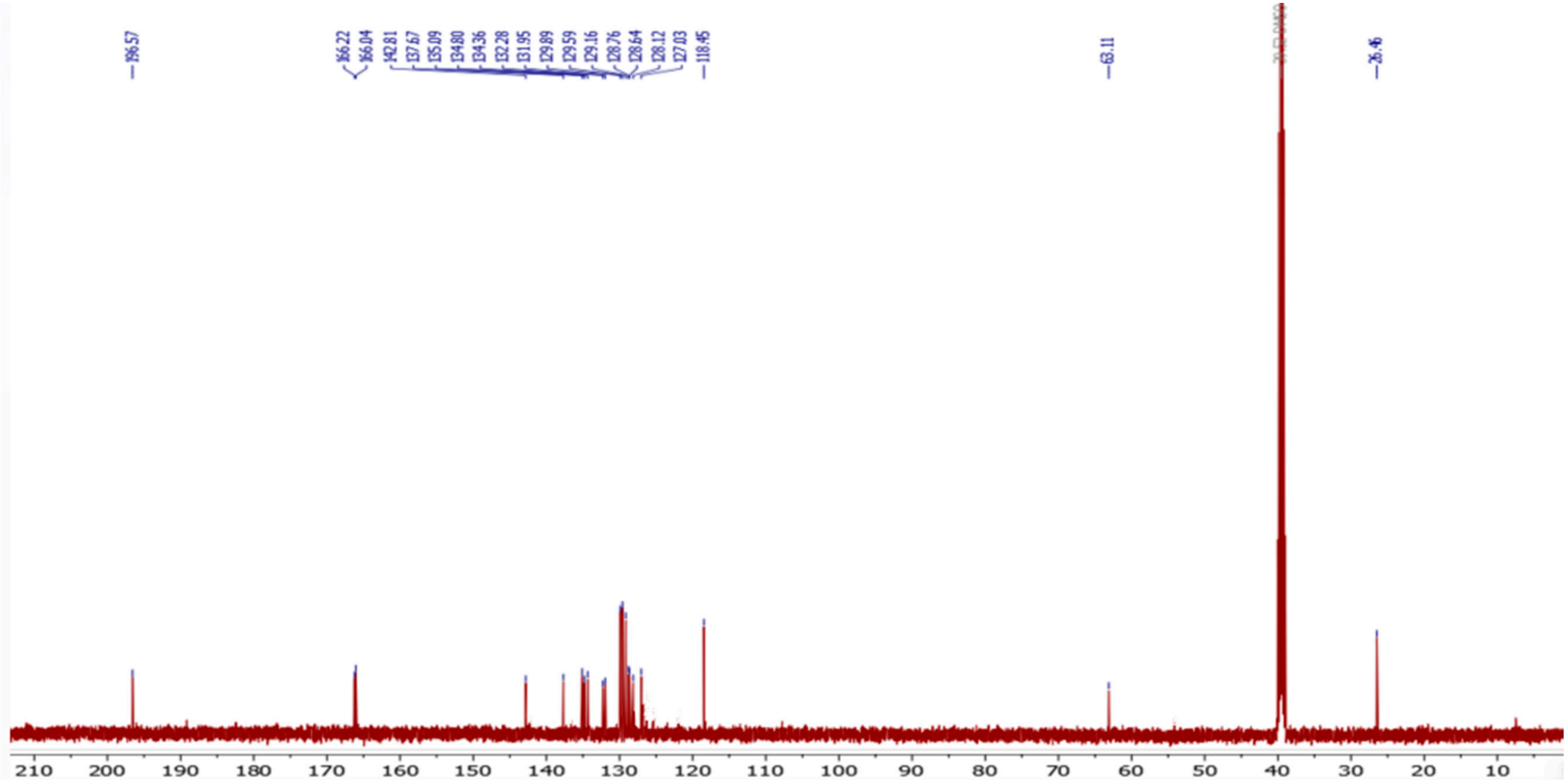

Figure S33: ^{13}C NMR spectrum of compound **5i**

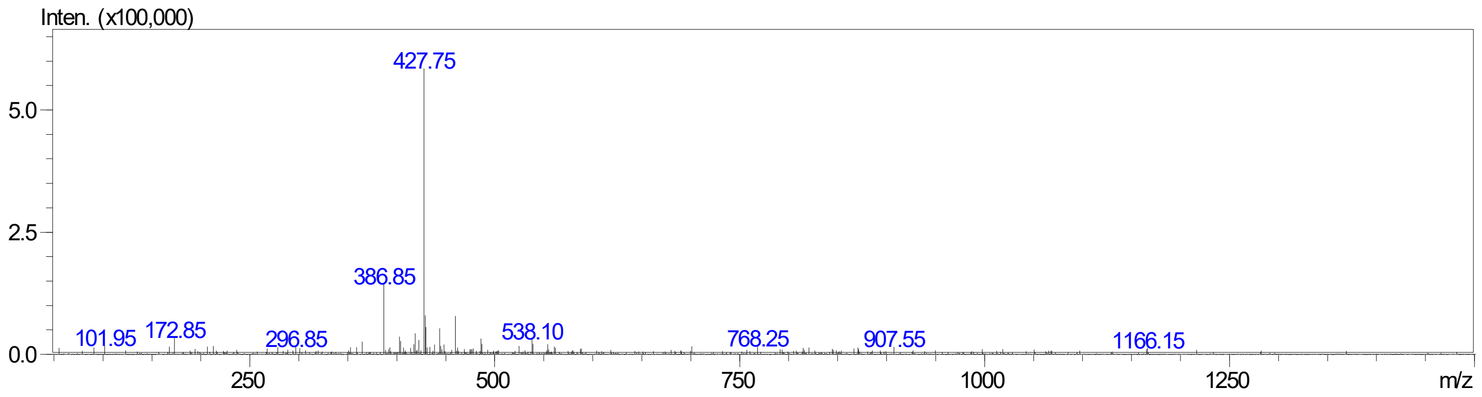

Figure S34: *Positive* LC-MS spectrum of compound **5i**

^{1}H NMR, ^{13}C NMR and LCMS spectrums of compound **6a**

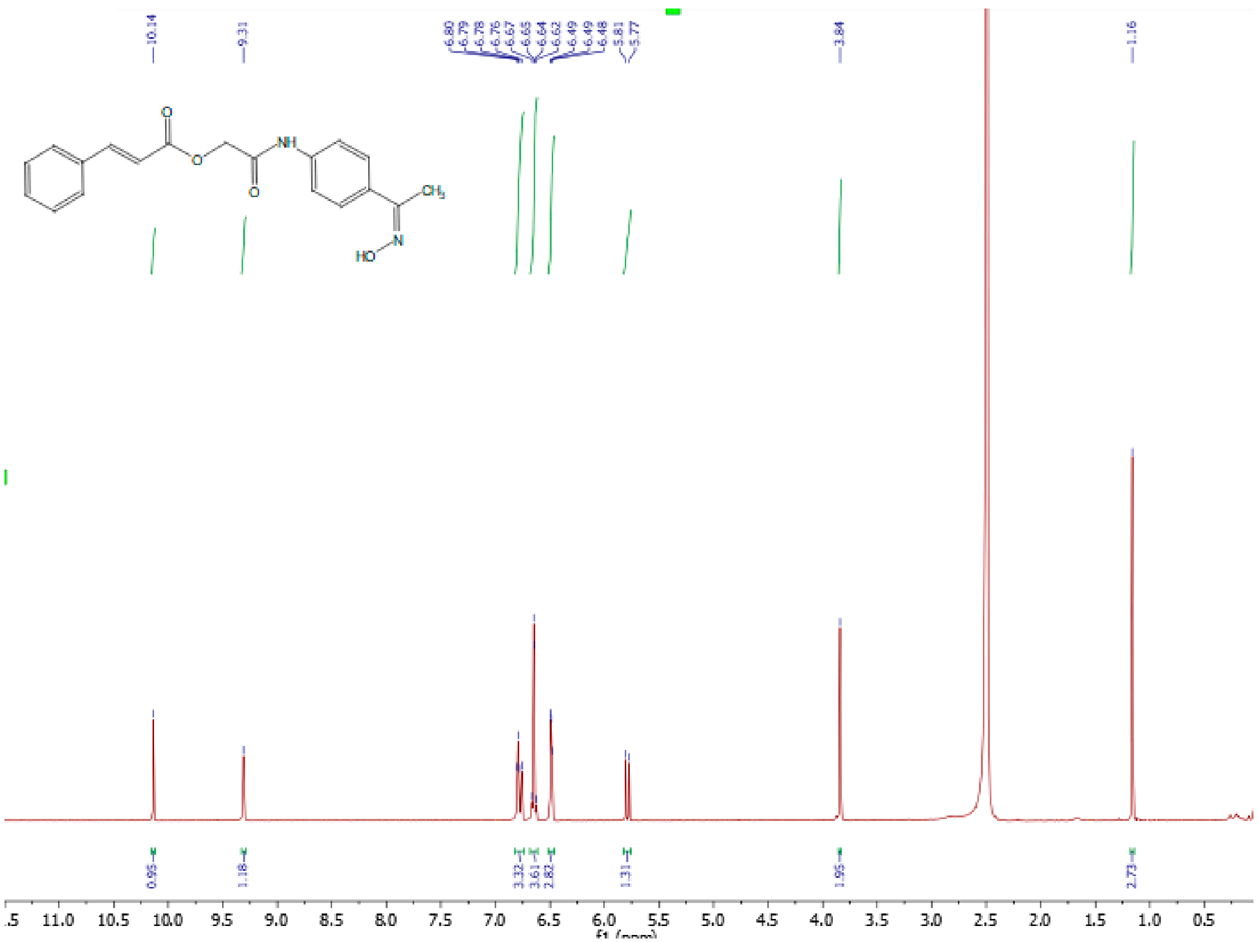

Figure S35: ^{1}H NMR spectrum of compound **6a**

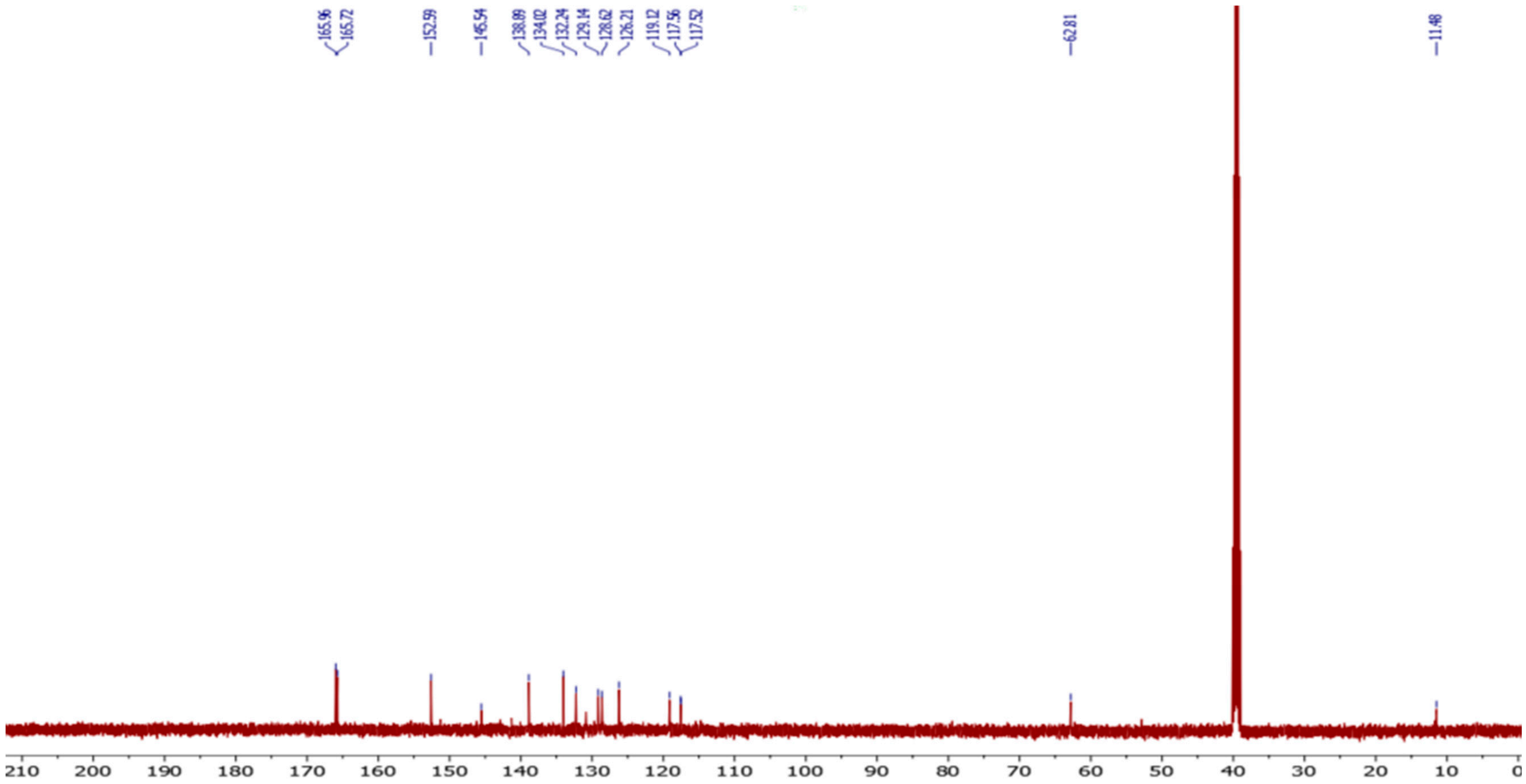

Figure S36: ^{13}C NMR spectrum of compound **6a**

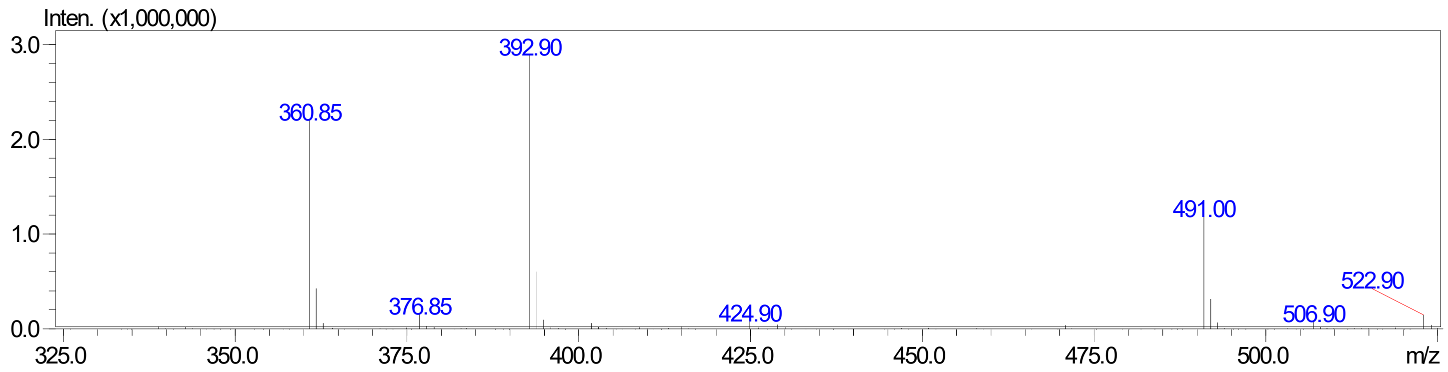

Figure S37: *Positive* LC-MS spectrum of compound **6a**

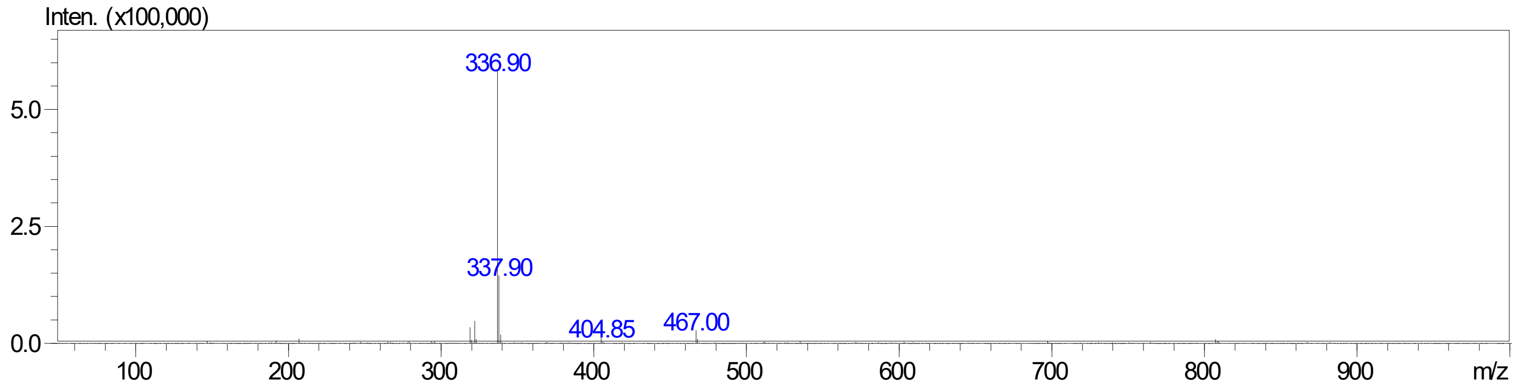

Figure S38: *Negative* LC-MS spectrum of compound **6a**

^{1}H NMR, ^{13}C NMR, HRMS and LCMS spectrums of compound **6b**

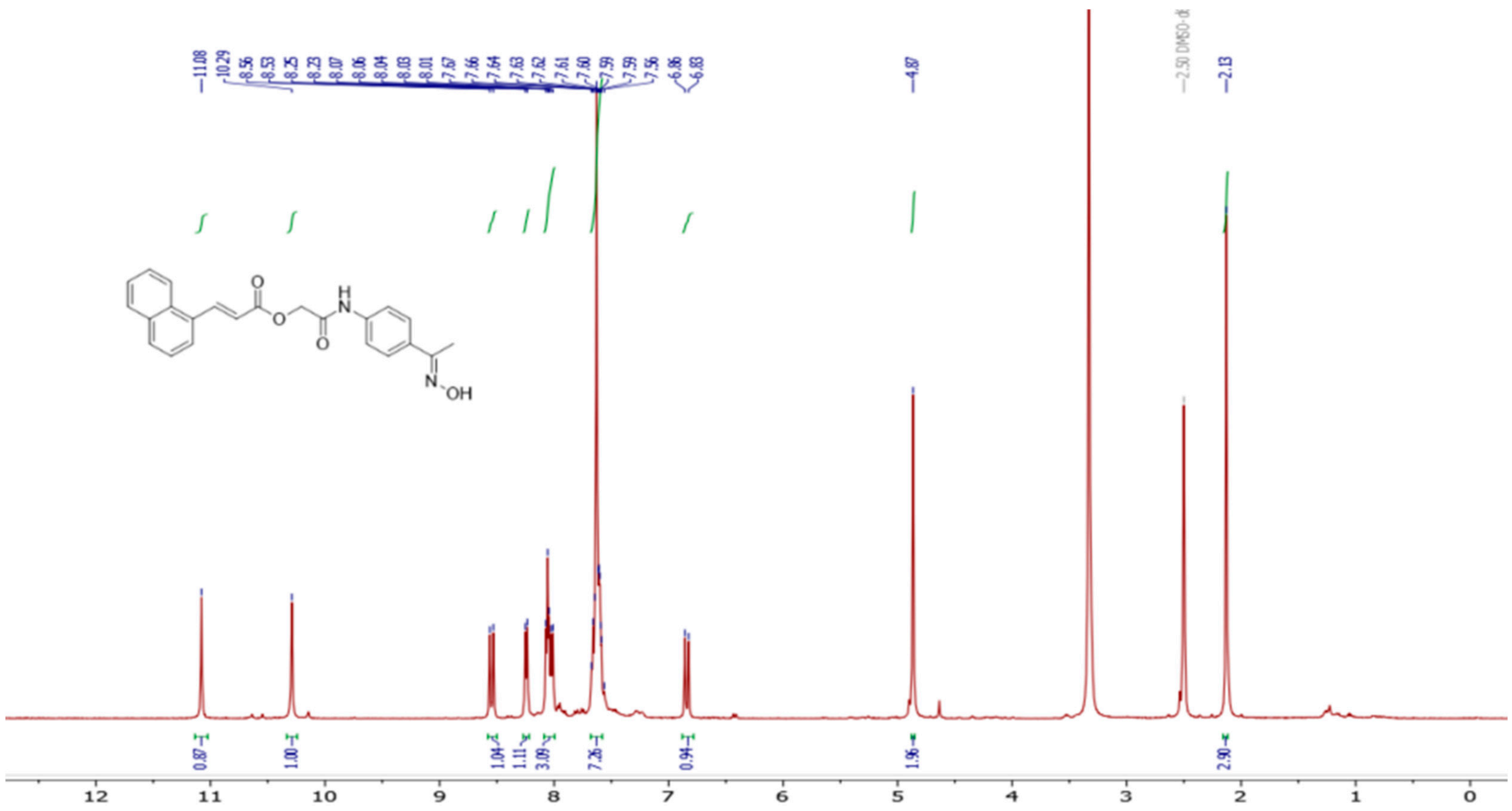

Figure S39: ^{1}H NMR spectrum of compound **6b**

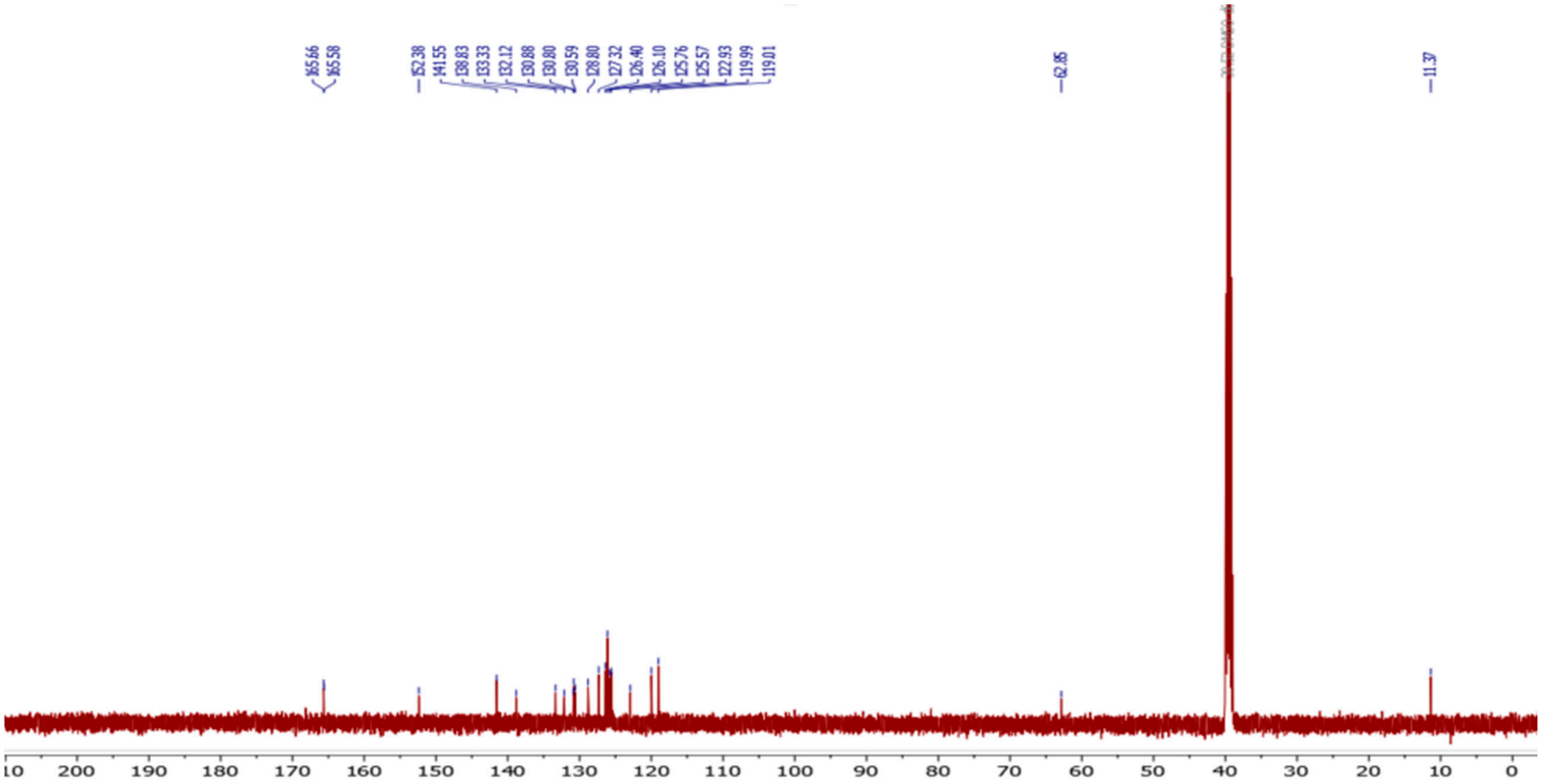

Figure S40: ^{13}C NMR spectrum of compound **6b**

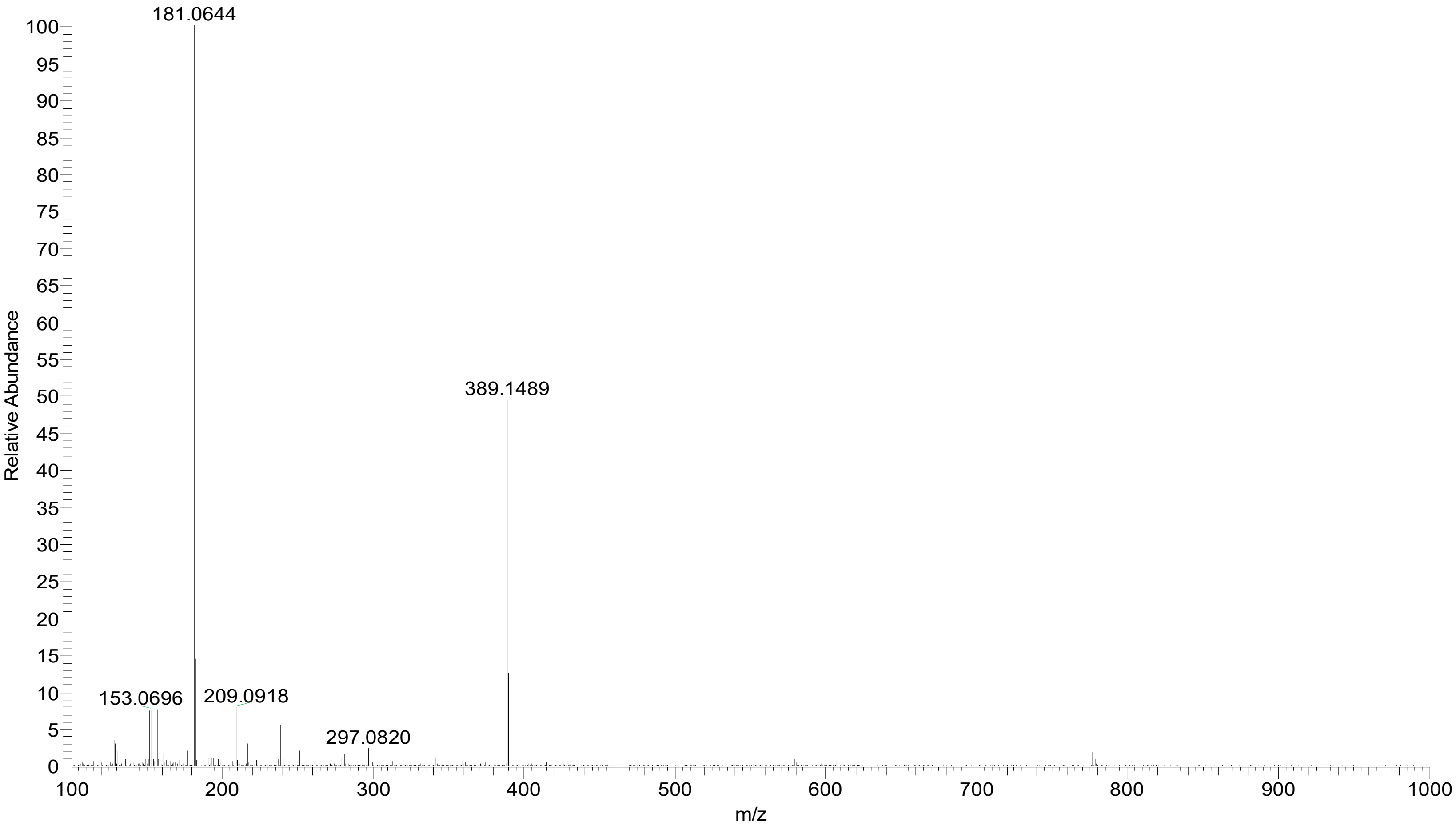

Figure S41: *Positive* HRMS spectrum of compound **6b**

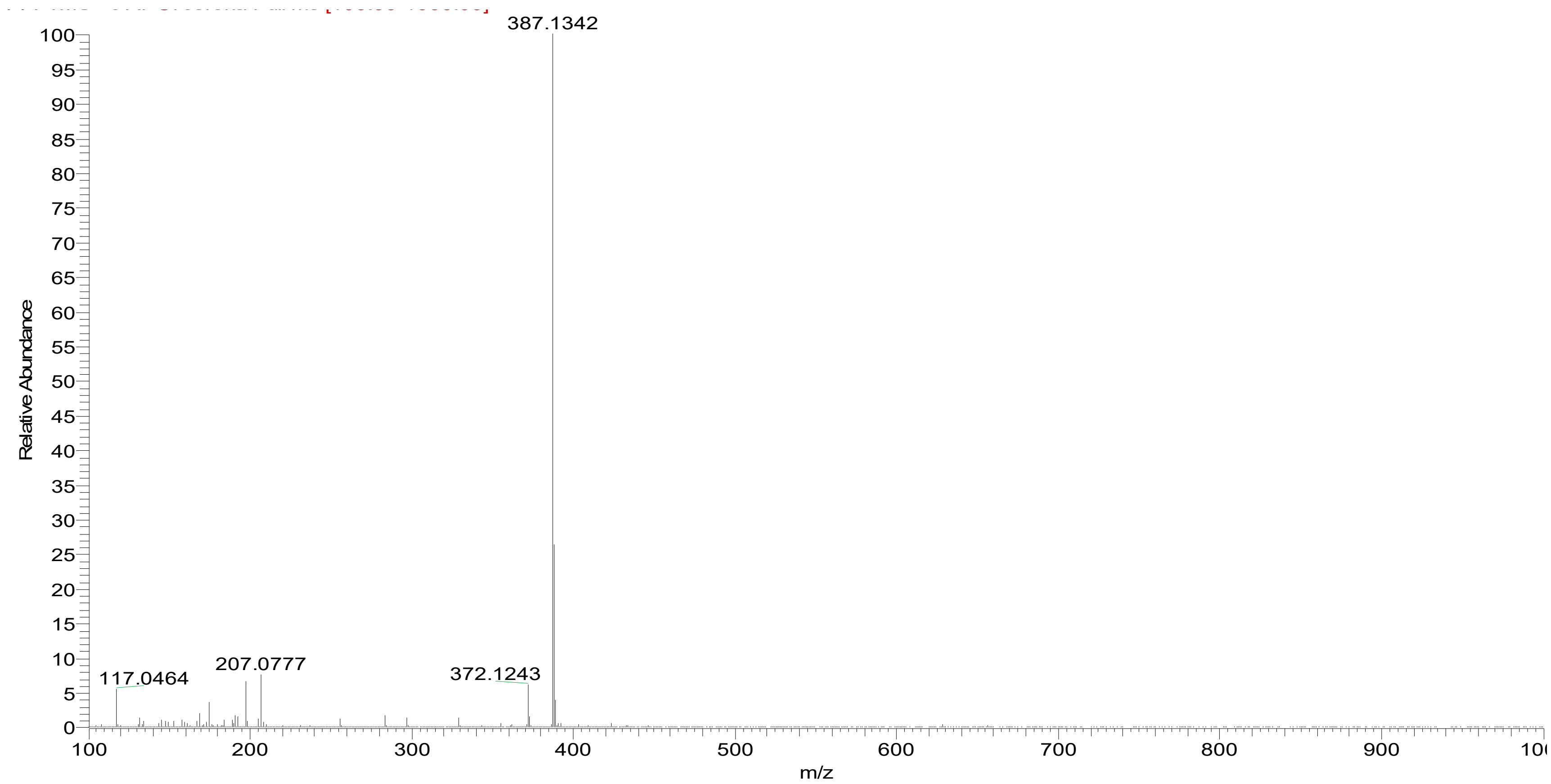

Figure S42: *Negative* HRMS spectrum of compound **6b**

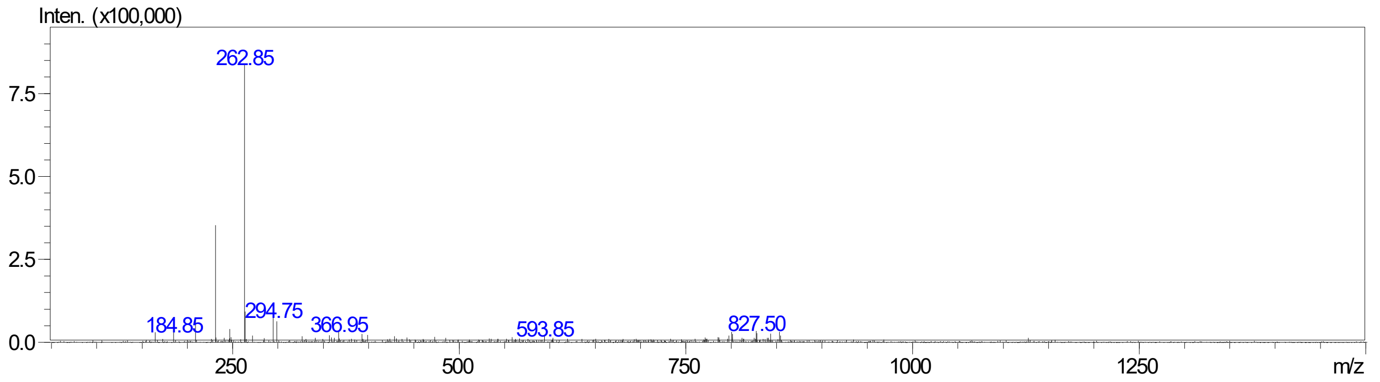

Figure S43: *Positive* LC-MS spectrum of compound **6b**

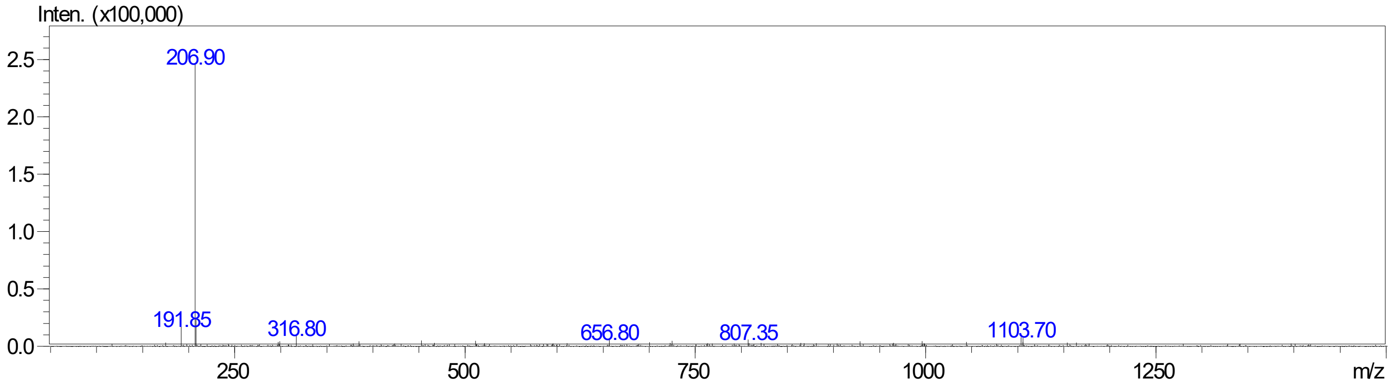

Figure S44: *Negative* LC-MS spectrum of compound **6b**

^{1}H NMR, ^{13}C NMR, HRMS and LCMS spectrums of compound **6c**

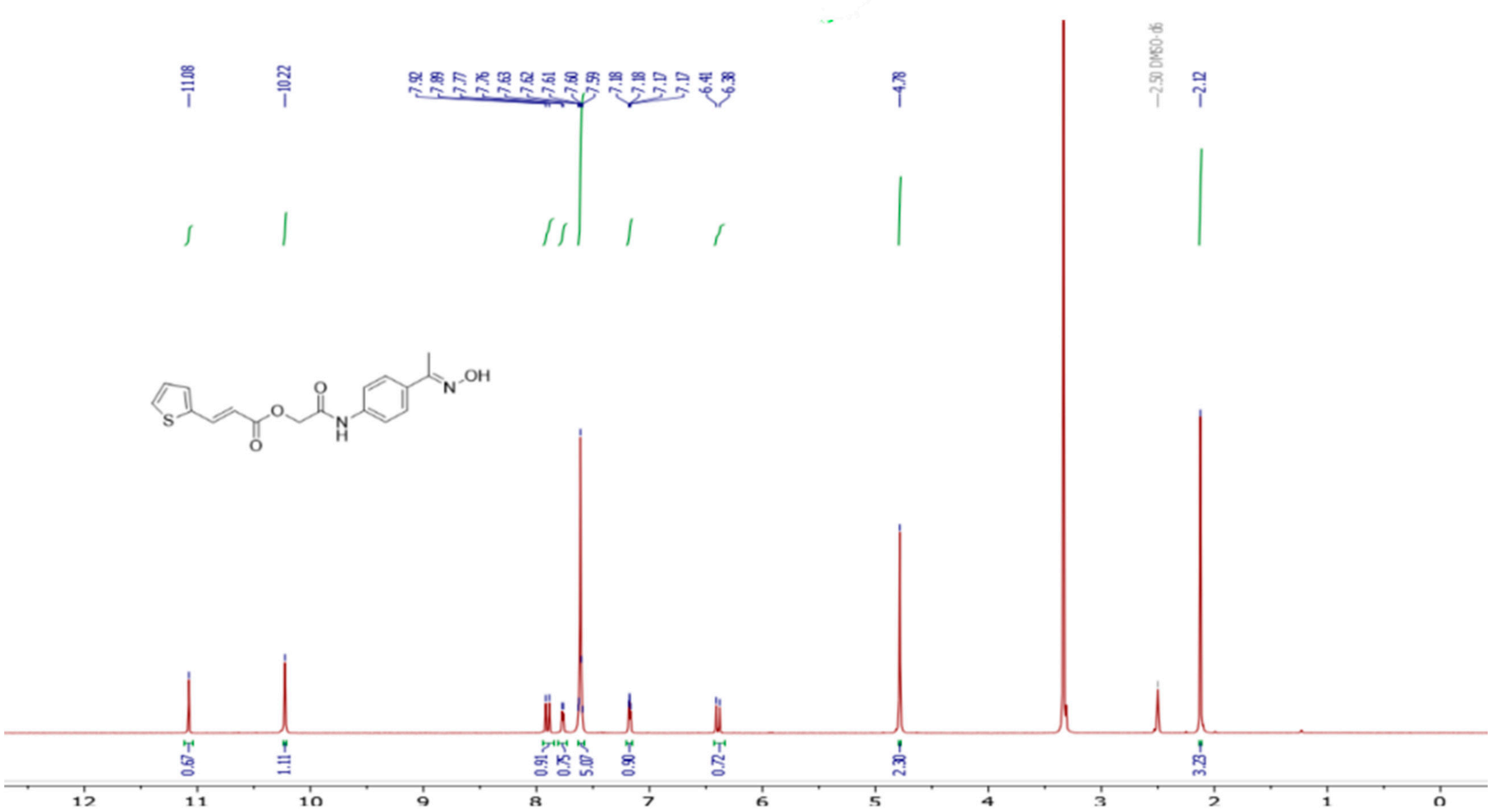

Figure S45: ^{1}H NMR spectrum of compound **6c**

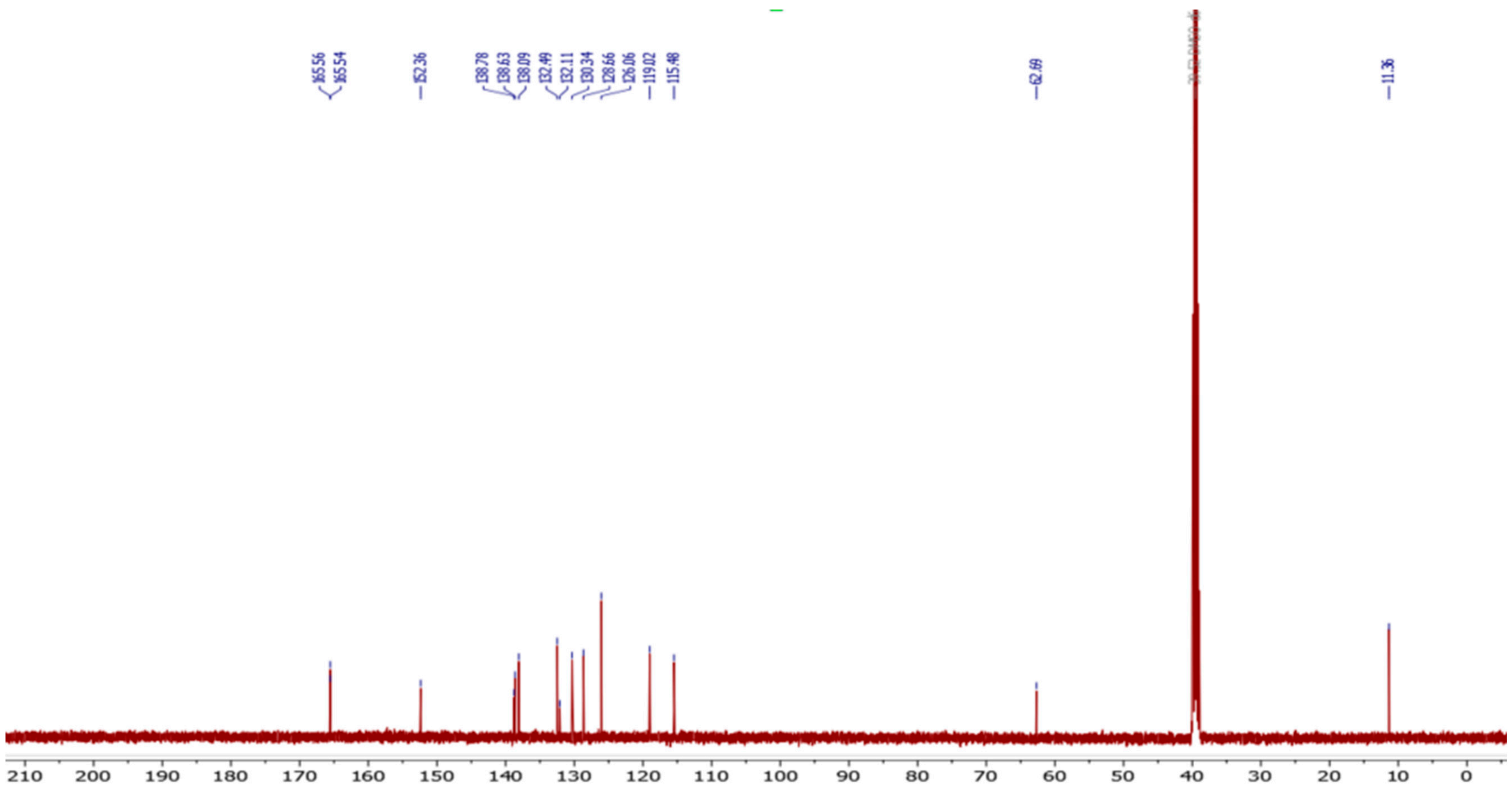

Figure S46: ^{13}C NMR spectrum of compound **6c**

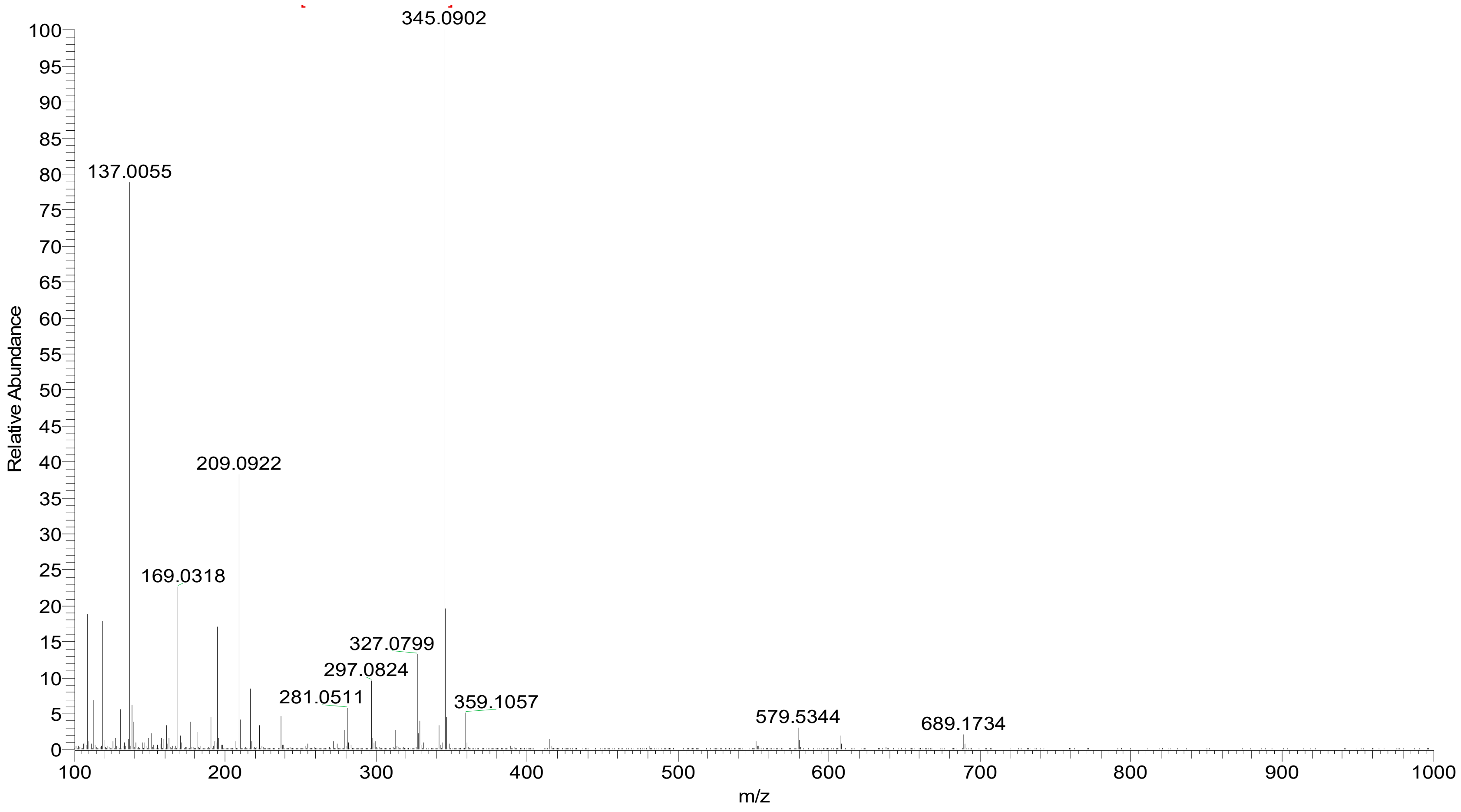

Figure S47: *Positive* HRMS spectrum of compound **6c**

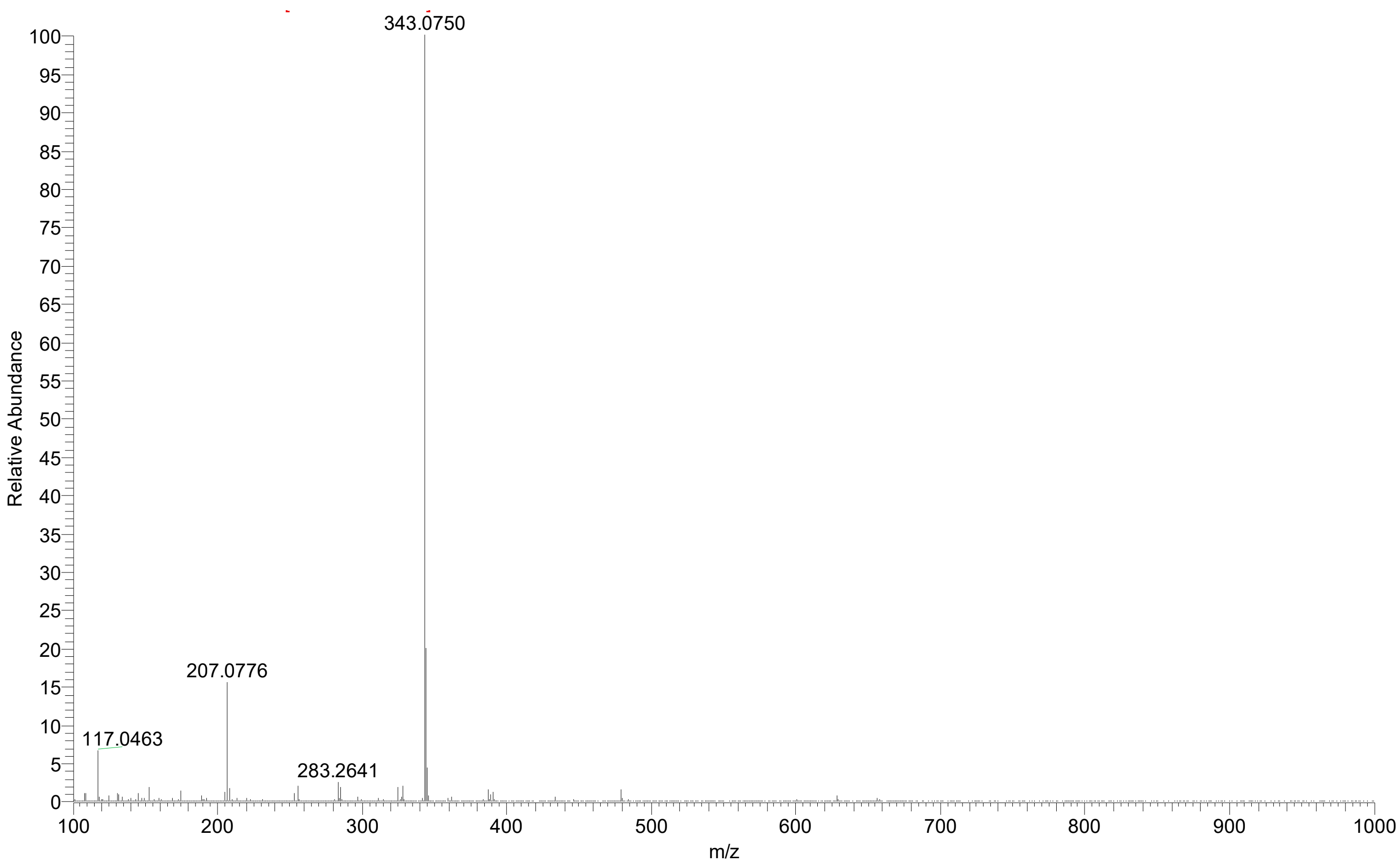

Figure S48: *Negative* HRMS spectrum of compound **6c**

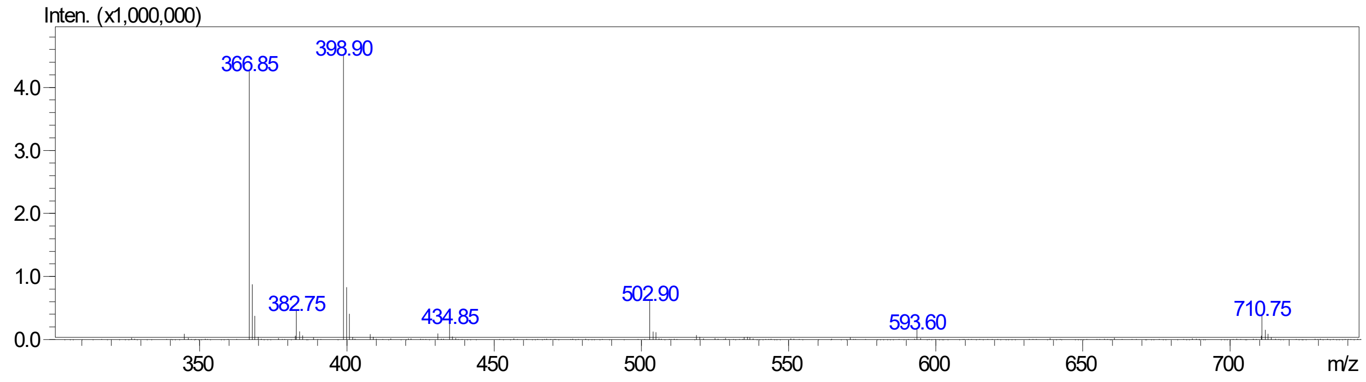

Figure S49: *Positive* LC-MS spectrum of compound **6c**

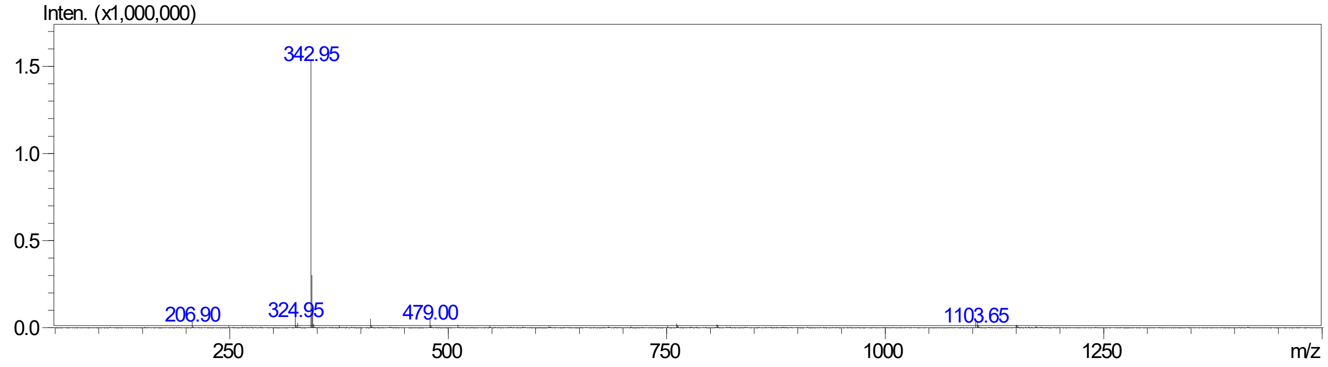

Figure S50: *Negative* LC-MS spectrum of compound **6c**

^1H NMR, ^{13}C NMR, HRMS and LCMS spectrums of compound **6d**

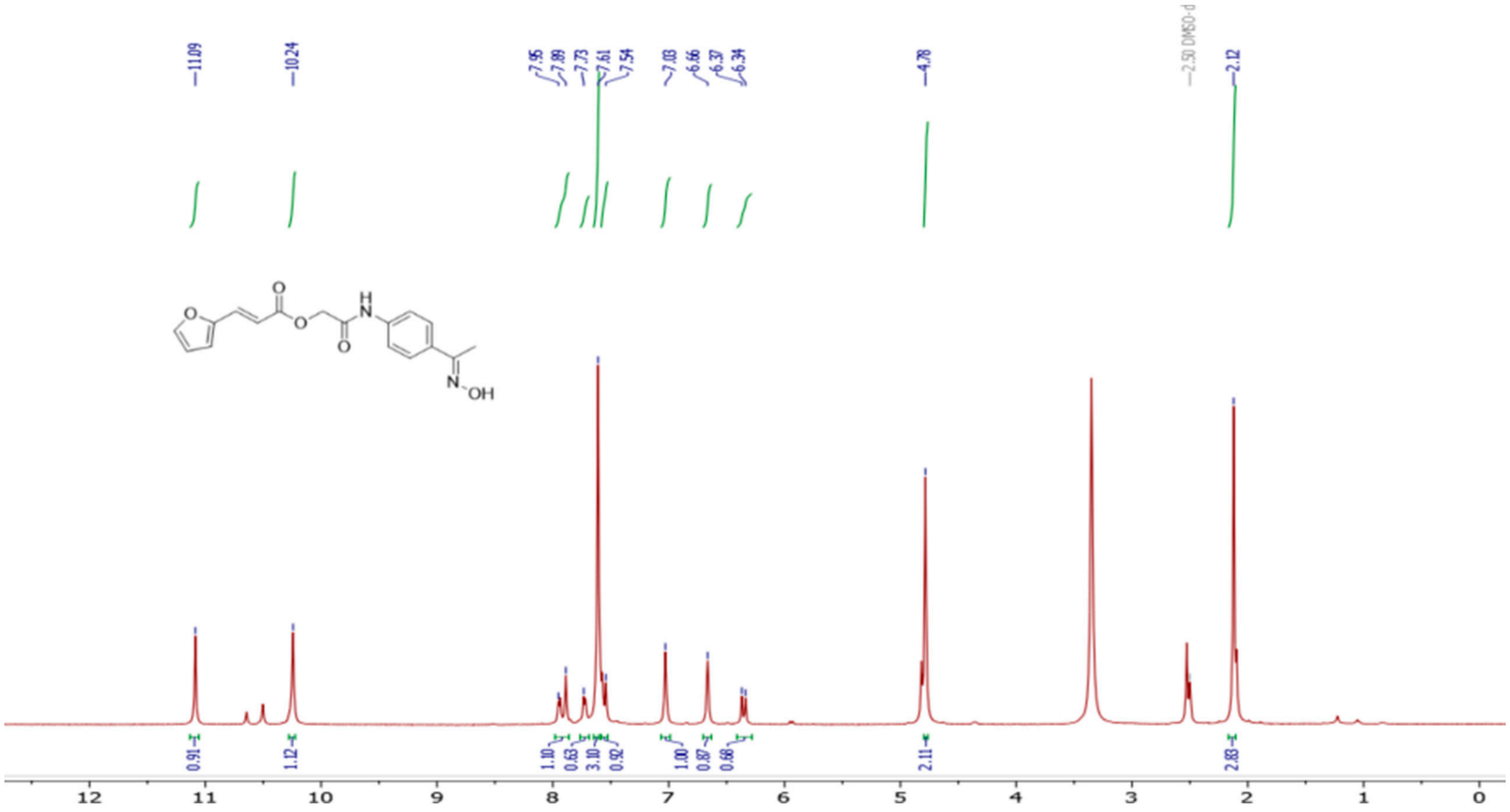

Figure S51: ^1H NMR spectrum of compound **6d**

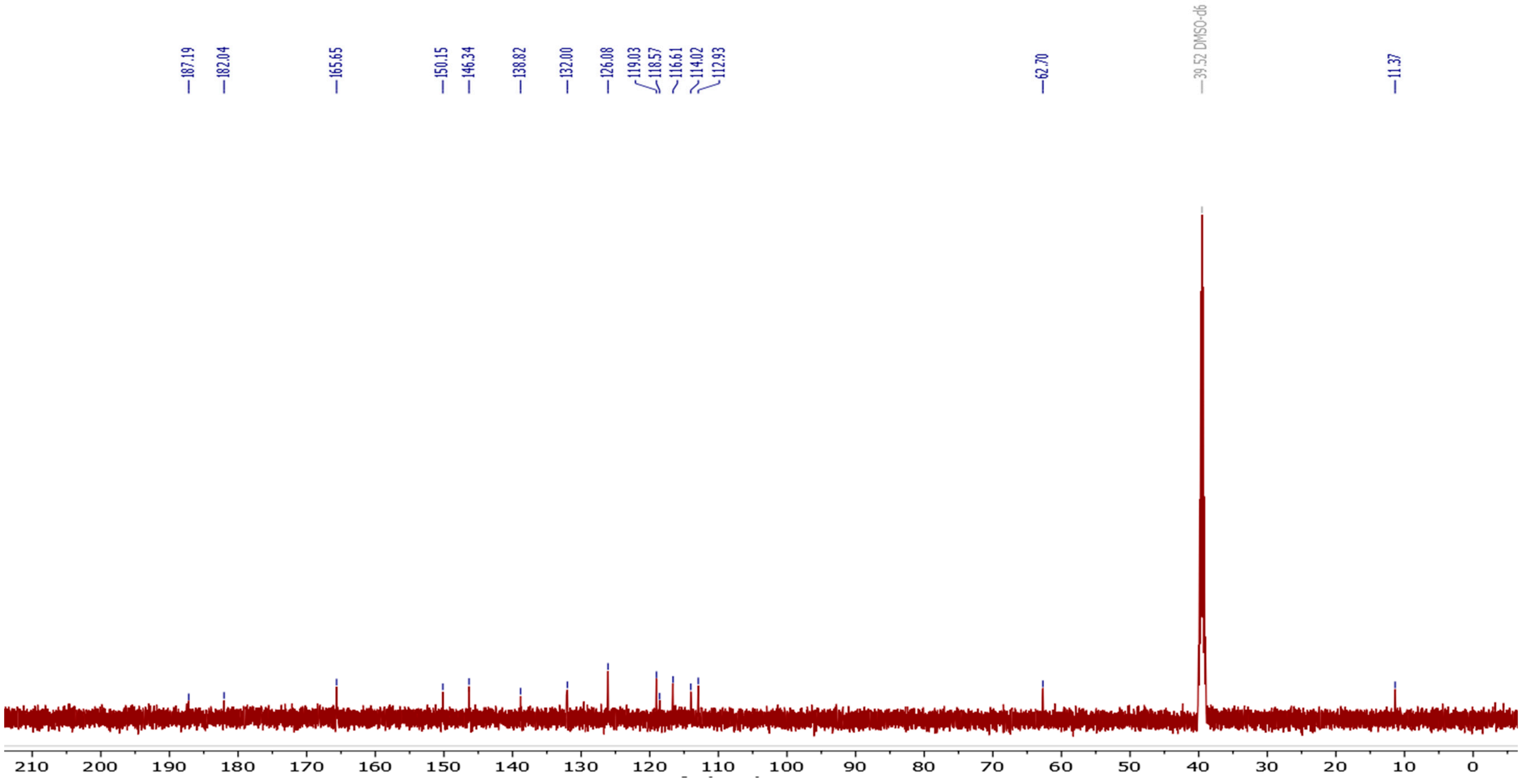

Figure S52: ^{13}C NMR spectrum of compound **6d**

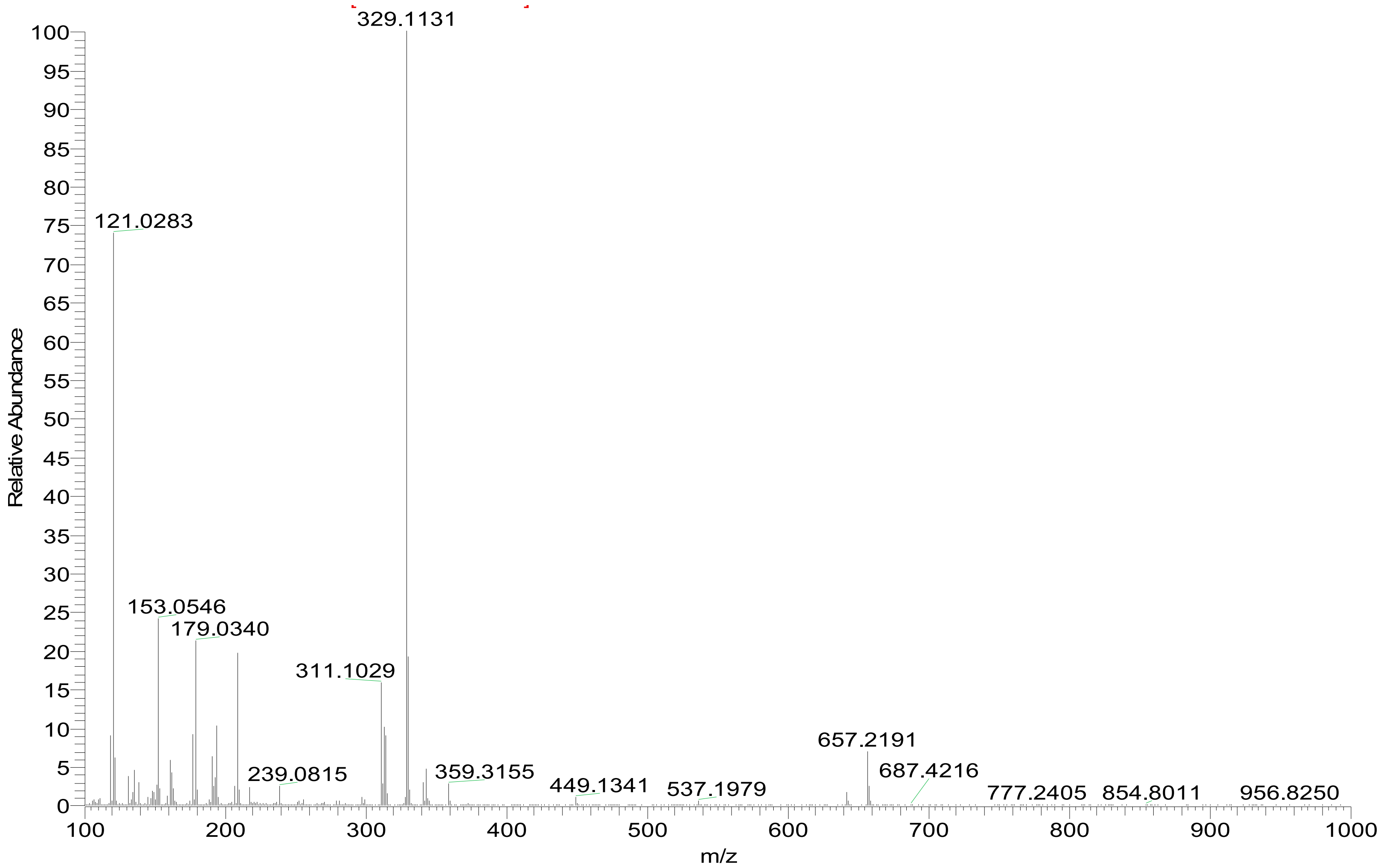

Figure S53: *Positive* HRMS spectrum of compound **6d**

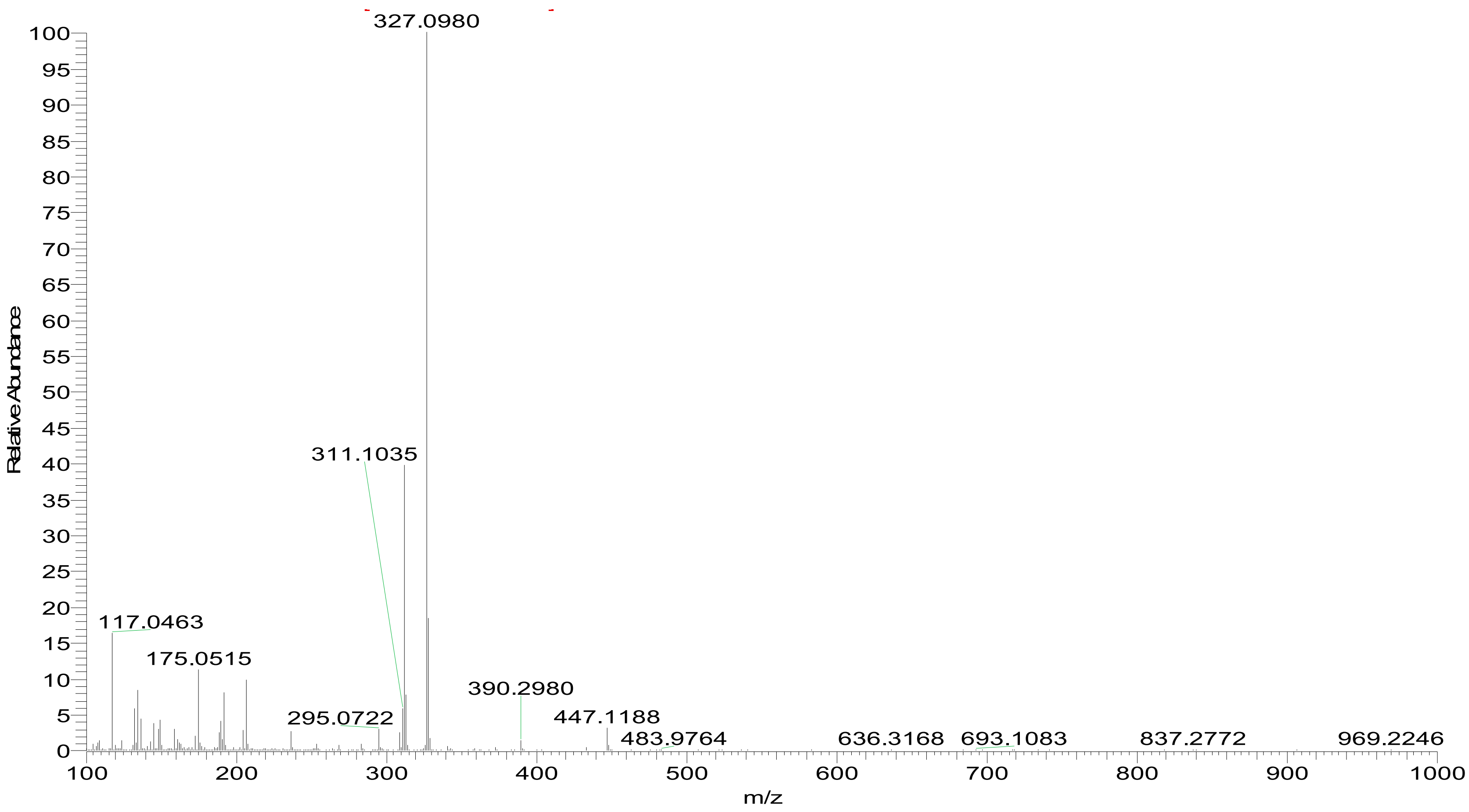

Figure S54: *Negative* LC-MS spectrum of compound **6d**

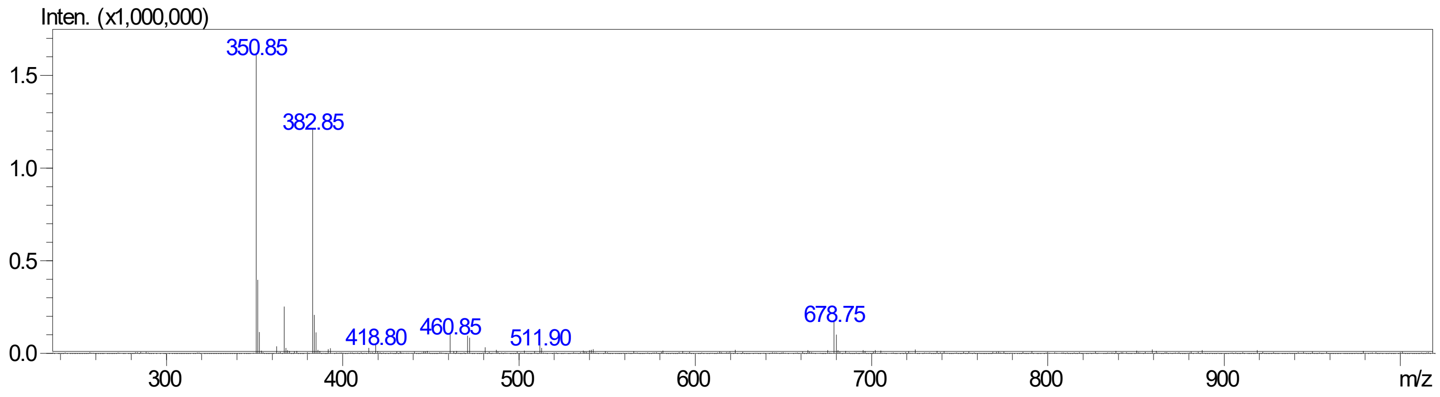

Figure S55: *Positive* LC-MS spectrum of compound **6d**

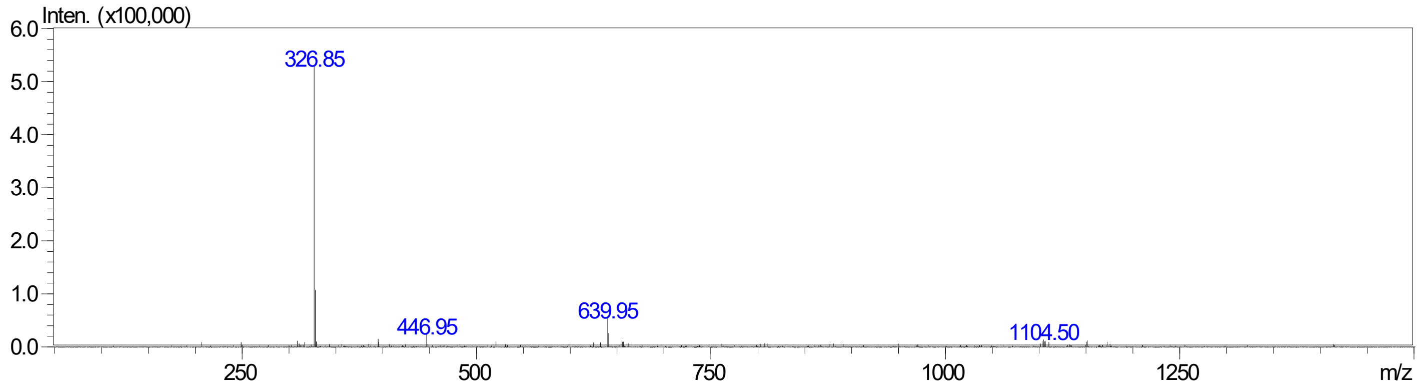

Figure S56: *Negative* LC-MS spectrum of compound **6d**

^{1}H NMR, ^{13}C NMR, HRMS and LCMS spectrums of compound **6e**

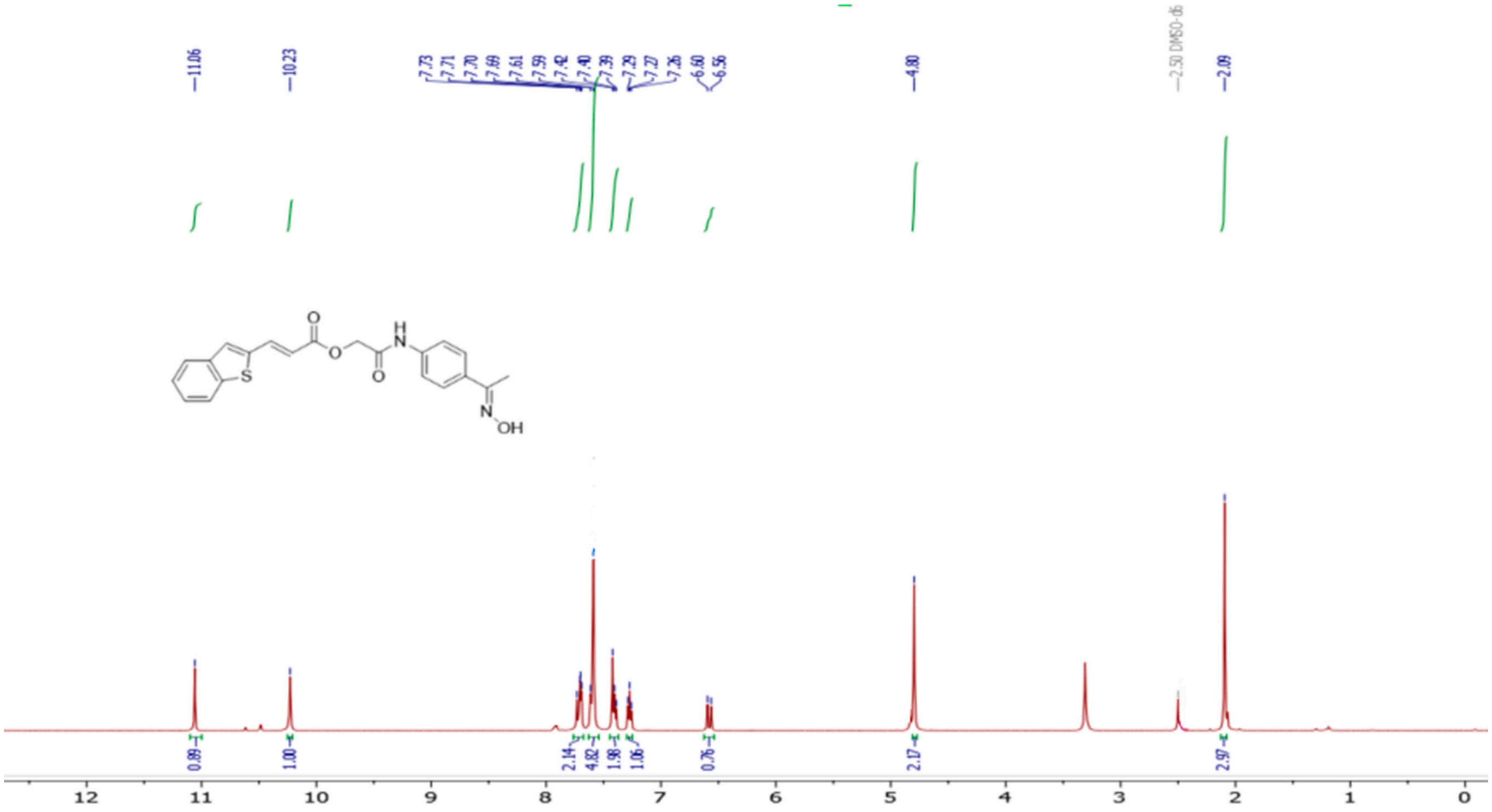

Figure S57: ^{1}H NMR spectrum of compound **6e**

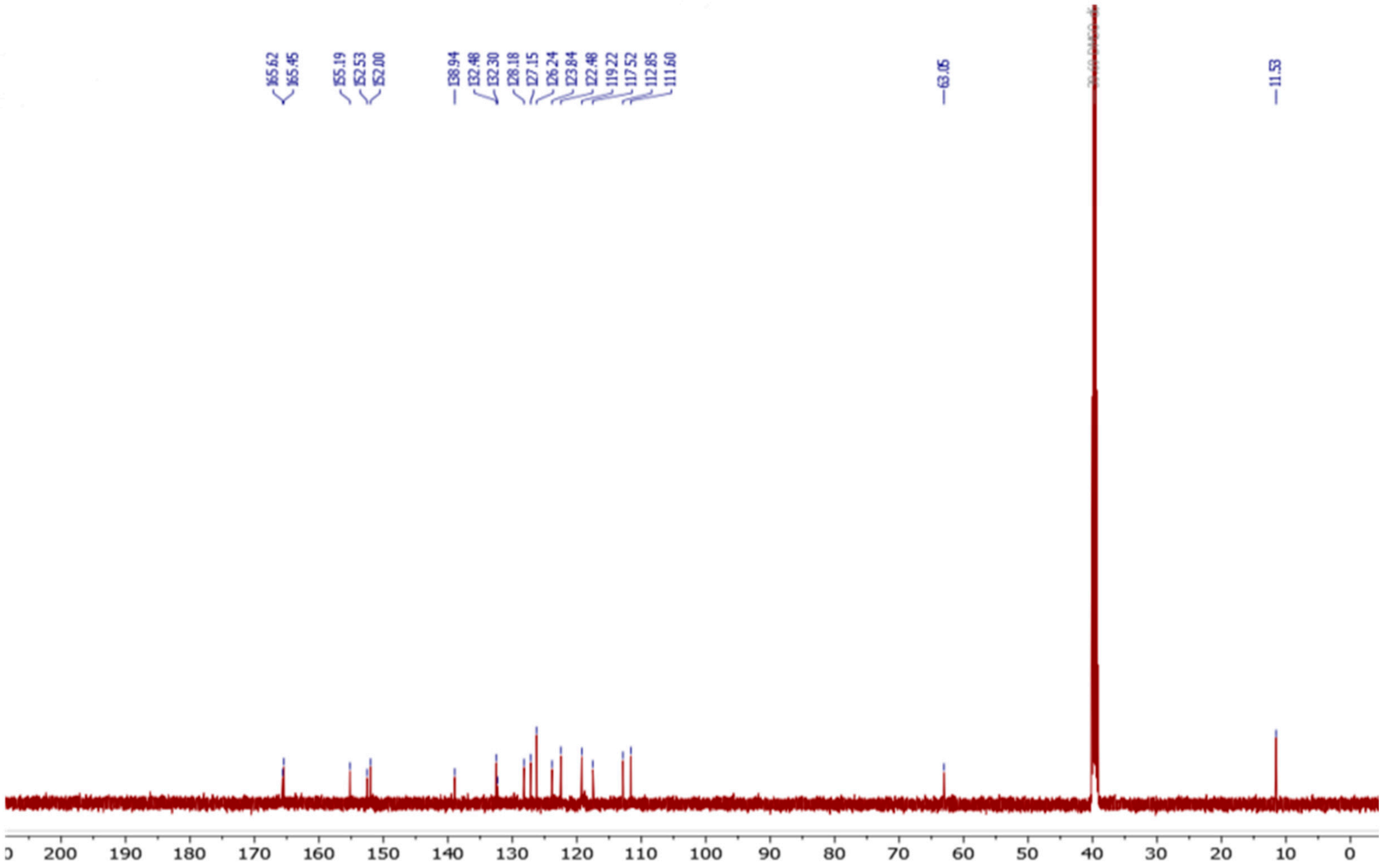

Figure S58: ^{13}C NMR spectrum of compound **6e**

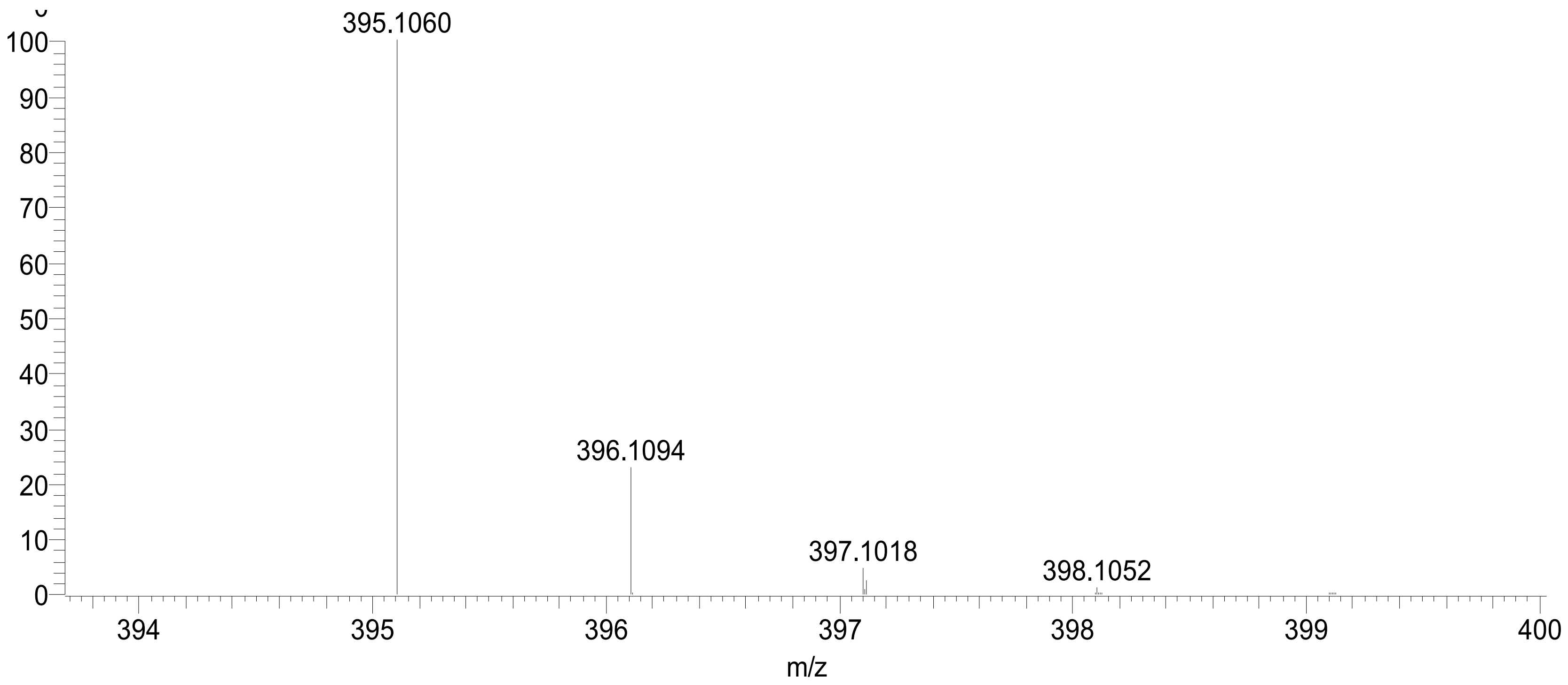

Figure S59: *Positive* HRMS spectrum of compound **6e**

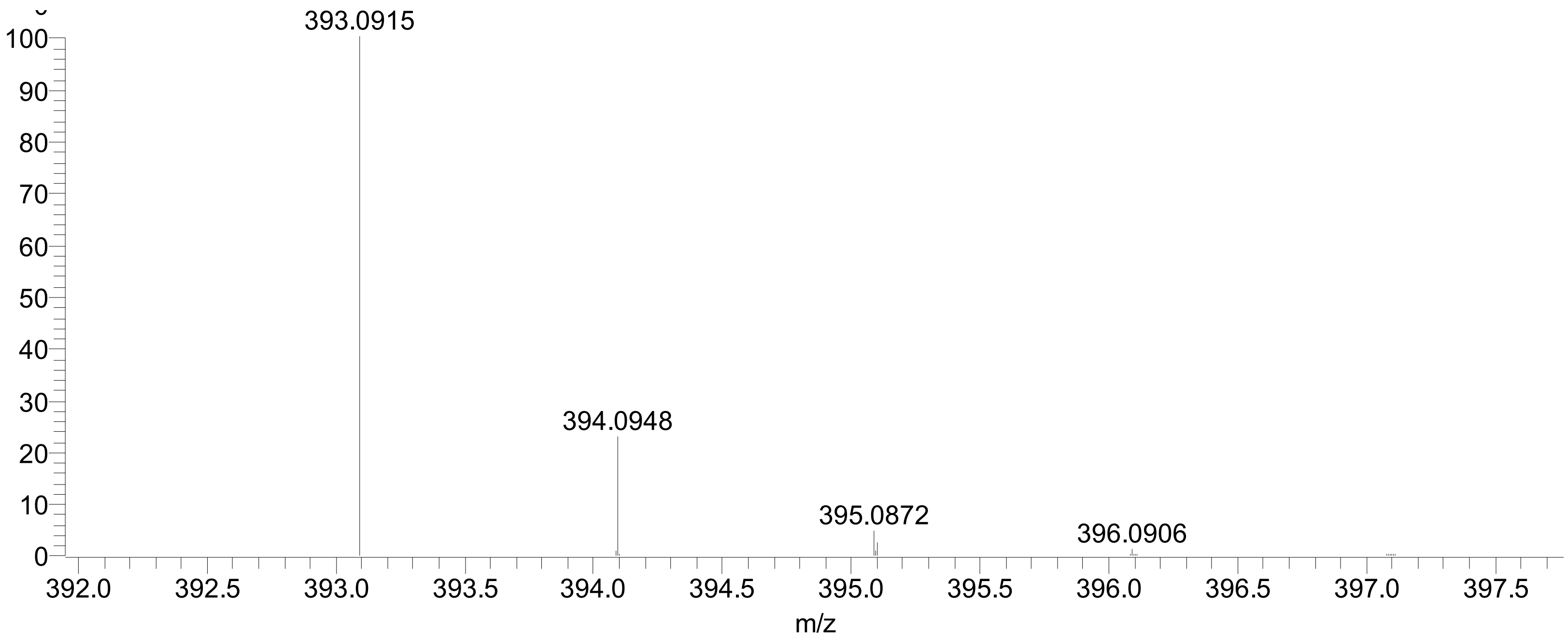

Figure S60: *Negative* HRMS spectrum of compound **6e**

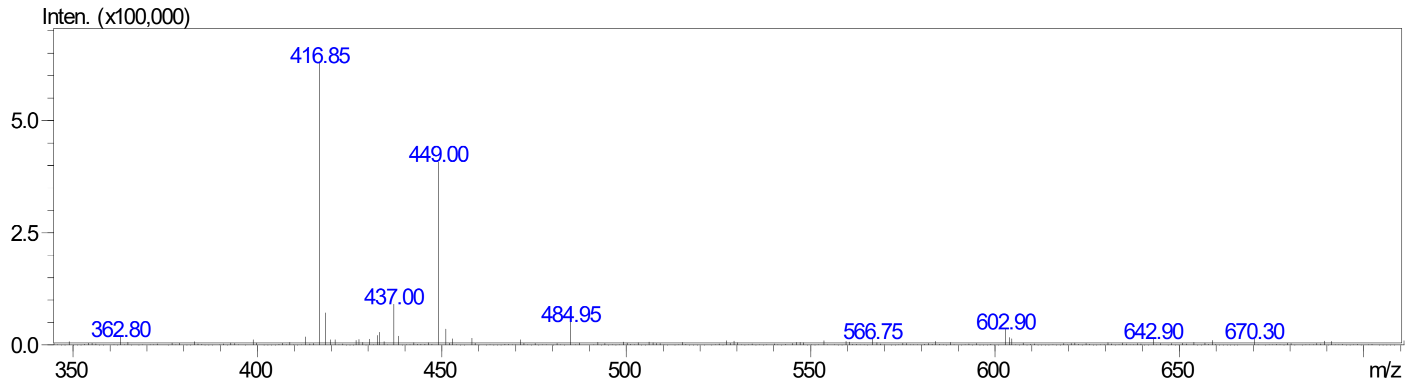

Figure S61: *Positive* LC-MS spectrum of compound **6e**

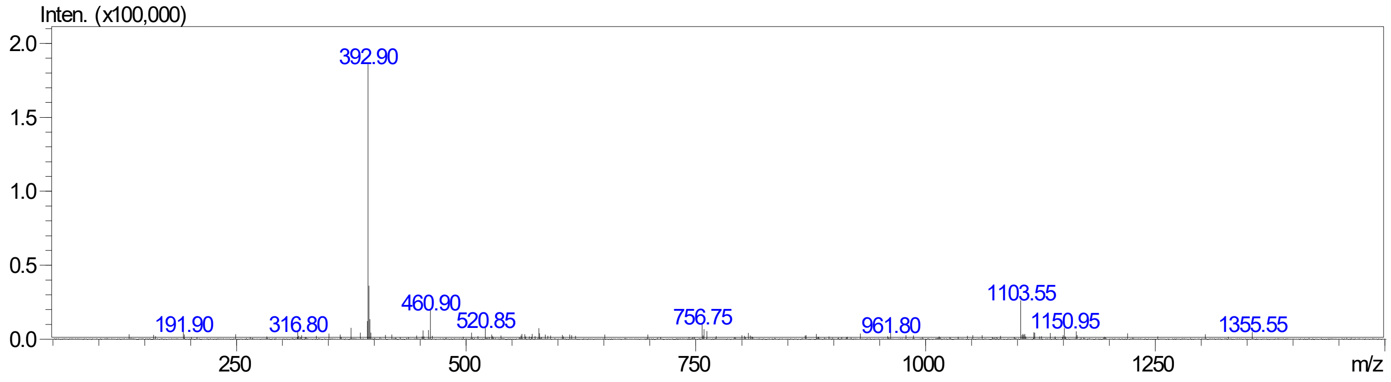

Figure S62: *Negative* LC-MS spectrum of compound **6e**

^{1}H NMR, ^{13}C NMR, HRMS and LCMS spectrums of compound **6f**

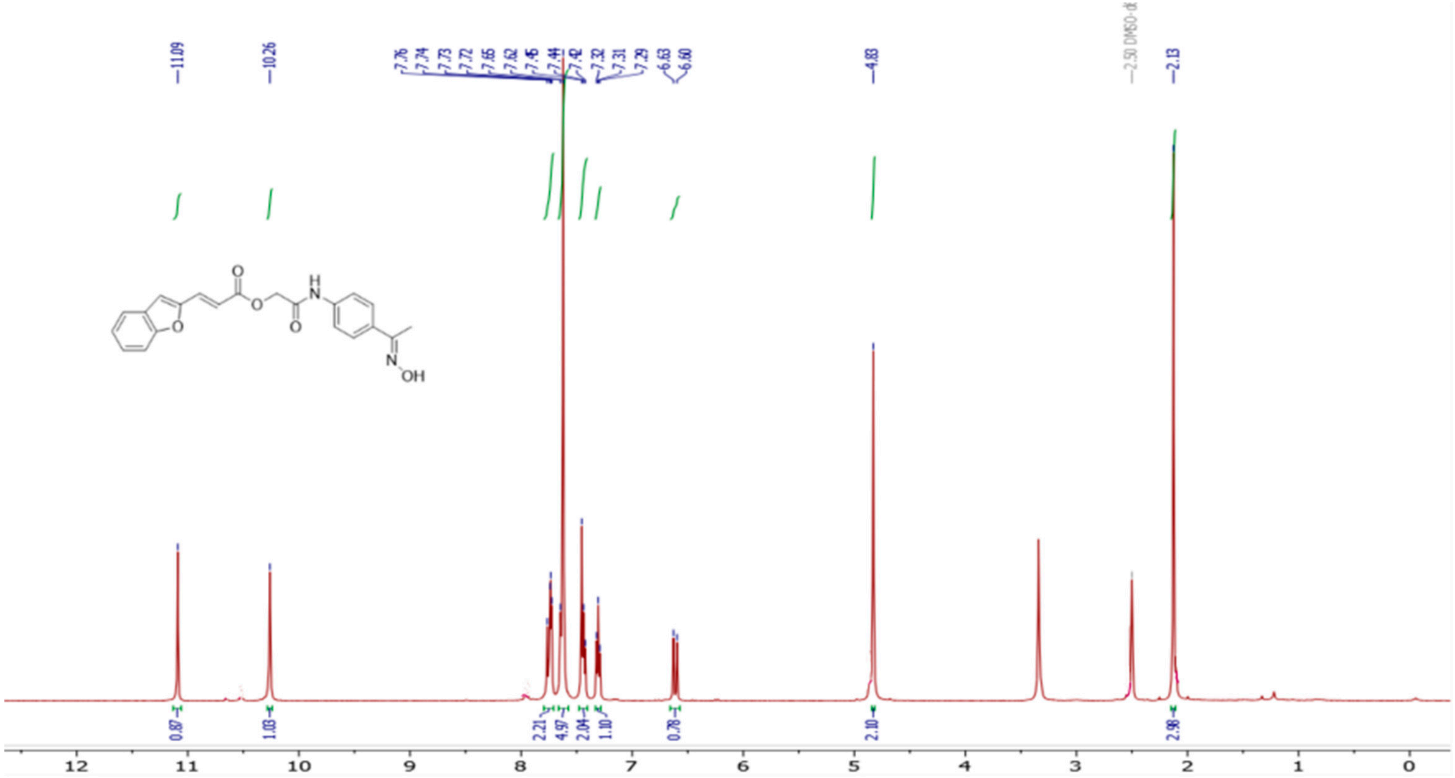

Figure S63: ^{1}H NMR spectrum of compound **6f**

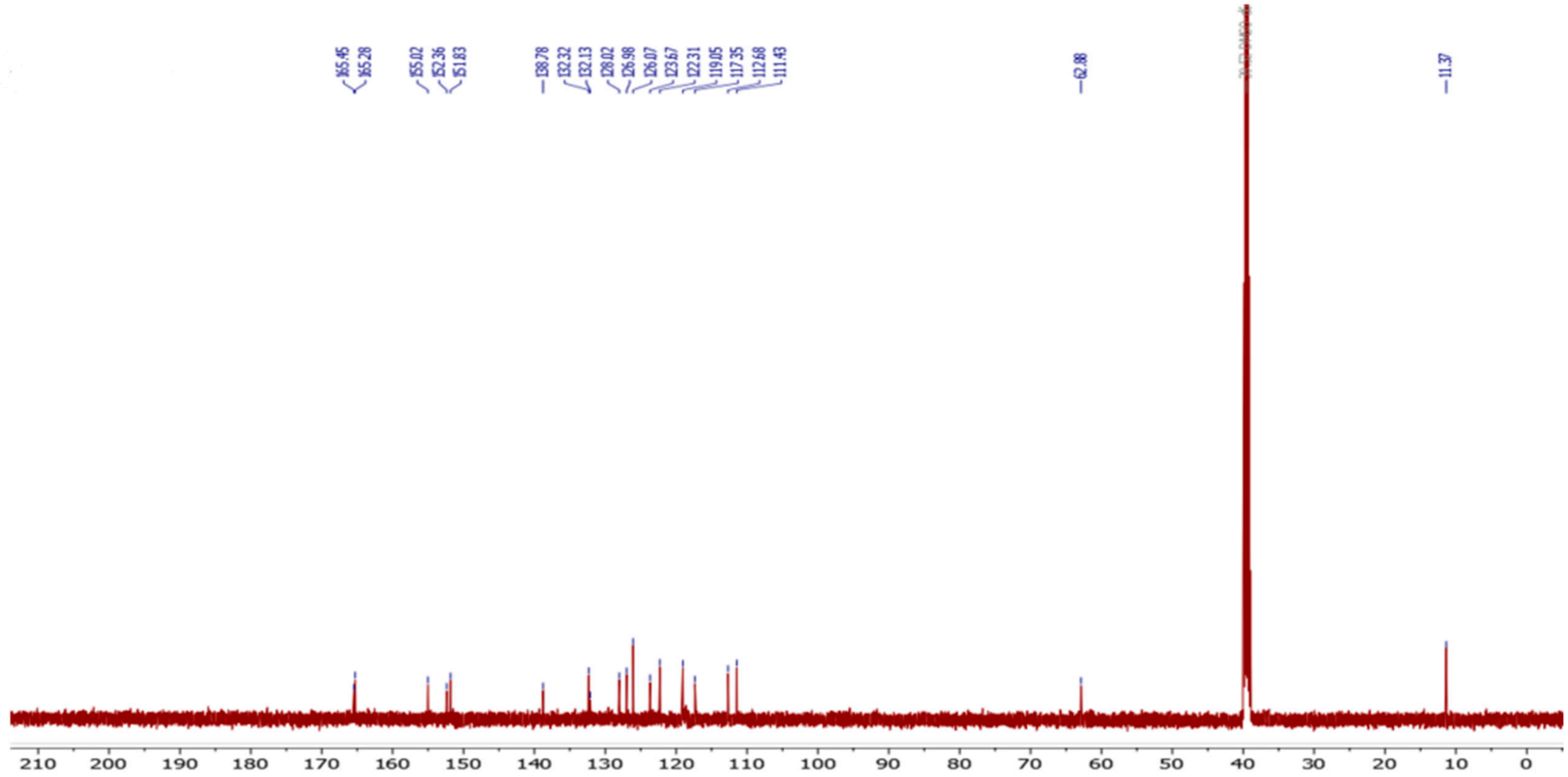

Figure S64: ^{13}C NMR spectrum of compound **6f**

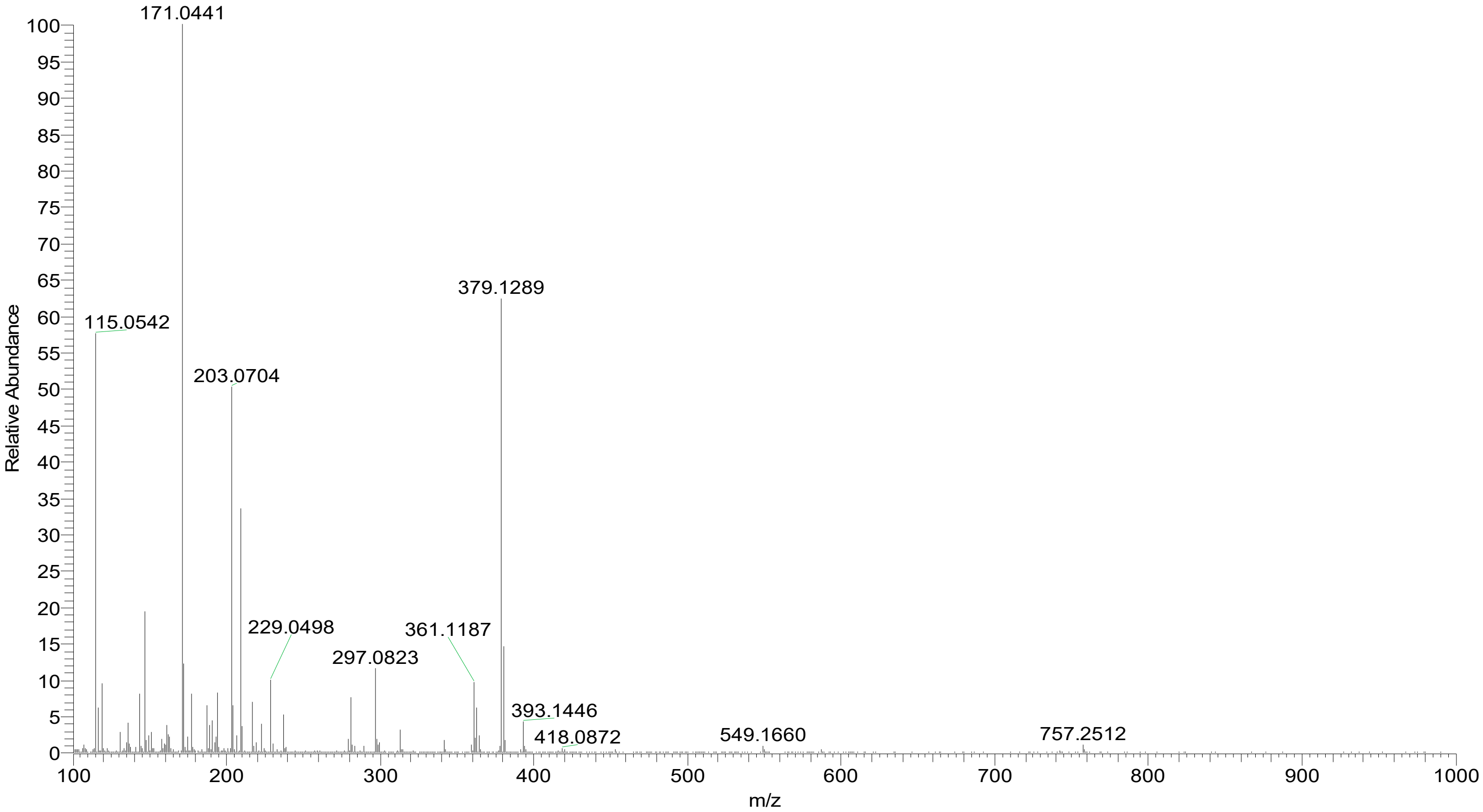

Figure S65: *Positive* HRMS spectrum of compound **6f**

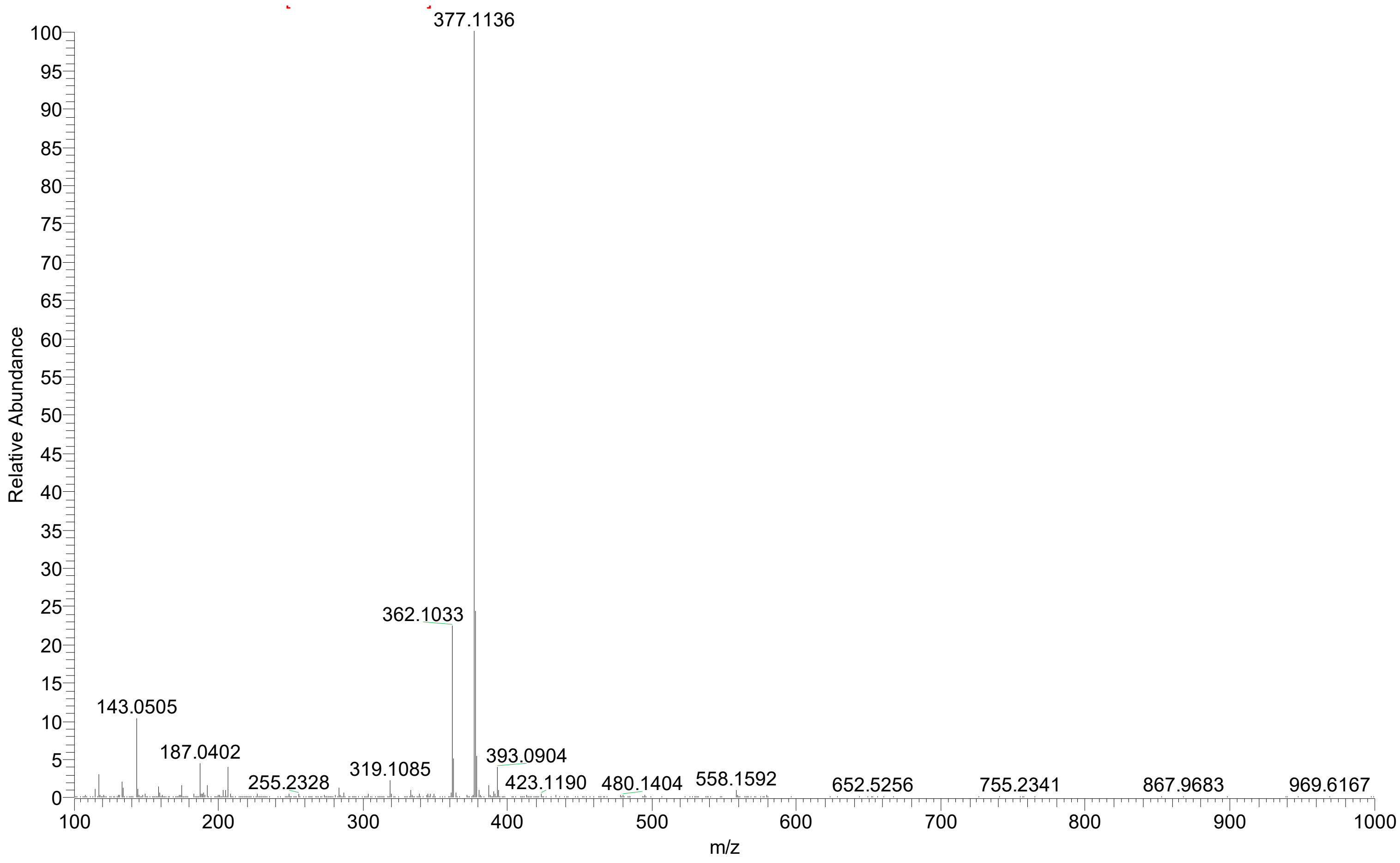

Figure S66: *Negative* HRMS spectrum of compound **6f**

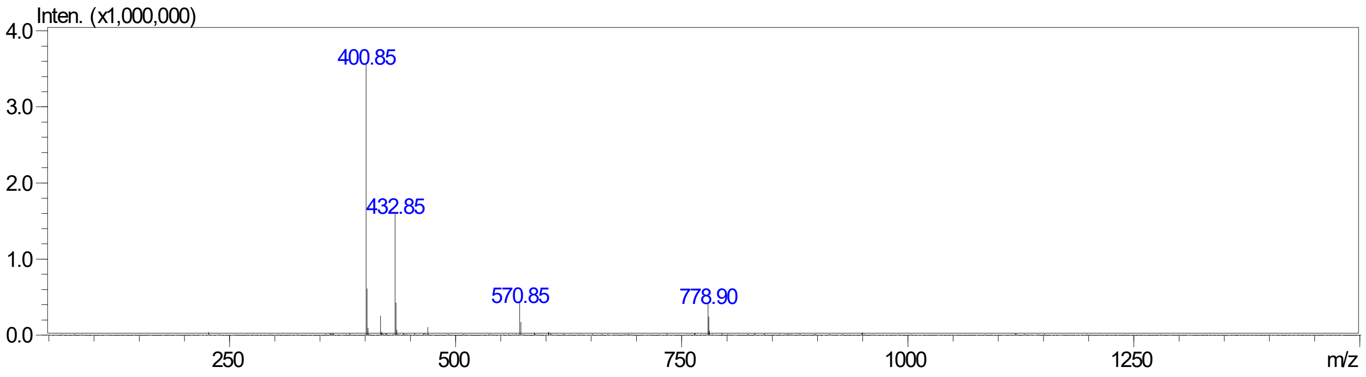

Figure S67: *Positive* LC-MS spectrum of compound **6f**

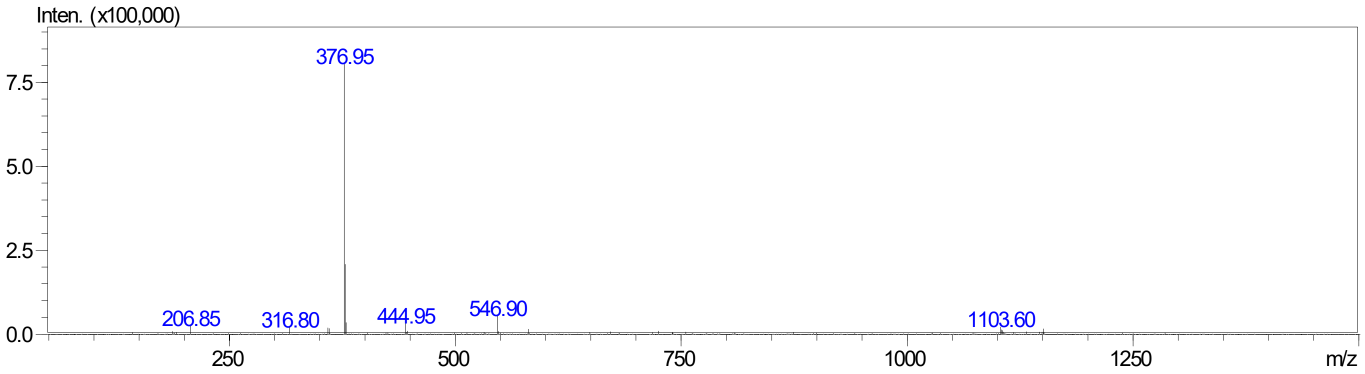

Figure S68: *Negative* LC-MS spectrum of compound **6f**

^{1}H NMR, ^{13}C NMR, HRMS and LCMS spectrums of compound **6g**

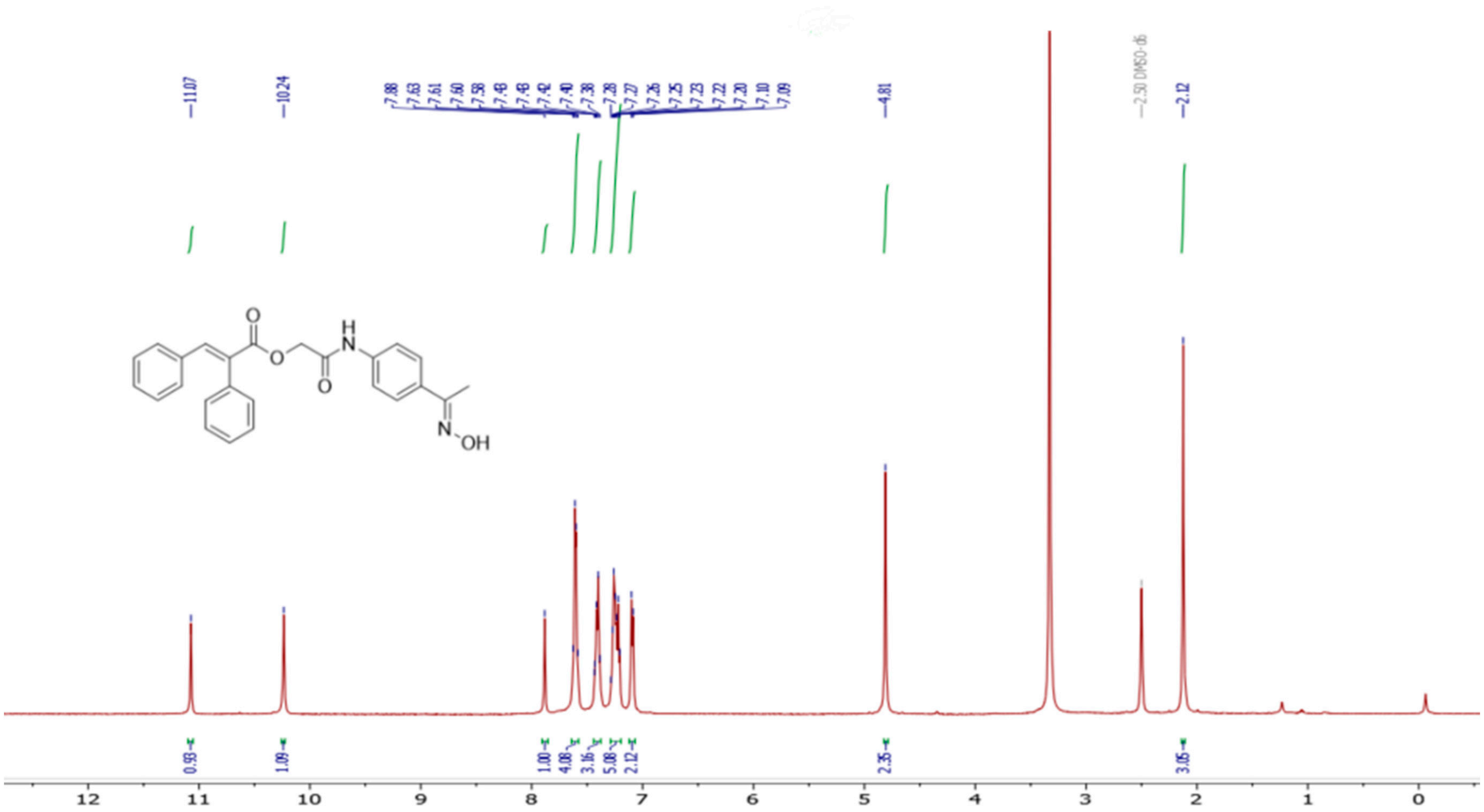

Figure S69: ^{1}H NMR spectrum of compound **6g**

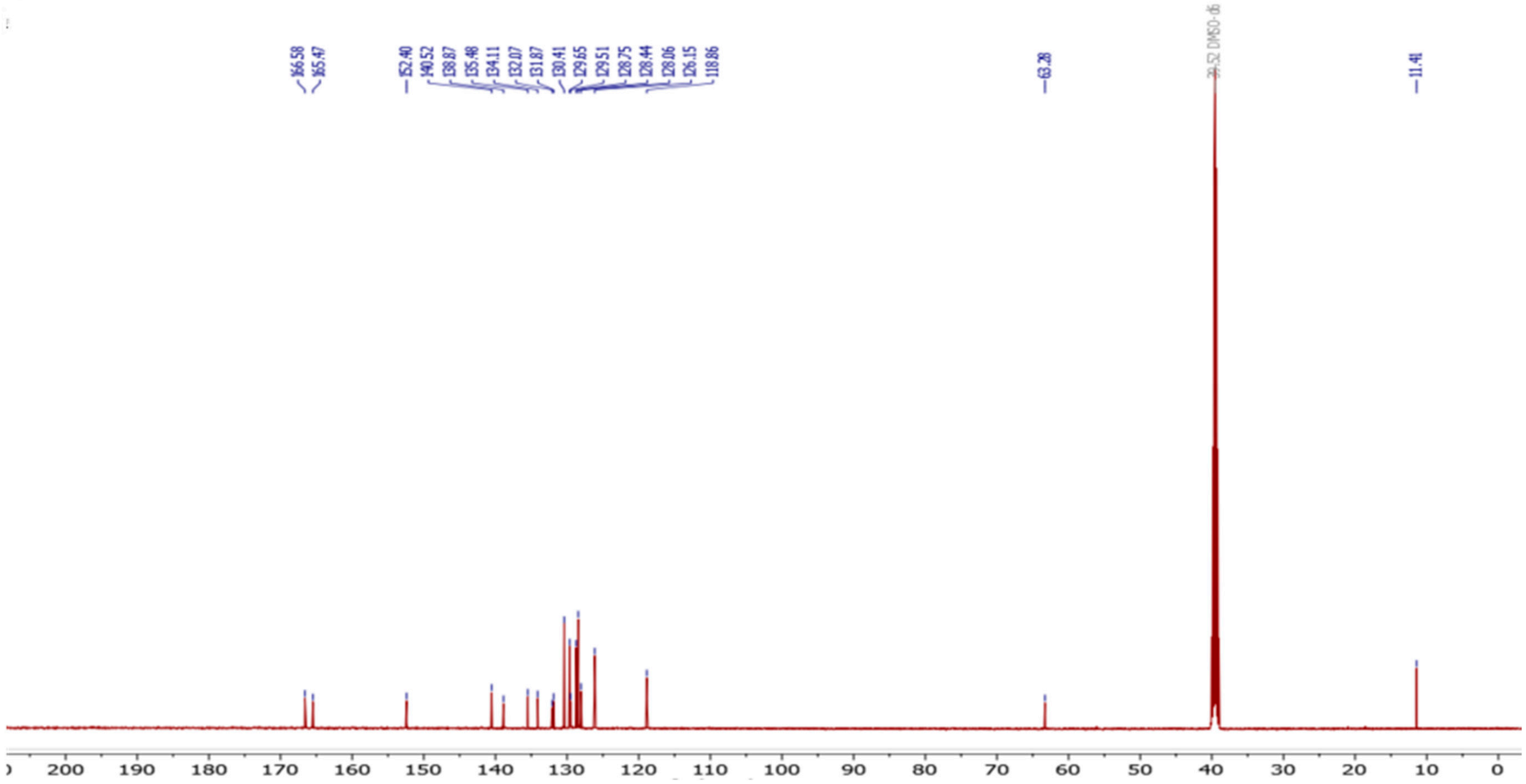

Figure S70: ^{13}C NMR spectrum of compound **6g**

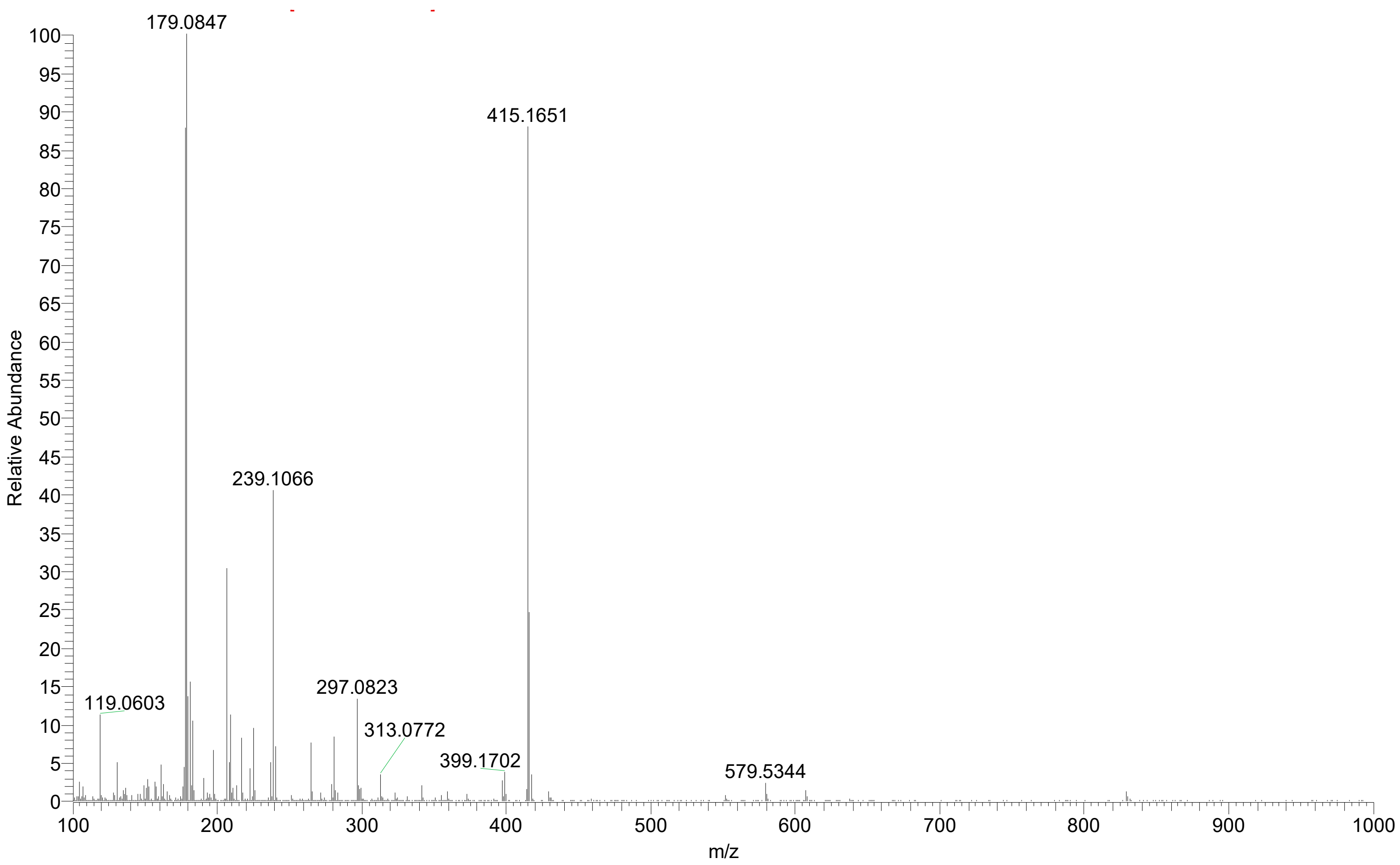

Figure S71: *Positive* HRMS spectrum of compound **6g**

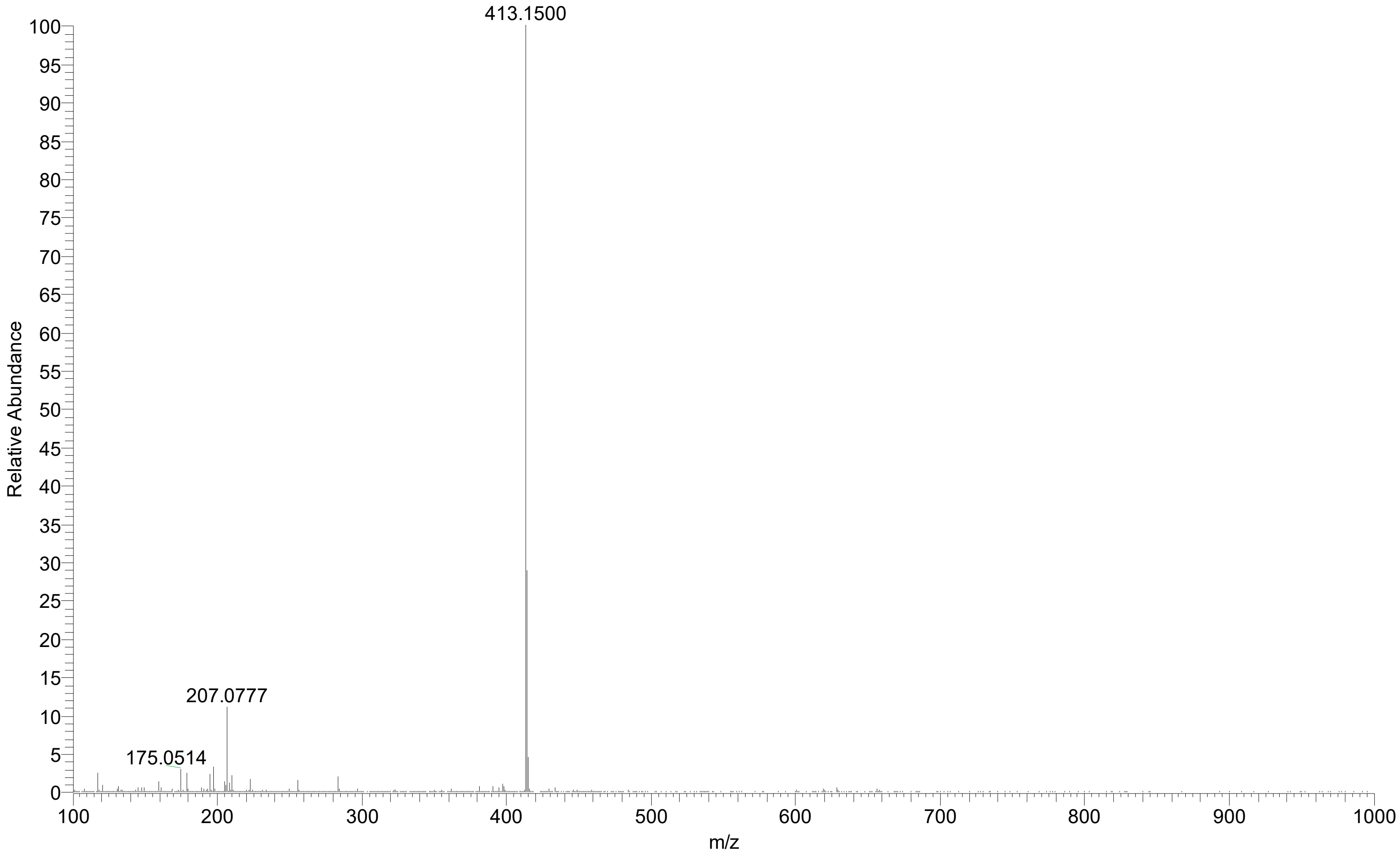

Figure S72: *Negative* HRMS spectrum of compound **6g**

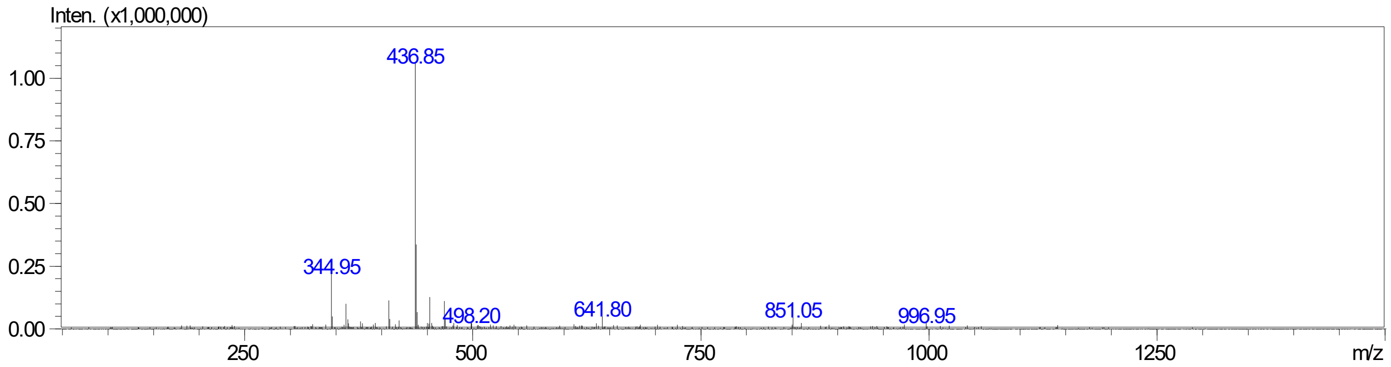

Figure S73: *Positive* LC-MS spectrum of compound **6g**

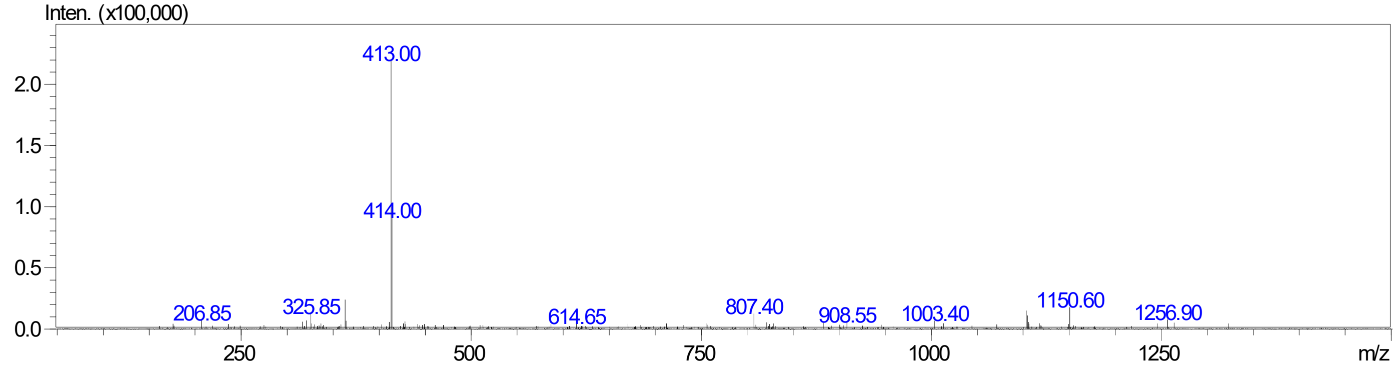

Figure S74: *Negative* LC-MS spectrum of compound **6g**

^{1}H NMR, ^{13}C NMR, HRMS and LCMS spectrums of compound **6h**

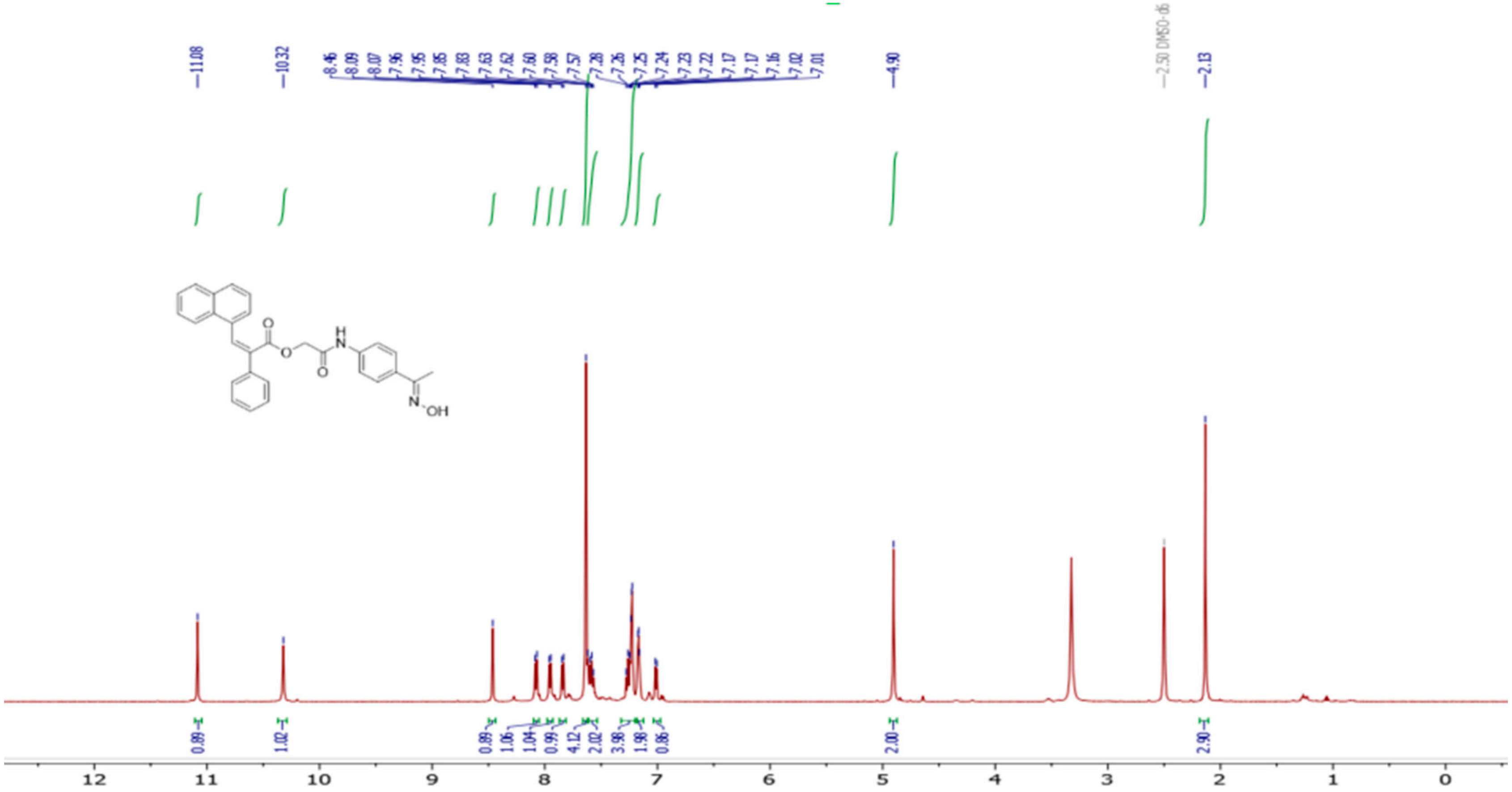

Figure S75: ^{1}H NMR spectrum of compound **6h**

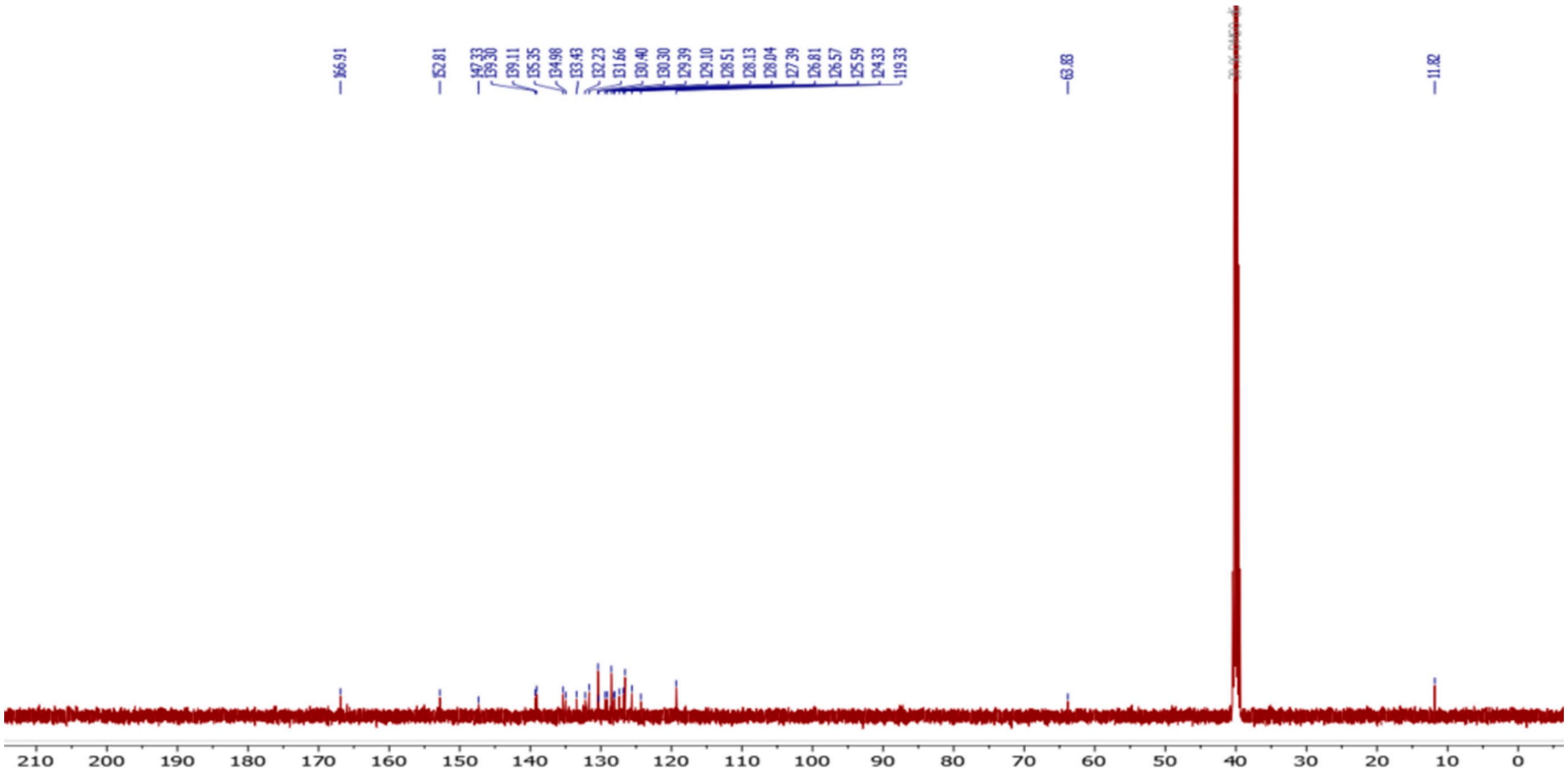

Figure S76: ^{13}C NMR spectrum of compound **6h**

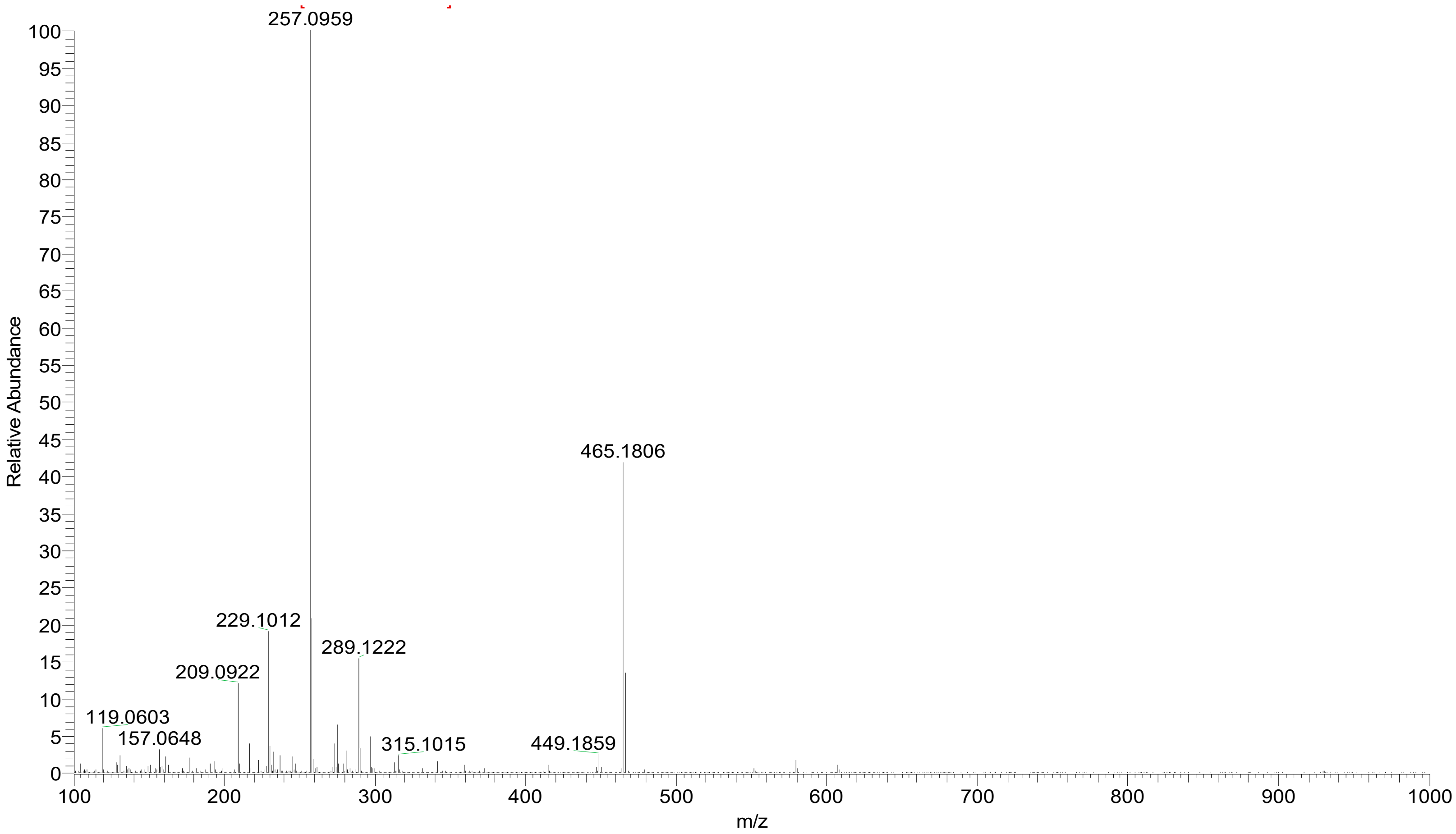

Figure S77: *Positive* HRMS spectrum of compound **6h**

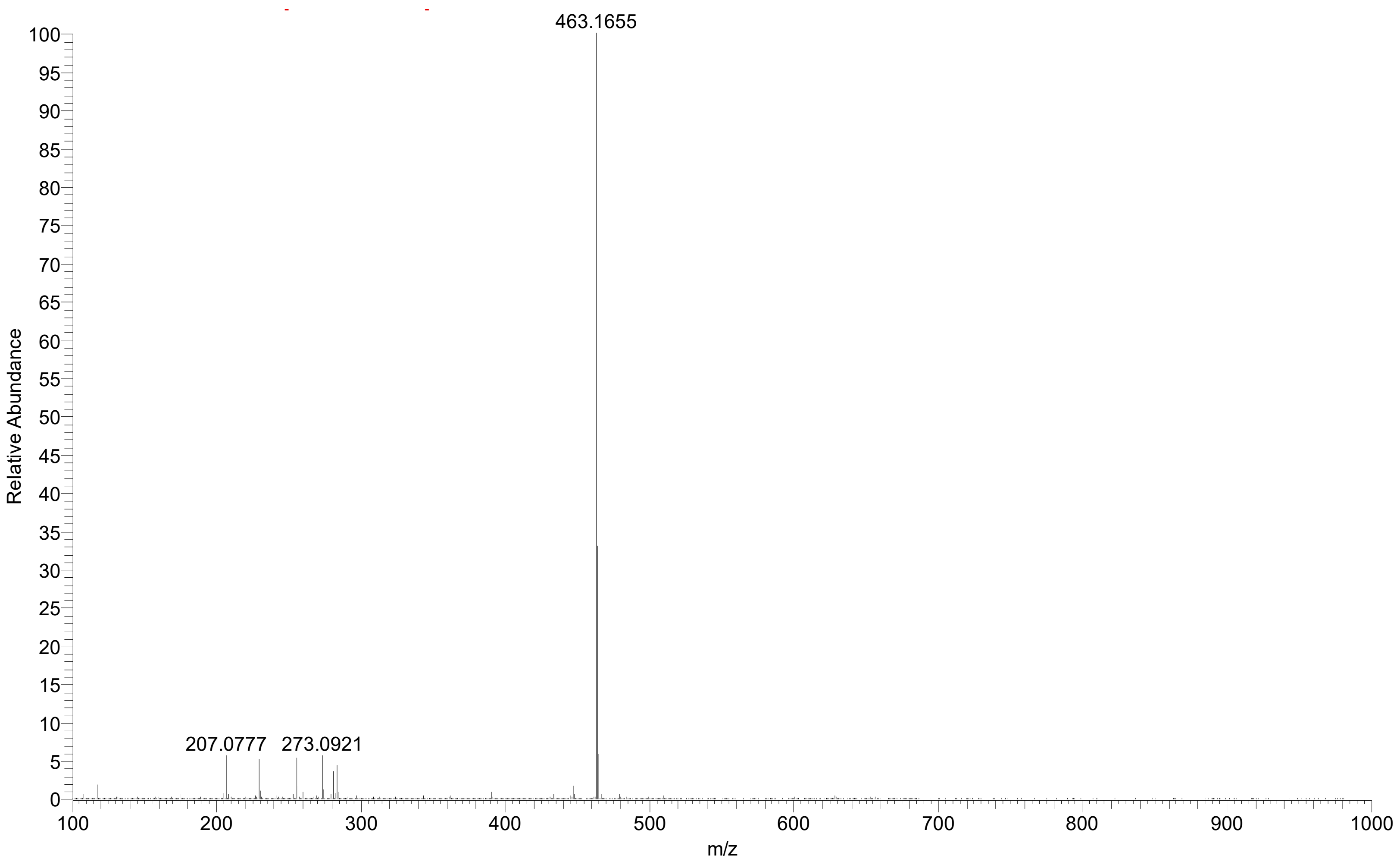

Figure S78: *Negative* HRMS spectrum of compound **6h**

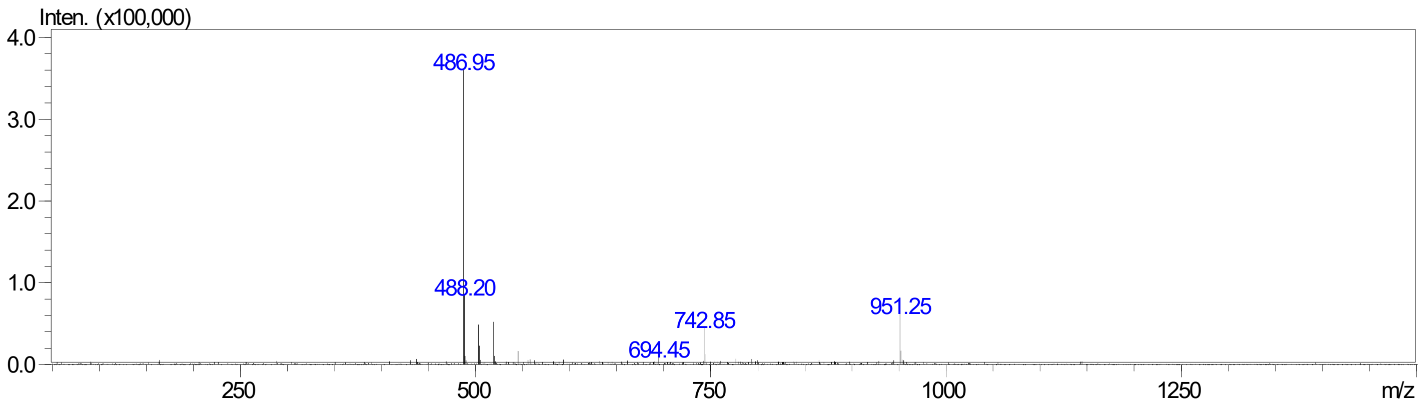

Figure S79: *Positive* LC-MS spectrum of compound **6h**

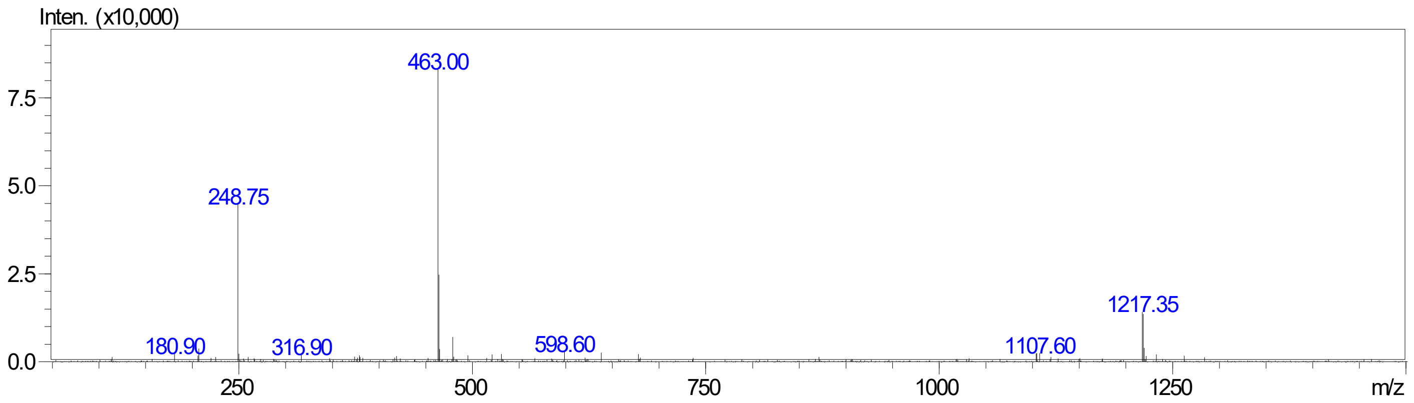

Figure S80: *Negative* LC-MS spectrum of compound **6h**

^{1}H NMR, ^{13}C NMR, HRMS and LCMS spectrums of compound **6i**

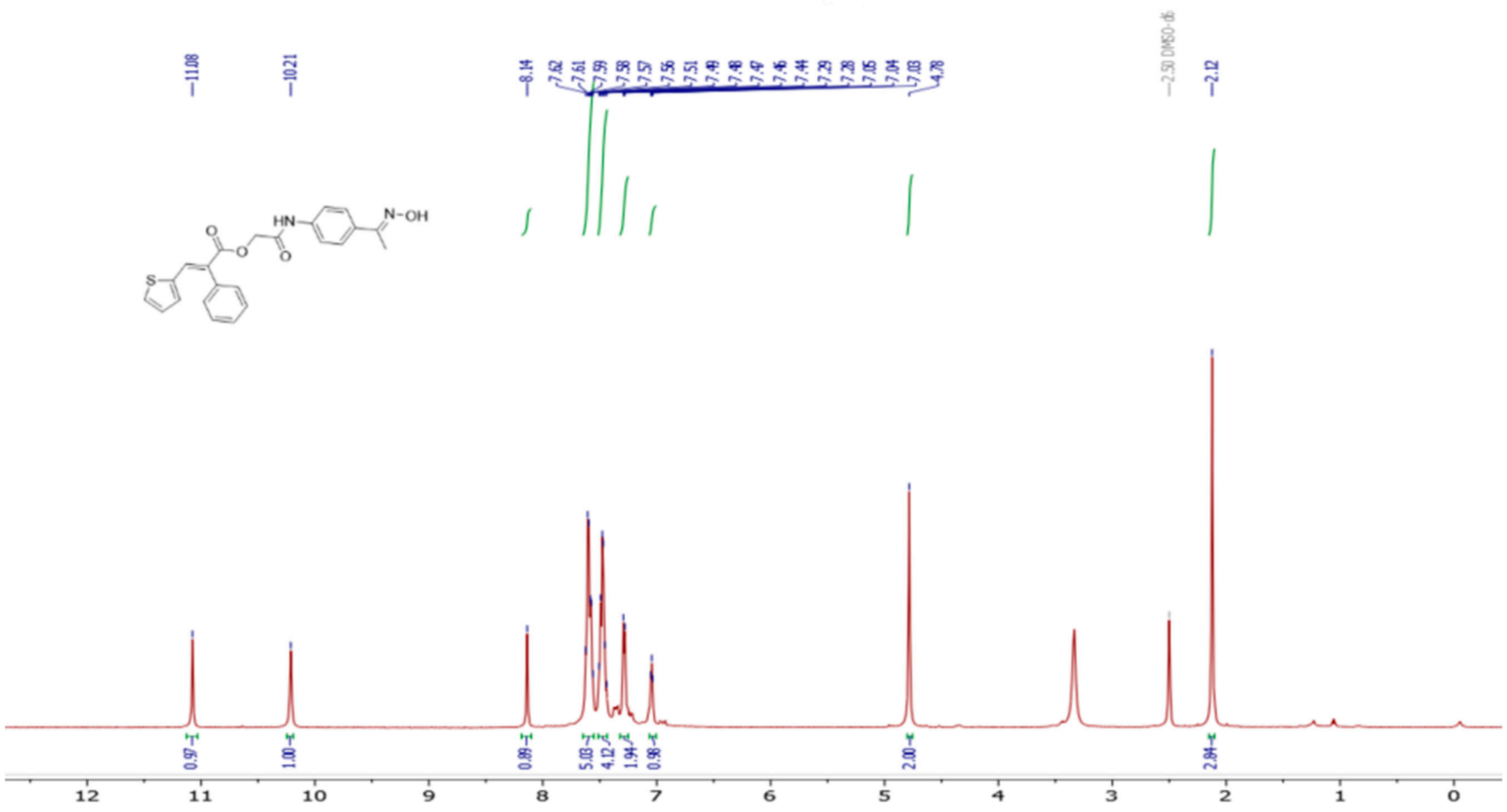

Figure S81: ^{1}H NMR spectrum of compound **6i**

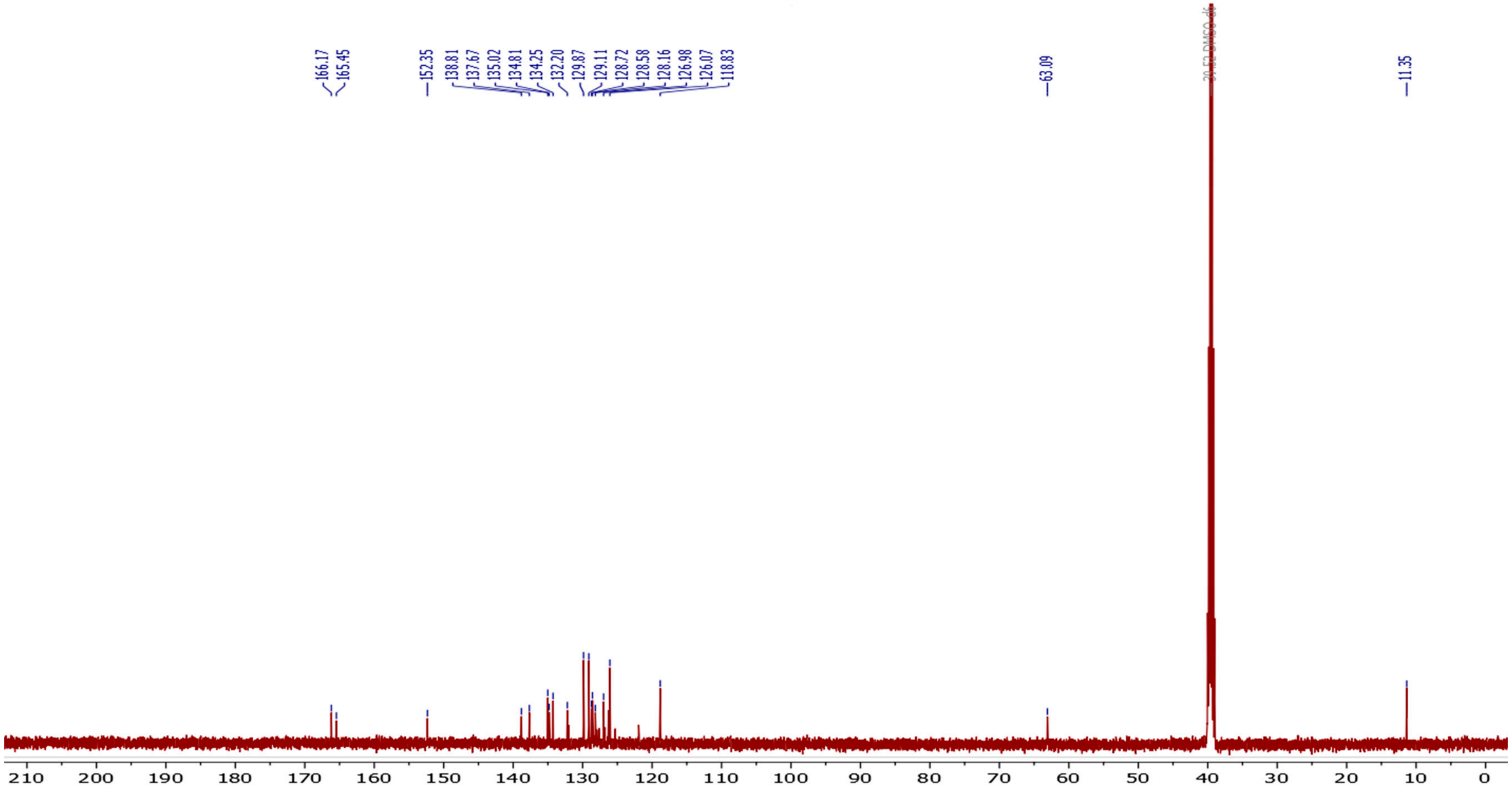

Figure S82: ^{13}C NMR spectrum of compound **6i**

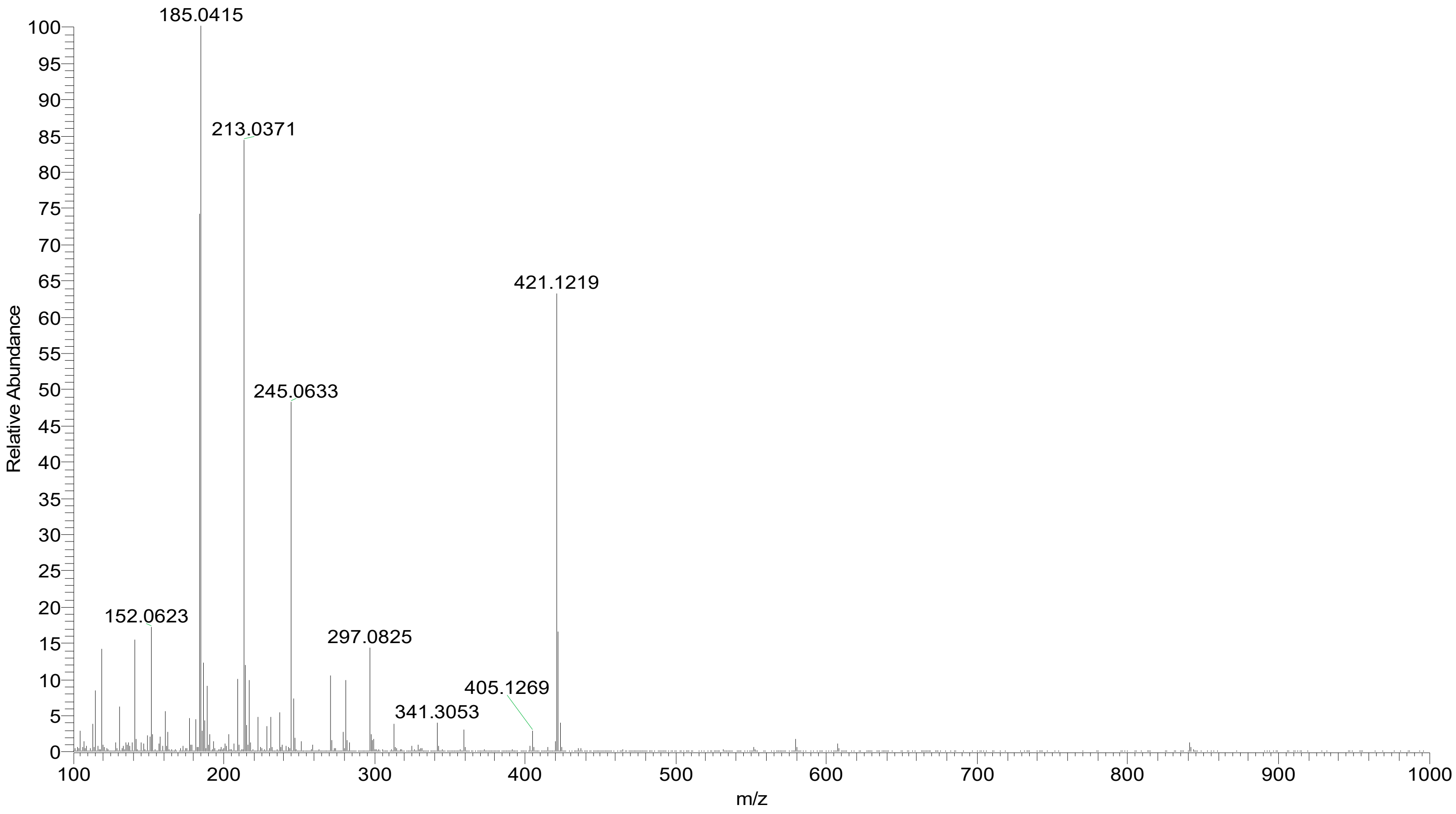

Figure S83: *Positive* HRMS spectrum of compound **6i**

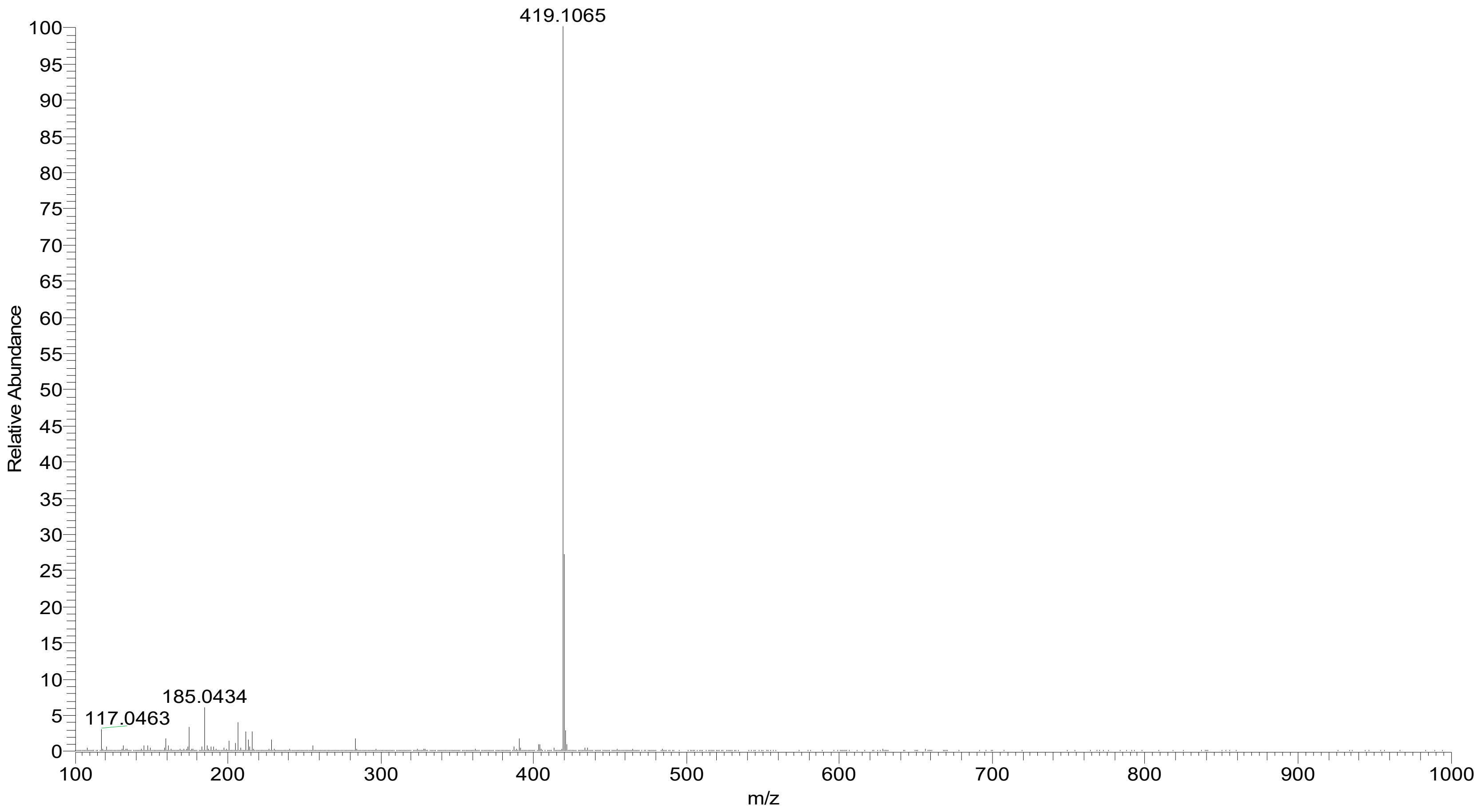

Figure S84: *Negative* HRMS spectrum of compound **6i**

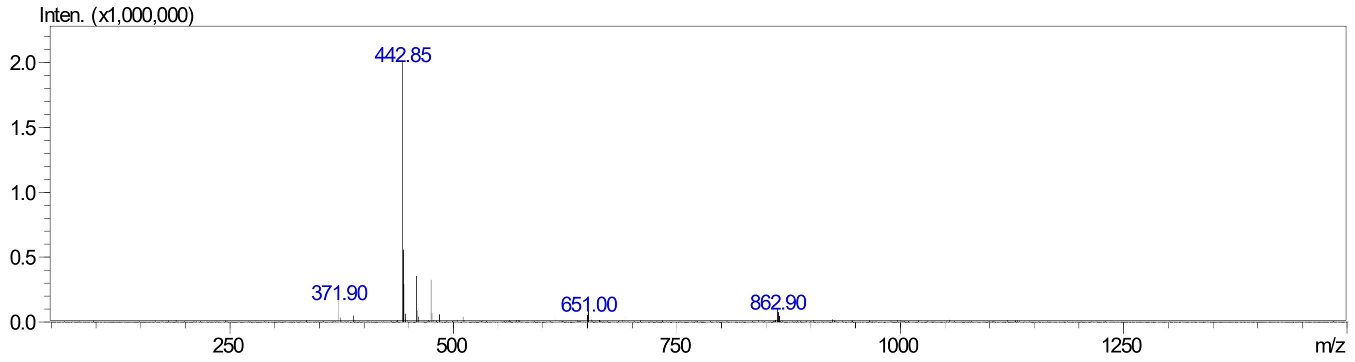

Figure S85: *Positive* LC-MS spectrum of compound **6i**

^{1}H NMR, ^{13}C NMR and LCMS spectrums of compound **9a**

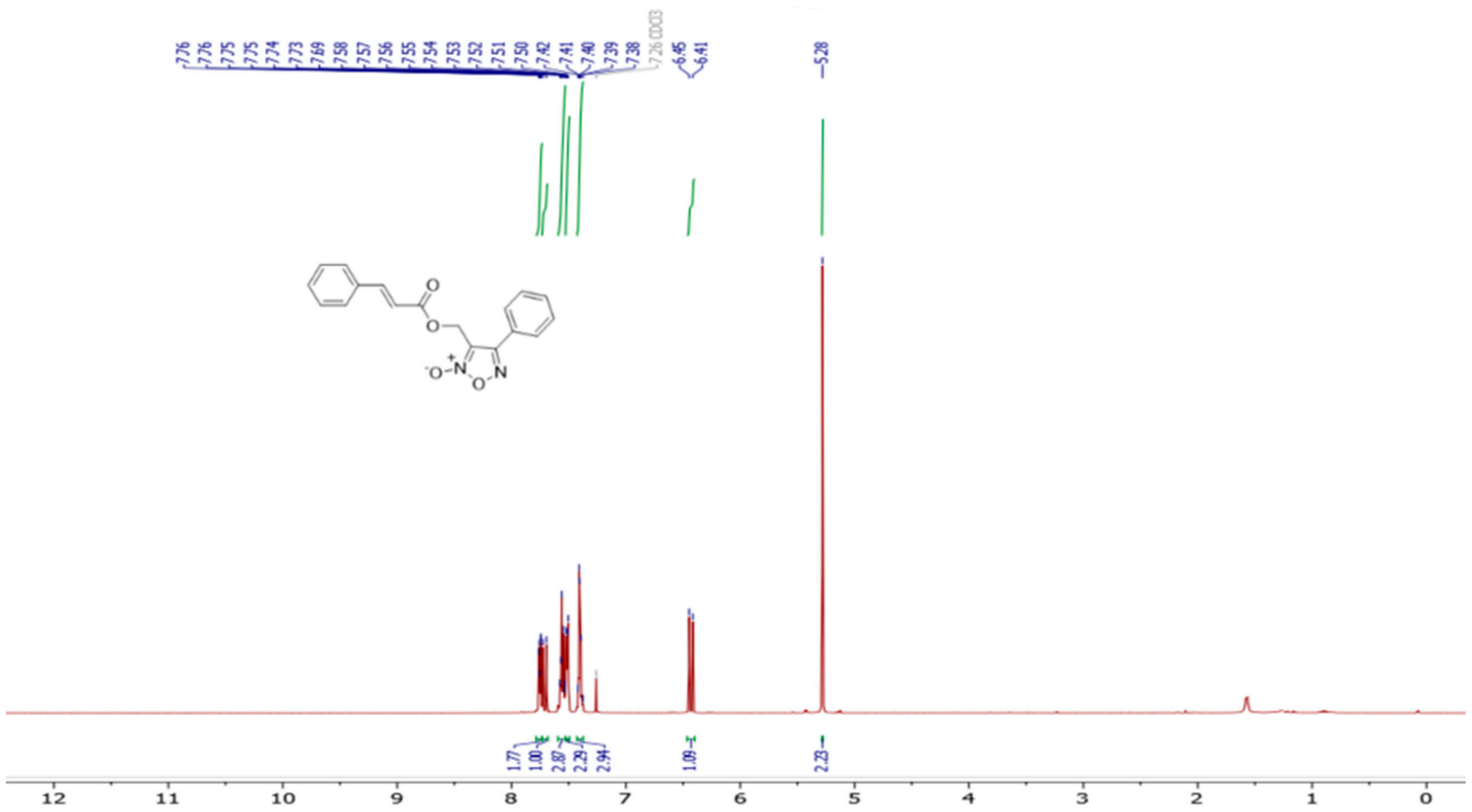

Figure S86: ^{1}H NMR spectrum of compound **9a**

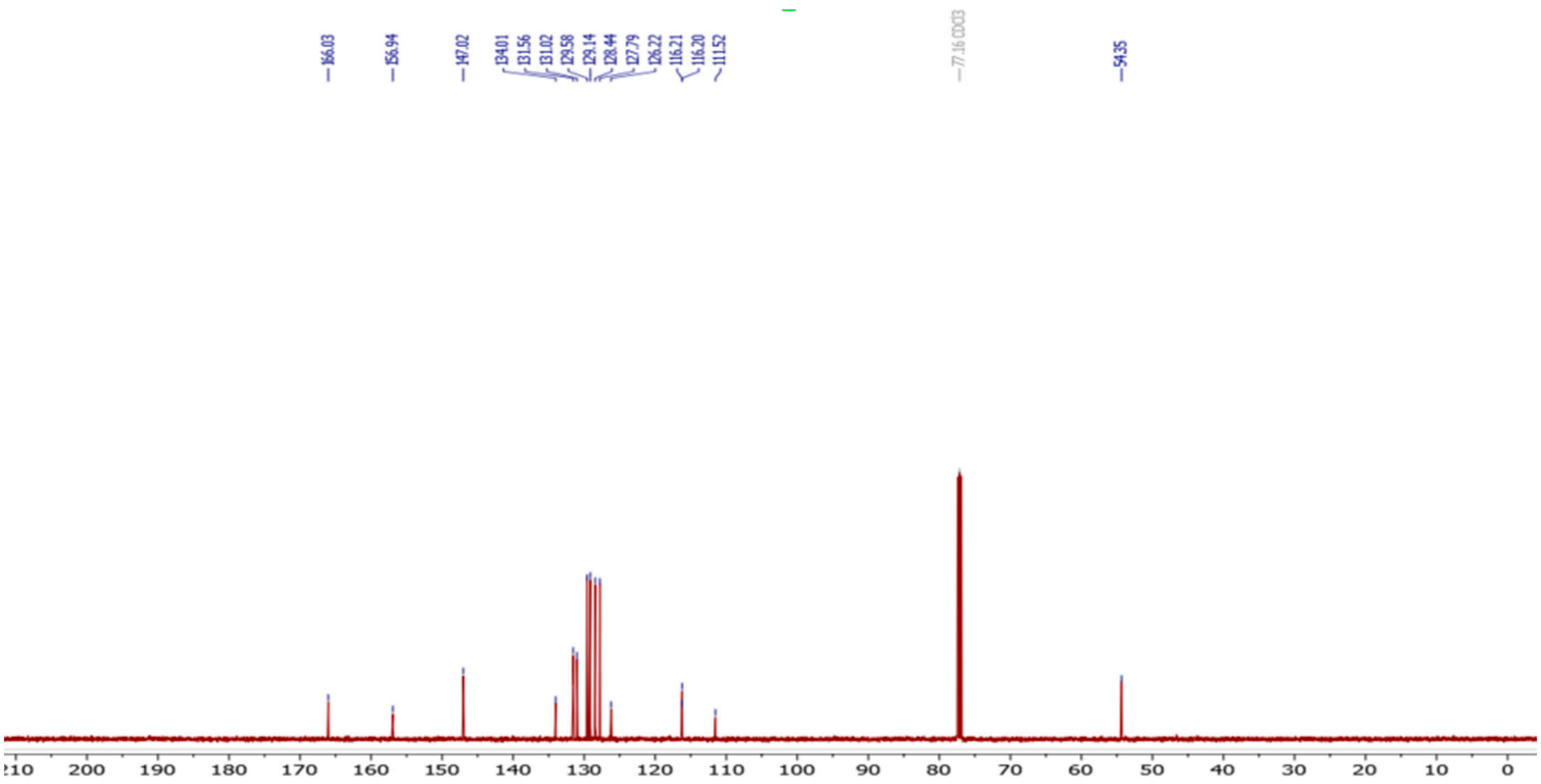

Figure S87: ^{13}C NMR spectrum of compound **9a**

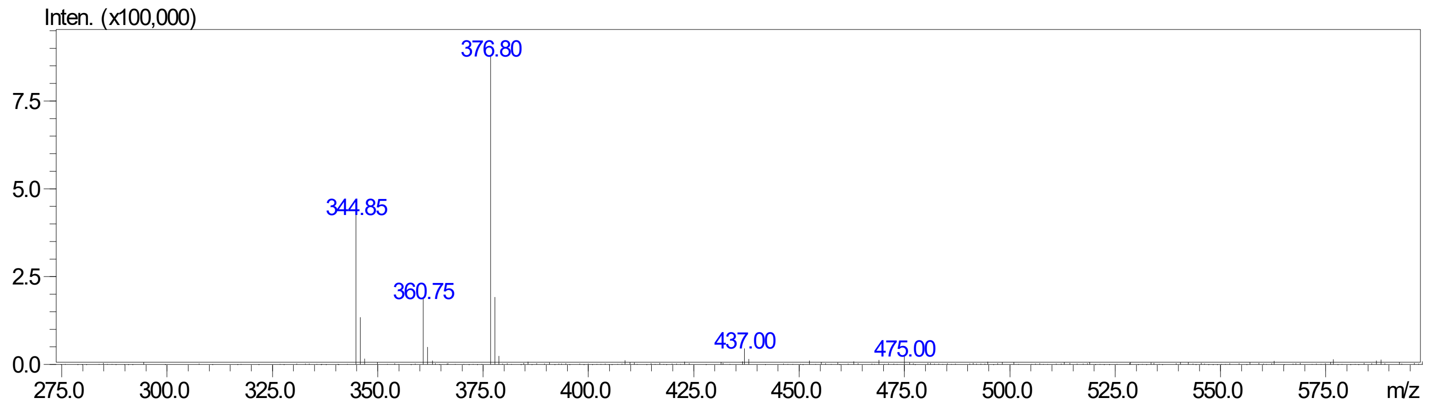

Figure S88: *Positive* LC-MS spectrum of compound **9a**

^{1}H NMR, ^{13}C NMR, HRMS and LCMS spectrums of compound **9b**

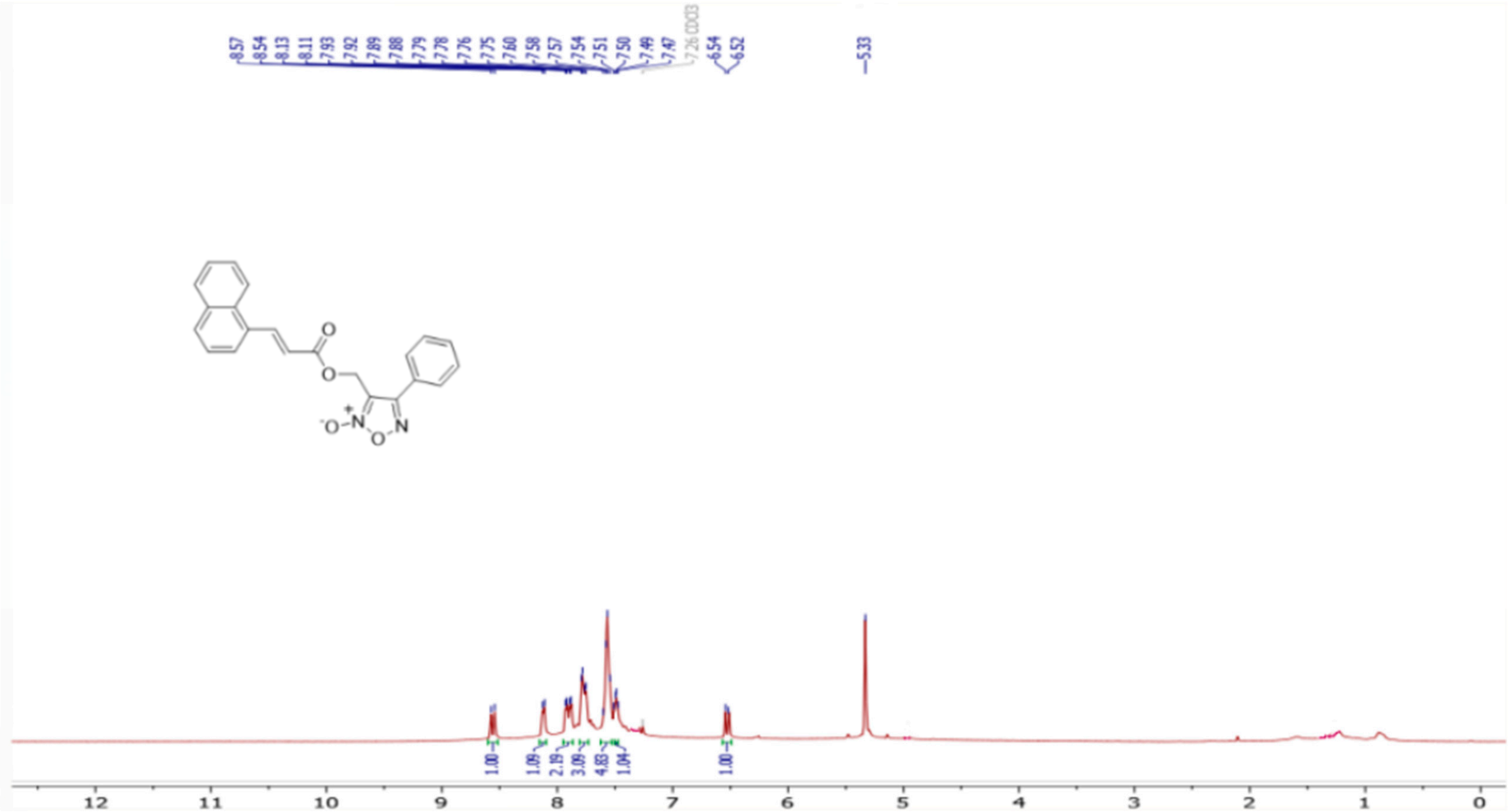

Figure S89: ^{1}H NMR spectrum of compound **9b**

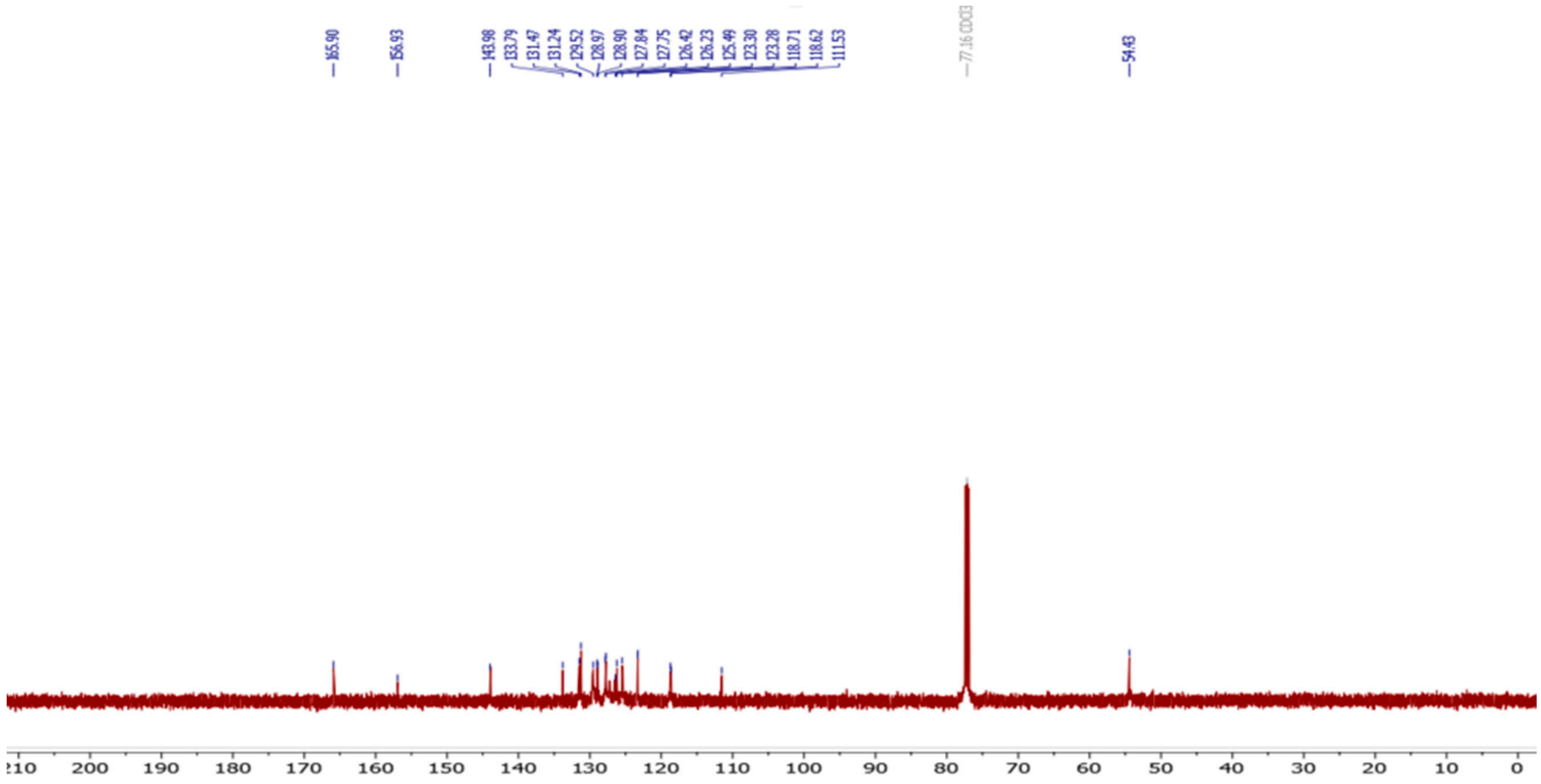

Figure S90: ^{13}C NMR spectrum of compound **9b**

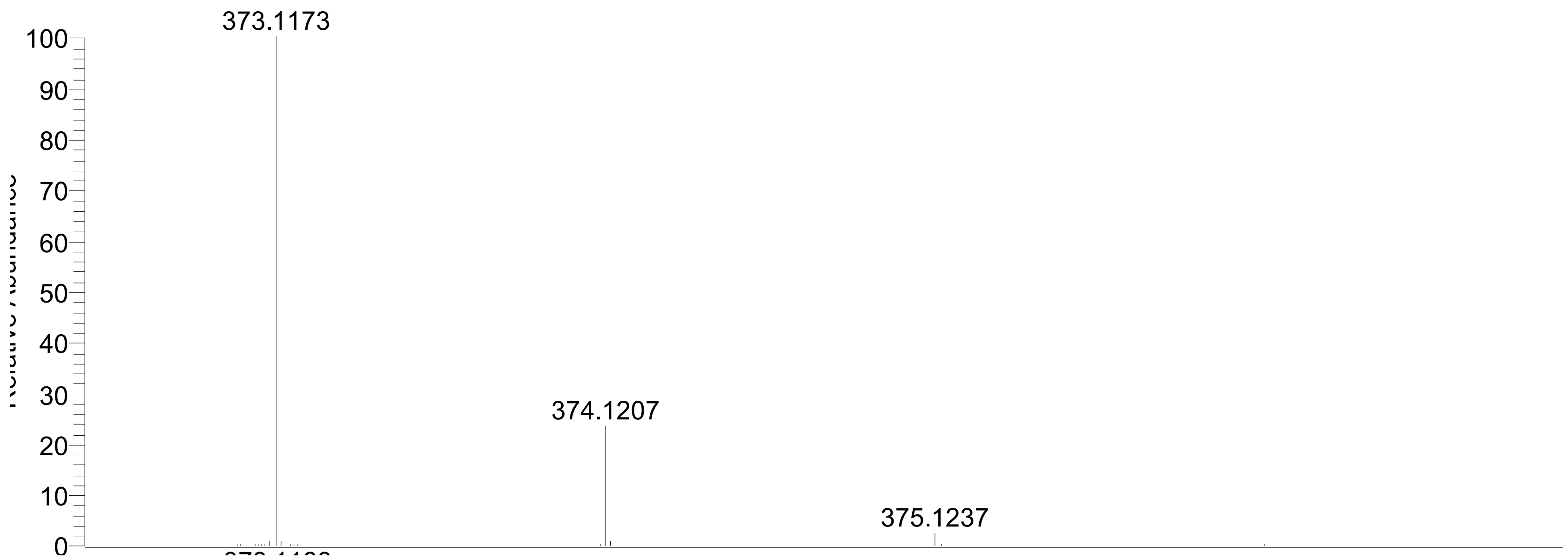

Figure S91: *Positive* HRMS spectrum of compound **9b**

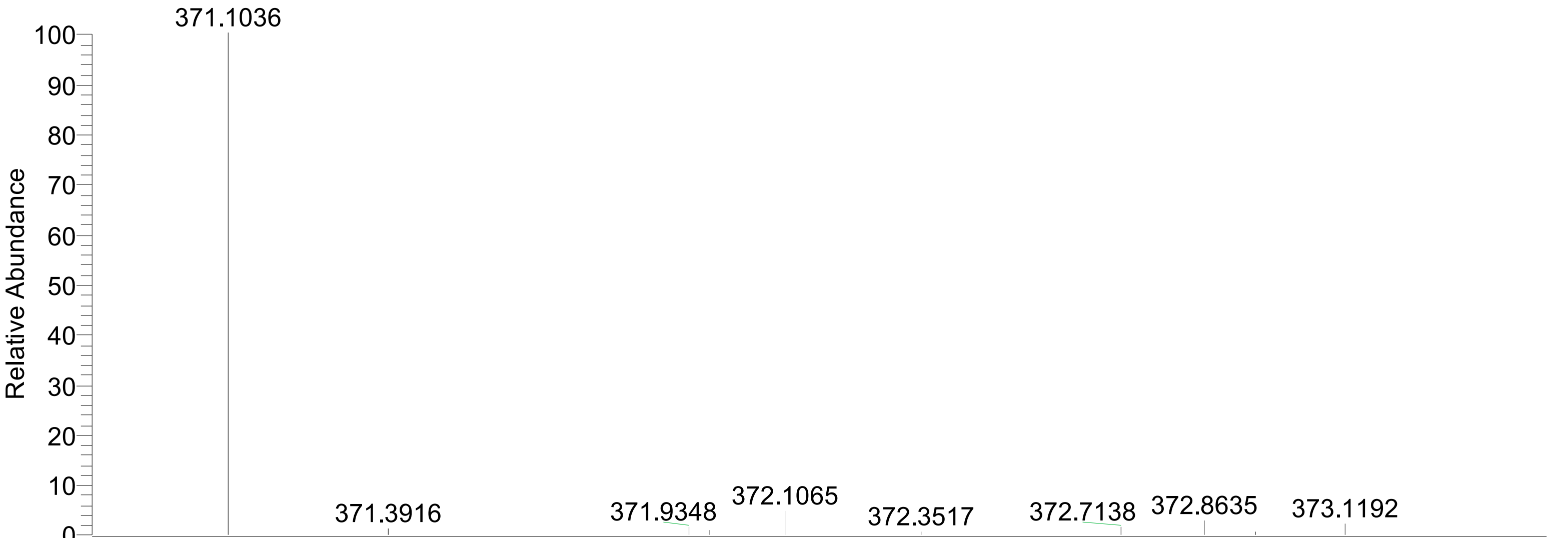

Figure S92: *Negative* HRMS spectrum of compound **9b**

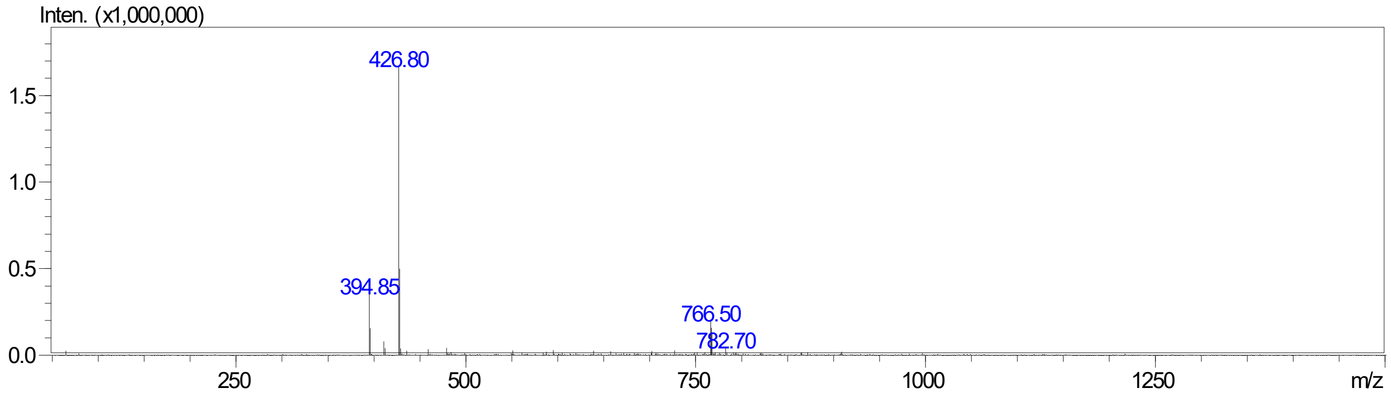

Figure S93: *Positive* LC-MS spectrum of compound **9b**

^{1}H NMR, ^{13}C NMR, HRMS and LCMS spectrums of compound **9c**

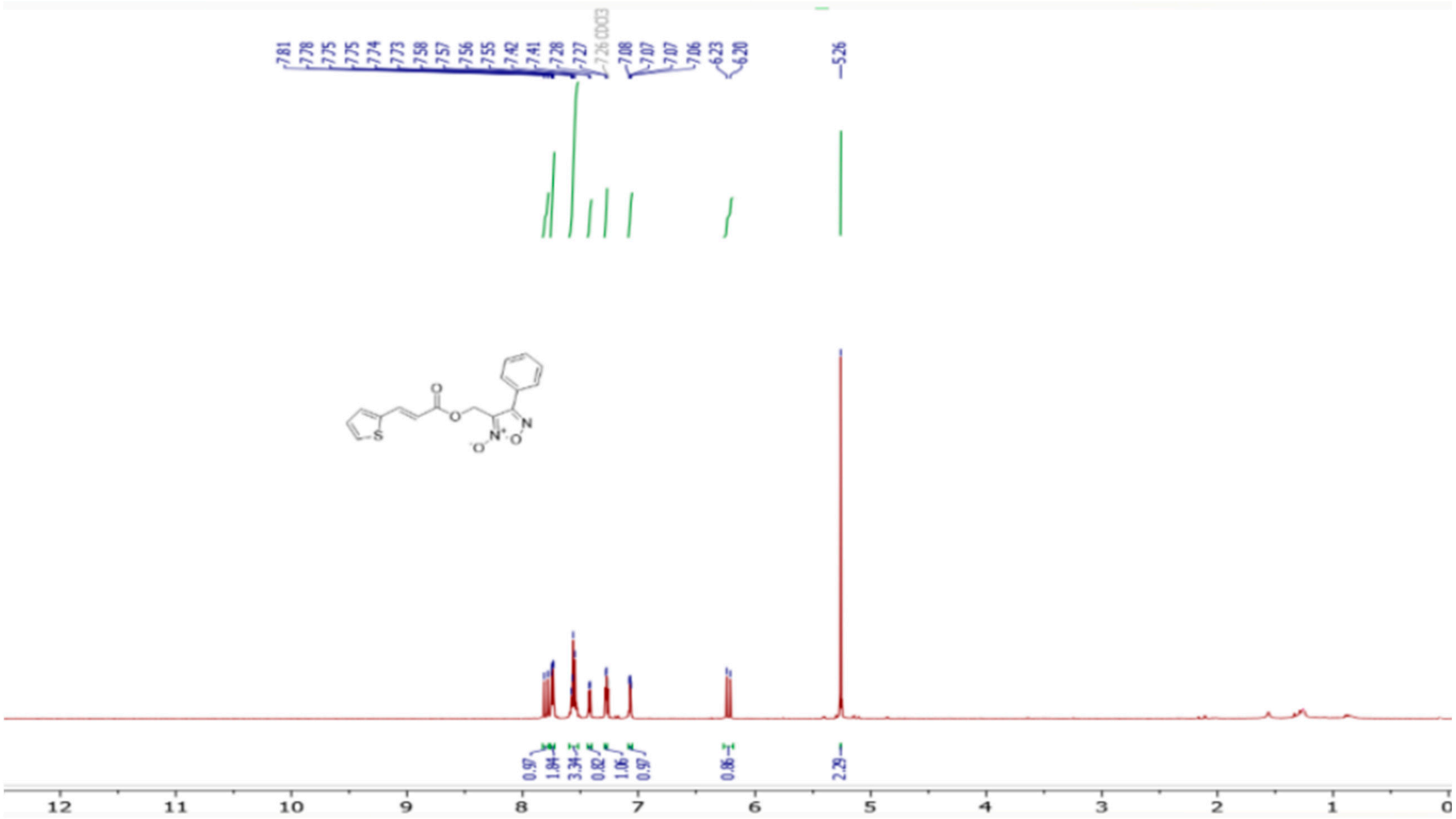

Figure S94: ^{1}H NMR spectrum of compound **9c**

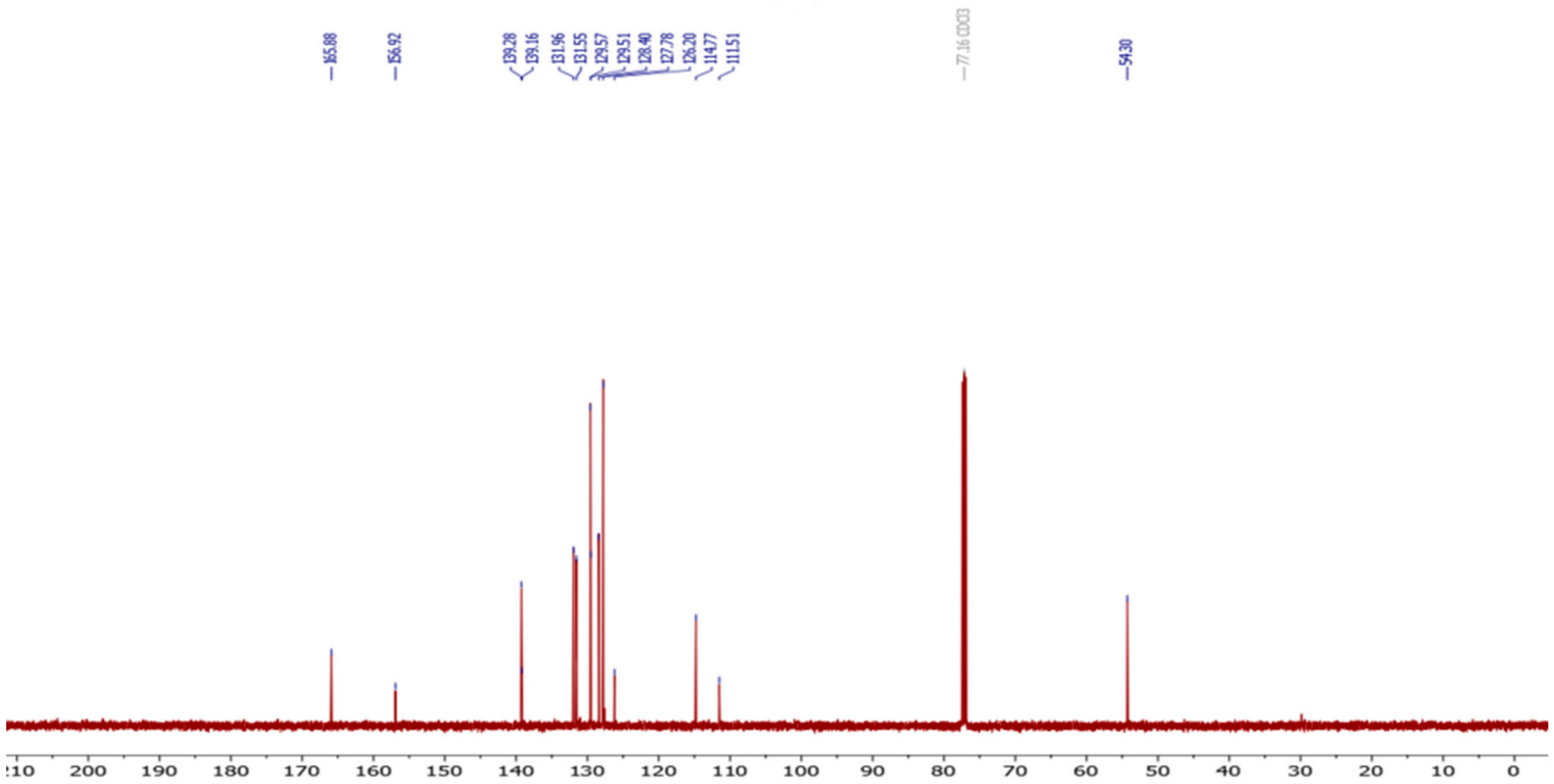

Figure S95: ^{13}C NMR spectrum of compound **9c**

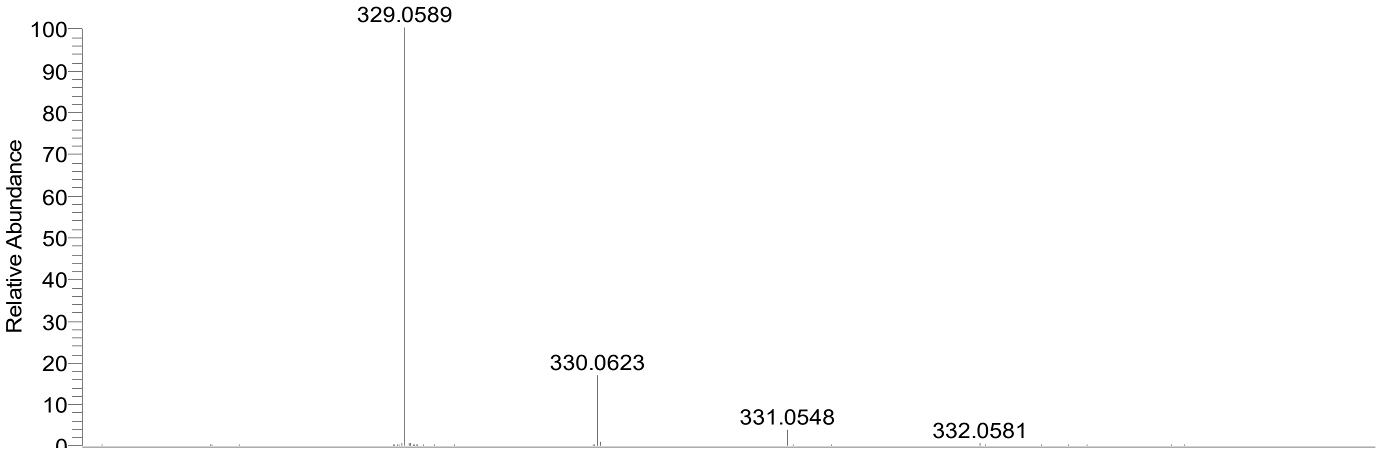

Figure S96: *Positive* HRMS spectrum of compound **9c**

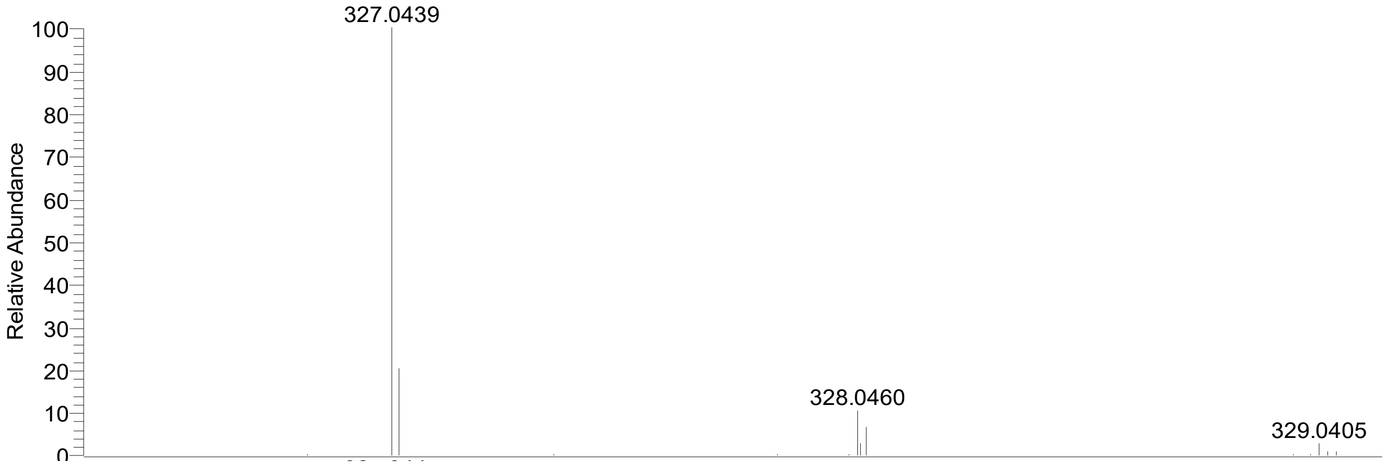

Figure S97: *Negative* HRMS spectrum of compound **9c**

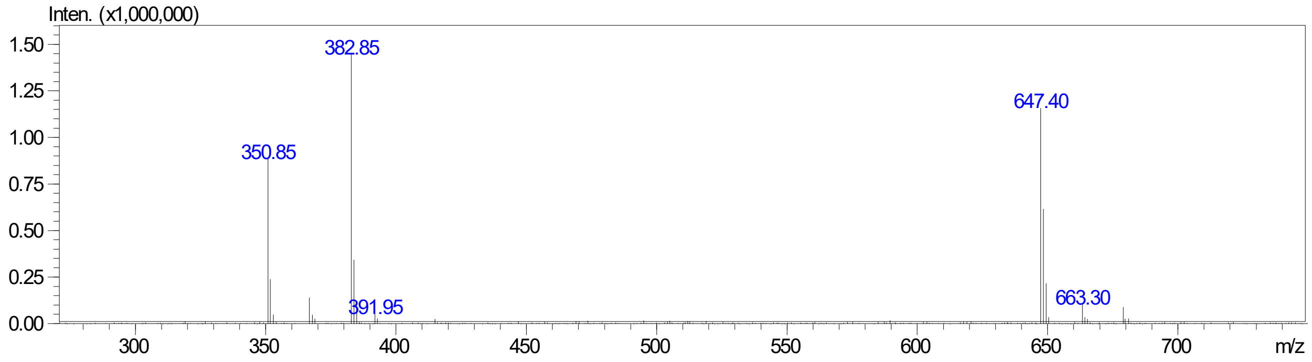

Figure S98: *Positive* LC-MS spectrum of compound **9c**

^{1}H NMR, ^{13}C NMR, HRMS and LCMS spectrums of compound **9d**

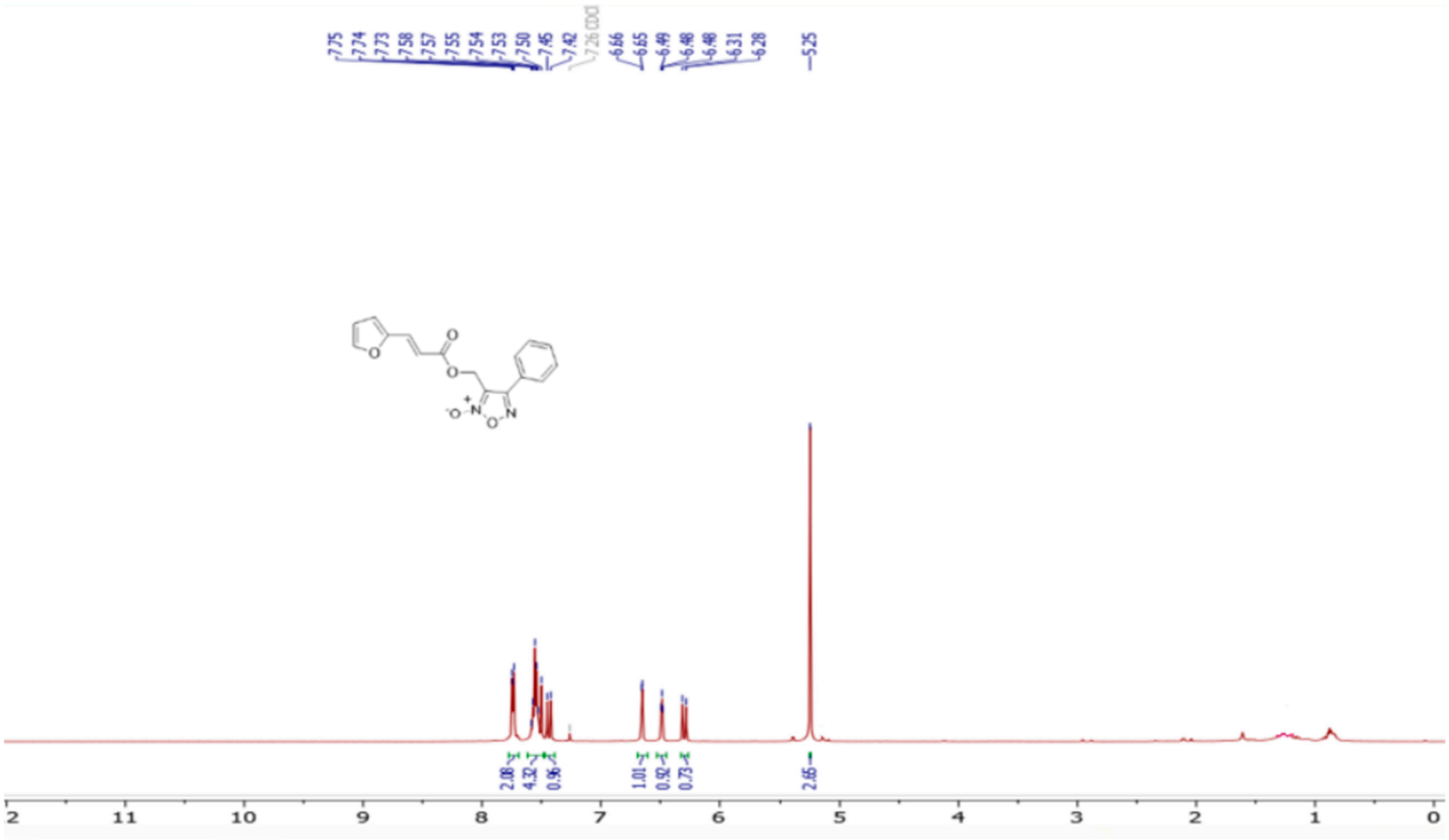

Figure S99: ^{1}H NMR spectrum of compound **9d**

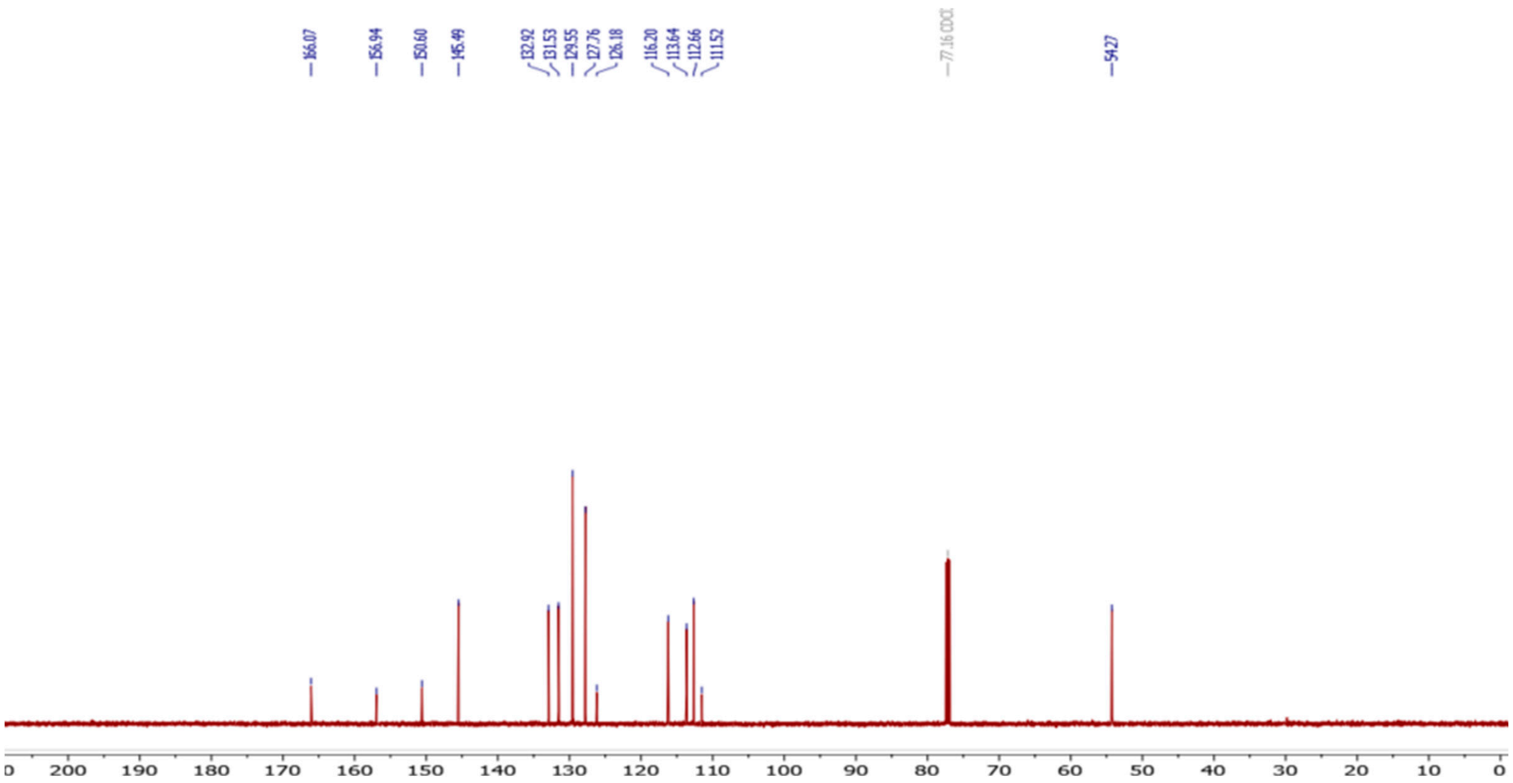

Figure S100: ^{13}C NMR spectrum of compound **9d**

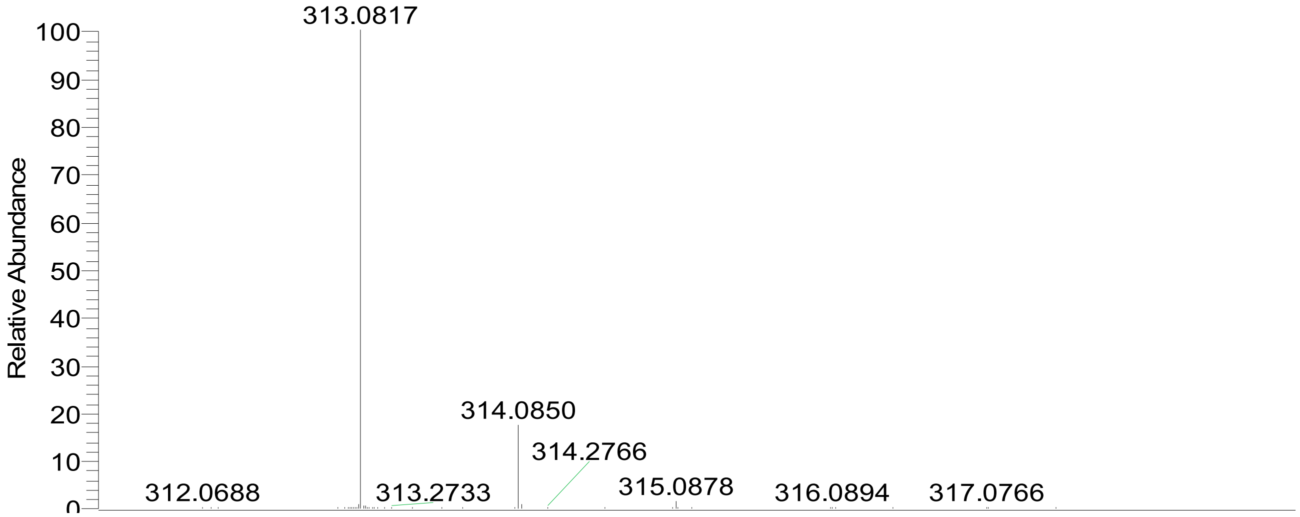

Figure S101: *Positive* HRMS spectrum of compound **9d**

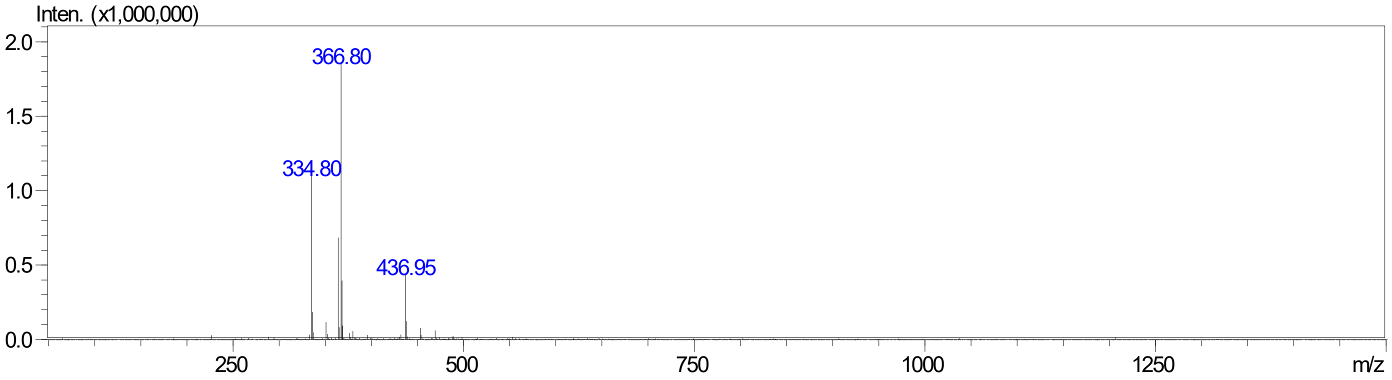

Figure S102: *Positive* LC-MS spectrum of compound **9d**

^{1}H NMR, ^{13}C NMR, HRMS and LCMS spectrums of compound **9e**

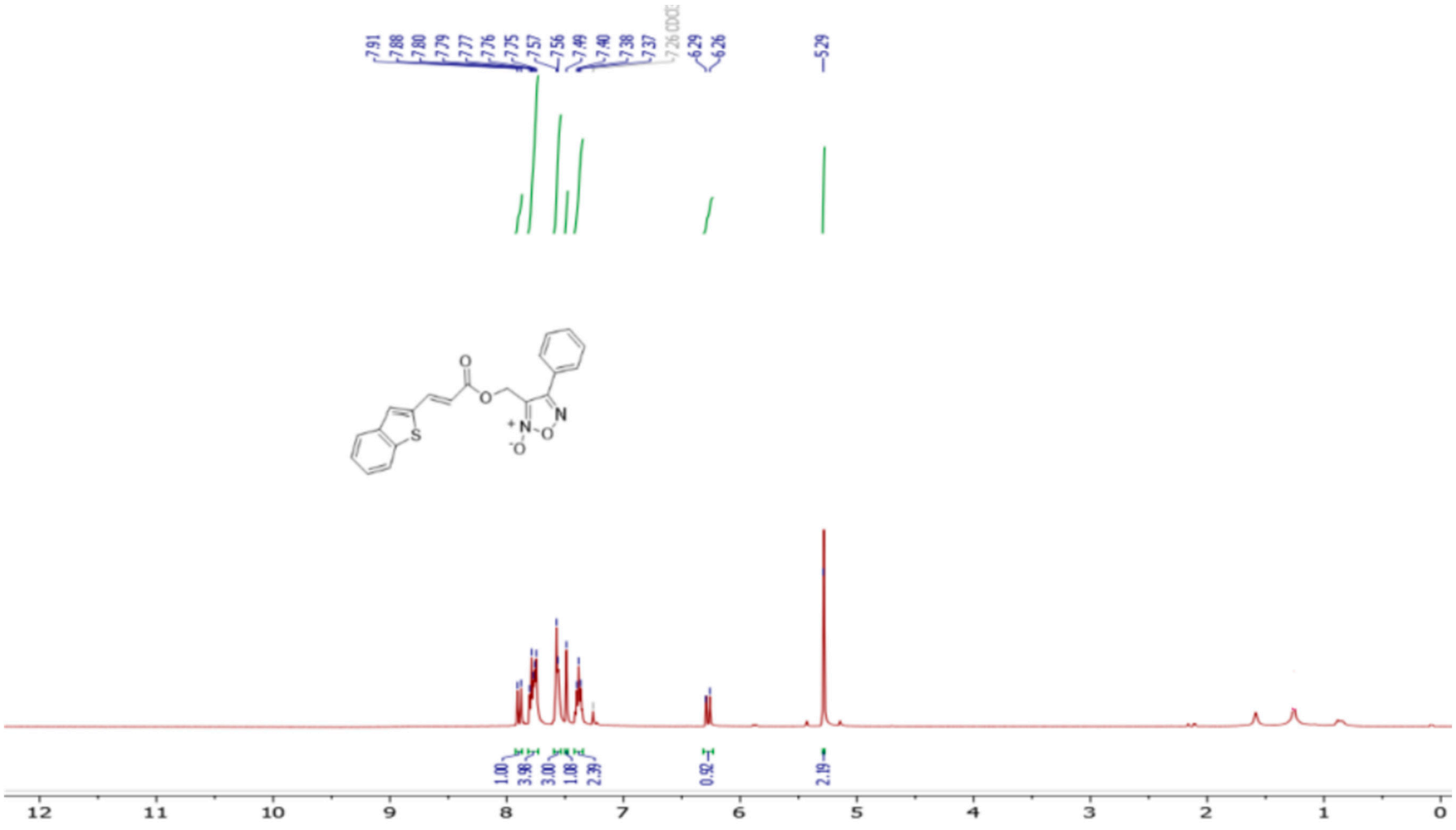

Figure S103: ^{1}H NMR spectrum of compound **9e**

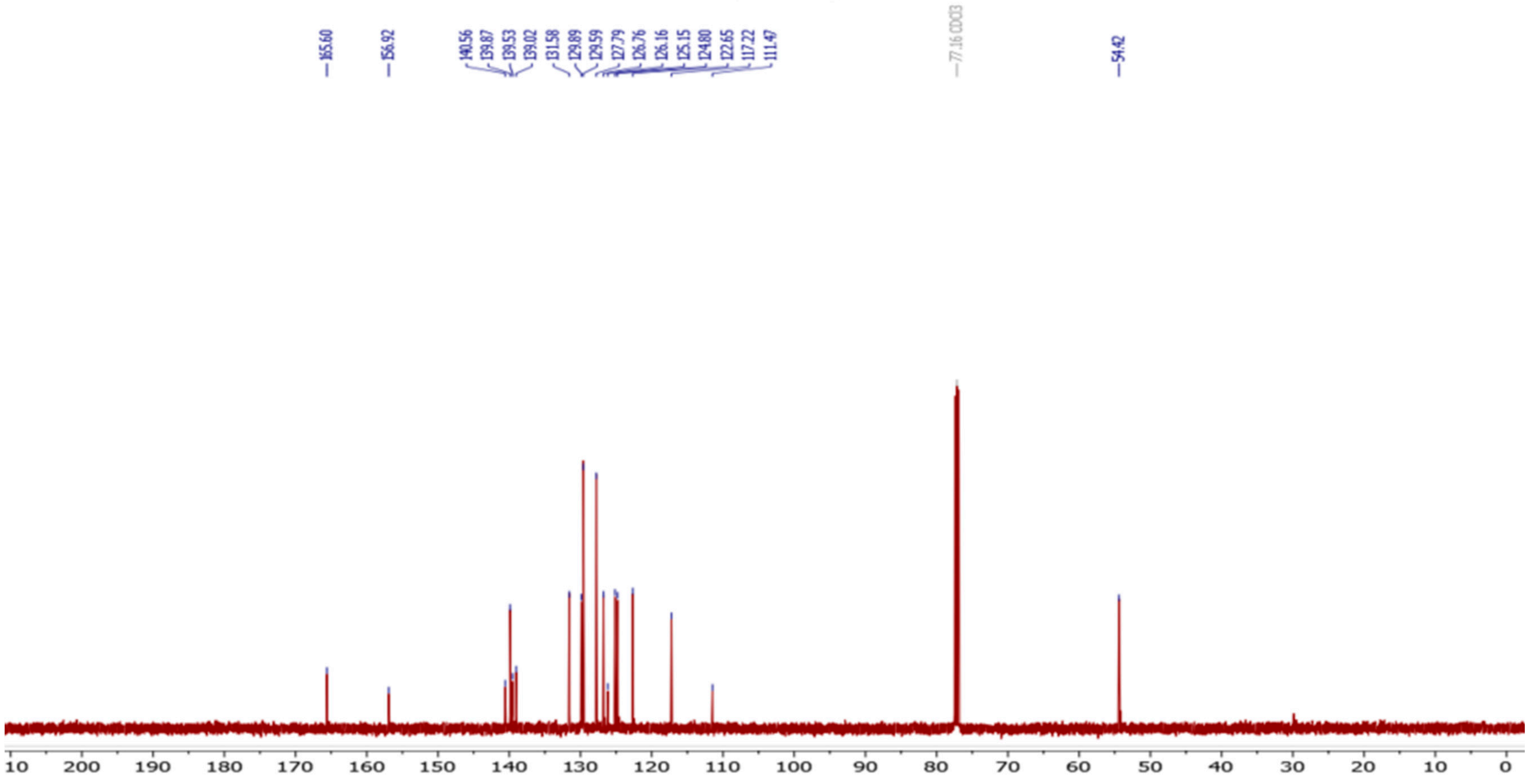

Figure S104: ^{13}C NMR spectrum of compound **9e**

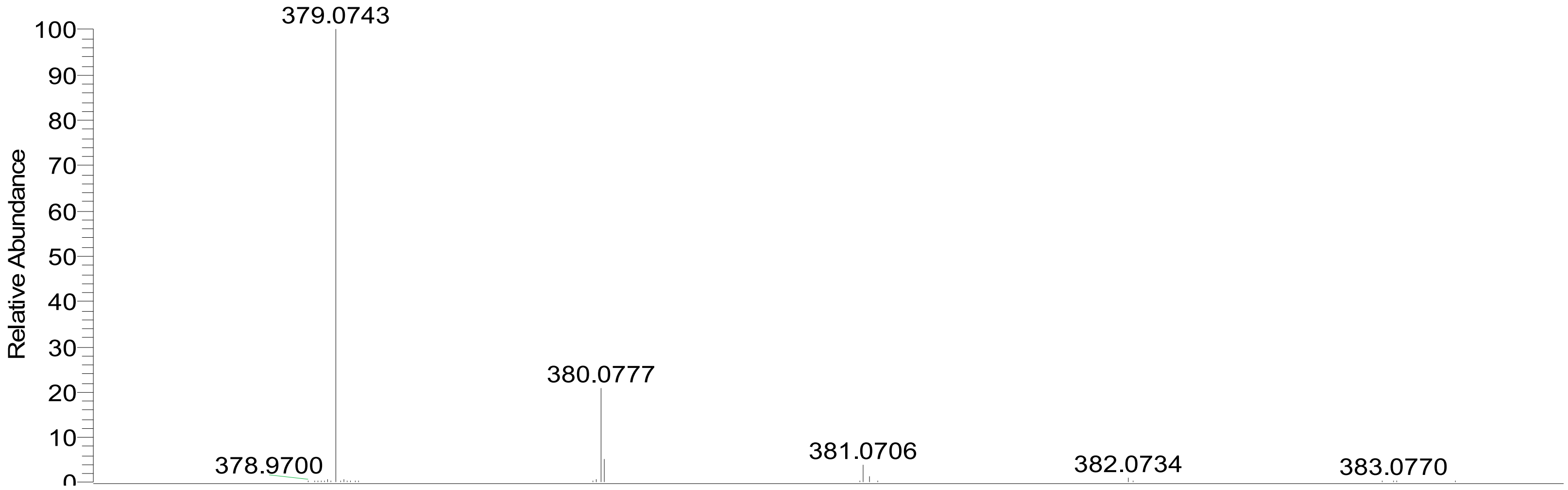

Figure S105: *Positive* HRMS spectrum of compound **9e**

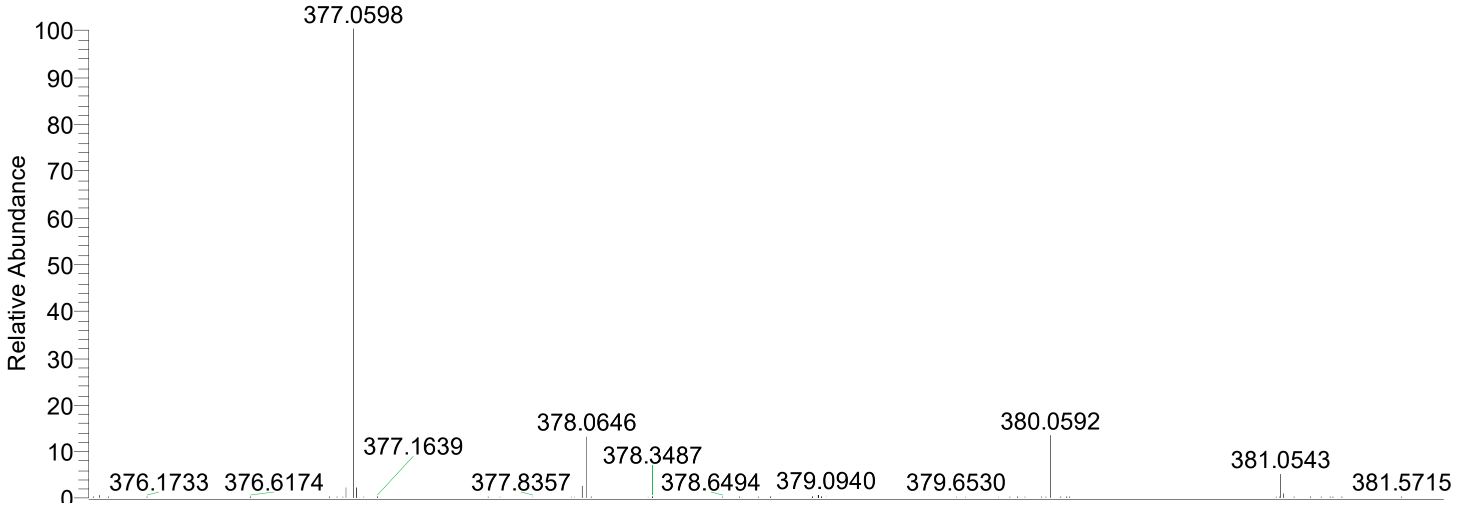

Figure S106: *Negative* HRMS spectrum of compound **9e**

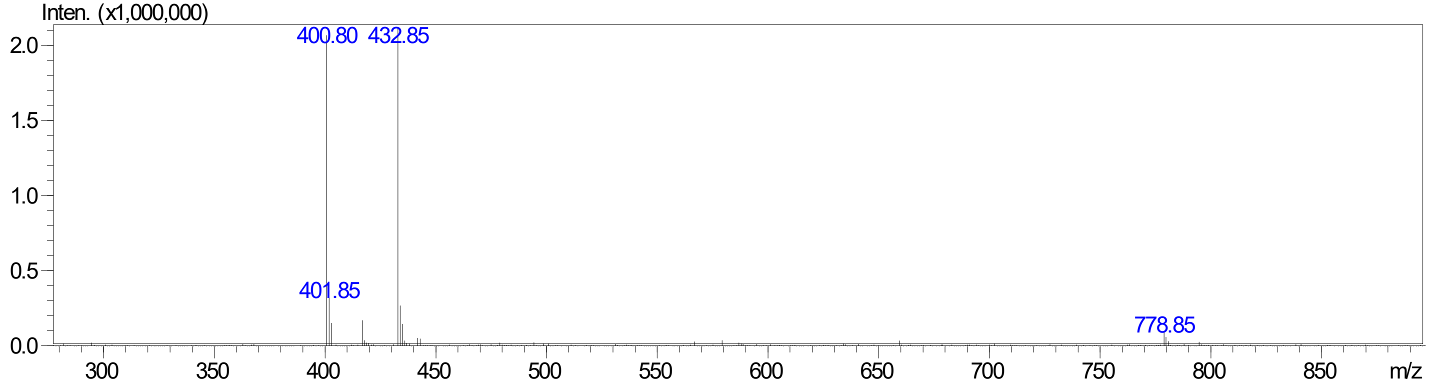

Figure S107: *Positive* LC-MS spectrum of compound **9e**

^{1}H NMR, ^{13}C NMR, HRMS and LCMS spectrums of compound **9f**

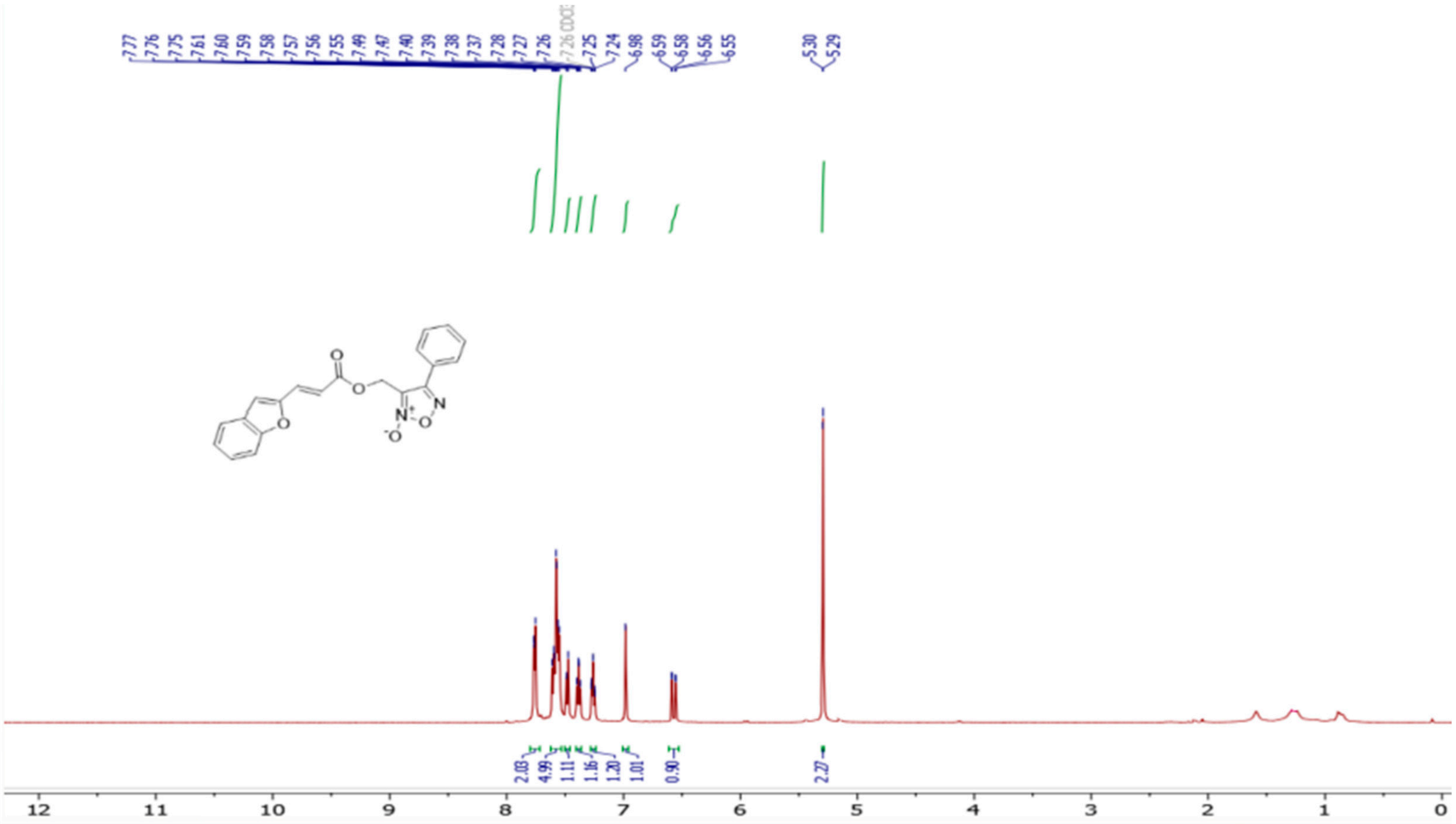

Figure S108: ^{1}H NMR spectrum of compound **9f**

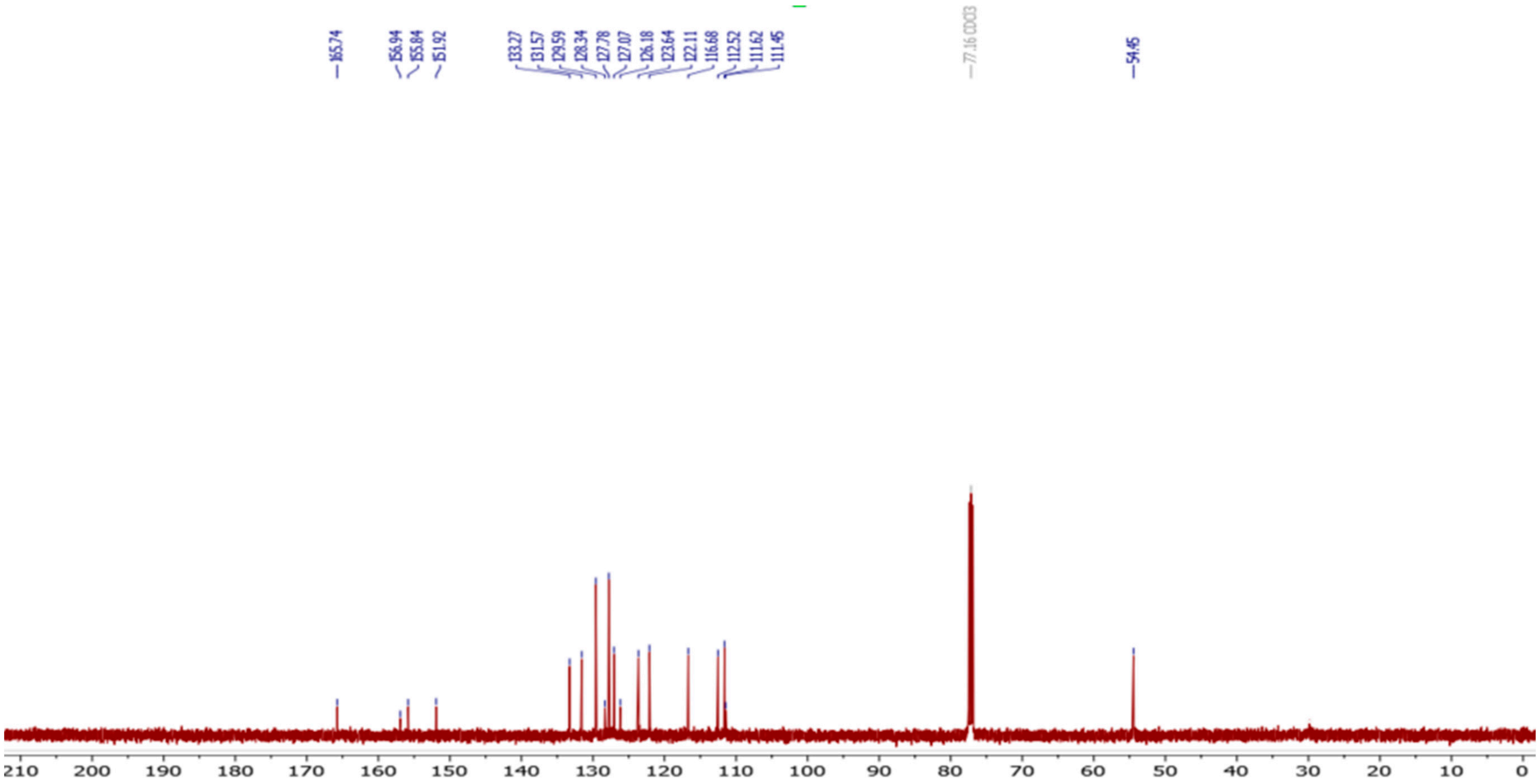

Figure S109: ^{13}C NMR spectrum of compound **9f**

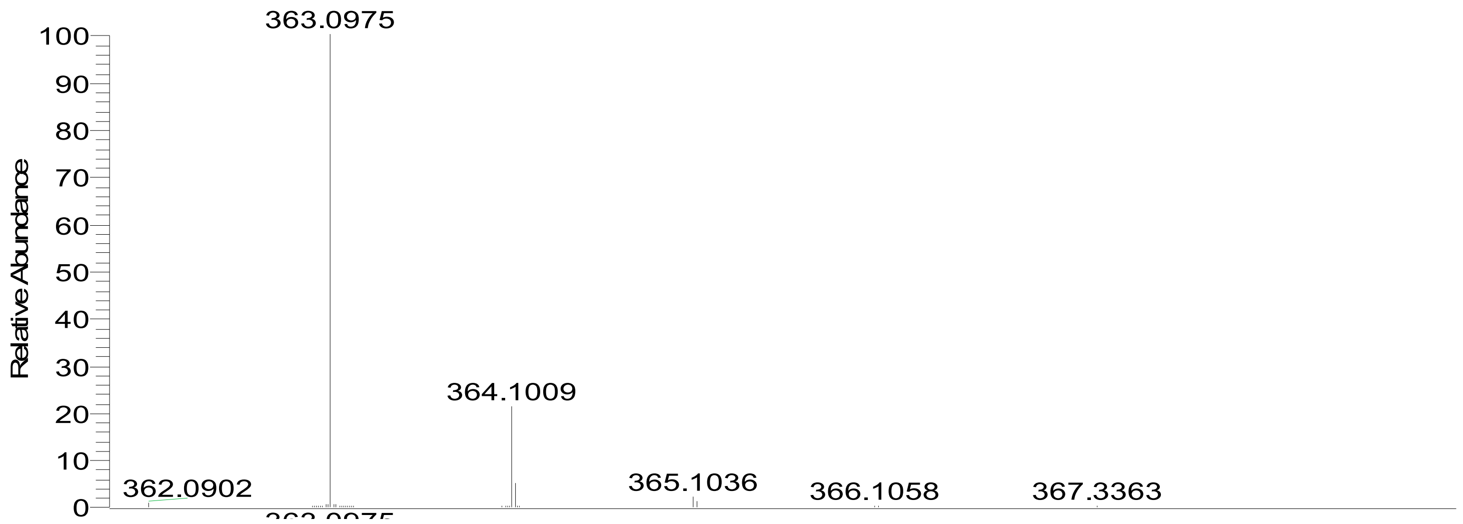

Figure S110: *Positive* HRMS spectrum of compound **9f**

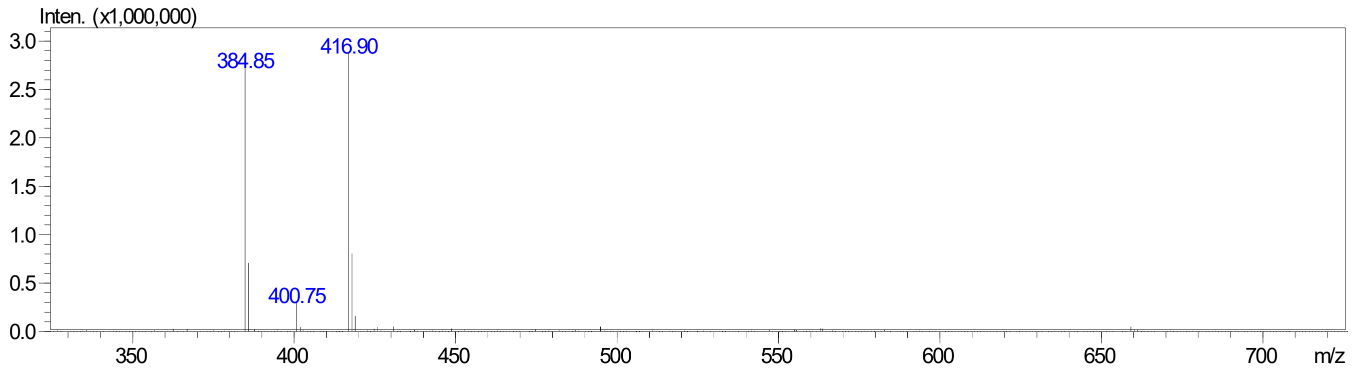

Figure S111: *Positive* LC-MS spectrum of compound **9f**

^{1}H NMR, ^{13}C NMR, HRMS and LCMS spectrums of compound **9g**

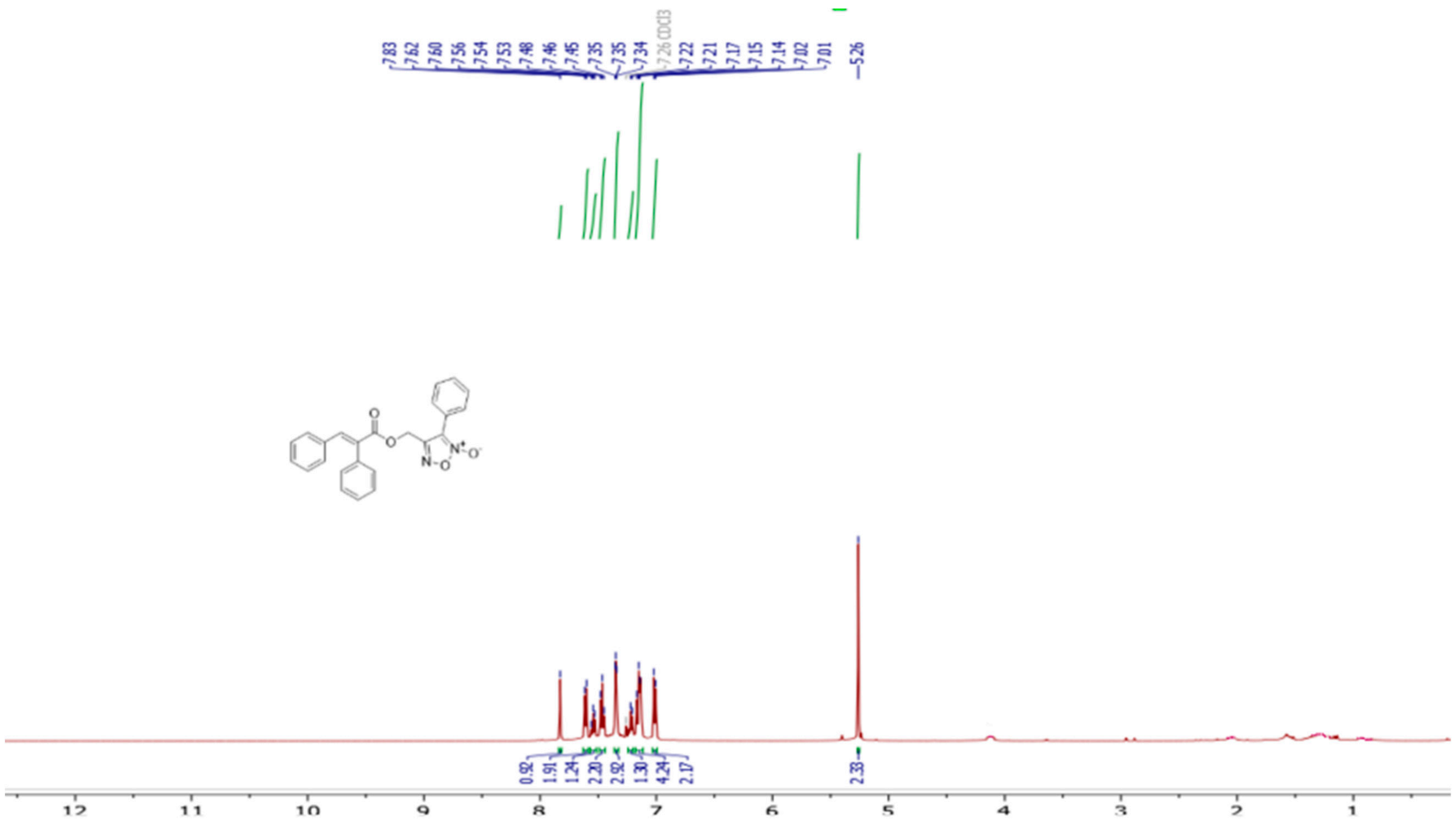

Figure S112: ^{1}H NMR spectrum of compound **9g**

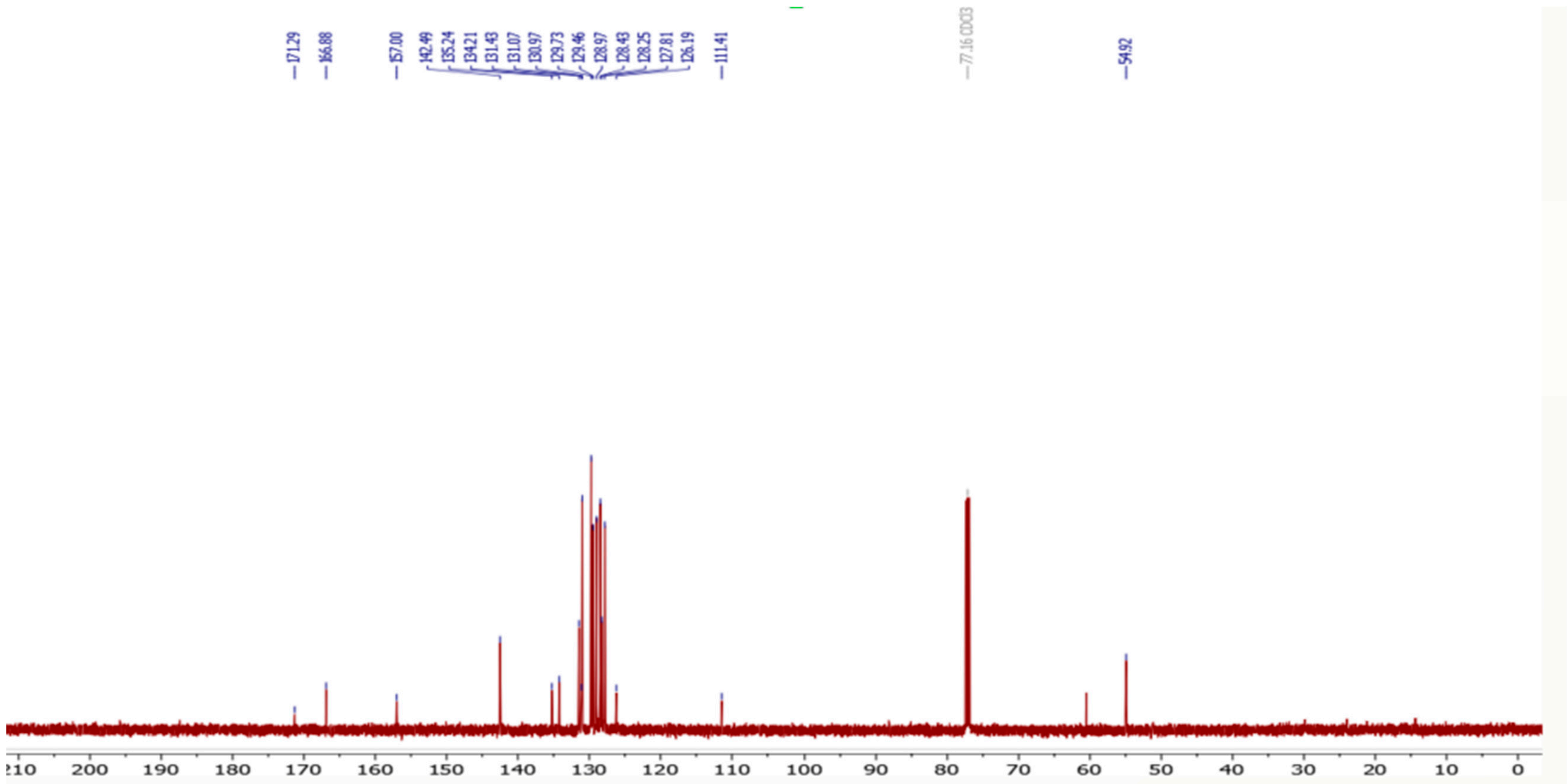

Figure S113: ^{13}C NMR spectrum of compound **9g**

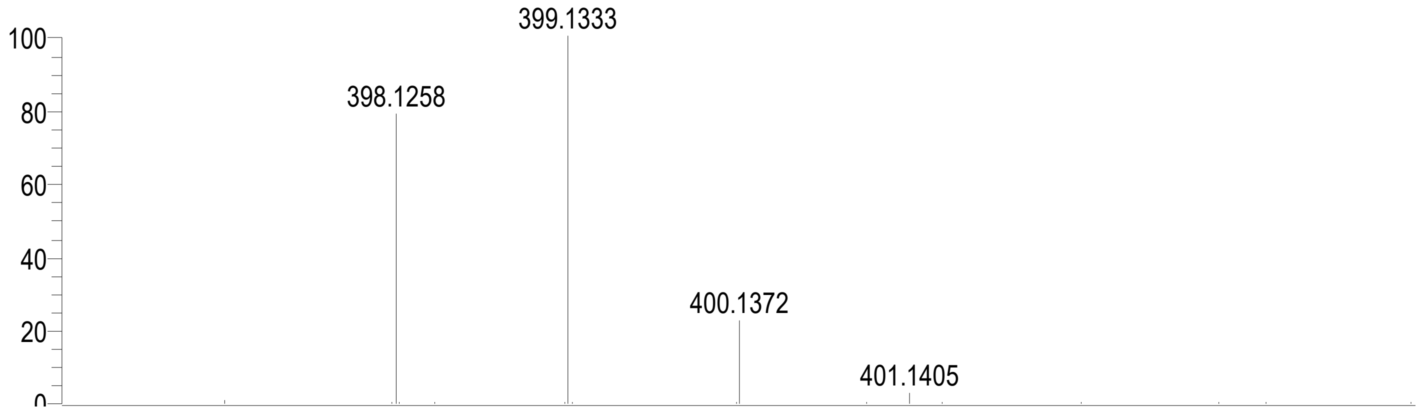

Figure S114: *Positive* HRMS spectrum of compound **9g**

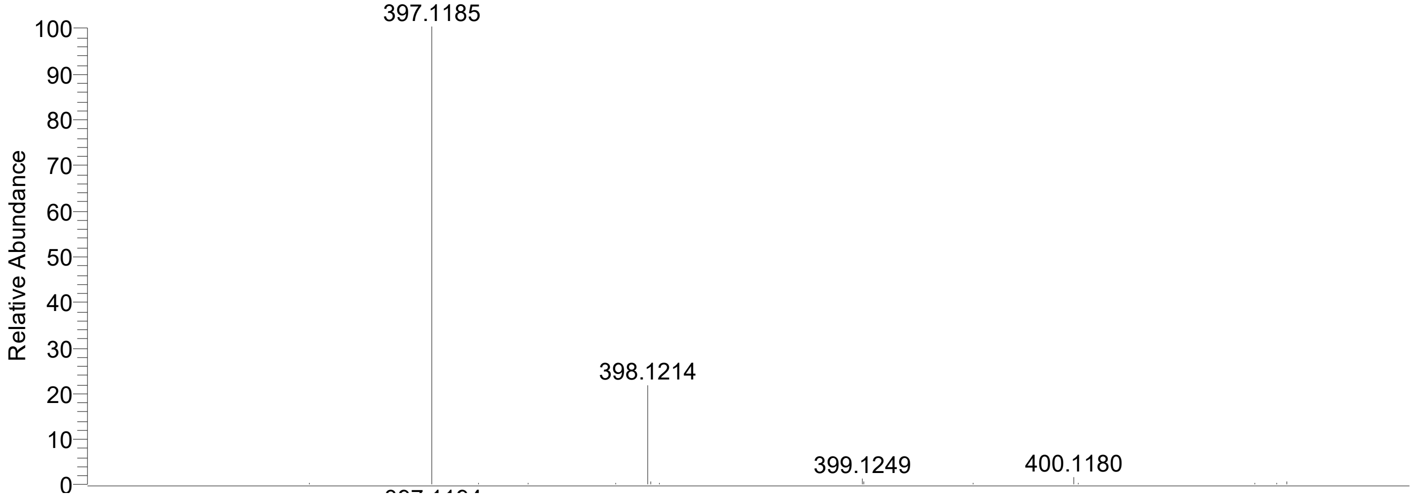

Figure S115: *Negative* HRMS spectrum of compound **9g**

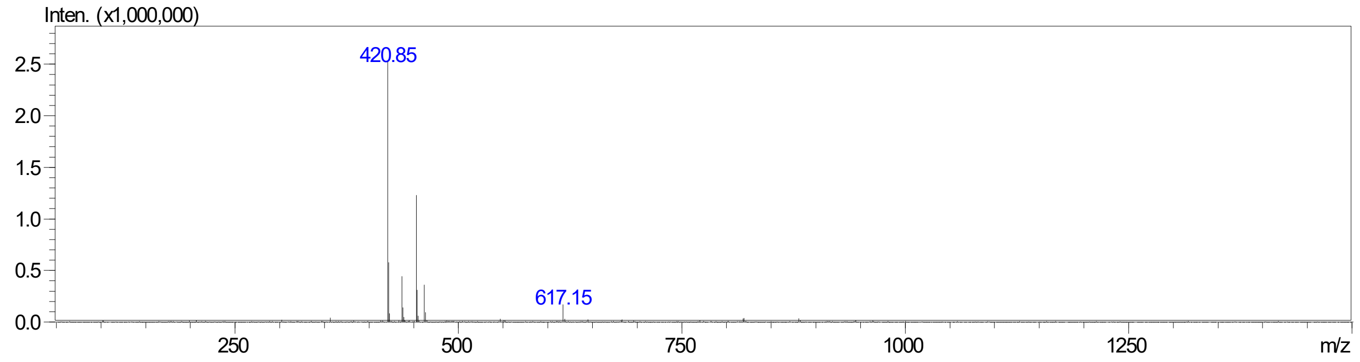

Figure S116: *Positive* LC-MS spectrum of compound **9g**

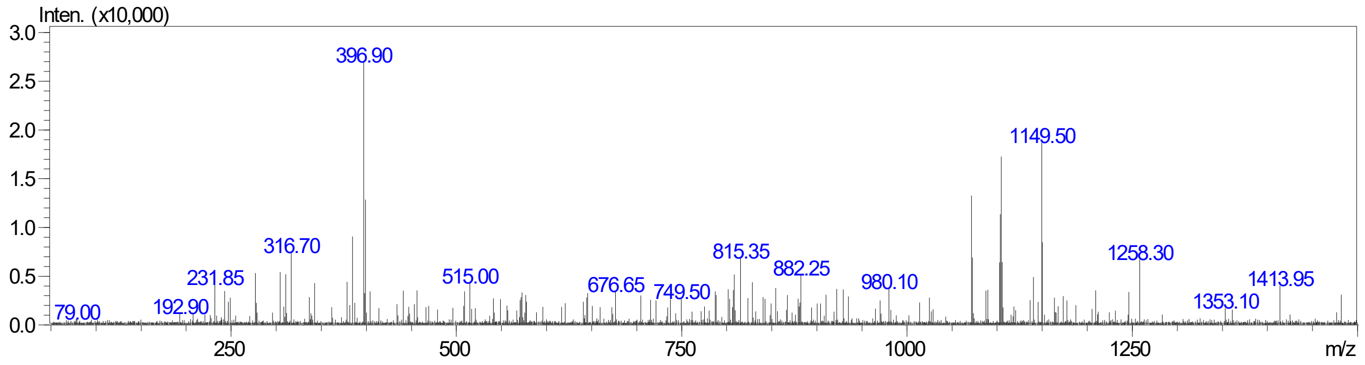

Figure S117: *Negative* LC-MS spectrum of compound **9g**

^{1}H NMR, ^{13}C NMR, HRMS and LCMS spectrums of compound **9h**

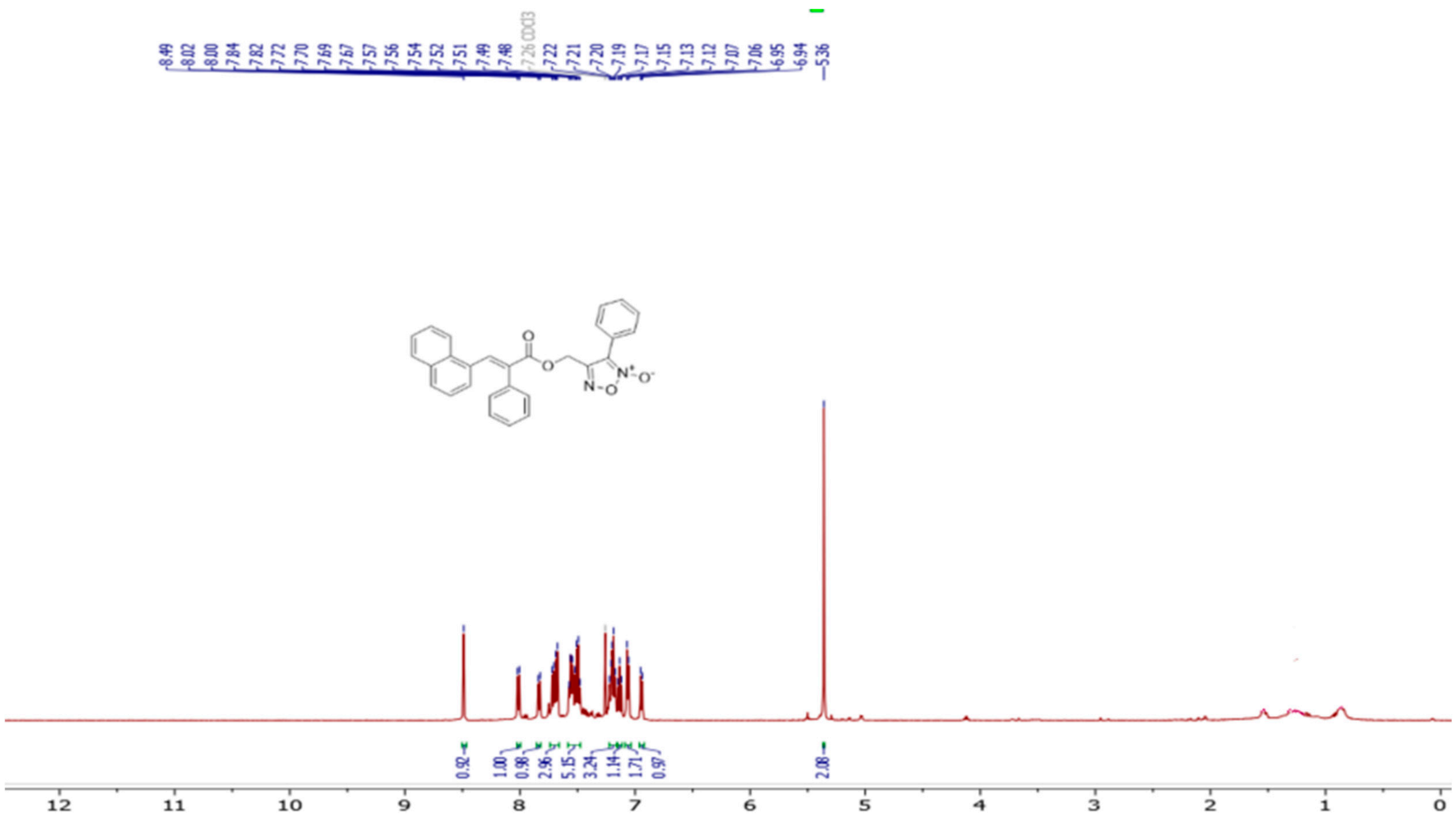

Figure S118: ^{1}H NMR spectrum of compound **9h**

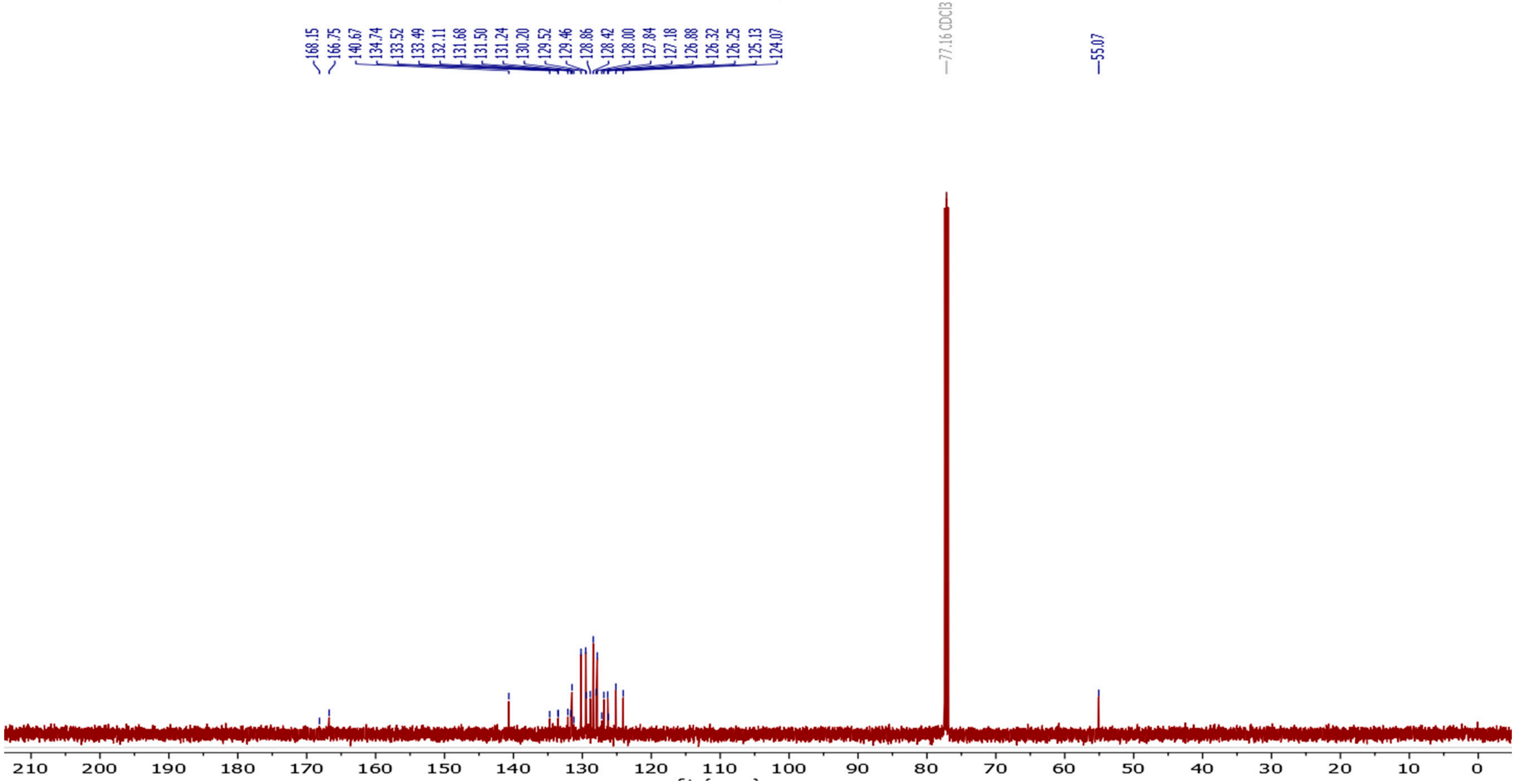

Figure S119: ^{13}C NMR spectrum of compound **9h**

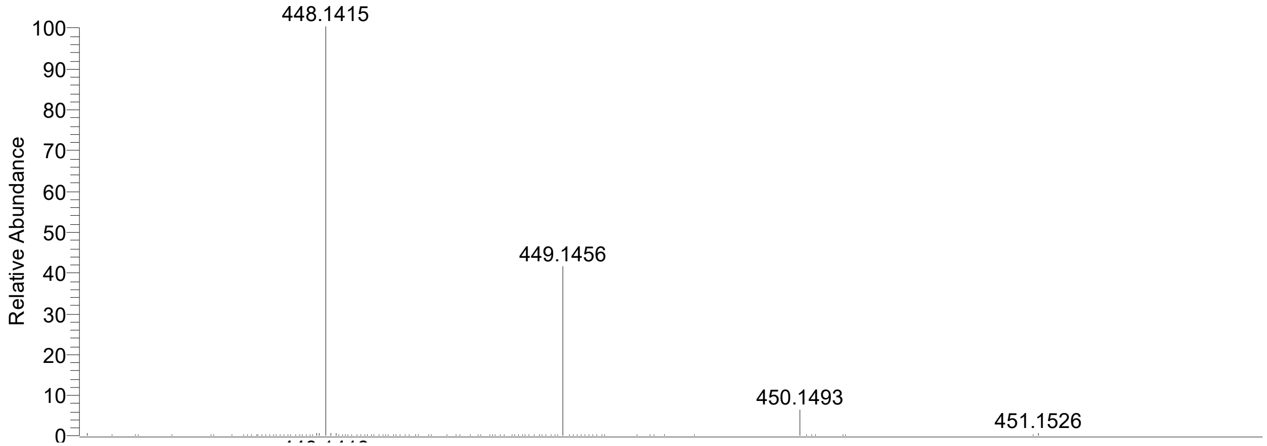

Figure S120: *Positive* HRMS spectrum of compound **9h**

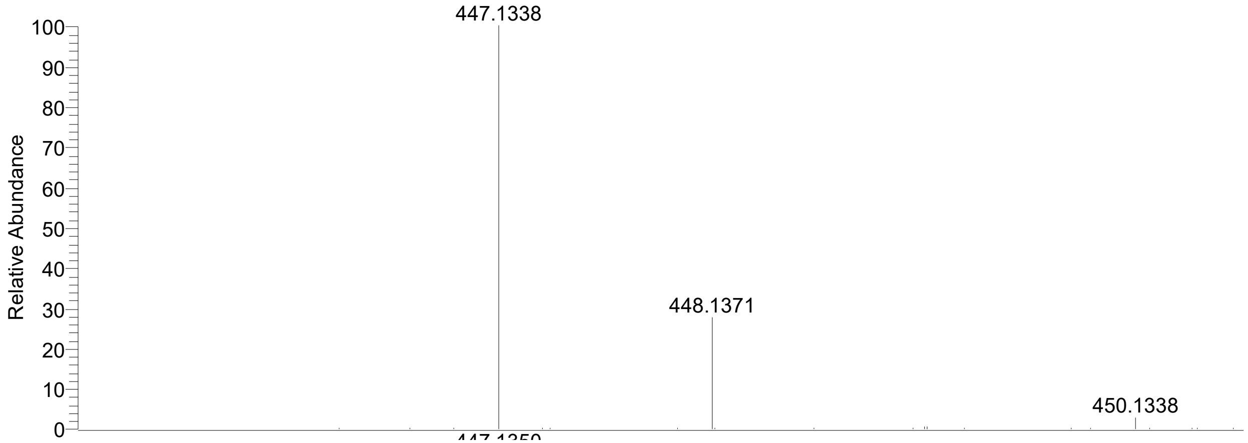

Figure S121: *Negative* HRMS spectrum of compound **9h**

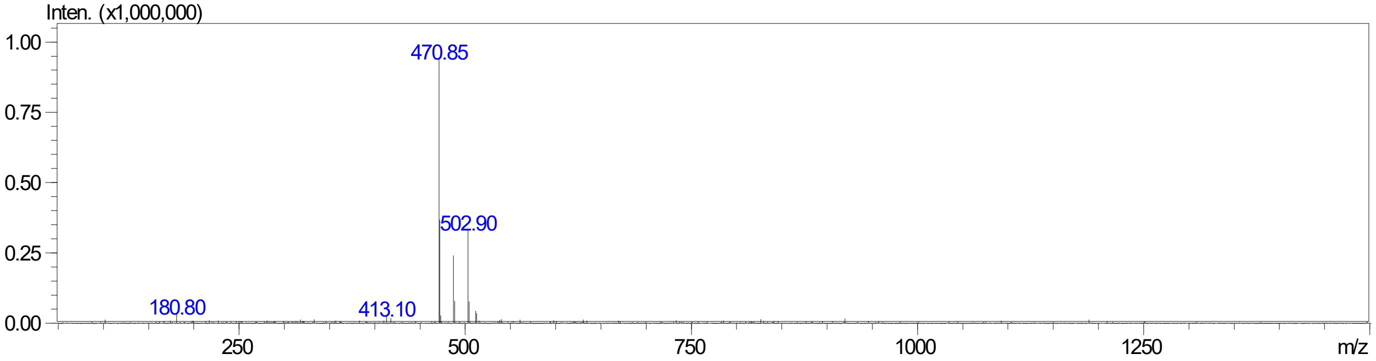

Figure S122: *Positive* LC-MS spectrum of compound **9h**

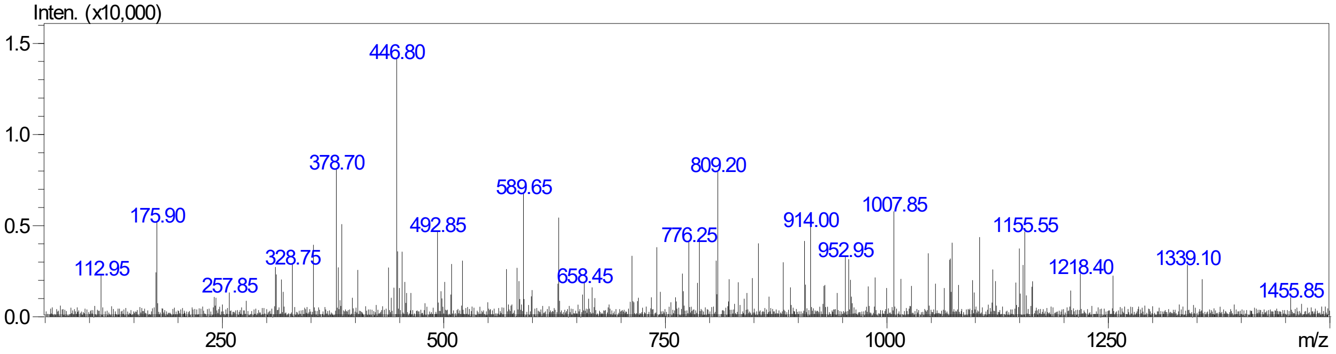

Figure S123: *Negative* LC-MS spectrum of compound **9h**

^{1}H NMR, ^{13}C NMR, HRMS and LCMS spectrums of compound **9i**

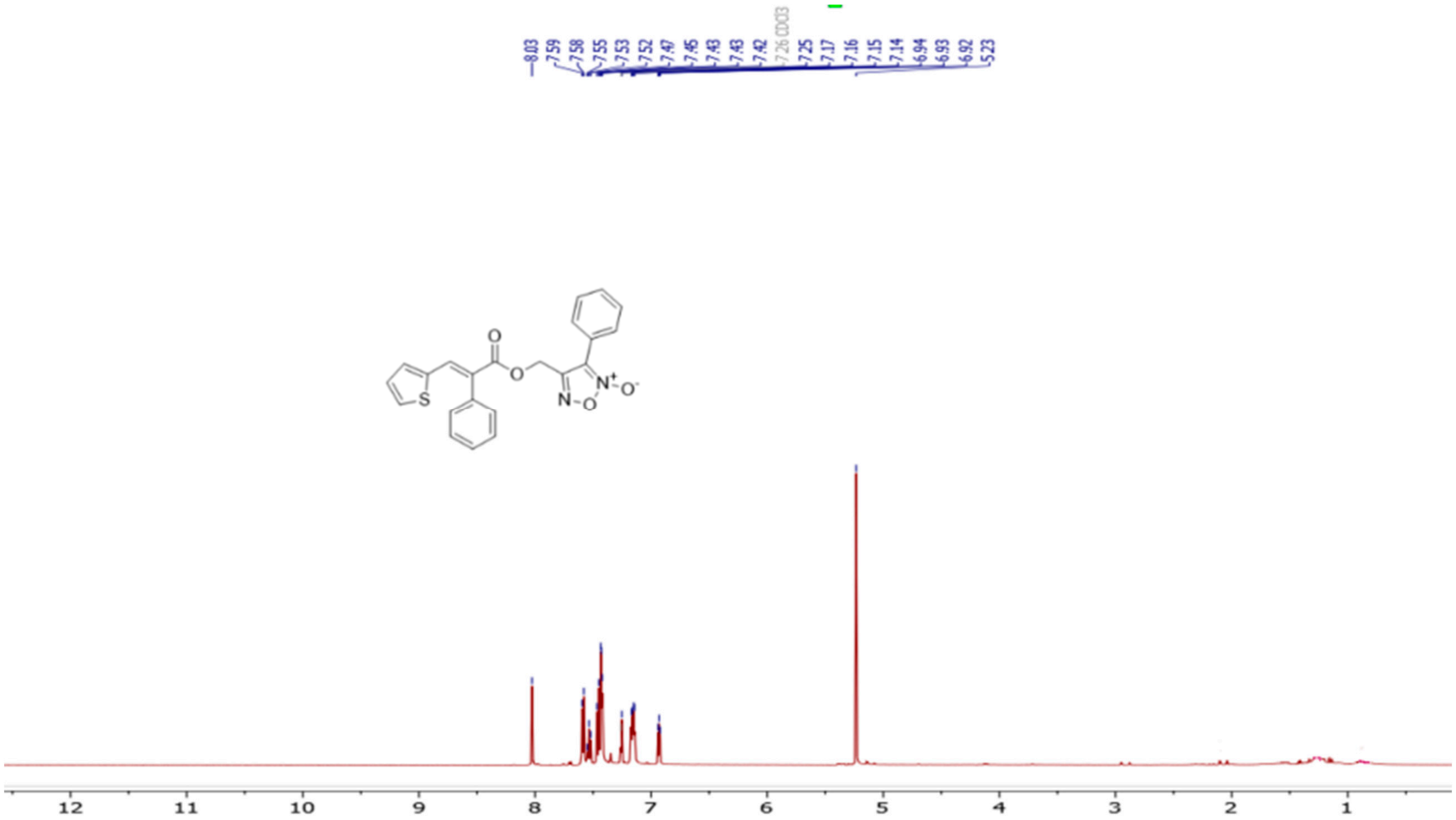

Figure S124: ^{1}H NMR spectrum of compound **9i**

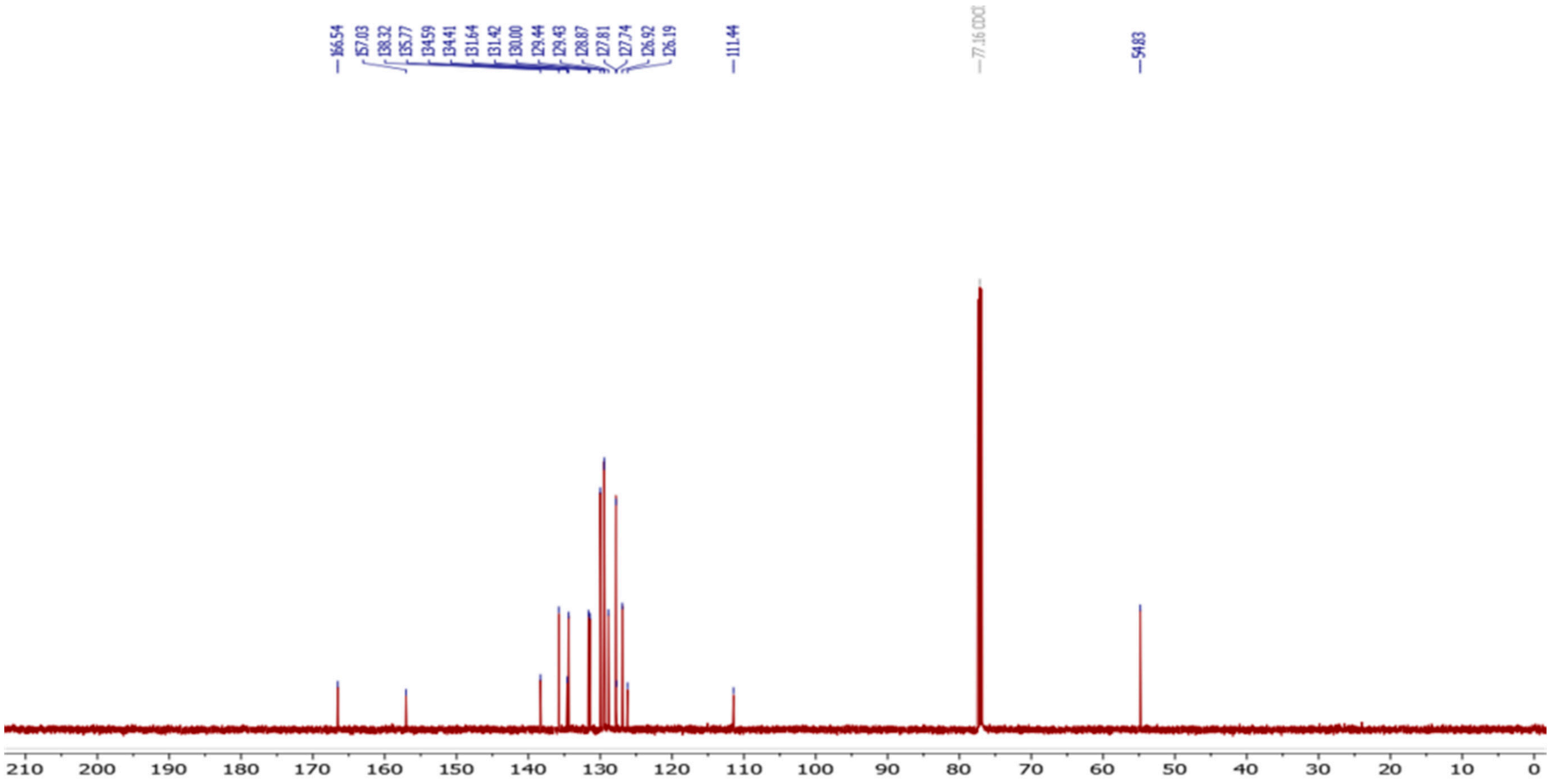

Figure S125: ^{13}C NMR spectrum of compound **9i**

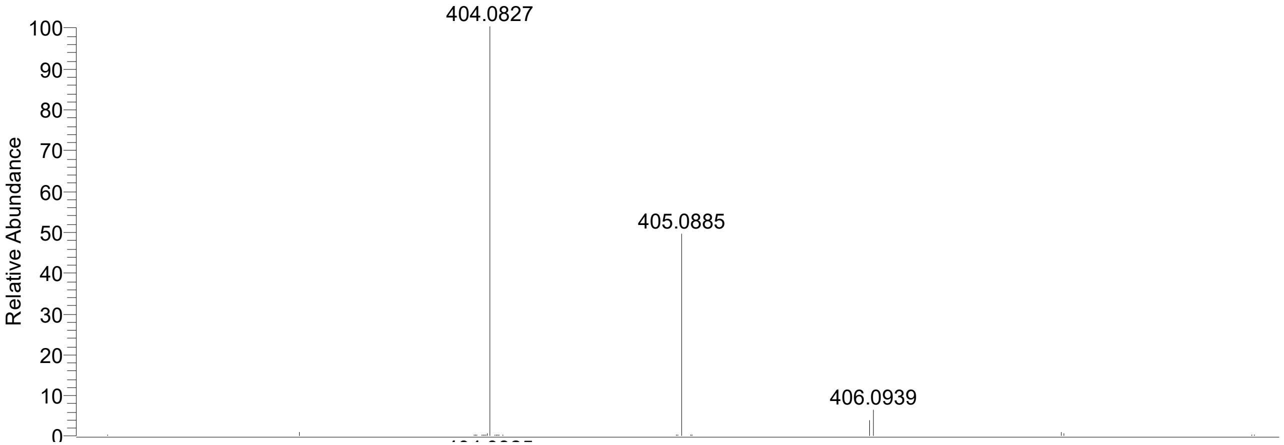

Figure S126: *Positive* HRMS spectrum of compound **9i**

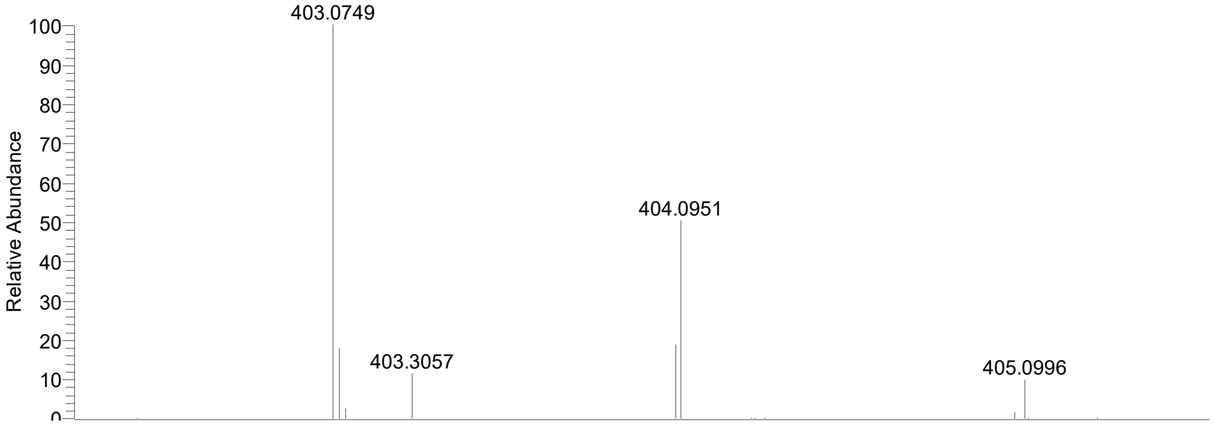

Figure S127: *Negative* HRMS spectrum of compound **9i**

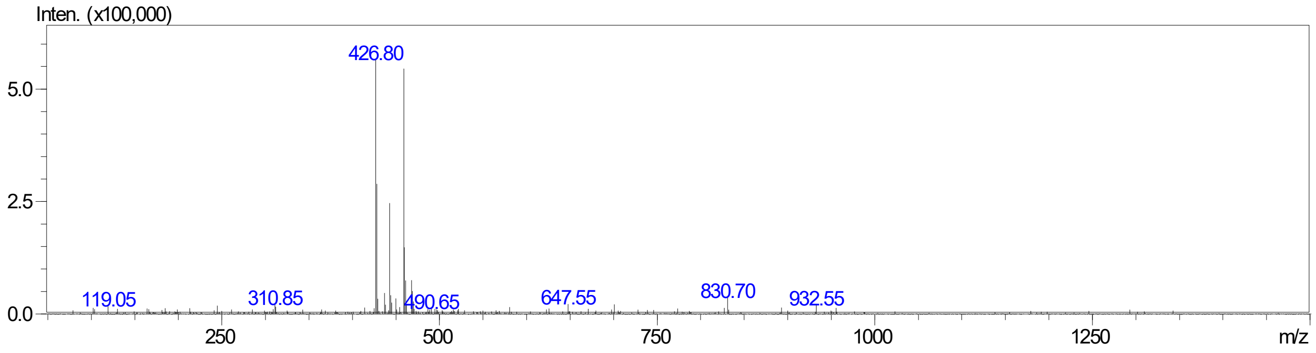

Figure S128: *Positive* LC-MS spectrum of compound **9i**

^{1}H NMR, ^{13}C NMR spectrums of compound **11**

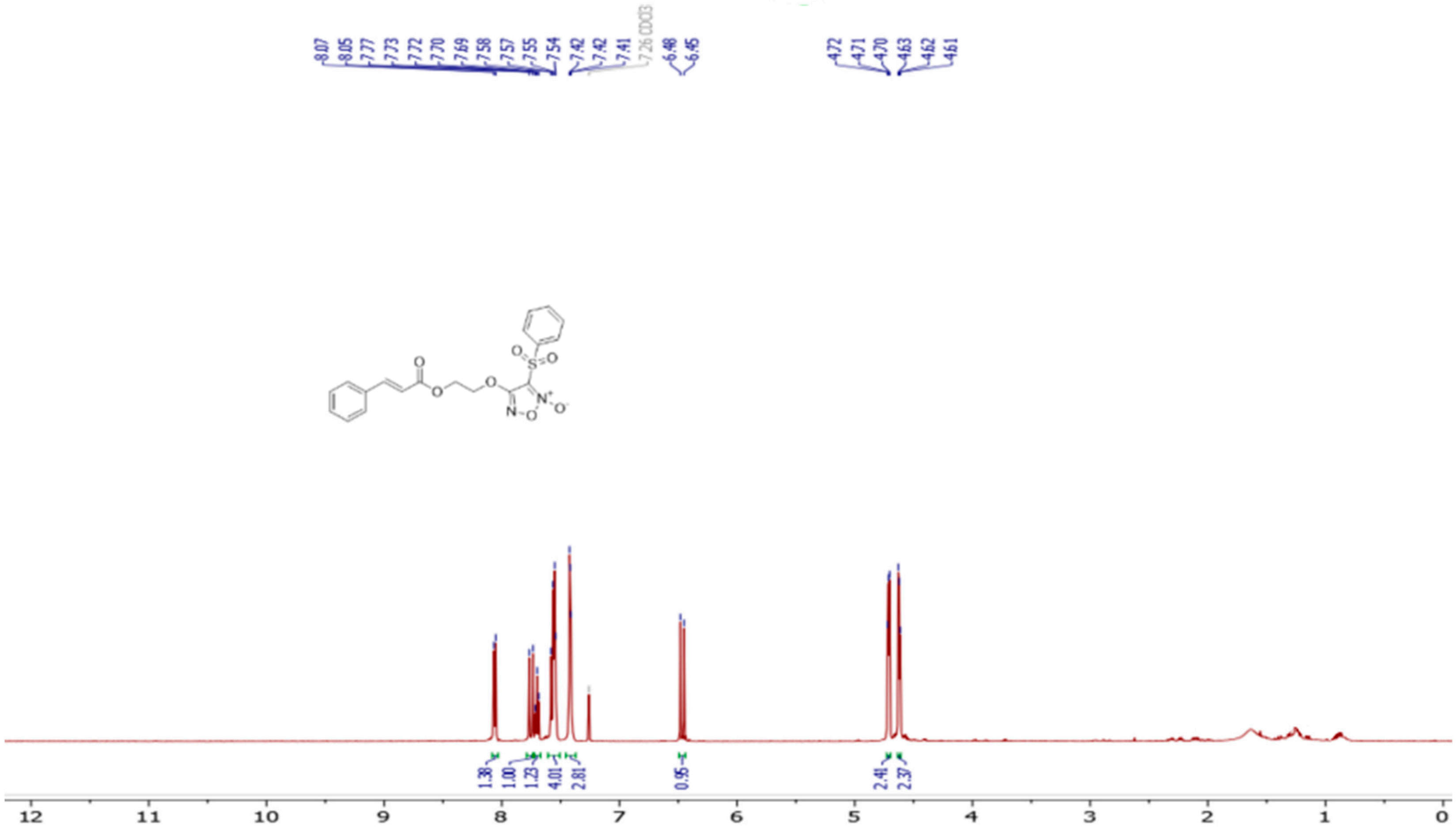

Figure S129: ^{1}H NMR spectrum of compound **11**

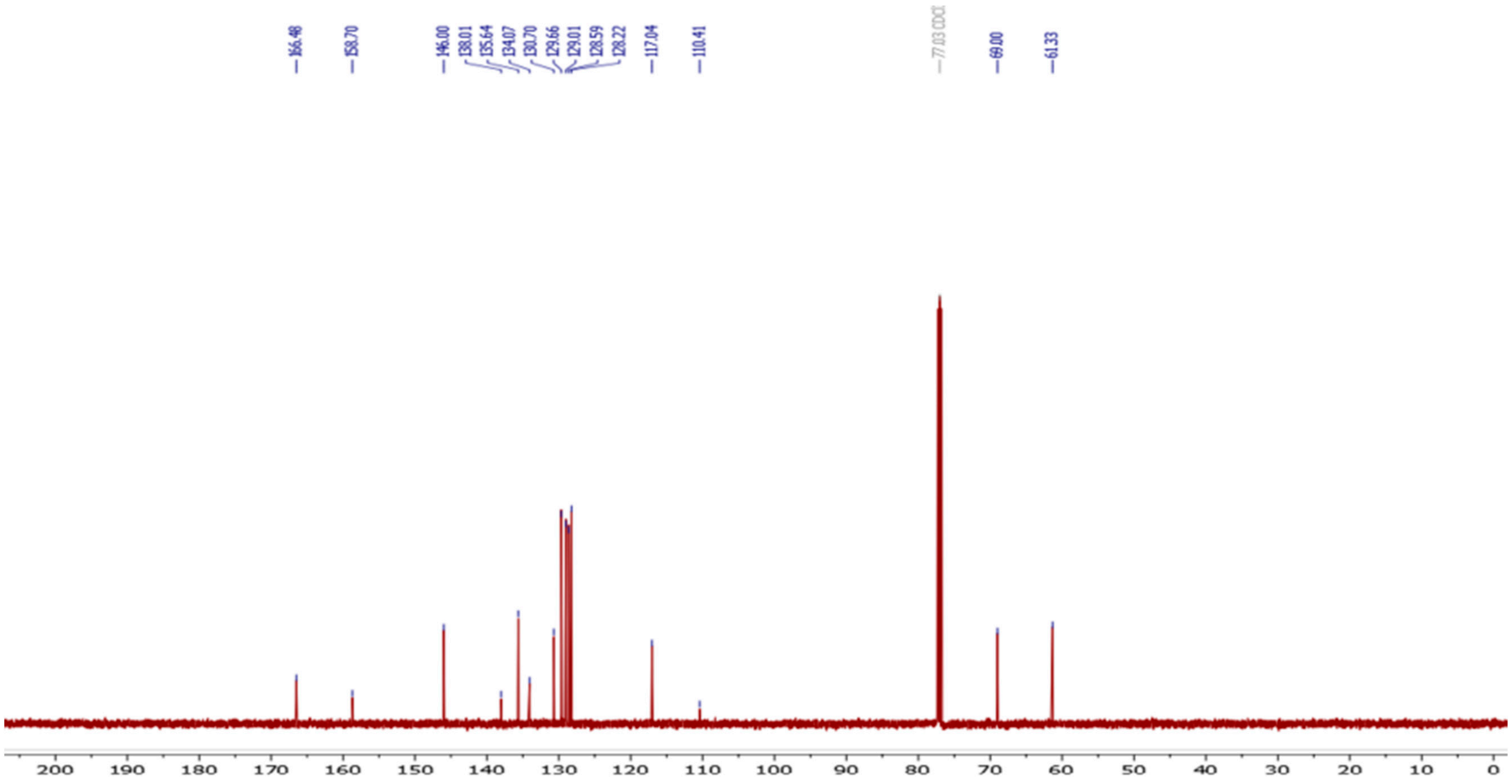

Figure S130: ^{13}C NMR spectrum of compound **11**
